# Conjugates of 1'-Aminoferrocene-1-carboxylic Acid and Proline: Synthesis, Conformational Analysis and Biological Evaluation

**DOI:** 10.3390/molecules190812852

**Published:** 2014-08-21

**Authors:** Monika Kovačević, Krešimir Molčanov, Kristina Radošević, Višnja Gaurina Srček, Sunčica Roca, Alan Čače, Lidija Barišić

**Affiliations:** 1 Department of Chemistry and Biochemistry, Faculty of Food Technology and Biotechnology, Pierottijeva 6, Zagreb 10000, Croatia; E-Mails: mkovacevic@pbf.hr (M.K.); alan.cace@gmail.com (A.C.); 2 Division of Physical Chemistry, Ruđer Bošković Institute, Bijenička cesta 54, Zagreb 10000, Croatia; E-Mail: Kresimir.Molcanov@irb.hr; 3 Department of Biochemical Engineering, Laboratory for Cell Technology, Aplication and Biotransformation, Faculty of Food Technology and Biotechnology, Pierottijeva 6, Zagreb 10000, Croatia; E-Mail: vgaurina@pbf.hr; 4 NMR Centre, Ruđer Bošković Institute, Bijenička cesta 54, Zagreb 10000, Croatia; E-Mail: sroca@irb.hr

**Keywords:** ferrocene, proline, peptidomimetic, intramolecular hydrogen bond, conformational analysis, X-ray crystallography, anticancer activity

## Abstract

Our previous studies showed that alteration of dipeptides Y-Fca-Ala-OMe (**III**) into Y-Ala-Fca-OMe (**IV**) (Y = Ac, Boc; Fca = 1'-aminoferrocene-1-carboxylic acid) significantly influenced their conformational space. The novel bioconjugates Y-Fca-Pro-OMe (**1**, Y = Ac; **2**, Y = Boc) and Y-Pro-Fca-OMe (**3**, Y = Boc; **4**, Y = Ac) have been prepared in order to investigate the influence of proline, a well-known turn-inducer, on the conformational properties of small organometallic peptides with an exchanged constituent amino acid sequences. For this purpose, peptides **1**–**4** were subjected to detailed spectroscopic analysis (IR, NMR, CD spectroscopy) in solution. The conformation of peptide **3** in the solid state was determined. Furthermore, the ability of the prepared conjugates to inhibit the growth of estrogen receptor-responsive MCF-7 mammary carcinoma cells and HeLa cervical carcinoma cells was tested.

## 1. Introduction

Peptides and proteins play crucial role in cellular signalling processes and account for numerous targets in medicine [1]. Despite their enormous diversity in biological function and structure, the use of peptides and proteins as drugs is limited due to: (*i*) their low metabolic stability towards proteolysis in the gastrointestinal tract and in serum; (*ii*) poor absorption after oral administration; (*iii*) rapid excretion through liver and kidneys and (*iv*) undesired effects caused by interaction of the conformationally flexible peptides with various receptors. The improvement or alteration of unfavourable structural and biological properties of peptide and proteins can be realized by using their mimetics [2]. A peptidomimetic is a peptide or non-peptide compound capable of mimicking the properties or biological activity of a peptide owing to the presence of secondary structure and other features that are analogous to those of the original peptide [2,3,4,5]. There are different approaches in the design and synthesis of peptidomimetics, *i*.*e*., the incorporation of the conformational constraints (non-peptide moieties) in order to reduce conformational flexibility, the amide bond replacement, the conjugation of peptides with small molecules and the backbone cyclization. 1,1'-Disubstituted ferrocene templates [ferrocene-1,1'-dicarboxylic acid (Fcd), ferrocene-1,1'-diamine (Fcda) and 1'-aminoferrocene-1-carboxylic acid (Fca)] were recognized as useful and efficient bioorganometallic constraints designed to induce chirally organized structures upon conjugation with natural amino acids {Fn-[CO-(AA)_m_-OMe]_2_ (**I**) [6,7,8,9,10,11,12,13]}, {Fn-[NH-(AA)_m_-OMe]_2_ (**II**) [14,15]}, {Y-Fca-(AA)_m_-OMe (**III**) [16,17,18,19,20,21]} and {Y-(AA)*_n_-*Fca-OMe (**IV**) [22]} [Fn = ferrocenylene; AA = amino acid; Y = di-*tert-*butyldicarbonate (Boc), acetamide (Ac); *m* = 1, 2; *n* = 1]. Namely, the introduction of the chiral peptide chains into the ferrocene scaffold enables their communication through intramolecular hydrogen bonds required for 3D structure formation and function of biological systems. Recently, Donoli *et al*. reported intramolecularly hydrogen-bonded series of 3_10_-helical peptides of different length containing terminal ferrocenyl units and based on the strongly foldameric achiral α-aminoisobutyric (Aib) acid [23,24].

Our group demonstrated that the attachment of alanine-containing sequences at *N*- or *C*- terminus of Fca [25] lead to peptidomimetics **III** [16,17,18,19,20,21] and **IV** [22] able to adopt key structural features of protein secondary structure. Certainly, the number and position of hydrogen bond donating sites as well as protecting groups on the N- and C-termini strongly influenced their conformational preferences. The peptide **III** was stabilized in an interchain intramolecular manner, *i*.*e*., 9-membered NH_Fca_···OC_Ala_ (**IIIA**) and 8-membered NH_Ala_···OC_Y_ hydrogen-bonded rings (**IIIB**). Upon the alteration of peptides **III** to **IV**, hydrogen bonding pattern was quite perturbed. Thereat, NH_Fca_ was engaged in intrachain manner through contacts with hydrogen-bond-accepting acetamide carbonyl groups (γ-turn, **IVA**) or alanine nitrogen (5-membered hydrogen-bonded ring, **IVB**). The interchain IHB, a motif common for peptides **III**, was realized in conformer **IVC** through 9-membered NH_Ala_···OC_Fca_ hydrogen-bonded ring (Table 1).

**Table 1:** Hydrogen bonding patterns of peptides derived from Fca and Ala (the dotted lines represent the hydrogen bonds).

Type	Hydrogen Bonding Patterns *			
**III**	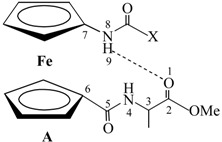		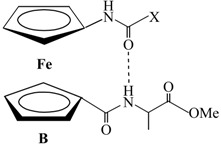	
**IV**	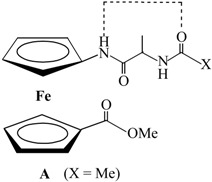	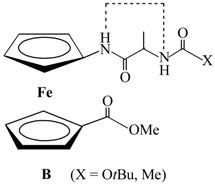		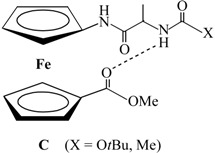

* The hydrogen bonding patterns were systematized in accordance with the nomenclature of 1,n'-disubstituted ferrocene peptides [26].

The present study covers the synthesis, conformational features and biological evaluation of Y-Fca-Pro-OMe (**V**) [**1**, Y = Ac; **2**, Y = Boc] and Y-Pro-Fca-OMe (**VI**) [**3**, Y = Boc; **4**, Y = Ac], analogues of peptides **III** and **IV**. The role of proline in the process of peptide folding, which is necessary to achieve the physiologically active 3D structure, is attributed to its unique structural properties. While the most peptide bonds exist in *trans* configuration to keep the side chains apart, the peptide imide bond that involves the NH group of the rigid five-membered pyrrolidine ring appears in both *trans* and *cis* forms. *Cis-trans* isomerization of proline-containing sequences affects numerous biological processes performed by peptides and proteins (cellular uptake, oligomerization, folding, catalysis). Furthermore, proline has been recognized as a turn inducer, e.g., proline can induce β-turns, especially if preceded or followed by aromatic amino acids [27,28,29].

Considering that peptide sequence and protecting groups at *N*- and *C*-terminal positions strongly influence the conformational features, molecular recognition, assembly and folding of small peptides [30], our goal is to determine the impact of proline unit on the conformational and biological properties of the corresponding bioorganometallics **V** and **VI** depending on amino acid sequence and employed protecting groups (Boc or Ac).

## 2. Results and Discussion

### 2.1. Chemistry

Ac-Fca-Pro-OMe (**1**) and Boc-Fca-Pro-OMe (**2**) were prepared by coupling of *N*-protected Fca [25] with *C*-protected proline following the well-established procedure [16,17,18,19,20,21]. Therefore, HCl·Pro-OMe was treated with an excess of NEt_3_ to give the free base which was coupled *in situ* with Y-Fca-OH (Y = Ac, Boc), previously activated by using the standard HOBt/EDC protocol.

**Scheme 1:**
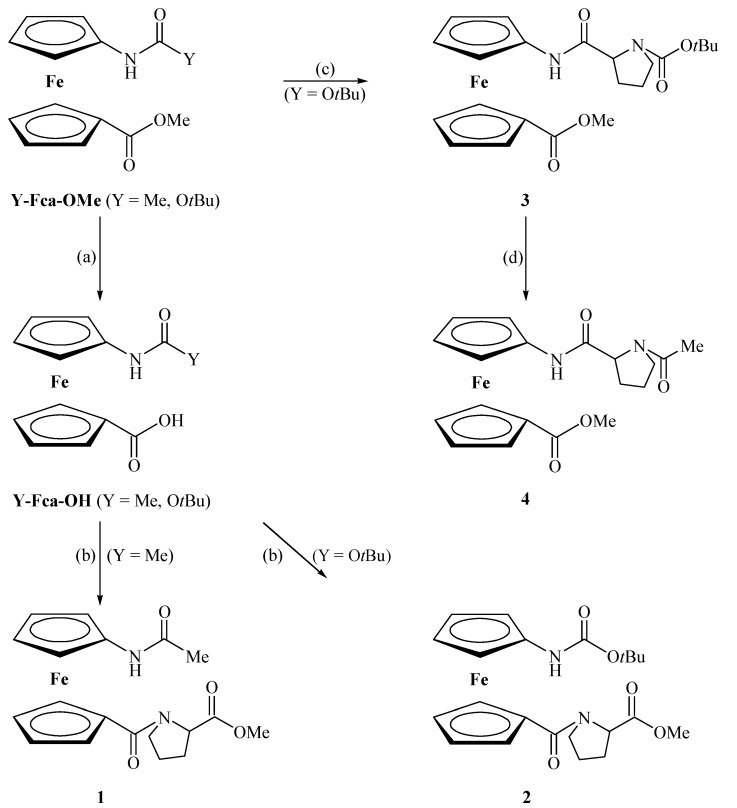
Synthesis of bioconjugates **1**–**4**.

Boc-Pro-Fca-OMe (**3**) and Ac-Pro-Fca-OMe (**4**), with an exchanged sequences of the constituent amino acids regarding to **1** and **2**, were obtained starting from Boc-Fca-OMe [25], applying the procedure used for the preparation of the recently described alanine analogues **IV** [22]. Upon Boc-deprotection in the presence of gaseous HCl, the resulting HCl·Fca-OMe was processed with an excess of NEt_3_ to liberate the *N*-terminus, followed with coupling with activated Boc-Pro-OH. The obtained Boc-Pro-Fca-OMe (**3**) was: (*i*) Boc-deprotected and (*ii*) Ac-protected upon treatment with acetyl chloride to give Ac-Pro-Fca-OMe (**4**) (Scheme 1).

*Reagents and conditions*: (**a**) NaOH/H_2_O, MeOH; (**b**) 1. EDC; HOBt, 2. HCl·Pro-OMe/Et_3_N; (**c**) 1. HCl_gas_, 2. Et_3_N, 3. Boc-Pro-OH/EDC, HOBt; (**d**) 1. HCl_gas_, 2. Et_3_N, 3. AcCl.

The structural properties of the synthesized compounds were examined in solution (for **1**–**4**) and in the solid state (**3**). The solution-phase analysis was based on the well-established strategy [16,17,18,19,20,21,22], *i*.*e*., the comparison of the spectroscopic values of a reference, a model and the new compounds. The monosubstituted analogues **X**–**XII** [17], unable to participate in IHBs, were selected as reference compounds. Since the required feature of the model substances is their structural resemblance to the tested compounds including the ability to form IHBs, proline-containing peptides **VII**–**IX** [31,32,33,34] and alanine-containing peptides **III** and **IV** [17,22] were employed as model compounds.

### 2.2. Conformational Analysis of 1–4 in Solution

Proline-containing bioorganometallics **1**–**4** were examined in solution by standard spectroscopic techniques (IR, NMR and CD spectroscopy) to assess their hydrogen-bonding potential which is of crucial importance for formation of ordered structures. Therefore, the impact of the ferrocene unit on the conformational properties of Ac-Fca-Pro-OMe (**1**), Boc-Fca-Pro-OMe (**2**), Boc-Pro-Fca-OMe (**3**) and Ac-Pro-Fca-OMe (**4**) was explored by comparison of their spectral data with those corresponding to the model peptides Boc-Phe-Pro-OMe (**VII**) Boc-Pro-Gly-OMe (**VIII**) and Ac-Pro-Gly-OMe (**IX**) reported by Ikawa and co-workers [31,32,33,34] (to the best of our knowledge, there is no literature data on Ac-AA-Pro-OMe, (AA = amino acid), an analogue of Ac-Fca-Pro-OMe (**1**)). The required feature of the model compounds is their structural semblance to the analyzed compounds, including their hydrogen-bonding potential. Model peptides **VIII** and **IX** were found to adopt 7-membered intramolecular NH_Gly_···OC_NHY_ hydrogen-bonded ring (Y = Boc, Ac), while the NH group of phenylalanine-containing peptide **VII** was free of hydrogen-bonding interactions.

Furthermore, we explored the influence of proline incorporated at *N*- or *C*-terminus of Fca, respectively, on hydrogen-bonding capability of the resulted peptides **1**–**4** by comparison with previously described alanine-containing model peptides Ac-Fca-Ala-OMe (**IIIa**), Boc-Fca-Ala-OMe (**IIIb**) [17], Boc-Ala-Fca-OMe (**IVa**) and Ac-Ala-Fca-OMe (**IVb**) [22]. The most stable conformations of **IIIa** and **IIIb** were established through interchain IHBs: NH_Fca_···OC_COOMe_ (type **A**, 9-membered) or NH_Ala_···OC_NHY_ (type **B**, 8-membered ring) (Y = Boc, Ac). Their constitutional isomers **IVa ** and **IVb** were stabilized through intrachain IHBs: NH_Fca_···OC_NHY_ (type **A**, 7-membered ring) or NH_Fca_···N_NHY_ (type **B**, 5-membered ring). An additional conformation **C** was embedded through interchain NH_NHY_···OC_COOMe_ IHB (9-membered ring) (Table 1). The IR spectroscopic data of model compounds **III**, **IV**, **VII**–**IX** is listed in Table 2.

**Table 2:** IR spectroscopic data (cm^−1^) of model compounds **III** ^[a]^, **IV** ^[a]^, **VII**-**IX** ^[b]^.

Compound	Formula	ν_NH_ (Free)	ν_NH_ (Assoc.)	ν_CO_ (Ester)	ν_CO_ (Amide I)	ν_CO_ (Amide II)
**IIIa**	Ac-Fca-Ala-OMe	3434 m	3351 w	1739 s	1682 m	1514 m
					1657 m	
**IIIb**	Boc-Fca-Ala-OMe	3433 m	3327 m	1731 s	1714 s	1518 m
					1655 m	
**IVa**	Boc-Ala-Fca-OMe	3423 m	3324 w	1709 brs	1557 m	1504 m
					1536 m	
**IVb**	Ac-Ala-Fca-OMe	3425 m	3290 w	1709 s	1664 m	1507 m
					1562 m	
					1538 m	
**VII**	Boc-Phe-Pro-OMe	~3450 m		^[c]^	^[c]^	^[c]^
**VIII**	Boc-Pro-Gly-OMe	~3430 m	~3310 w	^[c]^	^[c]^	^[c]^
**IX**	Ac-Pro-Gly-OMe	~3430 m	~3300 m	^[c]^	^[c]^	^[c]^

^[a]^ IR spectra were recorded in CH_2_Cl_2_ (*c* = 5 × 10^−2^ M); ^[b]^ IR spectra were recorded in CDCl_3_ (*c* = 1.25 × 10^−3^ M); ^[c]^ No data available.

#### 2.2.1. IR Spectroscopy

**Figure 1:**
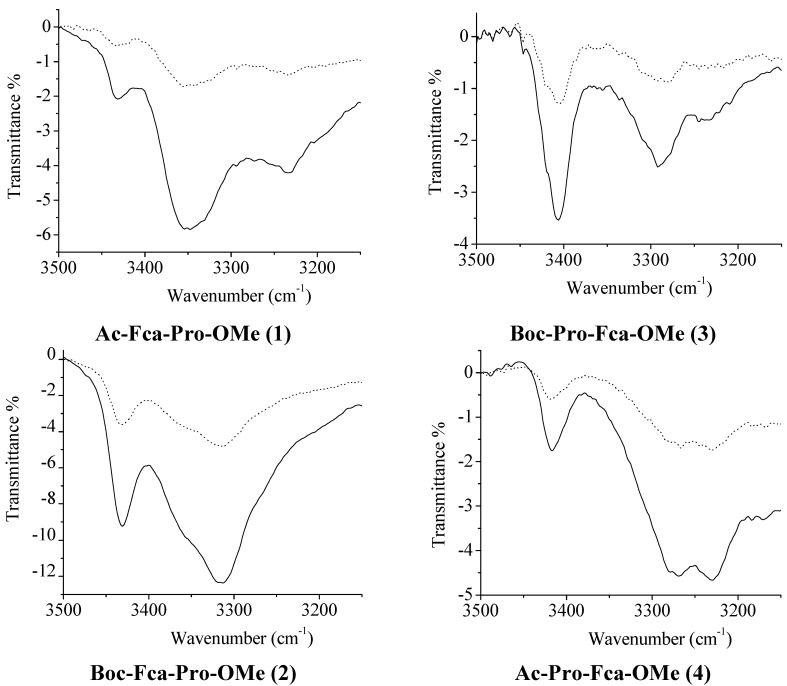
.The NH stretching vibrations in concentration-dependent IR spectra of **1**–**4** in CH_2_Cl_2_ (solid line: *c* = 5× 10^−2^ M, dashed line: *c* = 5× 10^−4^ M).

The amide NH and carbonyl stretching regions of the IR spectra of the peptides **1**–**4** indicate the presence of both associated and free states. Generally, the absorption bands below 3400 cm^−1^ are assigned to the hydrogen-bonded NH groups, while the higher absorptions are assigned to non-bonded NH groups. The intensification of the bands below 3400 cm^−1^ in IR spectra of peptides **1**, **2** and **4** suggests that the amount of their hydrogen*-*bonded states increases in population in comparison with peptide **3**. Moreover, two hydrogen-bonded internal rotamers of **1**, **3** and **4** are seen as two separate signals below 3300 cm^−1^ (Figure 1, Table 3).

**Table 3:** IR spectroscopic data ^[a]^ (cm^−1^) of reference (**X**–**XII**) and goal compounds **1**–**4**.

Compound	Formula	ν_NH_ (Free)	ν_NH_ (Assoc.)	ν_CO_ (Ester)	ν_CO_ (Amide I)	ν_CO_ (Amide II)
**X**	Fc-COOMe			1711		
**XI**	Fc-NHAc	3436 m			1684	
**XII**	Fc-NHBoc	3436 m			1723	
**1**	Ac-Fca-Pro-OMe	3434 w	3351 m 3231 m	1741	1676 1604	1535
**2**	Boc-Fca-Pro-OMe	3430 m	3356 sh 3315 m	1741 sh	1718 1604	1532
**3**	Boc-Pro-Fca-OMe	3419 sh 3405 m	3290 m 3234 w	1706	1694 1655	1532
**4**	Ac-Pro-Fca-OMe	3416 w	3272 m 3229 m	1709	1690 1623	1563

^[a]^ IR spectra were recorded in CH_2_Cl_2_ (*c* = 5 × 10^−2^ M). Fc = ferrocenyl, Fca = 1'-amino-ferrocene-1-carboxylic acid.

In order to determine whether the associated NH groups are engaged in an intra- or intermolecular manner, the concentration dependence of the solution NH absorption bands was tested. Since the ratio of the associated and free NH bands of **1**–**4** revealed unaffected upon successive dilution from *c* = 5 × 10^−2^ M to 5 × 10^−4^ M, their intramolecular employment is strongly indicated (Figure 1). Furthermore, the ratio of hydrogen-bonded and non-bonded NH peak intensities depends on the employed protecting group. The bulky Boc groups of **2** and **3** interfere with hydrogen bonding and reduce the content of their associated NH bands.

The carbonyl stretching frequencies indicate if the corresponding ester or amide groups are involved in hydrogen bonding. In order to assign the peaks observed in the carbonyl region, we compared the spectroscopic data of a reference and the new compounds. The corresponding monosubstituted analogues Fc-COOMe (**X**), Fc-NHAc (**XI**) and Fc-NHBoc (**XII**) (Fc = ferrocenyl) [17] served as a reference compounds due to their inability to form intramolecular hydrogen bonds (IHBs) (Table 3).

Since the carbonyl stretches of esters **1** and **2** at 1741 cm^−1^ correspond to the bands of associated model esters **III**, their engagement in hydrogen bonding is indicated. Contrarily, the carbonyl stretching absorptions of esters **3** and **4** that are correlated to those of non-bonded esters **IV** and **X**, indicate their non-involvement in hydrogen-bonding. Considering that hydrogen-bond-accepting urethane [30,35,36] and acetamide group [19] absorb at lower wavenumbers, the urethane carbonyl group of Boc-Pro-Fca-OMe (**3**) and acetamide carbonyl group of Ac-Pro-Fca-OMe (**4**), registered around 1690 cm^−1^, are expected to participate in hydrogen bonding. Boc and Ac carbonyl groups, attached at upper cyclopentadienyl ring (Cp) of Ac-Fca-Pro-OMe (**1**) and Boc-Fca-Pro-OMe (**2**), are precluded from participating in IHBs because of the unavailability of hydrogen-bonding-donating groups at the lower Cp ring. Therefore, their absorption frequencies are correlated with those belonging to the hydrogen-bonding free **XI** and **XII** (Table 3).

#### 2.2.2. NMR Spectroscopy

The hydrogen bonding engagement of the bioconjugates **1**–**4**, strongly indicated by the corresponding NH stretching absorptions below 3400 cm^−1^, was corroborated by the downfield shifts (δ > 7 ppm) of their NH groups in non-polar CDCl_3_. Certainly, the hydrogen-bonded NH groups of the previously described model compounds **III**, **IV** and **VII**–**IX** were found to resonate downfield in similar positions, while NH groups of the HB-free reference compounds **XI** and **XII** were upfield shifted (Table 4).

**Table 4:** ^1^H-NMR chemical shifts ^[a]^ of the NH protons (ppm) of a model (**III**, **IV**, **VII-IX**), a reference (**XI**, **XII**) and the goal compounds **1**–**4**. The proposed IHB patterns of a model and a reference compounds **VII-XVI**. The possible IHB patterns of the goal compounds **1**–**4**.

Compound	Formula	δ/ppm (NH_AA_) ^[b]^	δ/ppm (NH_Fca/Fc_)	IHB Pattern	IHB Ring Size
**IIIa**	Ac-Fca-Ala-OMe	6.71	7.93	NH_Fca_···OC_Ala_	9-membered
				NH_Ala_···OC_Ac_	8-membered
**IIIb**	Boc-Fca-Ala-OMe	6.77	6.40	NH_Fca_···OC_Ala_	9-membered
				NH_Ala_···OC_Boc_	8-membered
**IVa**	Boc-Ala-Fca-OMe	5.14	7.64	NH_Fca_···N_Ala_	5-membered
				NH_Ala_···OC_Fca_	9-membered
**IVb**	Ac-Ala-Fca-OMe	6.41	8.04	NH_Fca_···OC_Ac_	7-membered
				NH_Fca_···N_Ala_	5-membered
				NH_Ala_···OC_Fca_	9-membered
**VII**	Boc-Phe-Pro-OMe	^[c]^		^[d]^	
**VIII**	Ac-Pro-Gly-OMe	7.25(t)		NH_Gly_···OC_Ac_	7-membered
		6.23(c)			
**IX**	Boc-Pro-Gly-OMe	7.28(t)		NH_Gly_···OC_Boc_	7-membered
		6.49(c)			
**XI**	Fc-NHAc		6.49	^[d]^	
**XII**	Fc-NHBoc		5.55	^[d]^	
**1**	Ac-Fca-Pro-OMe ^[e]^		8.23(t)	NH_Fca_···OC_Pro_	9-membered
			8.38(c)		
**2**	Boc-Fca-Pro-OMe ^[e]^		7.12(t)	NH_Fca_···OC_Pro_	9-membered
			6.80(c)		
**3**	Boc-Pro-Fca-OMe ^[e]^		8.78(t)	NH_Fca_···OC_Pro_	7-membered
			7.53(c)		
**4**	Ac-Pro-Fca-OMe ^[e]^		8.81(t)	NH_Fca_···OC_Pro_	7-membered
			7.29(c)		

^[a]^ NMR spectra were measured in CDCl_3_ (*c* = 5 × 10^−2^ M); ^[b]^ AA = natural amino acid; ^[c]^ No data available; ^[d]^ IHB was not indicated; ^[e]^ The occurrence of both isomers [(c) = *cis*, (t) = *trans*] in solution at 298 K is confirmed by ^13^C-NMR on the basis of the well-documented shift differences observed for C_β_- and C_γ_-atoms [37,38].

**Figure 2:**
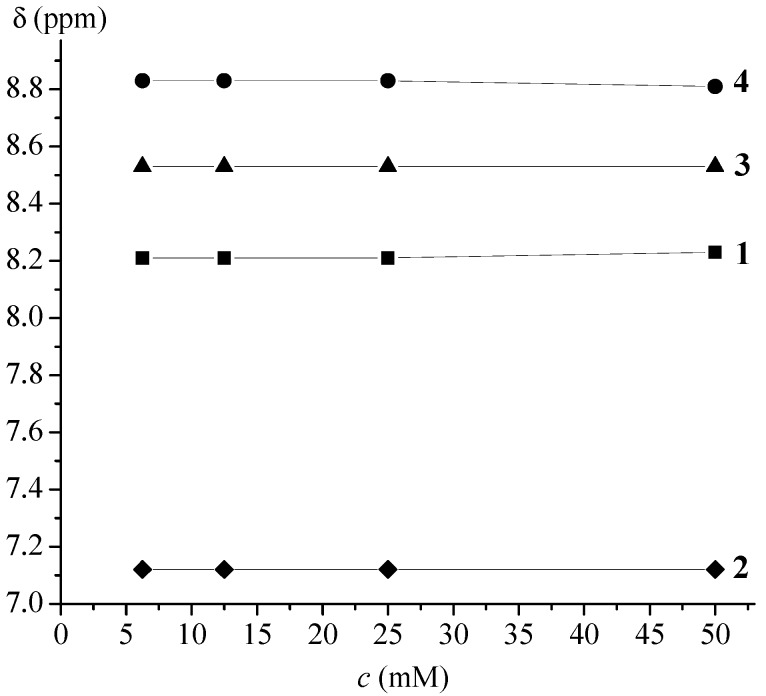
Concentration dependent NH*_trans_* chemical shifts of peptides **1**–**4**. [^1^H-NMR measurements were performed for a series of 6.25, 12.5, 25 and 50 mM solutions].

**Figure 3:**
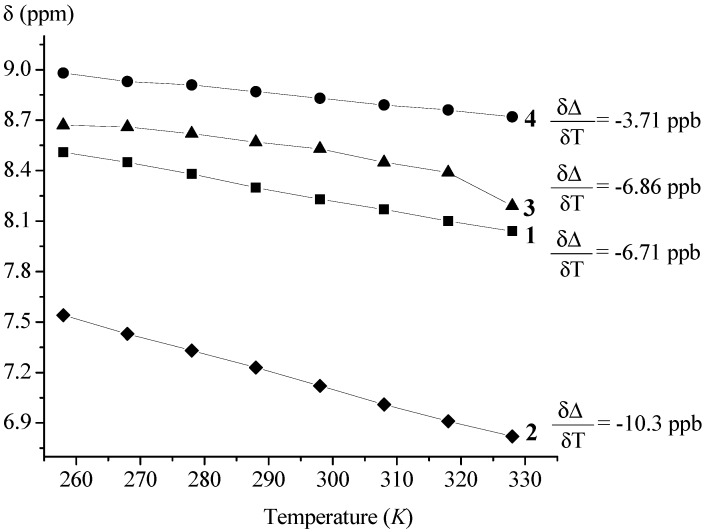
Temperature dependent NH*_trans_* chemical shifts of peptides **1**–**4** (*c* = 2.5× 10^−2^ M) in the temperature range of 258–328 K.

The goal of this paper was to determine the conformational consequences of: (*i*) the replacement of natural amino acid (phenylalanine or glycine) belonging to the model proline-containing peptides **VII**–**IX** with bioorganometallic amino acid (Fca) and (*ii*) the replacement of alanine units of Fca-containing model peptides **III** and **IV** with proline. Due to the lack of an amide hydrogen, proline residues of **1**–**4** are not able to donate hydrogen bonding. Their possible involvement in hydrogen bonding might be realized through inter- (9-membered) or intrachain (7-membered) IHB rings upon accepting NH_Fca_ (Table 4). Considering that the model peptides **III**, **IV** and **VII**–**IX** were stabilized through IHBs, the expected IHB patterns of the novel peptides **1**–**4** were explored by concentration- and temperature-dependent NMR analyses. The considerable changes in chemical shifts (Δδ = 1–4 ppm) upon dilution or heating are expected for intermolecular hydrogen bonding. Since the successive decreasing of concentration (50–6.25 mM) did not cause any discernible changes in chemical shifts of amide protons, the intramolecular hydrogen bonds, proposed by IR experiments, were corroborated (Figure 2). Furthermore, the results of temperature-dependent NMR spectroscopy were in accordance with these findings. The examined conjugates **1**–**4** demonstrated negligible dependence of the chemical shifts (Δδ < 0.7 ppm) on temperature (Figure 3).

The measurements of the temperature dependences of chemical shifts (Δδ/ΔT) in hydrogen bonding solvents enable differentiation of the solvent-exposed and solvent-shielded peptide NH groups [39,40,41,42,43,44,45]. Since DMSO causes rapid decomposition of ferrocene peptides, their temperature dependences were determined in CDCl_3_ solution [22,46,47]. Generally, the law Δδ/ΔT values (−2.4 ± 0.5 ppb/K) are measured for both exposed and shielded amide protons of short peptides. The larger temperature dependencies are characteristic for NH groups which were shielded from solvent at starting state, but became exposed through dissociation of the self-associated aggregates or unfolding of ordered conformations at increased temperatures [48]. Accordingly, larger temperature dependencies of concentration-independent amide protons of **1**–**4** emanate from the initially shielded species switched to the unshielded environment by unfolding of conformations organized through IHBs (Figure 3).

**Figure 4:**
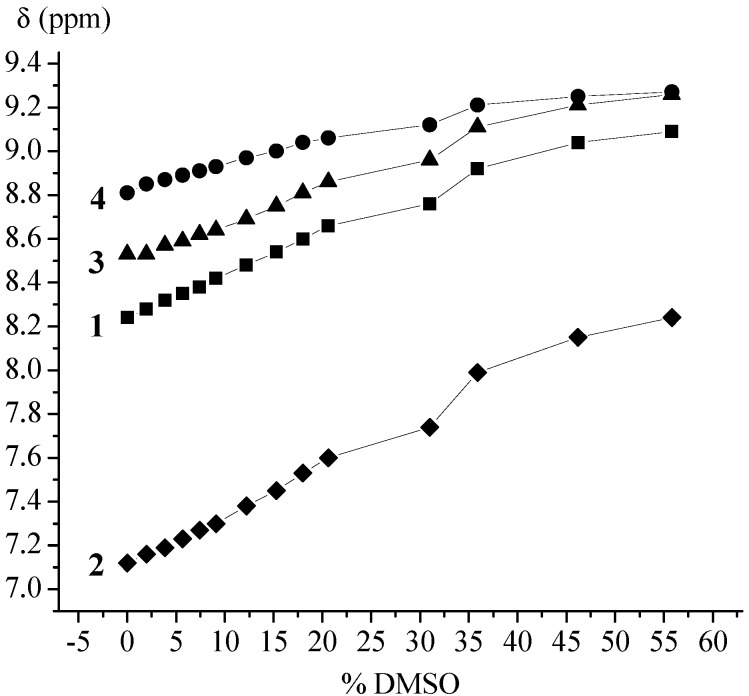
Solvent dependence of NH*_trans_* chemical shifts at varying concentrations of DMSO in CDCl_3_ (*c* = 2.5× 10^−2^ M, 298 K) to probe exposed *vs.* hydrogen-bonded amides.

Polarity and hydrogen bonding potency of a solvent are of outstanding significance in the evaluation of hydrogen bond strength. Owing to its accepting capability, DMSO disrupts peptide hydrogen bonds causing the unfolding of ordered structures [49]. Taking that in consideration, the strength of IHBs indicated by the results of IR and NMR analyses was explored by DMSO titration experiments (Figure 4) [16,17,18,19,20,21,22,50]. The highest chemical shift value of NH_Fca_ belonging to Ac-Pro-Fca-OMe (**4**) and the lowest chemical shift alteration upon addition of DMSO (Δδ = 0.42 ppm) indicate its engagement in a strong IHB. A similar behaviour in DMSO experienced amide protons of Ac-Fca-Pro-OMe [**1**, (Δδ = 0.85 ppm)] and Boc-Pro-Fca-OMe [**3**, (Δδ = 0.73 ppm)] suggesting their participation in IHBs of medium strength. Contrarily, upfield shifting (δ = 7.12 ppm) and the highest chemical shift variation (Δδ = 1.12 ppm) of NH_Fca_ belonging to Boc-Fca-Pro-OMe (**2**) indicate its involvement in weak IHB.

**Figure 5:**
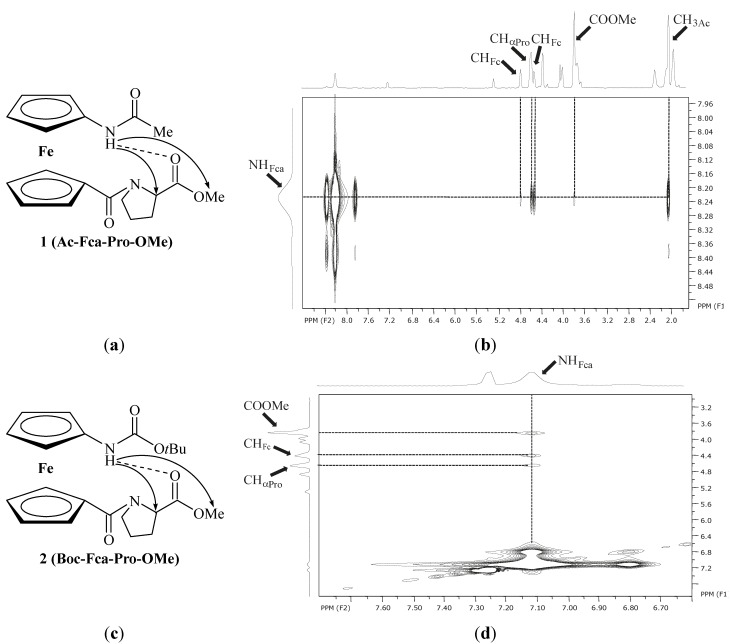
The proposed IHBs in peptides **1** (**a**) and **2** (**c**) are depicted with dashed lines and the interchain NOE connectivities are presented with arrows; the interchain NOE connectivities between the *C*- and *N*-terminiof **1** (**b**) and **2** (**d**) are depicted with dashed lines.

The IHB patterns of peptides **1**–**4**, proposed by IR and ^1^H-NMR analyses (Table 4), are supported by their NOESY spectra. The interchain NOE contacts between amide protons of Fca and proline belonging CH_α_ and COOMe protons strongly support the proposed 9-membered NH_Fca_···OC_Pro_ IHB rings in peptides **1** and **2** (Table 4, Figure 5). 

**Figure 6:**
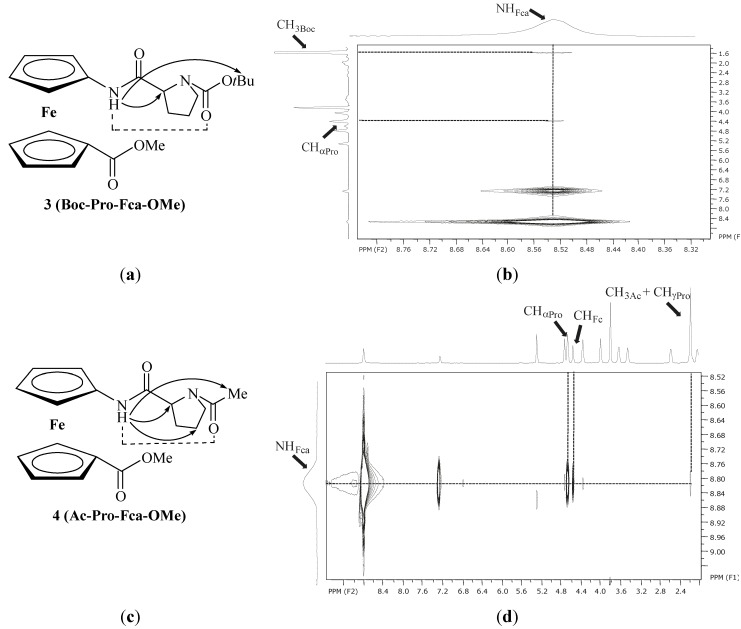
The proposed IHBs in peptides **3** (**a**) and **4** (**c**) are depicted with dashed lines and the intrachain NOE connectivities are presented with arrows; the intrachain NOE connectivities of **3** (**b**) and **4** (**d**) are depicted with dashed lines.

Furthermore, NOE contacts of amide protons of Fca within the peptide chain attached at upper Cp of **3** and **4**, promote the suggested intrachain NH_Fca_···OC_Pro_ IHBs (γ-turn) (Table 4, Figure 6). NMR investigations of the solvent influence on the *cis-trans* isomerization of small peptides [51,52,53,54,55,56,57,58,59] revealed the increased fraction of the *trans* form in less polar or non-polar solvents. It was also found that intramolecular hydrogen bonds are consistent with the *trans* form [60]. Multiple resonances detected for the same proton during the course of NMR analysis are attributed to the presence of rotamers if the rate of the interconversion (e.g., amide *cis-trans* isomerization) is slow relative to the NMR scale [37,40,61]. On heating, isomerization rate increases causing the coalescence of signals. The fully coalesced peaks at higher temperatures correspond to the weighted average chemical shift value for the two conformers on the NMR timescale. Contrary, slow proline isomerization at higher temperatures reveals the peaks resolved due to the persistence of *cis* and *trans* conformers at NMR timescale [62,63,64,65].

The NMR spectra recorded at: (*i*) decreased temperatures and (*ii*) an increased amount of DMSO, respectively, identified the *cis* and *trans* isomers of the new peptides **1**–**4**. The *trans:cis* ratio was calculated from integrated intensities of the appropriate resonances. Upon heating to 328 K, the two peaks of the *trans* and *cis* isomers of **1**–**4** coalesced into a single peak due to the medium (compound **3**) and rapid (compounds **1**, **2** and **4**) isomerization. In DMSO, peptides **3** and **4** underwent to slow or medium isomerization, respectively, while peptide **1** displayed rapid isomerization. Completely coalesced amide resonances of peptide **2** upon titration with DMSO reflect the average chemical shift of rapidly exchanged *cis* and *trans* rotamers as a consequence of weak IHB ineligible to induce isomer locking. In contrast, the abundance of the *trans* amide in peptide **4** is attributed to its stabilization through stronger IHB in comparison to those proposed for the bioconjugates **1**–**3** (Table 5). These finding are consistent with the above-mentioned conclusions [51,52,53,54,55,56,57,58,59,60,61,62,63,64,65] concerning the *cis-trans* isomerization of a proline imide bond of small peptides.

**Table 5:** The *trans-cis* ratios of imide bonds of peptides **1**–**4** measured by ^1^H-NMR.

**Ac-Fca-Pro-OMe (1)**		**T (K) ^[a]^**		**DMSO (%) ^[b]^**		**Boc-Fca-Pro-OMe (2)**		**T (K) ^[a]^**		**DMSO (%) ^[b]^**	
		228	328	0	36			228	328	0	36
δ (**ppm**)	*cis*	8.67	8.04	8.39	9.00	δ (**ppm**)	*cis*	7.14	6.83	6.80	7.99
	*trans*	8.51		8.23	8.92		*trans*	7.57		7.13	
	*trans:cis*	90:10		91:9	90:10		*trans:cis*	90:10		93:7	
**Boc-Pro-Fca-OMe (3)**		**T (K)**		**DMSO (%) ^[a]^**		**Ac-Pro-Fca-OMe (4)**		**T (K)**		**DMSO (%) ^[a]^**	
		228	328	0	36			228	328	0	36
δ (**ppm**)	*cis*	7.38	8.21	8.53	9.00	δ (**ppm**)	*cis*	7.63	8.72	7.29	9.41
	*trans*	8.67			9.10		*trans*	8.98		8.81	9.21
	*trans:cis*	66:33			56:44		*trans:cis*	94:6		93:7	74:26

^[a]^ The temperature-dependent NMR spectra were measured in CDCl_3_ (*c* = 2.5 × 10^−2^ M); ^[b]^ ^1^H NMR titration of CDCl_3_ solutions of **1**–**4** (*c* = 2.5 × 10^−2^ M) with DMSO was performed at 298 K.

#### 2.2.3. CD Spectroscopy

Circular dichroism is a valuable tool for evaluating the secondary structure, folding and binding properties of proteins [66]. The insertion of ferrocene into the chiral peptide environment induces Cotton effect in the region of ferrocene-based transitions around 480 nm owing to an ordered structures driven by hydrogen bonding between podand peptide chains [6,7,8,9,10,11,12,13,16,17,18,19,20,21,22] or within the same peptide chain [12,67]. Furthermore, it was shown that the sign of the CD signal of ferrocene peptides (which reflects an average of the entire molecular population) is influenced by the sequence of the natural peptide fragment [17,19,68], the type of *N*-protected groups attached at ferrocene core [19] and the type of the employed solvent [17,19].

**Figure 7:**
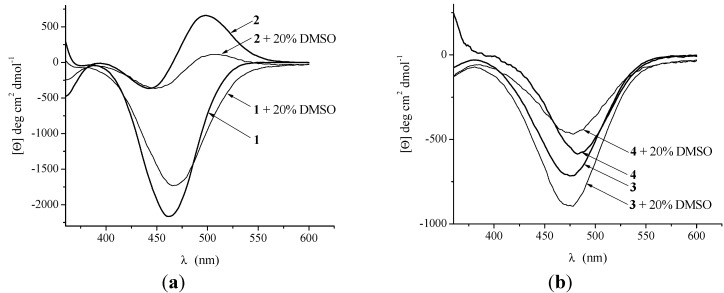
The Cotton effects in chirality-organized ferrocene peptides **1** and **2** (**a**) and **3** and **4** (**b**) in CH_2_Cl_2_ (*c* = 5 × 10^−3^ M) and CH_2_Cl_2_ (*c* = 5 × 10^−3^ M) containing 20% of DMSO.

Although the proposed hydrogen bonding patterns for peptides **1** and **2** are very similar, their CD spectra are quite different due to chromophoric reorientation induced by hydrogen bonding in the presence of different protecting groups [69]. The different intensities of the Cotton effects in the CD spectra of the bioconjugates **1**–**4** indicate the different levels of chiral organization. The absolute values of Cotton effects of **2**–**4**, ranged between 500 and 1,000 deg·cm^2^·dmol^−1^, are in a good agreement with previously described alanine analogues **III** [17] and **IV** [22]. The increased CD-activity of peptide **1** indicates higher level of chiral organization established through interchain NH_Fca_···OC_COOMe_ IHB (Figure 7). Moreover, the CD-silent ferrocene region in the spectrum of peptide **2**, obtained upon titration with DMSO, is attributed to the loss of the chiral organization owing to the cleavage of weak IHB. The decreasing of the CD intensity in a strong hydrogen-bond forming solvent, demonstrated herein for peptides **1** and **4**, has also been shown for previously described ferrocene peptides [16,19,20]. However, the addition of DMSO to CH_2_Cl_2_ solution of **3** caused the increment of the CD signal. This exception, reported for the several ferrocene ureidopeptides by Heinze *et al*. arose from substantial alteration of the chromophore setting of the examined peptide in CH_2_Cl_2_ and CH_2_Cl_2_/DMSO. Heinze reached the conclusion that the solvent dependent Cotton effects observed are greatly influenced by other ordering phenomena, while the stable conformations established through IHBs in non-polar solvents have only a minor influence [47].

#### 2.2.4. Crystal Structure of Boc-Pro-Fca-OMe (**3**)

**Figure 8:**
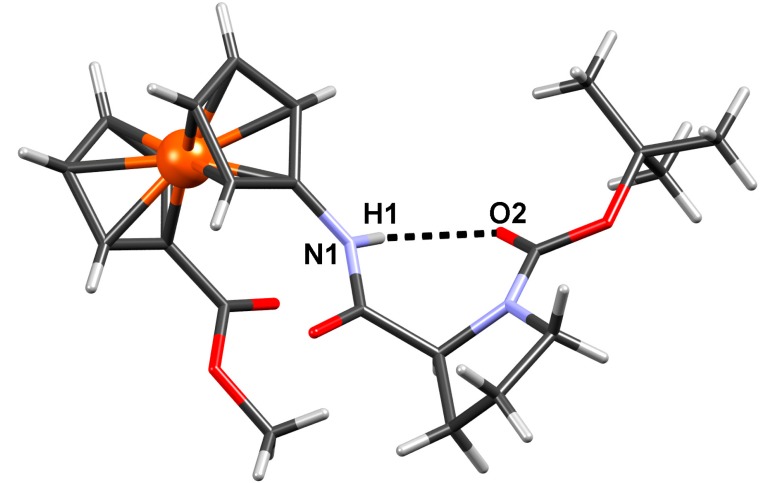
Conformation of Boc-Pro-Fca-OMe (**3**) in the crystal stabilised by *intrachain* intramolecular hydrogen bonding.

**Figure 9:**
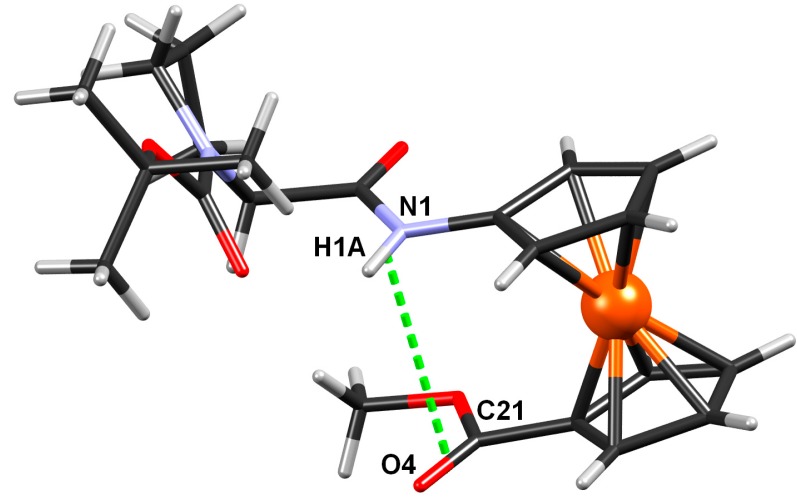
An additional stabilization of Boc-Pro-Fca-OMe (**3**) through *interchain* intramolecular dipolar interaction between CO and NH groups.

Single crystal X-ray diffraction confirmed existence of an intrachain hydrogen bond in Boc-Pro-Fca-OMe (**3**) which was established by NMR spectroscopy. Conformation of the compoundis stabilised by one N-H···O and four weak C-H···O intramolecular hydrogen bonds (Figure 8, Table 6). An additional stabilization is achieved through interchain dipolar interaction between a carbonyl group bound to one Cp ring and NH group bound to another Cp ring (Figure 9): the covalent bonds are approximately parallel (torsion angle between them being 5.9°), and distance between their midpoints is 3.33 Å. However, there are no intermolecular hydrogen bonds present, so the packing is dominated by dispersion interactions, only.

**Table 6:** Geometric parameters of hydrogen bonding.

*D*–H···A	*D*–H/Å	H··· *A*/Å	*D*···*A*/Å	*D*–H···*A*/°	Symm. op. on *A*
N1–H1···O2	0.86	2.10	2.810(10)	139	*x*, *y*, *z*
C2–H2···O1	0.93	2.46	2.906(12)	110	*x*, *y*, *z*
C8–H8B···O1	0.97	2.47	2.827(14)	101	*x*, *y*, *z*
C13–H13C···O2	0.96	2.37	2.969(14)	120	*x*, *y*, *z*
C15–H15C···O2	0.96	2.45	3.031(16)	119	*x*, *y*, *z*

### 2.3. Biological Evaluation of Bioconjugates **1**–**4**

The use of peptidomimetics is one of the most recent methods of drug design and development in medicinal chemistry due to various types of pharmacological activities displayed by them. Due to their versatile activities (antimicrobial, anticancer, antiviral, antimalarial, antioxidant, immunosuppressive activity, immunodetection, selectivity for DNA receptors *etc*. [70]), peptidomimetics are utilized to design and develop novel drug molecules which can be effective in the treatment of various human diseases. Since cancer is considered as the most serious health problem all over the world, the discovery of new potent, safe and selective antitumor agents is strongly needed. Peptidomimetics have potential as antitumor agents due to their anti-cancer activity reported in the literature. For example, anticancer peptidomimetics can bind to target proteins and induce cancer cells to enter apoptotic cell death by mimicking key interactions that activate apoptotic pathway in the specified cells [71]. Also, Liao *et al*. synthesized novel unnatural amino acid-substituted (hydroxyethyl)urea peptidomimetics which inhibited secretase and interfered with the malignancy of neuroblastomas and therefore showed that these peptidomimetics can be used as lead compounds for further development of novel anticancer drugs [72].

U.S. National Cancer Institute (NCI) phased out its murine leukemia P388 anticancer drug screening program in 1990 and developed *in vitro* primary screen based upon a panel of 60 different human tumor cell lines [73]. Only in first five years of that program 30,000 compounds involved in cancer researches worldwide have been tested according to NCI screen. Compounds interesting as potential anticancer drug are being tested over a defined range of concentrations to determine the relative degree of growth inhibition or cytotoxicity against each cell line.

In the ongoing research, evaluation of biological activity of novel Fca and proline conjugates **1**–**4** was done against human tumour cell lines. Since conventional anticancer drug discovery and development is focused on the search for cytotoxic agents by this preliminary*in vitro* screen we aimed to determine potential of compounds **1**–**4** as cytotoxic *i*.*e*., antitumor agents.

**Figure 10:**
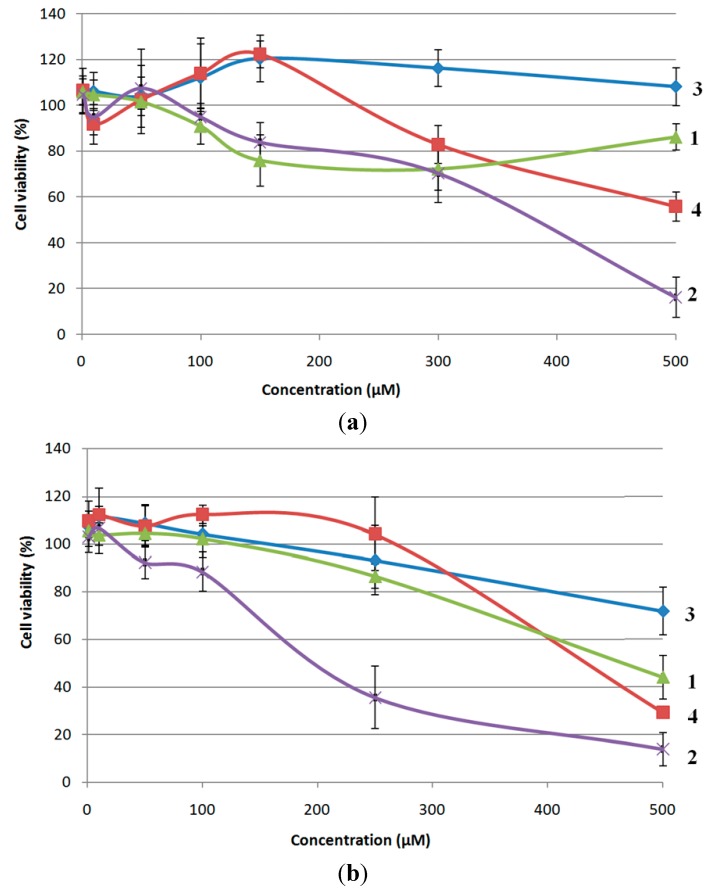
Effects of four different compounds on MCF-7 (**a**) and HeLa (**b**) cells viability assessed by the WST-1 assay. Values were represented as the mean ± SD from three independent experiments.

The biological activity of peptides **1**–**4**, with emphasis to possible anti-cancer activity, was tested *in vitro* and evaluated on the basis of their ability to inhibit growth of MCF-7 mammary carcinoma cells and HeLa cervical carcinoma cells. Cytotoxicity of the synthesized compounds was assessed on the basis of *in vitro* growth in 96-well plates measured by cell-mediated reduction of tetrazolium salt WST-1 and the results are expressed as cell viability (%) and presented in Figure 10.

Compound **3** does not possess antiproliferative potential toward MCF-7 cell line, while other tested compounds showed some potential to inhibit growth of MCF-7 cells (Figure 10a). The 50% inhibition of cell growth was not detected by the compounds **1**, **3** and **4** in the range of tested concentrations (1–500 µM) and therefore their IC_50_ values are considered to be higher than 500 µM. The highest cytotoxicity in MCF-7 cells is obtained by compound **2** with IC_50_ value of 357.72 µM. Similar results were obtained with HeLa cells (Figure 10b). The trend of cytotoxicity is the same as in MCF-7 cells; the weakest effect was provoked by compound **3** and the most pronounced inhibition of HeLa cells growth was observed in the treatment with a compound **2**. The compounds **1**, **2** and **4** have resulted with 50% inhibition of cell growth after the 72-hour treatment and therefore IC_50_ values for HeLa cells were calculated to be: 474.99 µM (compound **1**), 463.64 µM (compound **4**) and 203.27 µM (compound **2**). Considering that the peptide **2** is stabilized by the weakest IHB in comparison with conjugates **1**, **3** and **4**, these results are quite unexpected. But, having in mind that polarity or lipophilicity of the compound is a prerequisite for potential pharmaceutical applications, the obtained results might be attributed to the increased lipophilicity of peptide **2** (R_f_ = 0.50) with regard to more polar analogues **1** (R_f_ = 0.30), **3** (R_f_ = 0.33) and **4** (R_f_ = 0.22). Obtained IC_50_ values are relatively high when compared with compounds with well known anti-cancer activity, like doxorubicine or cisplatin, as the reference drugs. IC_50_ values for doxorubicin and cisplatin determined in different human cancer cell lines and obtained from literature survey varies mostly from 0.1 µM to 15 µM [74,75] depending on cell type. Since previously described conjugates of Fca with amino acids had not been subjected to biological evaluation, we were not able to explore how the introduction of proline into conjugates **1**–**4** influences the cytotoxicity of this class of peptidomimetics. The obtained results on cytotoxic activity of herein described bioorganometallics **1**–**4** represents the initial step in the future investigation of ferrocene-containing peptidomimetics.

Therefore, peptide **2**, which exibited the highest inhibitory effect among tested bioconjugates actually has poor cytotoxic activity toward MCF-7 and HeLa cells and can not be considered as potential anticancer drug candidate. Nevertheless, the obtained results are valuable as a guide how to further direct the synthesis of similar analogues with improved biological properties.

**Figure 11:**
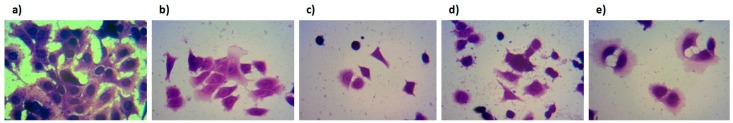
Light microscopy of MCF-7 cells stained with cystal-violet after 72 h of treatment with tested compounds **1**–**4**. Control cells (**a**) and cells treated with 500 µM **1** (**b**); **2** (**c**); **3** (**d**) and **4** (**e**). Magnification 400×.

The cells were daily examined by inverted light microscopy to observe if there are any visible morphological changes in MCF-7 cells. After treatment with synthesized compounds, cells were stained with crystal-violet and photographed (Figure 11).

Exposure to the highest concentration of the tested compounds **1**–**4** (500 µM) for 72 h resulted in reduction of cell number and decreased monolayer density (Figure 11b–e) when compared to control MCF-7 cells (Figure 11a) which were well attached and has typical epithelial morphology. Evidently, the proliferation of the MCF-7 cancer cell was inhibited by the testing compounds *in vitro* and observed morphological changes correspond with obtained cytotoxicity results, but further investigation is needed to explore possible mechanism of action and type of cell death.

## 3. Experimental Section

### 3.1. General Information

The syntheses were carried out under argon. The CH_2_Cl_2_ used for synthesis and FTIR was dried (P_2_O_5_), distilled over CaH_2_, and stored over molecular sieves (4 Å). EDC (Acros Organics, Geel, Belgium), HOBt (Aldrich, Schnelldorf, Germany), acetyl chloride (Aldrich, Schnelldorf, Germany) and proline (Acros Organics, Geel, Belgium), were used as received. The syntheses of Y-Fca-OMe andY-Fca-OH have been previously described [16,17,18,19,20,21,25]. The protection of *N*- or *C*-termini of proline was carried out by using sodium hydroxide, aqueous dioxane and di-*tert*-butyldicarbonate to give Boc-Pro-OH or gaseous HCl in MeOH to obtain HCl·Pro-OMe. Products were purified by preparative thin layer chromatography on silica gel (Kieselgel 60 HF254, Merck, Darmstadt, Germany) by using CH_2_Cl_2_ or mixture EtOAc/CH_2_Cl_2_ as eluents. Infrared spectra were recorded as CH_2_Cl_2_ solutions between NaCl windows by using a Bomem MB 100 mid FTIR spectrometer. (m) = medium, (w) = weak, (sh) = shoulder. The ^1^H and ^13^C NMR spectra were recorded at 600 MHz on Bruker Avance spectrometer and were referenced to the residual solvent peak (CDCl_3_, ^1^H: 7.26 ppm, ^13^C: 77.16 ppm). In the case of the CDCl_3_/*d*_6_-DMSO mixture, the calibration was done using Me_4_Si as internal standard due to the overlapping of the residual solvent peak of *d*_6_-DMSO with proline peaks. Double resonance experiments (COSY, NOESY and HMBC) were performed in order to assist in signal assignment. (s) = singlet, (d) = doublet, (m) = multiplet or (br) = broad. Unless otherwise noted, all spectra were recorded at 298 K. CD spectra were recorded using a Jasco-810 spectropolarimeter in CH_2_Cl_2_. Molar ellipticity coefficients, [*θ*], are in degrees, concentration, *c*, is given in mol·L^−1^, and path length, *l*, is given in cm, to give units for [*θ*] of deg·cm^2^·dmol^−1^. NMR titrations were performed by adding of 10 µL portions of the *d_6_*-DMSO in a NMR-tubes containing solutions of the examined peptides **1**–**4** in CDCl_3_ (*c* = 2.5 × 10^−2^ M). The spectra were recorded after each addition and *d_6_*-DMSO was added until no further change in the chemical shifts of the NHs were observed. CD titrations were performed in a similar manner by stepwise addition of DMSO into cuvettes containing solutions of the examined peptides **1**–**4** in CDCl_3_ (*c* = 5 × 10^−3^ M). The mass spectra were acquired using 4800 MALDI TOF/TOF-MS Analyzer. Preparation of intact samples. Peptide samples **1**–**4**, each were dissolved in deionized water to concentration of 0.05 mg/mL. Volume of 10 µL of each sample solution was purified using ZipTip C4 pipette tips (Millipore, Bedford, MA, USA) and dried, then dissolved in CHCA matrix (α-cyano-4-hydroxycinnamic acid, 5 mg/mL, Sigma–Aldrich) and dried onto the MALDI plate (volume of 1 µL). At the end, 0.05 µg of peptide was deposited onto the MALDI plate. MALDI TOF/TOF-MS. For direct profiling and MS/MS fragmentation study of peptides a 4800 Plus MALDI TOF/TOF analyzer (Applied Biosystems Inc., Foster City, CA, USA) equipped with a 200 Hz, 355 nm Nd:YAG laser was used. Acquisition was performed in positive ion reflector mode. Instrument parameters were set using the 4000 Series Explorer software version (V 3.5.3, Applied Biosystems Inc.). Mass spectra were obtained by averaging 1000 laser shots covering the mass range between *m/z* 9 and 4500. MS/MS was achieved by 1 or 2 kV collision induced dissociation (CID) in positive ion mode. For both MALDI-TOF MS and MS/MS analysis, CHCA matrix was used. Dried purified samples (both intact and derivatized) were diluted in 5 µL of matrix, 1 µL of mixture was spotted onto the MALDI plate and allowed to dry.

### 3.2. General Procedure for the Preparation of Compounds ***1*** and ***2***

*Ac-Fca-Pro-OMe* (**1**) *and* *Boc-Fca-Pro-OMe* (**2**). Ac-Fca-OH (200 mg, 0.76 mmol) and Boc-Fca-OH (262 mg, 0.76 mmol), prepared by hydrolysis of the fully protected Fca [25], were activated using EDC (162 mg, 0.84 mmol) and HOBt (114 mg, 0.84 mmol) in CH_2_Cl_2_, respectively. Pro-OMe (obtained from HCl·Pro-OMe (254 mg, 1.53 mmol) by treatment with Et_3_N in CH_2_Cl_2_, pH ~ 8) was added and the mixtures were stirred at room temperature until complete consumption of the ferrocene starting material, as monitored by TLC. The standard work-up (washing with a saturated aqueous solution of NaHCO_3_, 10% aqueous solution of citric acid and brine, drying over Na_2_SO_4_ and evaporation in vacuo) followed with TLC-purification of the crude products [CH_2_Cl_2_:EtOAc = 5:1 (**1**) and CH_2_Cl_2_:EtOAc = 10:1 (**2**)] gave orange resin of **1** (213 mg, 70%) and orange crystals of **2** (274 mg, 79%), respectively.

*Ac-Fca-Pro-OMe* (**1**). IR (Nujol) ν_max_ 3434 w (NH_free_), 3351, 3231 m (NH_assoc__.),_ 1741 s (C=O_COOMe_), 1676 s (C=O_Ac_), 1604 s (C=O_CONH_), 1535 s (amide II) cm^−1^; R_f_ = 0.30 (CH_2_Cl_2_:EtOAc = 5:1); ^1^H- NMR (CDCl_3_, 600 MHz) δ 8.38 (s, 0.09H, NH*_cis_*), 8.23 (s, 0.91H, NH*_trans_*), 4.80-4.78 (m, 1H, CH_Fn_), 4.62 (dt, 1H, *J*_1_ = 2.9 Hz, *J*_2_ = 1.5 Hz, CH_Fn_), 4.60 (m, 1H, CH_αPro_), 4.60–4.58 (m, 1H, CH_Fn_), 4.54 (dt, 1H, *J*_1_ = 2.7 Hz, *J*_2_ = 1.4 Hz, CH_Fn_), 4.40–4.37 (m, 2H, CH_Fn_), 4.07–4.05 (m, 1H, CH_Fn_), 4.03–4.01 (m, 1H, CH_Fn_), 3.81 (s, 3H, CH_3COOMe_), 3.79–3.73 (m, 2H, CH_δPro_), 2.33–2.28 (m, 1H, CH_βPro_), 2.11–2.08 (m, 1H, CH_γPro_), 2.06 (s, 3H, CH_3Ac_), 1.99–1.93 (m, 2H, CH_βPro_+CH_γPro_); ^13^C-NMR (CDCl_3_, 150 MHz) δ 173.78 (CO_COOMe_), 169.94 (CO_CO__-N_), 169.69 (CO_Ac_), 94.20 (C_qFn_), 79.92 (C_qFn_), 73.22, 71.49, 71.08, 70.46, 66.71, 66.54, 66.18, 64.71 (CH_Fn_*_trans_*), 70.46, 70.18, 70.06, 66.71, 66.42 (CH_Fn_*_cis_*), 60.90 (CH_αPro_*_cis_*), 60.15 (CH_αPro_ *_trans_*), 52.67 (CH_3COOMe_ *_cis_*), 52.53 (CH_3COOMe_ *_trans_*), 49.18 (CH_2δPro_ *_trans_*), 47.52 (CH_2δPro_ *_cis_*), 29.82 (CH_2βPro_ *_cis_*), 29.03 (CH_2βPro_ *_trans_*), 25.83 (CH_2γPro_ *_trans_*), 23.83 (CH_3Ac_), 22.36 (CH_2γPro_ *_cis_*); HRMS (MALDI) *m/z* 398.0939 (calcd for C_19_H_22_N_2_O_4_Fe, 398.0923).

*Boc-Fca-Pro-OMe* (**2**): mp 125–128 °C. IR (Nujol) ν_max_ 3430 m (NH_free_), 3356 sh, 3315 m (NH_assoc__.)_, 1741 sh (C=O_COOMe_), 1718 s (C=O_Boc_), 1604 s (C=O_CONH_), 1532 s (amide II) cm^−1^; R_f_ = 0.50 (CH_2_Cl_2_:EtOAc = 10:1); ^1^H-NMR (CDCl_3_, 600 MHz) δ 7.12 (br. s, 0.93H, NH*_trans_*), 6.80 (br. s, 0.07H, NH*_cis_*), 4.88–4.84 (m, 1H, CH_Fn_), 4.65 (dt, 1H, *J*_1_ = 2.7 Hz, *J*_2_ = 1.5 Hz, CH_Fn_), 4.62 (dd, 1H, *J*_1_ = 8.8 Hz, *J*_2_ = 6.6 Hz, CH_αPro_), 4.42–4.37 (m, 2H, CH_Fn_), 4.37–4.34 (m, 1H, CH_Fn_), 4.07–4.03 (m, 1H, CH_Fn_), 3.98–3.95 (m, 1H, CH_Fn_), 3.87–3.80 (m, 1H, CH_δPro_), 3.81 (s, 3H, CH_3COOMe_), 3.77–3.73 (m, 1H, CH_δPro_), 2.28–2.24 (m, 1H, CH_βPro_), 2.16–2.14 (m, 1H, CH_γPro_), 2.04–1.95 (m, 2H, CH_βPro_+CH_γPro_), 1.49 (s, 9H, CH_3Boc_); ^13^C-NMR (CDCl_3_, 150 MHz) δ 174.01 (CO_COOMe_), 169.77 (CO_CO__-N_), 153.91 (CO_Boc_), 96.79 (C_qFn_), 79.80 (C_qFn_), 76.36 (C_qBoc_), 72.77, 71.86, 71.22, 70.96, 66.09, 65.45, 64.40, 62.75 (CH_Fn_ *_trans_*), 70.64, 70.49, 69.98 (CH_Fn_ *_cis_*), 60.64 (CH_αPro_ *_cis_*), 60.36 (CH_αPro_ *_trans_*), 52.57 (CH_3COOMe_), 48.77 (CH_2δPro_ *_trans_*), 47.50 (CH_2δPro_ *_cis_*), 32.03 (CH_2βPro_ *_cis_*), 28.58 (CH_2βPro_ *_trans_*), 28.57 (CH_3Boc_), 25.98 (CH_2γPro_ *_trans_*), 22.17 (CH_2γPro_ *_cis_*). HRMS (MALDI) *m/z* 456.1353 (calcd for C_22_H_28_N_2_O_5_Fe, 456.1342).

### 3.3. General Procedure for the Preparation of Compounds **3** and **4**

*Boc-Pro-Fca-OMe* (**3**)*and* *Ac-Pro-Fca-OMe* (**4**). HCl·Fca-OMe, obtained from the fully protected Fca (500 mg, 1.4 mmol) by action of gaseous HCl in CH_2_Cl_2_, was treated with NEt_3_ to give unstable free base [22] which was coupled with Boc-Pro-OH (602 mg, 2.8 mmol) using standard EDC/HOBt method. After standard work-up and TLC purification (CH_2_Cl_2_-EtOAc = 5:1), yellow crystals of Boc-Pro-Fca-OMe (**3**, 1060 mg, 83%) were obtained. The transformation of Boc-peptide **3** (400 mg, 0.89 mmol) to Ac-analogue **4** was carried out by action of acetyl chloride (380 μL, 5.34 mmol): the free base, obtained via hydrochloride salt provided by deprotection of **3** in the above described manner, was cooled at 0 °C and acetyl chloride was dropwise added. After stirring in an ice bath for 15 min, TLC indicated no residual starting material remained and the reaction mixture was poured into water and extracted with CH_2_Cl_2_. The combined organic phases were washed with a brine, dried over Na_2_SO_4_ and evaporated to dryness *in vacuo*. The resulting crude product was purified by TLC on silicagel (EtOAc) to give orange solid of **4** (325 mg, 92%).

*Boc-Pro-Fca-OMe* (**3**): mp 128–132 °C; IR IR (Nujol) ν_max_3419 sh, 3405 m (NH_free_), 3290 m, 3234 w (NH_assoc__._), 1706 s (C=O_COOMe_), 1694 s (C=O_Boc_), 1655 s (C=O_CONH_), 1532 s (amide II) cm^−1^; R_f_ = 0.33 (CH_2_Cl_2_:EtOAc = 5:1); ^1^H-NMR* (CDCl_3_, 600 MHz) δ 8.78 (s, 0.66H, NH*_trans_*), 7.53 (s, 0.33H, NH*_cis_*), 4.80–4.77 (m, 0.67H, CH_Fn_ *_trans_*), 4.76 (dt, 0.66H, *J*_1_ = 2.6 Hz, *J*_2_ = 1.3 Hz, CH_Fn_ *_trans_*), 4.74 (dt, 0.66H, *J*_1_ = 2.7 Hz, *J*_2_ = 1.3 Hz, CH_Fn_ *_trans_*), 4.70 (dt, 0.32H, *J*_1_ = 2.5 Hz, *J*_2_ = 1.3 Hz, CH_Fn_ *_cis_*), 4.69 (dt, 0.33H, *J*_1_ = 2.4 Hz, *J*_2_ = 1.2 Hz, CH_Fn_ *_cis_*), 4.64 (dt, 0.32H, *J*_1_ = 2.7 Hz, *J*_2_ = 1.4 Hz, CH_Fn_ *_cis_*), 4.49 (dt, 0.34H, *J*_1_ = 2.7 Hz, *J*_2_ = 1.4 Hz, CH_Fn_ *_cis_*), 4.48–4.45 (m, 0.64H, CH_Fn_ *_trans_*), 4.44–4.42 (m, 0.34H, CH_Fn_ *_cis_*), 4.40–4.38 (m, 0.33H, CH_Fn_ *_cis_*), 4.38–4.37 (m, 0.65H, CH_Fn_ *_trans_*), 4.36–4.33 (m, 1.4H, CH_αPro_ *_trans_*+CH_Fn_ *_trans_*), 4.26 (dd, 0.33H, *J*_1_ = 8.7 Hz, *J*_2_ = 3.4 Hz, CH_αPro_ *_cis_*), 4.11–4.09 (m, 0.32H, CH_Fn_ *_cis_*), 4.09–4.07 (m, 0.32H, CH_Fn_ *_cis_*), 4.02–3.99 (m, 1.32H, CH_Fn_ *_trans_*), 3.79 (s, 0.98H, CH_3__COOMe_ *_cis_*), 3.78 (s, 1.94H, CH_3__COOMe_ *_trans_*), 3.62 (ddd, 0.35H, *J*_1_ = 10.9 Hz, *J*_2_ = 7.4 Hz, *J*_3_ = 3.6 Hz, CH_δ__Pro_*_cis_*), 3.52-3.46 (m, 0.36H, CH_δ__Pro_ *_cis_*), 3.44 (ddd, 0.70H, *J*_1_ = 10.3 Hz, *J*_2_ = 7.2 Hz, *J*_3_ = 2.8 Hz, CH_δ__Pro_ *_trans_*), 3.28 (td, 0.7H, *J*_1_ = 10.2 Hz, *J*_2_ = 7.2 Hz, CH_δ__Pro_ *_trans_*), 2.43–2.38 (m, 0.70H, CH_β__Pro_ *_trans_*), 2.29–2.21 (m, 0.31H, CH_β__Pro_ *_cis_*), 2.21–2.15 (m, 0.34H, CH_β__Pro_ *_cis_*), 2.00–1.90 (m, 2.07H, CH_γ__Pro_ *_trans_*+ CH_γ__Pro_ *_cis_*), 1.90-1.83 (m, 0.63H, CH_β__Pro_ *_trans_*), 1.48 (s, 5.97H, CH_3__Boc_ *_trans_*), 1.45 (s, 3.08H, CH_3__Boc_ *_cis_*). ^13^C-NMR* (CDCl_3_, 150 MHz) δ 172.39 (CO_COOMe_ *_cis_*), 172.21 (CO_COOMe_ *_trans_*), 171.52 (CO_CO__-__NH_ *_cis_*), 170.24 (CO_CO__-__NH_ *_trans_*), 156.34 (CO_Boc_ *_trans_*), 155.06 (CO_Boc_ *_cis_*), 95.30 (C_qFn_ *_trans_*), 93.94 (C_qFn_ *_cis_*), 81.19 (C_qFn_), 80.91 (C_qBoc_ *_trans_*), 73.04, 71.10, 70.69, 66.54, 66.33, 62.54, 62.40, (CH_Fn_ *_trans_*), 72.74, 71.44, 70.95, 66.84, 66.82, 63.77, 62.98 (CH_Fn_ *_cis_*), 72.90 (CH_Fn_), 61.59 (CH_α__Pro_ *_cis_*), 59.83 (CH_α__Pro_ *_trans_*), 52.25 (CH_3__COOMe_ *_cis_*), 52.08 (CH_3__COOMe_ *_trans_*), 47.17 (CH_2__δ__Pro_), 31.21 (CH_2β__Pro_ *_cis_*), 28.33 (CH_3__Boc_), 27.21 (CH_2β__Pro_ *_trans_*), 24.71 (CH_2γ__Pro_ *_trans_*), 23.77 (CH_2γ__Pro_ *_cis_*); HRMS (MALDI) *m/z*456.136 (calcd for C_22_H_28_N_2_O_5_Fe, 456.1342). * Due to the poor signal resolution at the higher temperatures, NMR spectra were recorded at 228 K.

*Ac-Pro-Fca-OMe* (**4**): mp 136–138 °C; IR IR (Nujol) ν_max_ 3416 w (NH_free_), 3272 m, 3229 m (NH_assoc.),_ 1709 s (C=O_COOMe_), 1690 s (C=O_Ac_), 1623 s (C=O_CONH_), 1563 s (amide II) cm^−1^; R_f_ = 0.22 (CH_2_Cl_2_:EtOAc = 5:1); ^1^H-NMR (CDCl_3_, 600 MHz) δ 8.81 (s, 0.92H, NH*_trans_*), 7.29 (s, 0.08H, NH*_cis_*), 4.75–4.72 (m, 2H, CH_Fn_), 4.67–4.65 (m, 2H, CH_Fn+_CH_αPro_), 4.63 (m, 0.06H, CH_Fn_ *_cis_*), 4.56 (dt, 0.93H, *J_1_* = 2.7 Hz, *J_2_* = 1.4 Hz, CH_Fn_ *_trans_*), 4.54–4.53 (m, 0.06H, CH_Fn_ *_cis_*), 4.40–4.39 (m, 0.16H, CH_Fn_ *_cis_*), 4.38–4.36 (m, 1.87H, CH_Fn_ *_trans_*), 4.10–4.08 (m, 0.14H, CH_Fn_ *_cis_*), 4.01–3.99 (m, 1.86H, CH_Fn_ *_trans_*), 3.79 (s, 3H, CH_3COOMe_), 3.63 (ddd, 1H, *J_1_*= 10.3 Hz, *J_2_* = 9.1 Hz, *J_3_* = 3.0 Hz, CH_δPro_), 3.45 (td, 1H, *J_1_* = 9.9 Hz, *J_2_* = 7.0 Hz, CH_δPro_), 2.59–2.56 (m, 1H, CH_βPro_), 2.20–2.15 (m, 1H, CH_γPro_), 2.18 (s, 3H, CH_3Ac_), 2.06–2.01 (m, 1H, CH_γPro_), 1.87–1.81 (m, 1H, CH_βPro_); ^13^C-NMR (CDCl_3_, 150 MHz) δ 171.77 (CO_COOMe_), 171.67 (CO_CO-NH_), 169.30 (CO_Ac_), 95.78 (C_qFn_), 72.29 (C_qFn_), 72.55, 72.53, 71.41, 71.22, 66.33, 66.27, 63.11, 62.99, (CH_Fn_ *_trans_*), 71.73, 71.35, 66.85, 66.72, 64.08, 63.65 (CH_Fn_*_cis_*), 60.15 (CH_αPro_), 51.94 (CH_3COOMe_ *_cis_*), 51.68 (CH_3COOMe_ *_trans_*), 48.51 (CH_2__δPro_ *_trans_*), 47.11 (CH_2__δPro_ *_cis_*), 31.36 (CH_2βPro_ *_cis_*), 26.81 (CH_2βPro_ *_trans_*), 25.23 (CH_3Ac_), 23.22 (CH_2γPro_ *_trans_*), 22.71 (CH_2γPro_ *_cis_*); HRMS (MALDI) *m/z* 398.0936 (calcd for C_19_H_22_N_2_O_4_Fe, 398.0923).

### 3.4. Crystallography

Crystals suitable for single-crystal X-ray diffraction analysis were grown from CDCl_3_ by slow evaporation of the solvent at low temperature (277 K). Single crystal measurements were performed on an Oxford Diffraction Xcalibur Nova R (microfocus Cu tube) at room temperature [293(2) K]. Program package CrysAlis PRO [76] was used for data reduction. The structures were solved using SHELXS97 [77] and refined with SHELXL97 [77]. The models were refined using the full-matrix least squares refinement; all non-hydrogen atoms were refined anisotropically. Hydrogen atoms bound to C atoms were modelled as riding entities using the AFIX command. Molecular geometry calculations were performed by PLATON [78], and molecular graphics were prepared using ORTEP-3 [79], and CCDC-Mercury [80]. Crystallographic and refinement data for the structures reported in this paper are shown in Table 7.

**Table 7:** Crystallographic, data collection and structure refinement details.

Compound	*Boc-Pro-Fca-OMe* *(3)*
Empirical formula	C_22_H_28_FeN_2_O5
Formula wt./g·mol^−1^	456.31
Crystal dimensions/mm	0.08 × 0.06 × 0.03
Space group	*P* 2_1_
*a*/Å	10.951(5)
*b*/Å	8.571(5)
*c*/Å	11.944(5)
α/°	90
β/°	107.315(5)
γ/°	90
Z	2
*V*/Å^3^	1070.3(9)
*D*_calc_/g cm^−3^	1.416
µ/mm^−1^	5.954
*Θ* range/°	3.88–75.86
*T*/K	293(2)
Radiation vawelength	1.54179 (Cu *K*α)
Diffractometer type	Xcalibur Nova
Range of *h*, *k*, *l*	−13 < *h* < 13; −10 < *k* < 10; −11 < *l* < 14
Reflections collected	5054
Independent reflections	3430
Observed reflections ( *I* ≥ 2σ)	3108
Absorption correction	Multi-scan
*R_int_*	0.0507
*R* (*F*)	0.1024
*R_w_*(*F*^2^)	0.2806
Goodness of fit	1.268
H atom treatment	Constrained
No. of parameters	271
No. of restraints	1
Δρ_max_, Δρ_min_ (eÅ^−3^)	1.3091; −1.272

CCDC 1011219 contains the supplementary crystallographic data for this paper. This supplementary crystallographic data can be obtained free of charge via www.ccdc.cam.ac.uk/conts/retrieving.html (or from the Cambridge Crystallographic Data Centre, 12, Union Road, Cambridge CB2 1EZ, UK; Fax: +44 1223 336033; or deposit@ccdc.cam.ac.uk).

### 3.5. Biological Evaluation

#### 3.5.1. Cell Culture and Stock Solutions

MCF-7 cell line derived from human breast adenocarcinoma (ATCC No. HTB-22) and HeLa cell line derived from the human cervical adenocarcinoma (ATCC No. CCL-2) were obtained from the Ruđer Bošković Institute, Zagreb, Croatia. Both cell lines were maintained in Dulbecco’s modified Eagle’s medium (DMEM, Gibco, Paisley, UK) supplemented with 10% heat-inactivated fetal bovine serum (FBS, Gibco) in the incubator at 310 K in humidified atmosphere with 5% CO_2_. Stock solutions of tested compounds **1**–**4** were prepared as 10 mM solutions in ethanol (EtOH) and stored at 277 K. The stock solutions were further diluted with culture medium prior to use in cytotoxicity assay.

#### 3.5.2. Cytotoxicity Assay

The effect of the synthesised compounds **1**–**4** on cell proliferation was examined by the WST-1 assay (Roche, Mannheim, Germany), modification of classical MTT test [81]. MCF-7 and HeLa cells from the exponential growth phase were trypsinized and plated out in 96-well plates at a density of 5 × 10^4^ cells/well in 100 µL of media. After overnight cell growth, the media was replaced with fresh one containing different concentrations of individual compound (1–500 µM). The appropriate volume of EtOH was added to control cells, but the final concentration of EtOH in the control and treated cells was less than 0.2% and had no interference with the biological activities tested. Following exposure for 72 h, 10 µL of tetrazolium salt WST-1 {4-[3-(4-Iodophenyl)-2-(4-nitrophenyl)-2*H*-5-tetrazolio]-1,3-benzene disulfonate) was added to each well and cells were incubated for the further 4 h. The absorbance was measured at 450 nm on the microplate reader (Tecan, Mannedorf, Switzerland). The experiments were performed three times with four parallels for each concentration and data were expressed as the means ± S.D. Cell viability was presented as percentage of treated cells *vs.* control cells by setting the viability of control cell as 100%. The IC_50_ values, defined as the concentration of tested compound that resulted with 50% growth inhibition, were calculated from the dose-response curves using equations of best-fitted trend-lines.

#### 3.5.3. Staining with Crystal-Violet

Light microscopy was used to observe the morphological changes during the treatment. MCF-7 cells (1 × 10^5^ cells/mL) were seeded in the plate with 6 wells and after overnight cell growth exposed to the highest concentration of tested compounds (500 µM) for 72 h. The cells were then washed with PBS and stained with crystal-violet dye solution (Sigma–Aldrich, St. Louis, MO, USA). Images of control and treated MCF-7 cells were taken using an inverted microscope (Carl Zeiss, Göttingen, Germany) and Dino-Eye digital camera.

## 4. Conclusions

Four novel conjugates **1**–**4** of Fca and proline have been prepared. Their conformations in solution have been elucidated using IR, NMR and CD spectroscopic methods. In addition, the crystal structure of peptide **3** has been resolved.

Due to lack of the amide proton at the lower Cp ring of **1** and **2**, their stabilization by intramolecular hydrogen bonding has been provided through *interchain* interaction of NH_Fca_ and OC_Pro_ (9-membered IHB ring) which corresponds to IHB pattern of the type **A** presented in their alanine analogues **III**. Hence, the conformation **B** founded in **III** has not been realized. The alteration of peptides **1** and **2** into **3** and **4**, respectively, notably influenced their conformational settings. The same donor (NH_Fca_) and acceptor (OC_Pro_) were engaged in hydrogen bonding, but in a different manner, *i*.*e*., an *intrachain* IHBs have been founded. With respect to their alanine analogues **IV**, stabilized by three alternative patterns of hydrogen bonding, peptides **3** and **4** have been adopted 7-membered IHB ring (γ-turn) correlated to **IVA** conformation. Although it has been shown that hydrogen bonding is somewhat difficult in the presence of the bulky Boc group, its replacement with an Ac group did not cause significant conformational changes.

The biological evaluation of peptides **1**–**4**, performed with regard to antiproliferative effect on MCF-7 and HeLa cell line, has been revealed no or rather modest cytotoxic effects only at the maximal tested concentration in the high micromolar range. Those results are not satisfactory if we consider the tested compounds as promising cytotoxic agents candidates, but are valuable as a guideline to direct the synthesis of the corresponding analogues with improved biological properties. The goal of the presented biological evaluation was to find lead structures as a starting point for future search of optimized drug candidates what will surely be our further scientific interest.

## Supplementary Materials

Supplementary materials (HRMS, ^1^H and ^13^C NMR spectra of novel compounds **1**–**4**, ^1^H NMR titration of compounds **1**–**4** with DMSO in CDCl_3_, Variable-temperature ^1^H NMR spectra of **1**–**4** and checkCIF_PLATON report of compound **3**) can be accessed at: http://www.mdpi.com/1420-3049/19/8/12852/s1.

## Supplementary File

Supplementary File 1

## References

[1] Ko E., Liu J., Burgess K. (2011). Minimalist and universal peptidomimetics. Chem. Soc. Rev..

[2] Giannis A., Kolte T. (1993). Peptidomimetics for Receptor Ligands-Discovery, Development, and Medical Perspectives. Angew. Chem. Int. Ed. Engl..

[3] Vagner J., Qu H., Hruby V.J. (2008). Peptidomimetics, a synthetic tool of drug discovery. Curr. Opin. Chem. Biol..

[4] Kahn M. (1993). Peptide Secondary Structure Mimetics: Recent Advances and Future Challenges. Synlett.

[5] Olson G.L., Bolin D.R., Bonner M.P., Bos M., Cook C.M., Fry D.C., Graves B.J., Hatada M., Hill D.E. (1993). Concepts and Progress in the Development of Peptide Mimetics. J. Med. Chem..

[6] Nomoto A., Moriuchi T., Yamazaki S., Ogawa A., Hirao T. (1998). A Highly Ordered Ferrocene System Regulated by Podand Peptide Chains. Chem. Commun..

[7] Moriuchi T., Nomoto A., Yoshida K., Ogawa A., Hirao T. (2001). Chirality Organization of Ferrocenes Bearing Podand Dipeptide Chains: Synthesis and Structural Characterization. J. Am. Chem. Soc..

[8] Moriuchi T., Nagai T., Hirao T. (2005). Chirality Organization of Ferrocenes Bearing Dipeptide Chains of Heterochiral Sequence. Org. Lett..

[9] Moriuchi T., Nagai T., Hirao T. (2006). Induction of γ-Turn-Like Structure in Ferrocene Bearing Dipeptide Chains via Conformational Control. Org. Lett..

[10] Moriuchi T., Nomoto A., Yoshida K., Hirao T. (1999). Characterization of Ferrocene Derivatives Bearing Podand Dipeptide Chains (-l-Ala-l-Pro-OR). J. Organomet. Chem..

[11] Moriuchi T., Nomoto A., Yoshida K., Hirao T. (2001). Intramolecular Conformational Control in Ferrocenes Bearing Podand Dipeptide Chains. Organometallics.

[12] Moriuchi T., Hirao T. (2010). Design of Ferrocene-Dipeptide Bioorganometallic Conjugates to induce Chirality-Organized Structures. Acc. Chem. Res..

[13] Moriuchi T., Hirao T. (2012). Dipeptide-induced chirality organization. J. Inc. Phenom. Macrocycl. Chem..

[14] Chowdhury S., Mahmoud K.A., Schatte G., Kraatz H.-B. (2005). Amino acid conjugates of 1,1′-diaminoferrocene. Synthesis and chiral organization. Org. Biomol. Chem..

[15] Djaković S., Siebler D., Čakić Semenčić M., Heinze K., Rapić V. (2008). Spectroscopic and theoretical study of asymmetric 1,1'-diaminoferrocene conjugates of alpha-amino acids. Organometallics.

[16] Barišić L., Dropučić M., Rapić V., Pritzkow H., Kirin S.I., Metzler-Nolte N. (2004). The first oligopeptide derivative of 1'-aminoferrocene-1-carboxylic acid shows helical chirality with antiparallel strands. Chem. Commun..

[17] Barišić L., Čakić M., Mahmoud K.A., Liu Y.-N., Kraatz H.-B., Pritzkow H., Kirin S.I., Metzler-Nolte N., Rapić V. (2006). Helically chiral ferrocene peptides containing 1'-amino-ferrocene-1-carboxylic acid subunit as turn inducers. Chem. Eur. J..

[18] Barišić L., Rapić V., Metzler-Nolte N. (2006). Incorporation of the unnatural organometallic amino acid 1'-aminoferrocene-1-carboxylic acid (Fca) into oligopeptides by a combination of Fmoc and Boc solid phase synthetic methods. Eur. J. Inorg. Chem..

[19] Čakić Semenčić M., Siebler D., Heinze K., Rapić V. (2009). Bis- and Trisamides Derived from 1'-Aminoferrocene-1-carboxylic Acid and alpha-Amino Acids: Synthesis and Conformational Analysis. Organometallics.

[20] akić Semenčić M., Heinze K., Förster C., Rapić V. (2010). Bioconjugates of 1'-Aminoferrocene-1-carboxylic Acid with (*S*)-3-Amino-2-methylpropanoic Acid and l-Alanine. Eur. J. Inorg. Chem..

[21] Lapić J., Siebler D., Heinze K., Rapić V. (2007). Conformational Analysis of Heteroannularly Substituted Ferrocene Oligoamides. Eur. J. Inorg. Chem..

[22] Barišić L., Kovačević M., Mamić M., Kodrin I., Mihalić Z., Rapić V. (2012). Synthesis and Conformational Analysis of Methyl *N*-Alanyl-1'-aminoferrocene-1-carboxylate. Eur. J. Inorg. Chem..

[23] Donoli A., Marcuzzo V., Moretto A., Cardena R., Santi S., Toniolo C. (2011). Charge mapping in peptide chains by oxidation of the terminal ferrocenyl group. Org. Lett..

[24] Donoli A., Marcuzzo V., Moretto A., Crisma M., Toniolo C., Cardena R., Bisello A., Santi S. (2013). New bis-Ferrocenyl End-Capped Peptides: Synthesis and Charge Transfer Properties. J. Pept. Sci..

[25] Barišić L., Rapić V., Kovač V. (2002). Ferrocene Compounds. XXIX Efficient Syntheses of 1'-Aminoferrocene-1-carboxylic Acid Derivatives. Croat. Chem. Acta.

[26] Kirin S.I., Kraatz H.-B., Metzler-Nolte N. (2006). Systematizing structural motifs and nomenclature in 1,*n*'-disubstituted ferrocene peptides. Chem. Soc. Rev..

[27] Vanhoof G., Goossens F., De Meester I., Hendriks D., Scharpé S. (1995). Proline motifs in peptides and their biological processing. FASEB J..

[28] Kay B.K., Williamson M.P., Sudol M. (2000). The importance of being proline: The interaction of proline-rich motifs in signalling proteins with their cognate domains. FASEB J..

[29] Troganis A., Gerothanassis I.P., Athanassiou Z., Mavromoustakos T., Hawkes G.E., Sakarellos C. (2000). Thermodynamic origin of cis/trans isomers of a proline-containing beta-turn model dipeptide in aqueous solution: A combined variable temperature ^1^H-NMR, two-dimensional ^1^H,^1^H gradient enhanced nuclear Overhauser effect spectroscopy (NOESY), one-dimensional steady-state intermolecular ^13^C,^1^H NOE, and molecular dynamics study. Biopolymers.

[30] Ganesh S., Jayakumar R. (2003). Role of N-t-Boc group in helix initiation in a novel tetrapeptide. J. Peptide Res..

[31] Ishimoto B., Tonan K., Ikawa S. (1999). Coupling of intramolecular hydrogen bonding to the cis-to-trans isomerization of a proline imide bond of small model peptides. Spectrochim. Acta A Mol. Biomol. Spectrosc..

[32] Sugawara M., Tonan K., Ikawa S. (2001). Effect of solvent on the *cis–trans* conformational. equilibrium of a proline imide bond of short model peptides. Spectrochim. Acta A Mol. Biomol. Spectrosc..

[33] Yusuke J., Tonan K., Ikawa S. (2002). Competitive formation of 10*-* and 7*-*membered hydrogen-bonded rings of proline-containing model peptides. Spectrochim. Acta A Mol. Biomol. Spectrosc..

[34] Tonan K., Ikawa S. (1996). Intramolecular Hydrogen Bonding and Conformation of Small Peptides: Variable-Temperature FTIR Study on *N-*Acetyl*-*L*-*Pro*-*L*-*Leu*-*Gly*-*NH_2_ and Related Compounds. J. Am. Chem. Soc..

[35] Ananthanarayanan V.S., Cameron T.S. (1988). Proline*-*containing beta-turns. IV. Crystal and solution conformations of tert*-*butyloxycarbonyl*-*l*-*prolyl*-*d*-*alanine and tert-butyloxycarbonyl*-*l*-*prolyl*-*d*-*alanyl*-*l*-*alanine. Int. J. Peptide Protein Res..

[36] Ning L., de-Ning W., Sheng-Kang Y. (1996). Hydrogen bonding between urethane and urea: band assignment for the carbonyl region of *FT*i.r. spectrum. Polymer.

[37] Dorman D.E., Bovey F.A. (1973). Carbon*-*13 magnetic resonance spectroscopy. Spectrum of proline in oligopeptides. J. Org. Chem..

[38] Dumy P., Keller M., Ryan D.E., Rohwedder B., Wohr T., Mutter M. (1997). Pseudo-Prolines as a Molecular Hinge: Reversible Induction of *cis* Amide Bonds into Peptide Backbones. J. Am. Chem. Soc..

[39] Llinás M., Klein M.P. (1975). Charge relay at the peptide bond. A proton magnetic resonance study of solvation effects on the amide electron density distribution. J. Am.Chem.Soc..

[40] Kessler H. (1982). Conformation and Biological Activity of Cyclic Peptides. Angew. Chem. Int. Ed. Engl..

[41] Iqbal M., Balaram P. (1982). Aggregation of apolar peptides in organic solvents. Concentration dependence of ^1^H-nmr parameters for peptide NH groups in 3_10_ helical decapeptide fragment of suzukacillin. Biopolymers.

[42] Vijayakumar E.K.S., Balaram P. (1983). Stereochemistry of α-Aminoisobutyric Acid Peptides in Solution: Helical Conformations of Protected Decapeptides with Repeating Aib-l-Ala and Aib-l-Val Sequences. Biopolymers.

[43] Andersen N.H., Neidigh J.W., Harris S.M., Lee G.M., Liu Z., Tong H. (1997). Extracting Information from the Temperature Gradients of Polypeptide NH Chemical Shifts. 1. The Importance of Conformational Averaging. J. Am. Chem. Soc..

[44] Baxter N.J., Williamson M.P. (1997). Temperature dependence of 1H chemical shifts in proteins. J. Biomol. NMR.

[45] Lee H.-J., Park H.-M., Lee K.-B. (2007). The beta-turn scaffold of tripeptide containing an azaphenylalanine residue. Biophys. Chem..

[46] Appoh F.E., Sutherland T.C., Kraatz H.-B. (2004). Changes in the hydrogen bonding pattern in ferrocene peptides. J. Organomet. Chem..

[47] Lapić J., Pavlović G., Siebler D., Heinze K., Rapić V. (2008). Structural, spectroscopic and theoretical study of ferrocene ureidopeptides. Organometallics.

[48] Stevens E.S., Sugawara N., Bonora G.M., Toniolo C. (1980). Conformational analysis of linear peptides. 3. Temperature dependence of NH chemical shifts in chloroform. J. Am. Chem. Soc..

[49] Srivastava K.R., Kumar A., Goyal B., Durani S. (2011). Stereochemistry and solvent role in protein folding: Nuclear magnetic resonance and molecular dynamics studies of poly-l and alternating-l,d homopolypeptides in dimethyl sulfoxide. J. Phys. Chem. B..

[50] Sladojevich F., Guarna A., Trabocchi A. (2010). Evaluation of stereochemically dense morpholine-based scaffolds as proline surrogates in β-turn peptides. Org. Biomol. Chem..

[51] Madison V., Schellman J. (1970). Location of proline derivatives in conformational space. I. Conformational calculations; optical activity and NMR experiments. Biopolymers.

[52] Montagut M., Lemanceau B., Bellocq A.-M. (1974). Conformational analysis of thyrotropin releasing factor by proton magnetic resonance spectroscopy. Biopolymers.

[53] Higasijima T., Tasumi M., Miyazawa T. (1977). ^1^H nuclear magnetic resonance studies of *N*-acetyl-l-proline *N*-methylamide. Molecular conformations, hydrogen bondings, and thermodynamic quantities in various solvents. Biopolymers.

[54] Madison V., Kopple K.D. (1980). Solvent-dependent conformational distributions of some dipeptides. J. Am. Chem. Soc..

[55] Eberhardt E.S., Loh S.N., Hinck A.P., Raines R.T. (1992). Solvent Effects on the Energetics of Prolyl Peptide Bond Isomerization. J. Am. Chem. Soc..

[56] Eberhardt E.S., Loh S.N., Raines R.T. (1993). Thermodynamic Origin of Prolyl Peptide Bond Isomers. Tetrahedron Lett..

[57] Eberhardt E.S., Raines R.T. (1994). Amide-Amide and Amide-Water Hydrogen Bonds: Implications for Protein Folding and Stability. J. Am. Chem. Soc..

[58] McDonald D.Q., Still W.C. (1996). Molecular Mechanics Parameters and Conformational Free Energies of Proline-Containing Peptides. J. Org. Chem..

[59] Eberhardt E.S., Panisik N., Raines R.T. (1996). Inductive Effects on the Energetics of Prolyl Peptide Bond Isomerization: Implications for Collagen Folding and Stability. J. Am. Chem. Soc..

[60] Deetz M.J., Fahey J.E., Smith B.D. (2001). NMR studies of hydrogen bonding interactions with secondary amide and urea groups. J. Phys. Org. Chem..

[61] Sarkar S.K., Young P.E., Sullivan C.E., Torchia D.A. (1984). Detection of cis and trans X-Pro peptide bonds in proteins by ^13^C NMR: Application to collagen. Proc. Natl. Acad. Sci. USA.

[62] O’Neal K.D., Chari M.V., Mcdonald C.H., Cook R.G., Yu-Lee L.Y., Morrisett J.D., Shearer W.T. (1996). Multiple cis-trans conformers of the prolactin receptor proline-rich motif (PRM) peptide detected by reverse-phase HPLC, CD and NMR spectroscopy. Biochem J..

[63] Husain R.D., McCandless J., Stevenson P.J., Large T., Guthrie D.J.S., Walker B. (2002). Detection of cis-trans isomers of a synthetic peptide fragment of Erythropoietin. J. Chromatogr. Sci..

[64] Bragg R.A., Clayden J., Morris G.A., Pink J.H. (2002). Stereodynamics of bond rotation in tertiary aromatic amides. Chem. Eur. J..

[65] Berggren K., Vindebro R., Bergström C., Spoerry C., Persson H., Fex T., Kihlberg J., von Pawel-Rammingen U., Luthman K. (2012). 3-Aminopiperidine-Based Peptide Analogues as the First Selective Noncovalent Inhibitors of the Bacterial Cysteine Protease IdeS. J. Med. Chem..

[66] Kelly S.M., Price N.C. (2000). The Use of Circular Dichroism in the Investigation of Protein Structure and Function. Curr. Protein Pept. Sci..

[67] Kovač V., Čakić Semenčić M., Kodrin I., Roca S., Rapić V. (2013). Ferrocene-dipeptide conjugates derived from aminoferrocene and 1-acetyl-1'-aminoferrocene: Synthesis and conformational studies. Tetrahedron.

[68] Jios J.L., Kirin S.I., Buceta N.N., Weyhermüller T., della Védova C.O., Metzler-Nolte N. (2007). Synthesis and structural characterization of metallated bioconjugates: C-terminal labeling of amino acids with aminoferrocene. J. Organomet. Chem..

[69] Byun Y.S., Lightner D.A. (1991). Exciton coupling from dipyrrinone chromophores. J. Org. Chem..

[70] Kharb R., Rana M., Sharma P.C., Yar M.S. (2011). Therapeutic importance of peptidomimetics in medicinal chemistry. J. Chem. Pharm. Res..

[71] Li L., Thomas R.M.M., Suzuki H., de Brabander J.K., Wang X., Harran P.G. (2004). A small molecule Smac mimic potentiates TRAIL- and TNFalpha-mediated cell death. Science.

[72] Liao Y.-F., Wang B.-J., Hsu W.-M., Lee H., Liao C.-Y., Wu S.-Y., Cheng H.-T., Hu M.K. (2007). Unnatural Amino Acid-Substituted (Hydroxyethyl)urea Peptidomimetics Inhibit γ-Secretase and Promote the Neuronal Differentiation of Neuroblastoma Cells. Mol. Pharmacol..

[73] Boyd M.R., Paull K.D. (1995). Some Practical Considerations and Applications of the National Cancer Institute *in Vitro* Anticancer Drug Discovery Screen. Drug. Develop. Res..

[74] Cortés R., Crespo M., Davin L., Martín R., Quirante J., Ruiz D., Messeguer R., Calvis C., Baldomà L., Badia J. (2012). Seven-membered cycloplatinated complexes as a new family of anticancer agents. X-ray characterization and preliminary biological studies. Eur. J. Med. Chem..

[75] Bayoumi A.H. (2012). Antiproliferative Properties of Vinyl Dipeptides: Synthesis and MCF-7 Cell Line Testing. Open J. Med. Chem..

[76] (2007). CrysAlis PRO.

[77] Sheldrick G.M. (2008). A short history of SHELX. Acta Crystallogr..

[78] Spek A.L. (2003). Single-crystal structure validation with the program *PLATON*. J. Appl. Cryst..

[79] Farrugia L.J. (1997). ORTEP-3 for Windows-A version of ORTEP-III with a Graphical User Interface (GUI). J. Appl. Cryst..

[80] Macrae C.F., Edgington P.R., McCabe P., Pidcock E., Shields G.P., Taylor R., Towler M., van de Streek J. (2006). Mercury: visualization and analysis of crystal structures. J. Appl. Cryst..

[81] Mosmann T. (1983). Rapid colorimetric assay for cellular growth and survival, application to proliferation and cytotoxic assays. J. Immunol. Methods.

