# Supplementary material for: Conjugates of 1'-Aminoferrocene-1-carboxylic Acid and Proline: Synthesis, Conformational Analysis and Biological Evaluation
Source: Molecules. 2014 Aug 21;19(8):12852–80. doi: 10.3390/molecules190812852 (PMC6271532; doi:10.3390/molecules190812852)

Supplementary Information

Compound 1

Figure S1. MALDI TOF/TOF-MS spectrum of **1**.

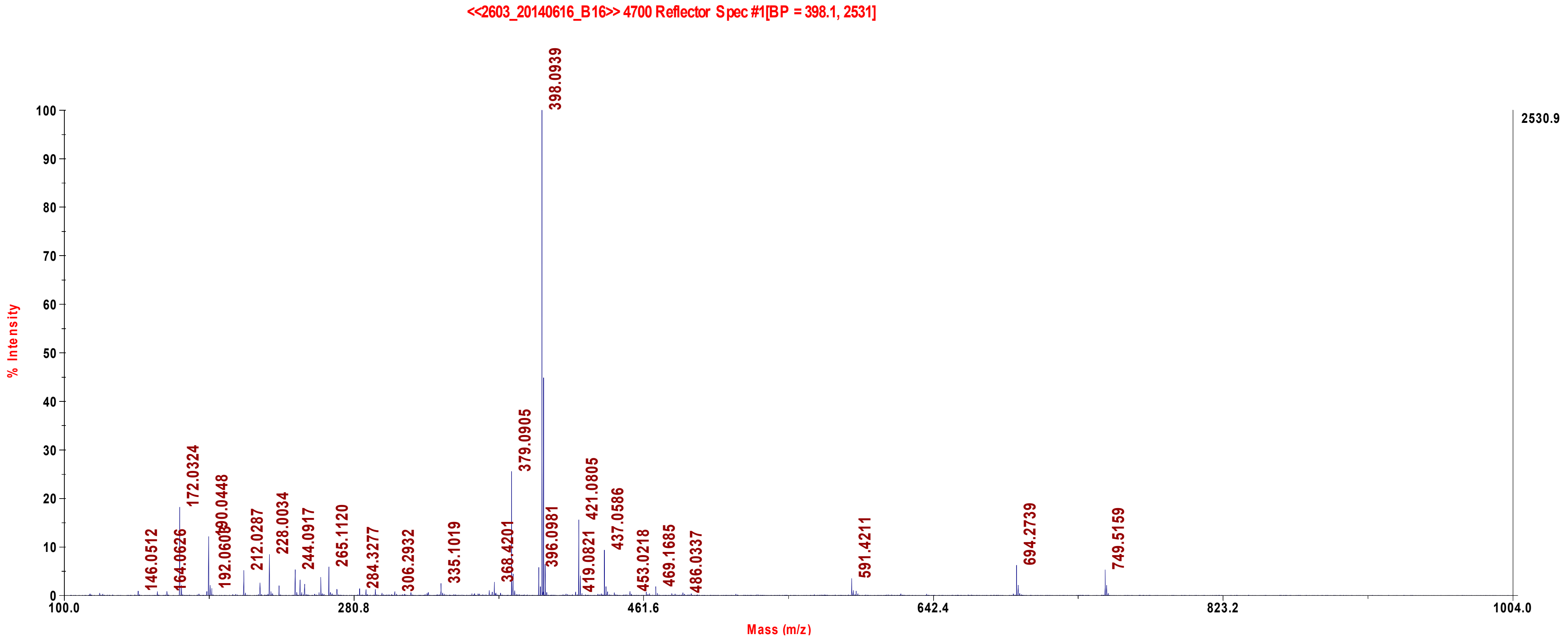

Figure S2. ^{1}H NMR spectra of **1**.

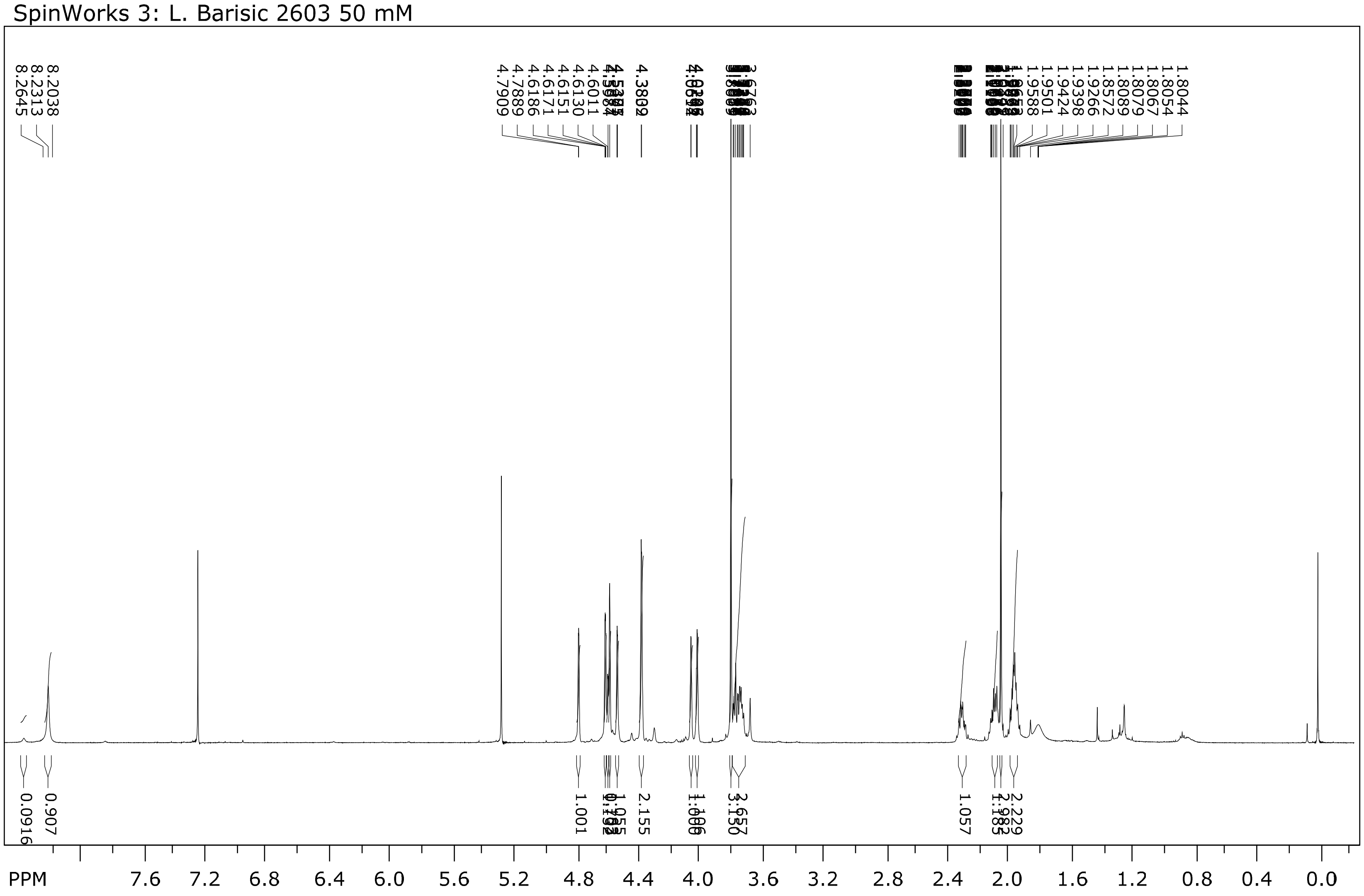

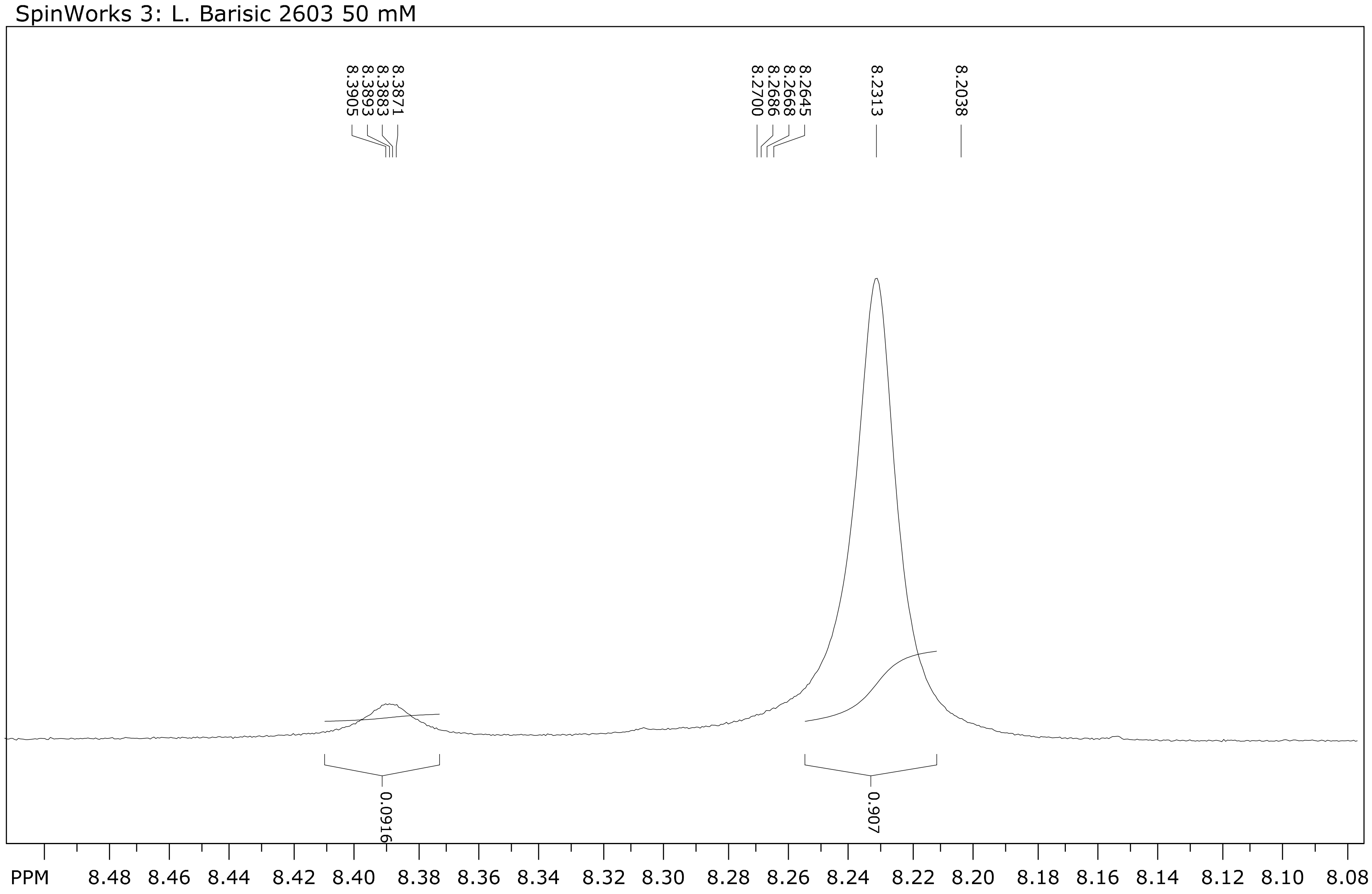
SpinWorks 3: L. Barisic 2603 50 mM
8.3905
8.3893
8.3883
8.3871
8.2700
8.2686
8.2668
8.2645
8.2313
8.2038
0.0916
0.907
PPM
8.48 8.46 8.44 8.42 8.40 8.38 8.36 8.34 8.32 8.30 8.28 8.26 8.24 8.22 8.20 8.18 8.16 8.14 8.12 8.10 8.08

SpinWorks 3: L. Barisic 2603 50 mM

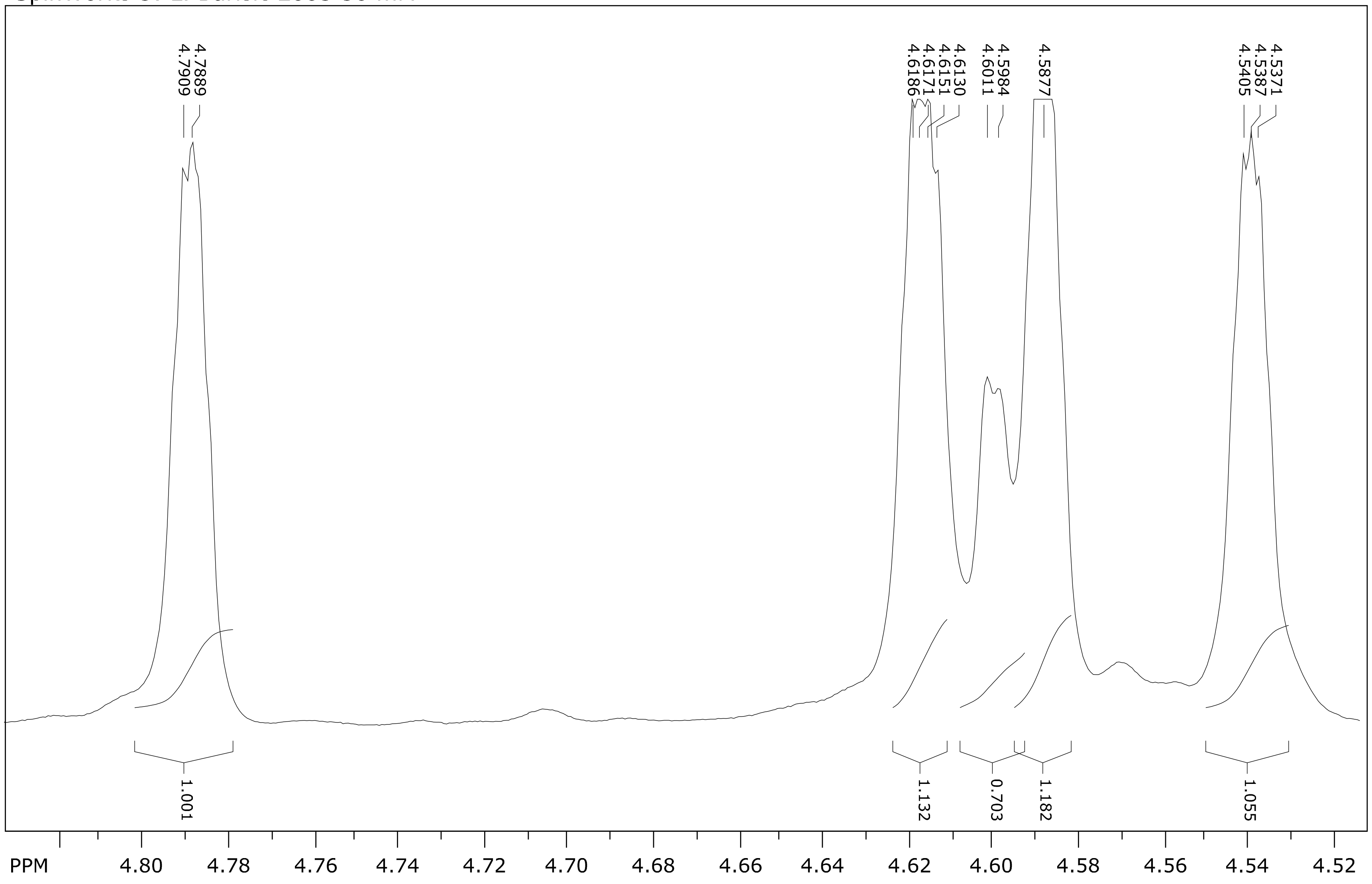

SpinWorks 3: L. Barisic 2603 50 mM

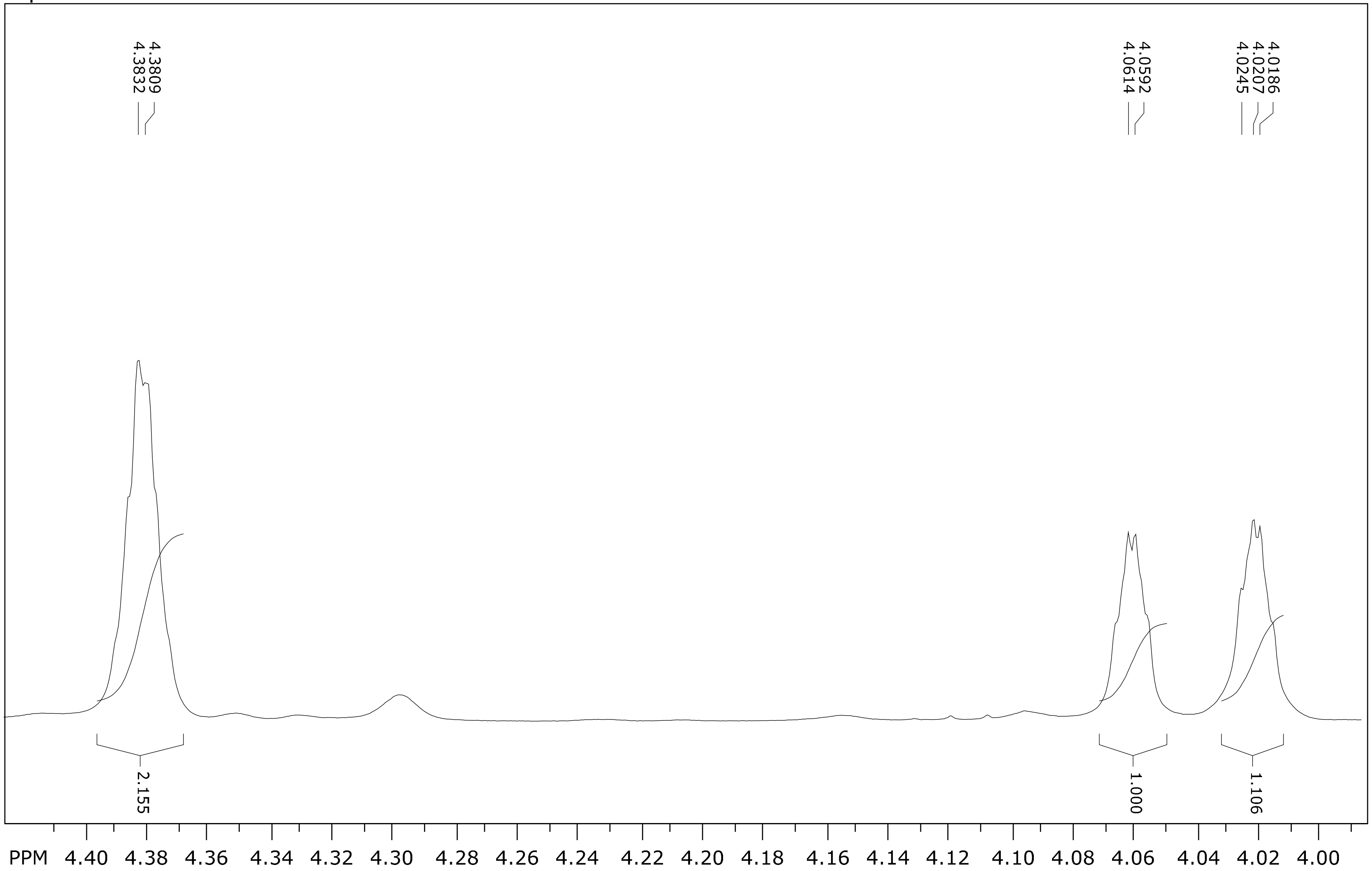

SpinWorks 3: L. Barisic 2603 50 mM

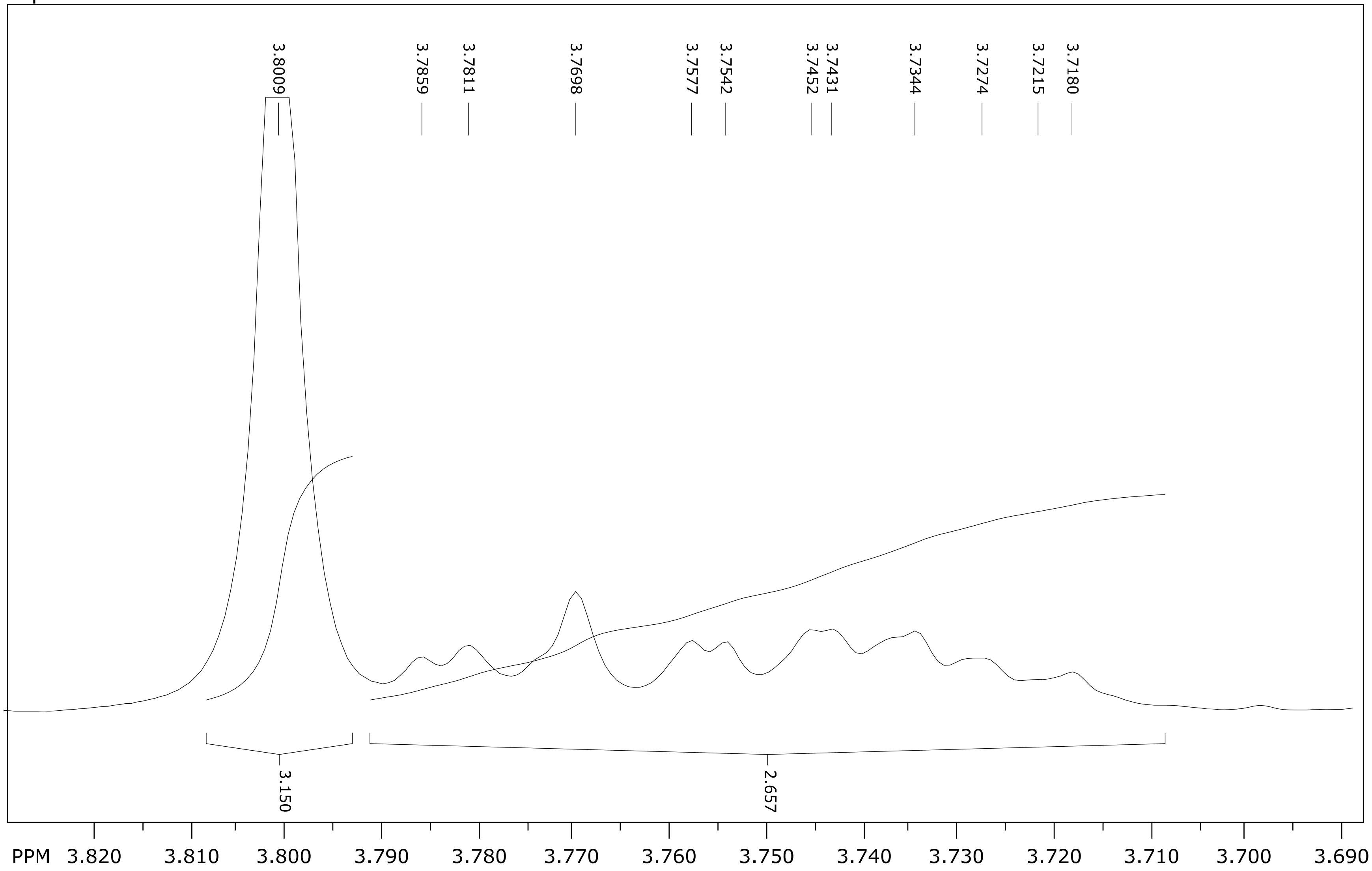

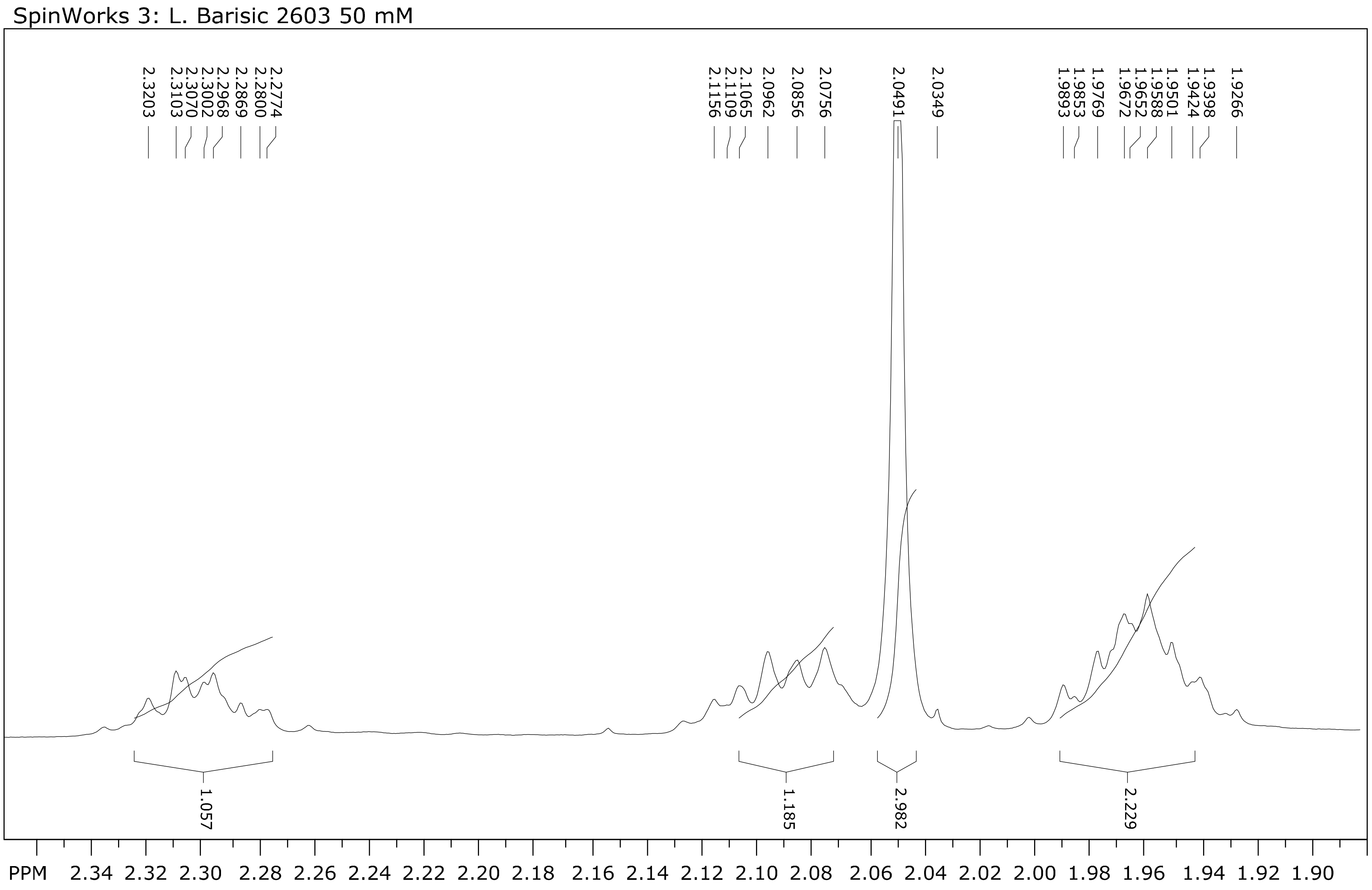
SpinWorks 3: L. Barisic 2603 50 mM
2.3203
2.3103
2.3070
2.3002
2.2968
2.2869
2.2800
2.2774
2.1156
2.1109
2.1065
2.0962
2.0856
2.0756
2.0491
2.0349
1.9893
1.9853
1.9769
1.9672
1.9652
1.9588
1.9501
1.9424
1.9398
1.9266
1.057
1.185
2.982
2.229
PPM 2.34 2.32 2.30 2.28 2.26 2.24 2.22 2.20 2.18 2.16 2.14 2.12 2.10 2.08 2.06 2.04 2.02 2.00 1.98 1.96 1.94 1.92 1.90

Figure S3. ^{13}C NMR spectra of **1**.

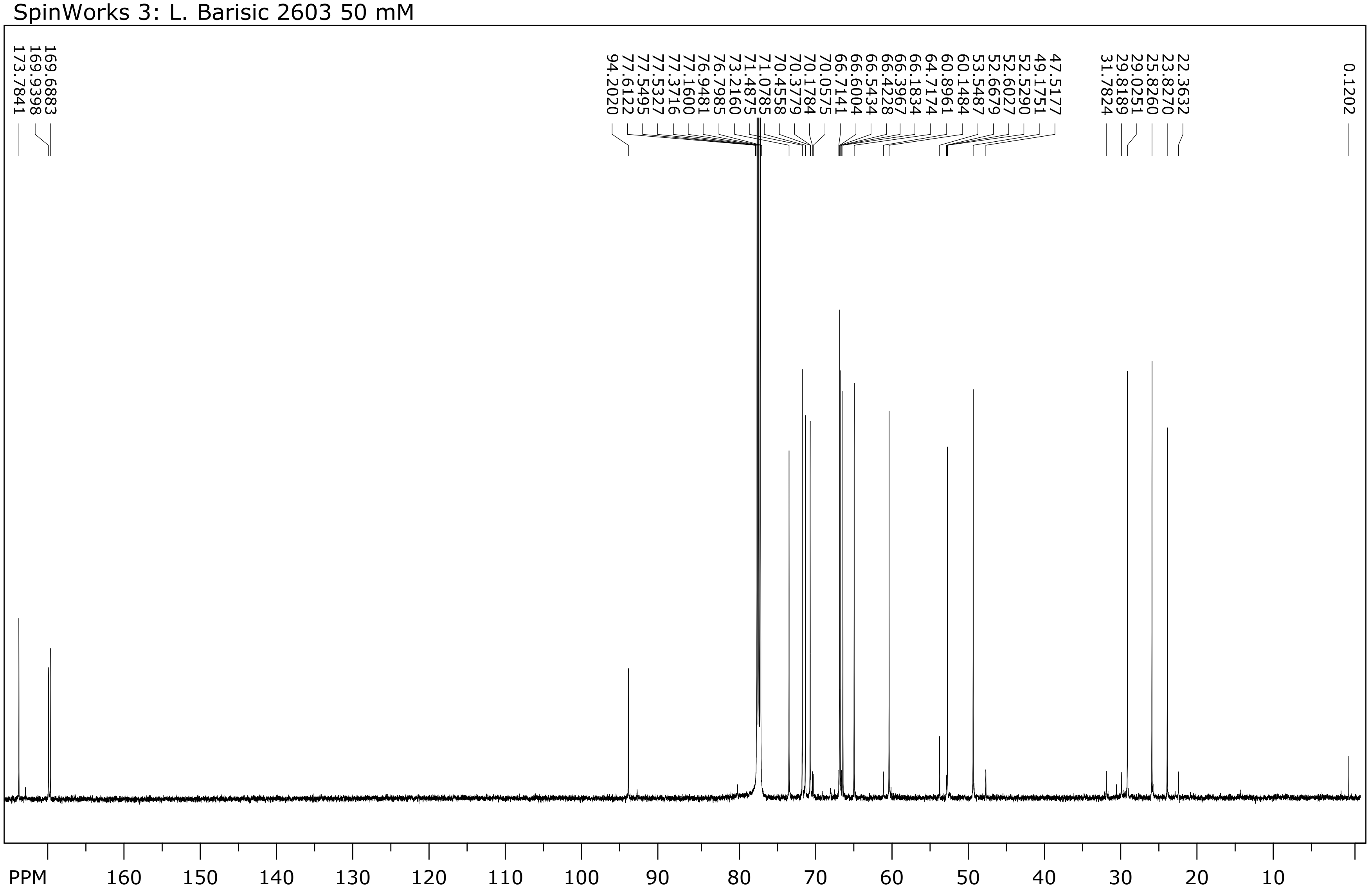

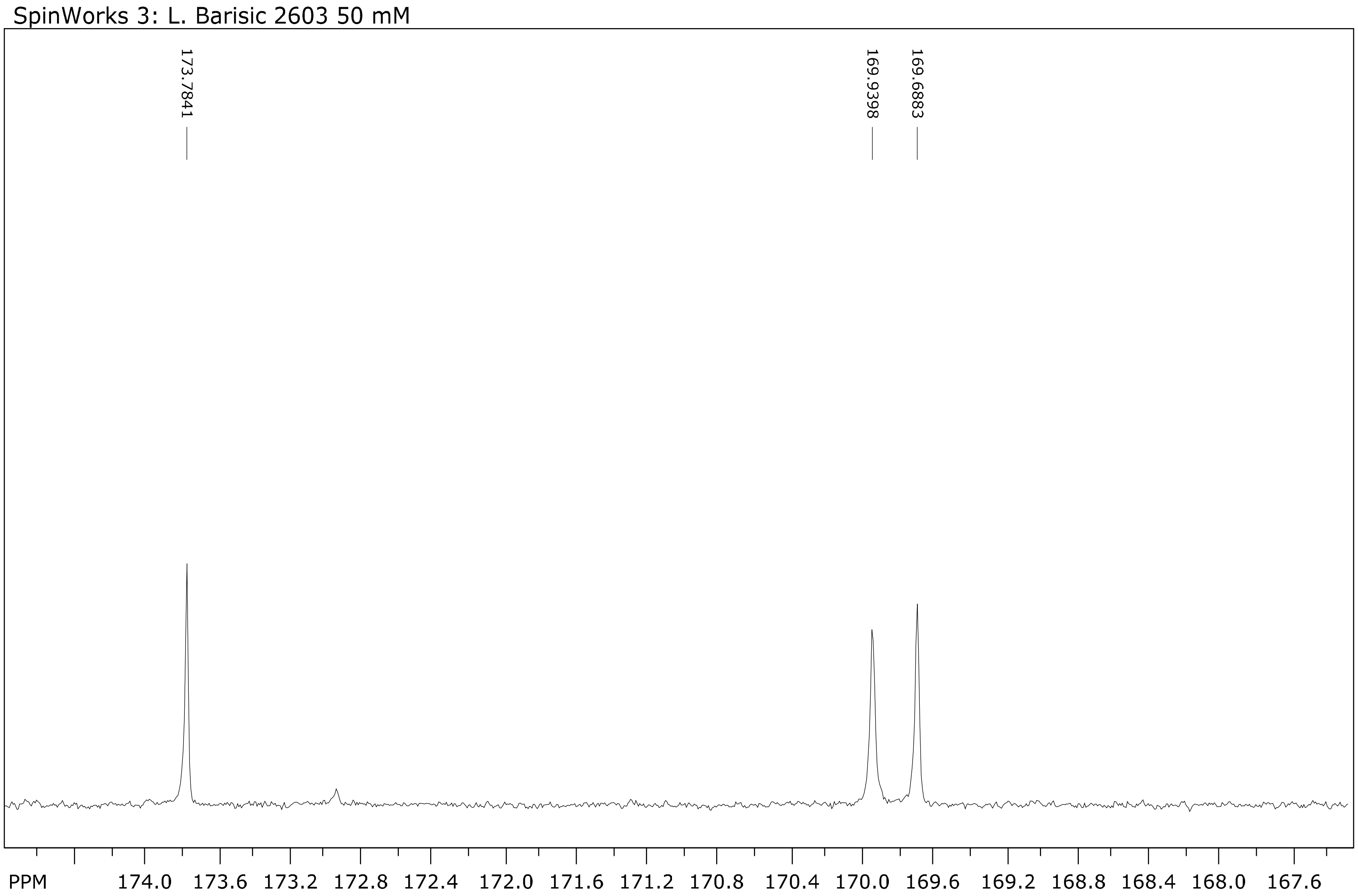
SpinWorks 3: L. Barisic 2603 50 mM
173.7841
169.9398
169.6883
PPM
174.0
173.6
173.2
172.8
172.4
172.0
171.6
171.2
170.8
170.4
170.0
169.6
169.2
168.8
168.4
168.0
167.6

SpinWorks 3: L. Barisic 2603 50 mM

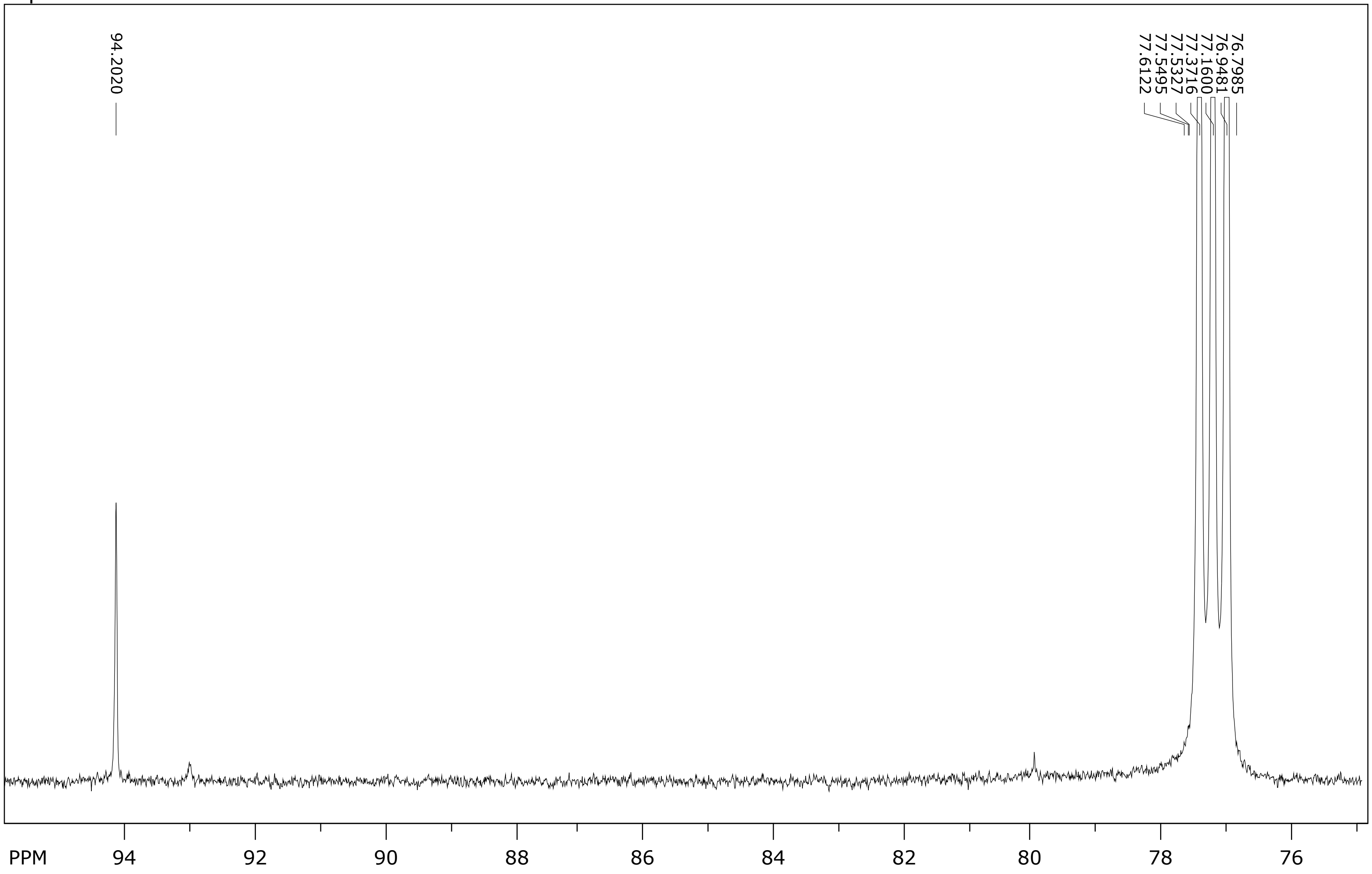

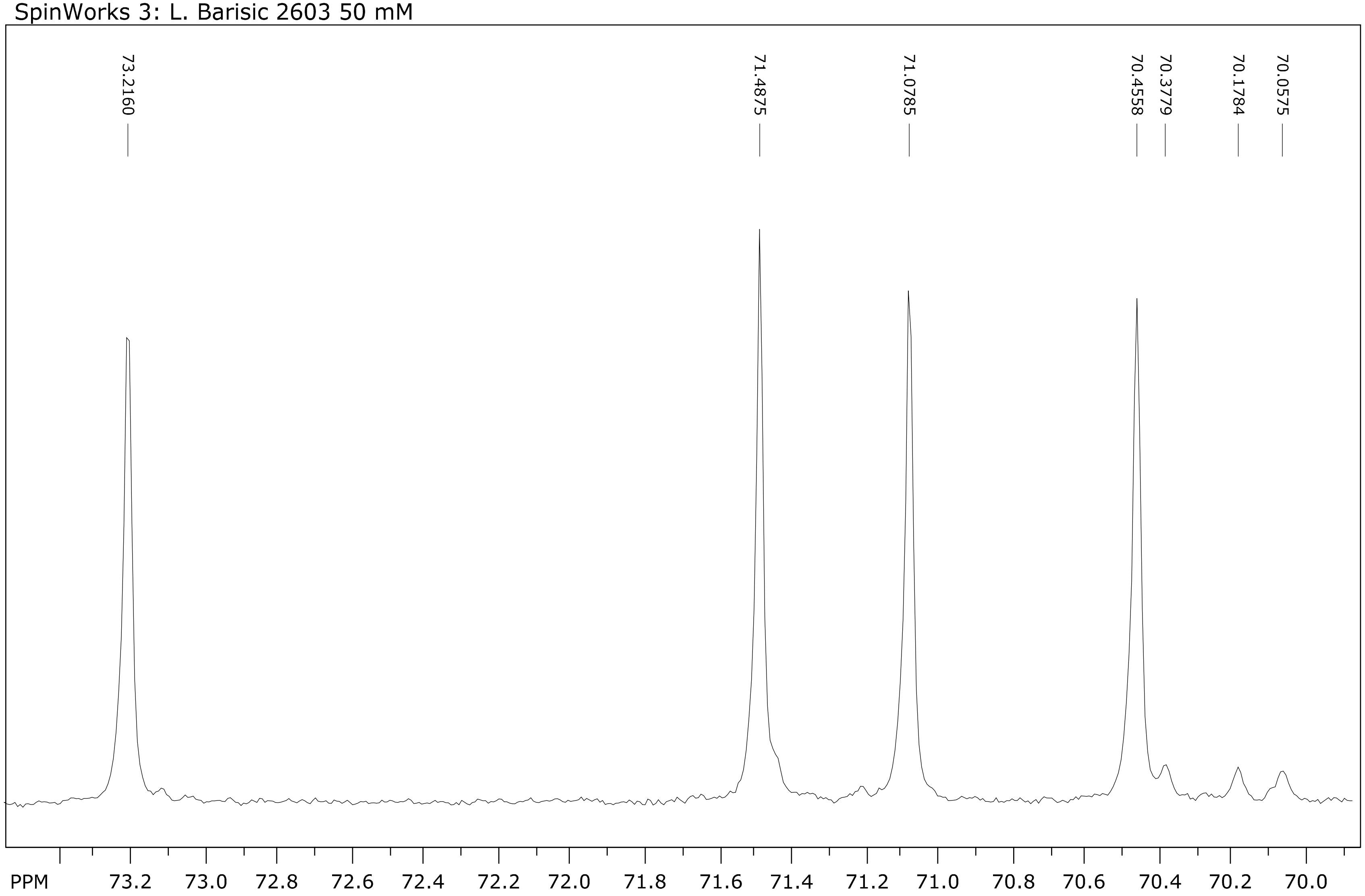
SpinWorks 3: L. Barisic 2603 50 mM
73.2160
71.4875
71.0785
70.4558
70.3779
70.1784
70.0575
PPM
73.2
73.0
72.8
72.6
72.4
72.2
72.0
71.8
71.6
71.4
71.2
71.0
70.8
70.6
70.4
70.2
70.0

SpinWorks 3: L. Barisic 2603 50 mM

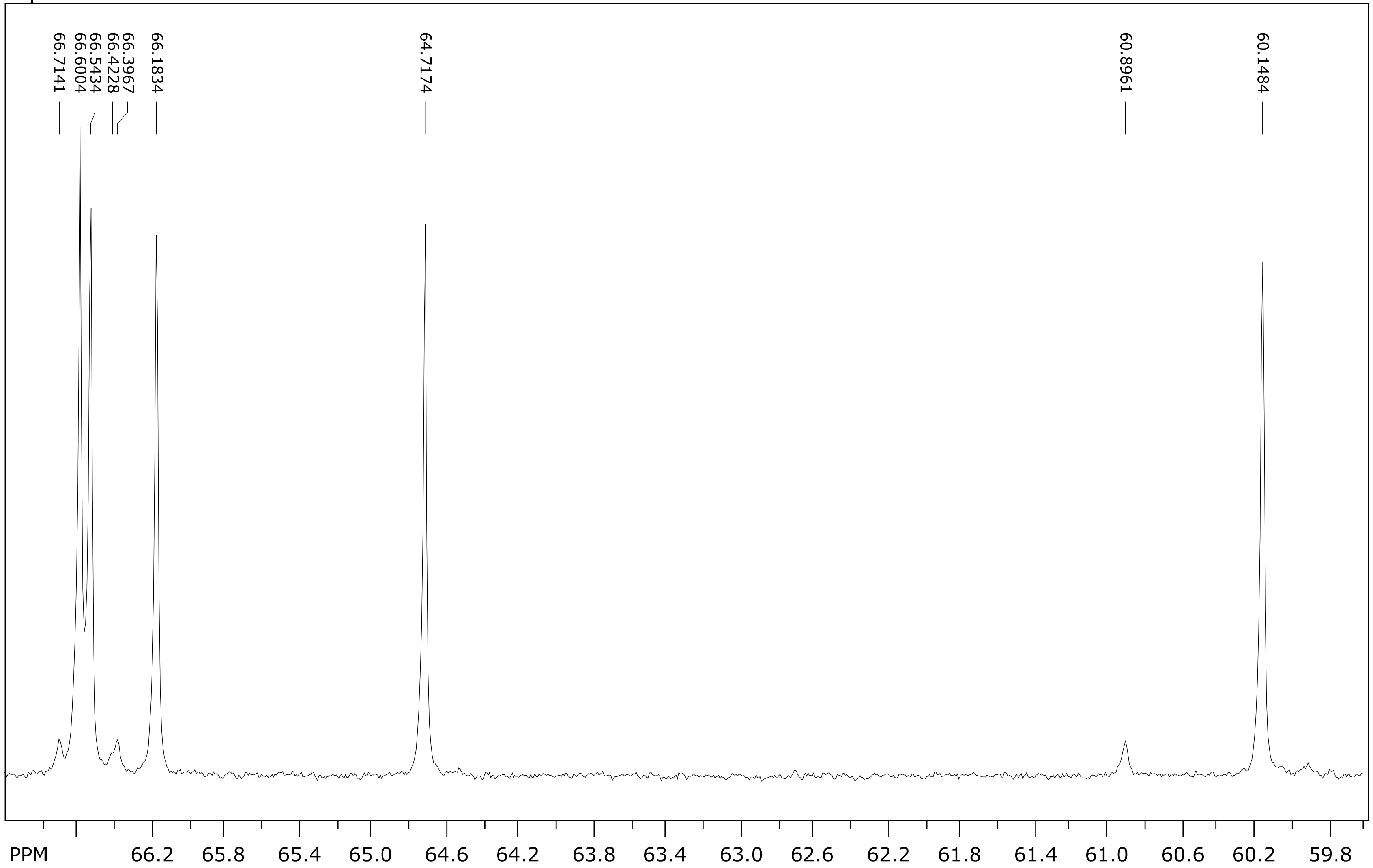

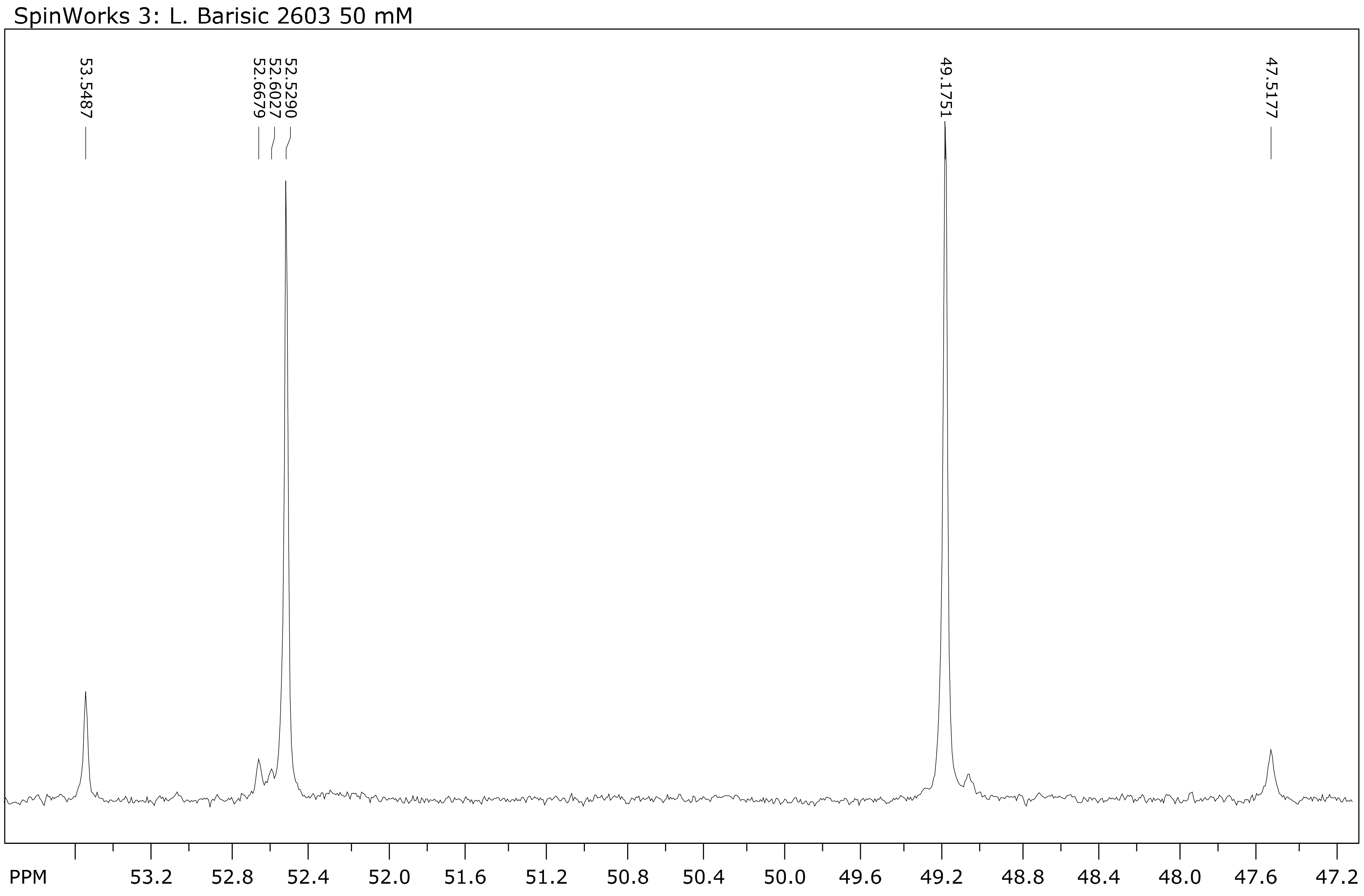
SpinWorks 3: L. Barisic 2603 50 mM
53.5487
52.6679
52.6027
52.5290
49.1751
47.5177
PPM
53.2
52.8
52.4
52.0
51.6
51.2
50.8
50.4
50.0
49.6
49.2
48.8
48.4
48.0
47.6
47.2

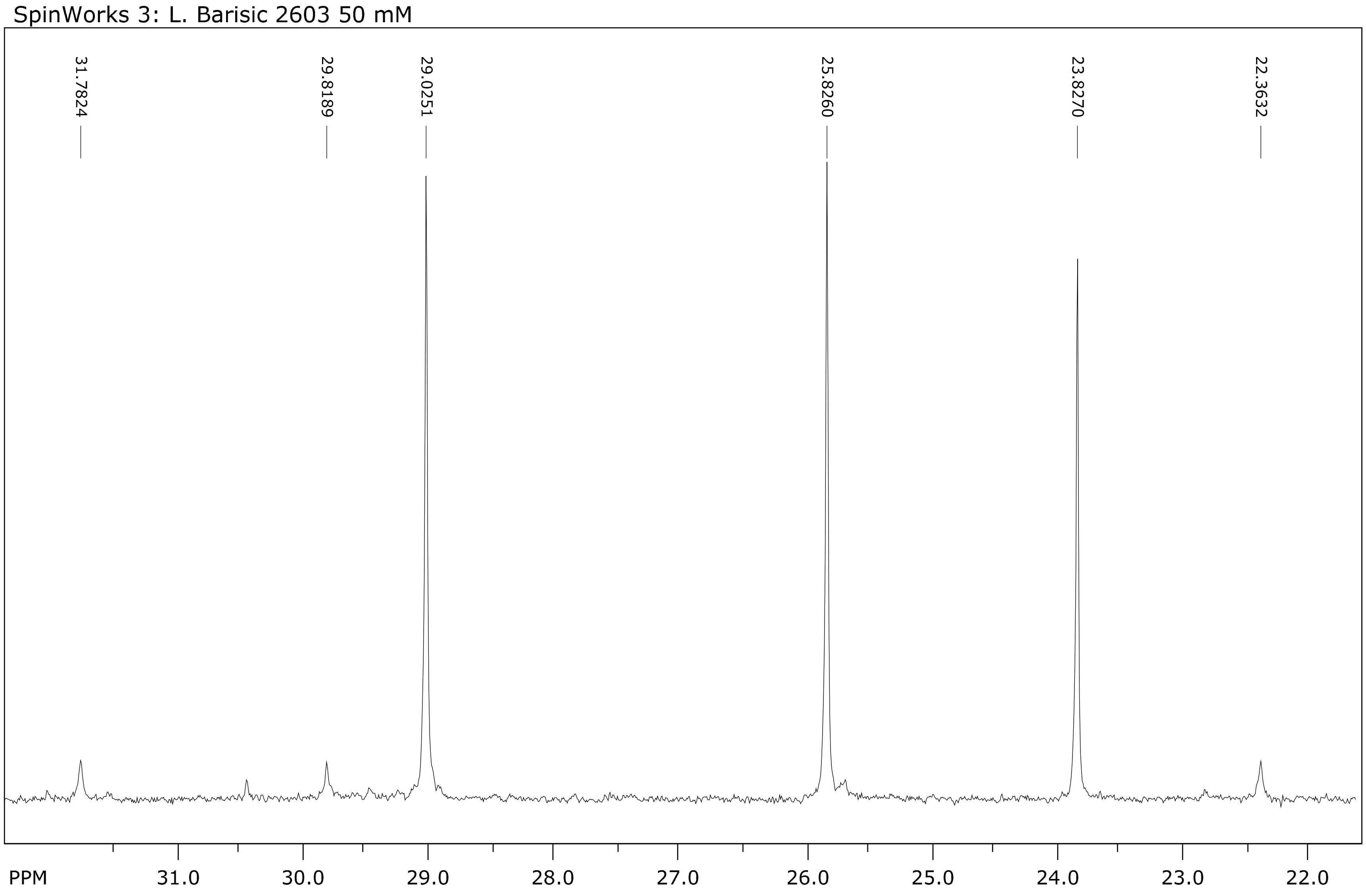
SpinWorks 3: L. Barisic 2603 50 mM
31.7824
29.8189
29.0251
25.8260
23.8270
22.3632
PPM
31.0
30.0
29.0
28.0
27.0
26.0
25.0
24.0
23.0
22.0

Figure S4. ^{1}H NMR titration of compound **1** with DMSO in $CDCl_3$.

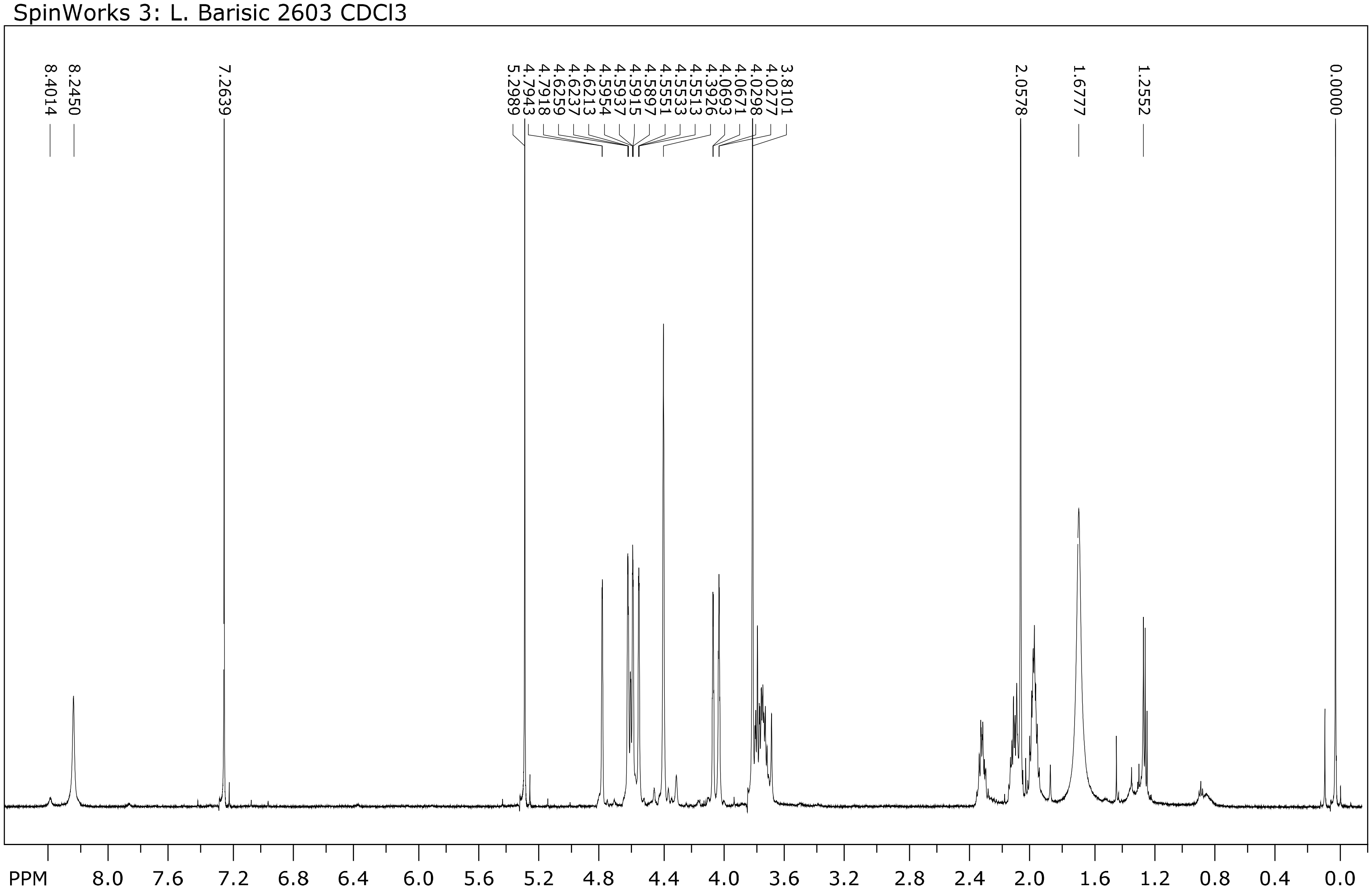

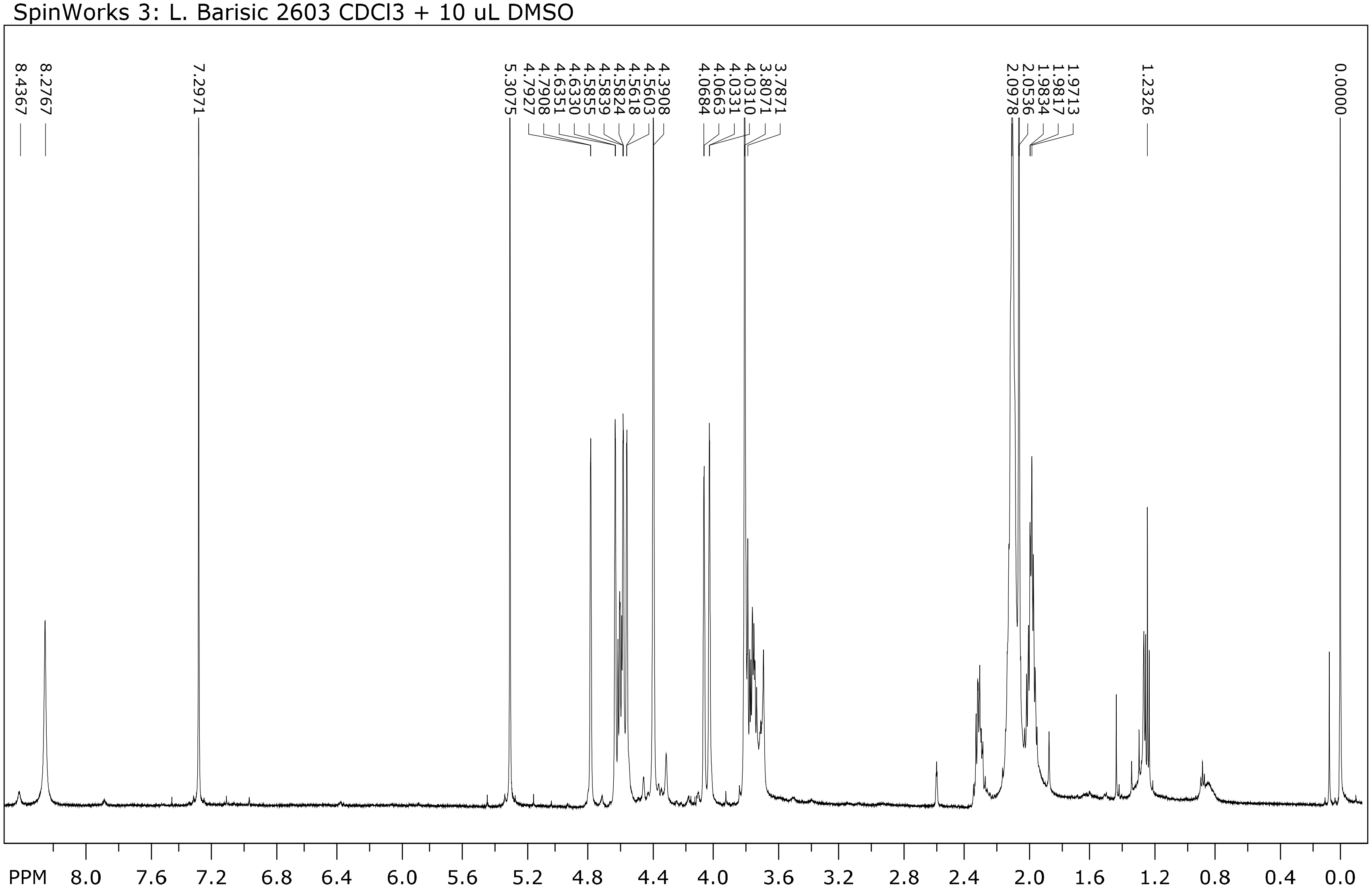
SpinWorks 3: L. Barisic 2603 CDCl3 + 10 uL DMSO
8.4367
8.2767
7.2971
5.3075
4.7927
4.7908
4.6351
4.6330
4.5855
4.5839
4.5824
4.5618
4.5603
4.3908
4.0684
4.0663
4.0331
4.0310
3.8071
3.7871
2.0978
2.0536
1.9834
1.9817
1.9713
1.2326
0.0000
PPM
8.0
7.6
7.2
6.8
6.4
6.0
5.6
5.2
4.8
4.4
4.0
3.6
3.2
2.8
2.4
2.0
1.6
1.2
0.8
0.4
0.0

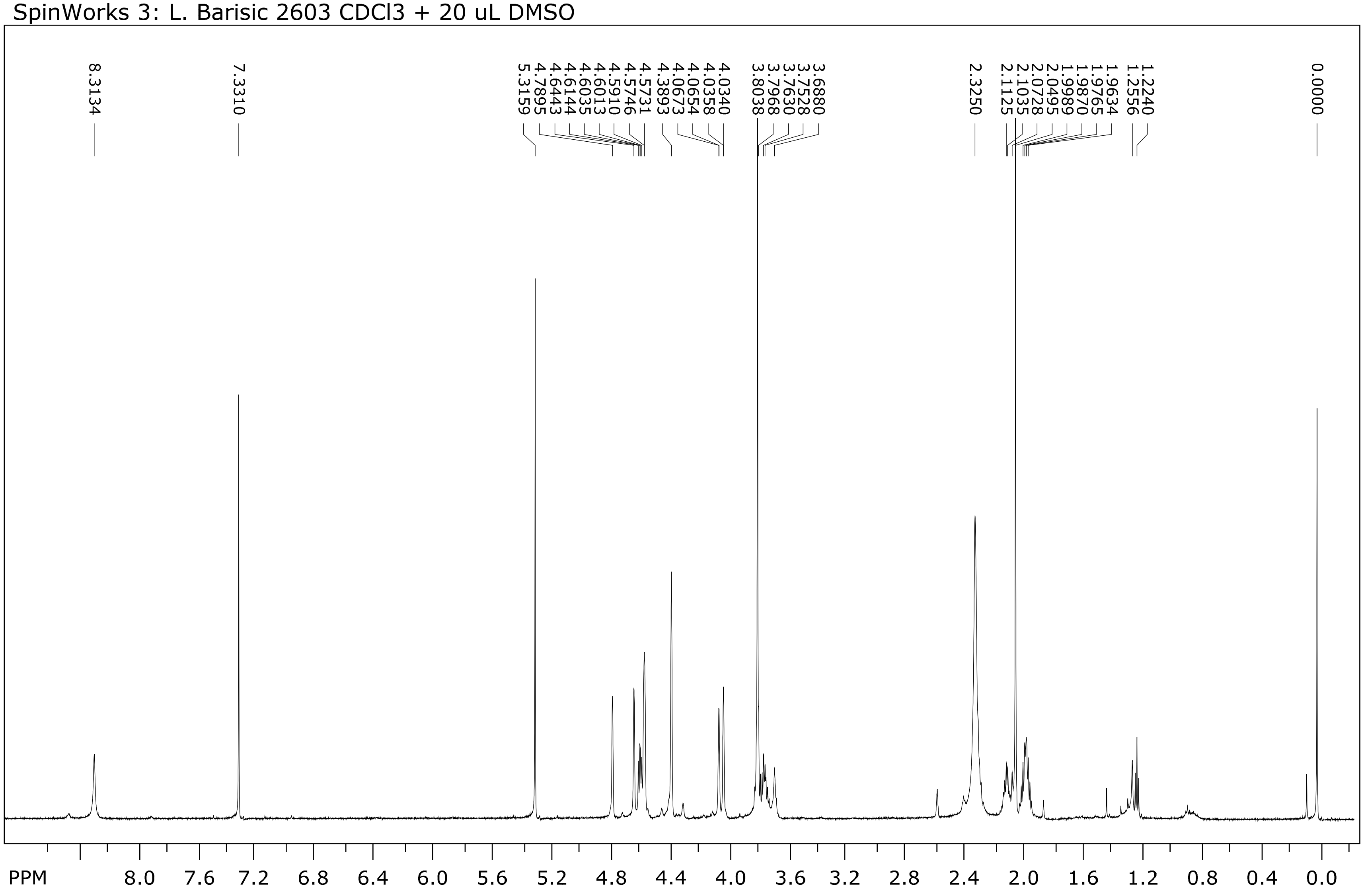

SpinWorks 3: L. Barisic 2603 CDCl3 + 20 uL DMSO
8.3134
7.3310
5.3159
4.7895
4.6443
4.6144
4.6035
4.6013
4.5910
4.5746
4.5731
4.3893
4.0673
4.0654
4.0358
4.0340
3.8038
3.7968
3.7630
3.7528
3.6880
2.3250
2.1125
2.1035
2.0728
2.0495
1.9989
1.9870
1.9765
1.9634
1.2556
1.2240
0.0000
PPM
8.0
7.6
7.2
6.8
6.4
6.0
5.6
5.2
4.8
4.4
4.0
3.6
3.2
2.8
2.4
2.0
1.6
1.2
0.8
0.4
0.0

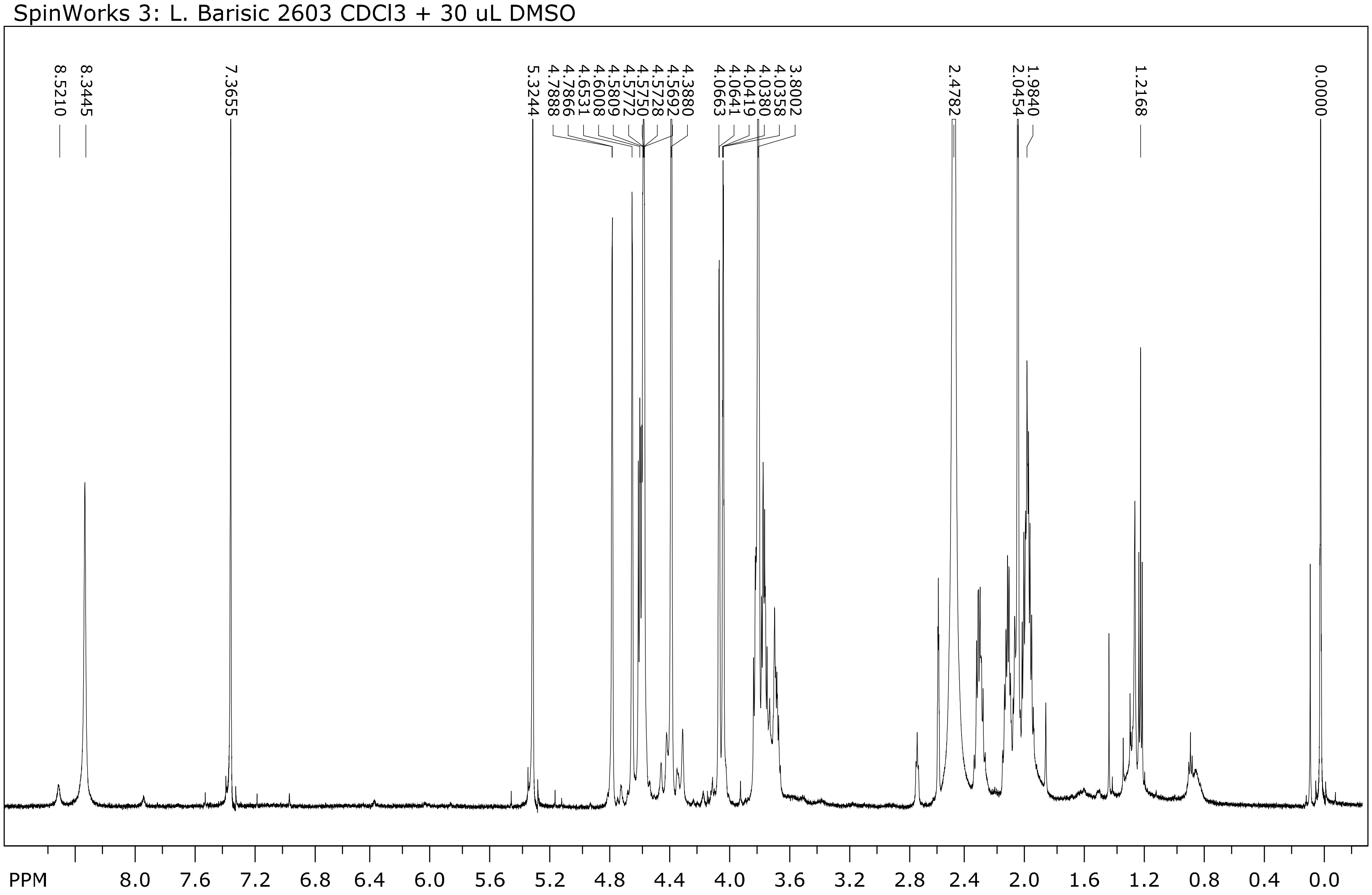
SpinWorks 3: L. Barisic 2603 CDCl3 + 30 uL DMSO
8.5210
8.3445
7.3655
5.3244
4.7888
4.7866
4.6531
4.6008
4.5809
4.5772
4.5750
4.5728
4.5692
4.3880
4.0663
4.0641
4.0419
4.0380
4.0358
3.8002
2.4782
2.0454
1.9840
1.2168
0.0000
PPM
8.0 7.6 7.2 6.8 6.4 6.0 5.6 5.2 4.8 4.4 4.0 3.6 3.2 2.8 2.4 2.0 1.6 1.2 0.8 0.4 0.0

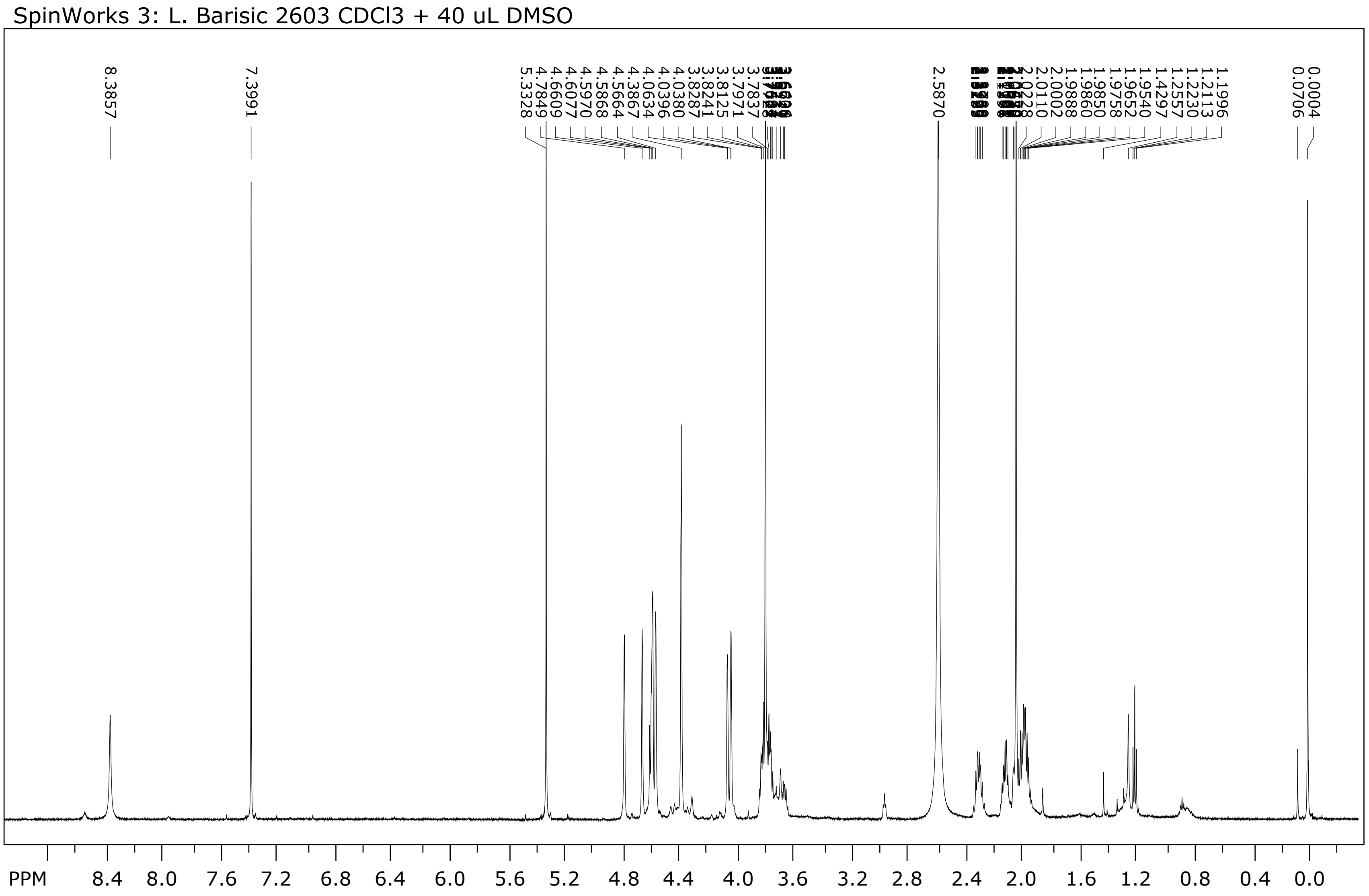
SpinWorks 3: L. Barisic 2603 CDCl3 + 40 uL DMSO
PPM
8.4
8.0
7.6
7.2
6.8
6.4
6.0
5.6
5.2
4.8
4.4
4.0
3.6
3.2
2.8
2.4
2.0
1.6
1.2
0.8
0.4
0.0

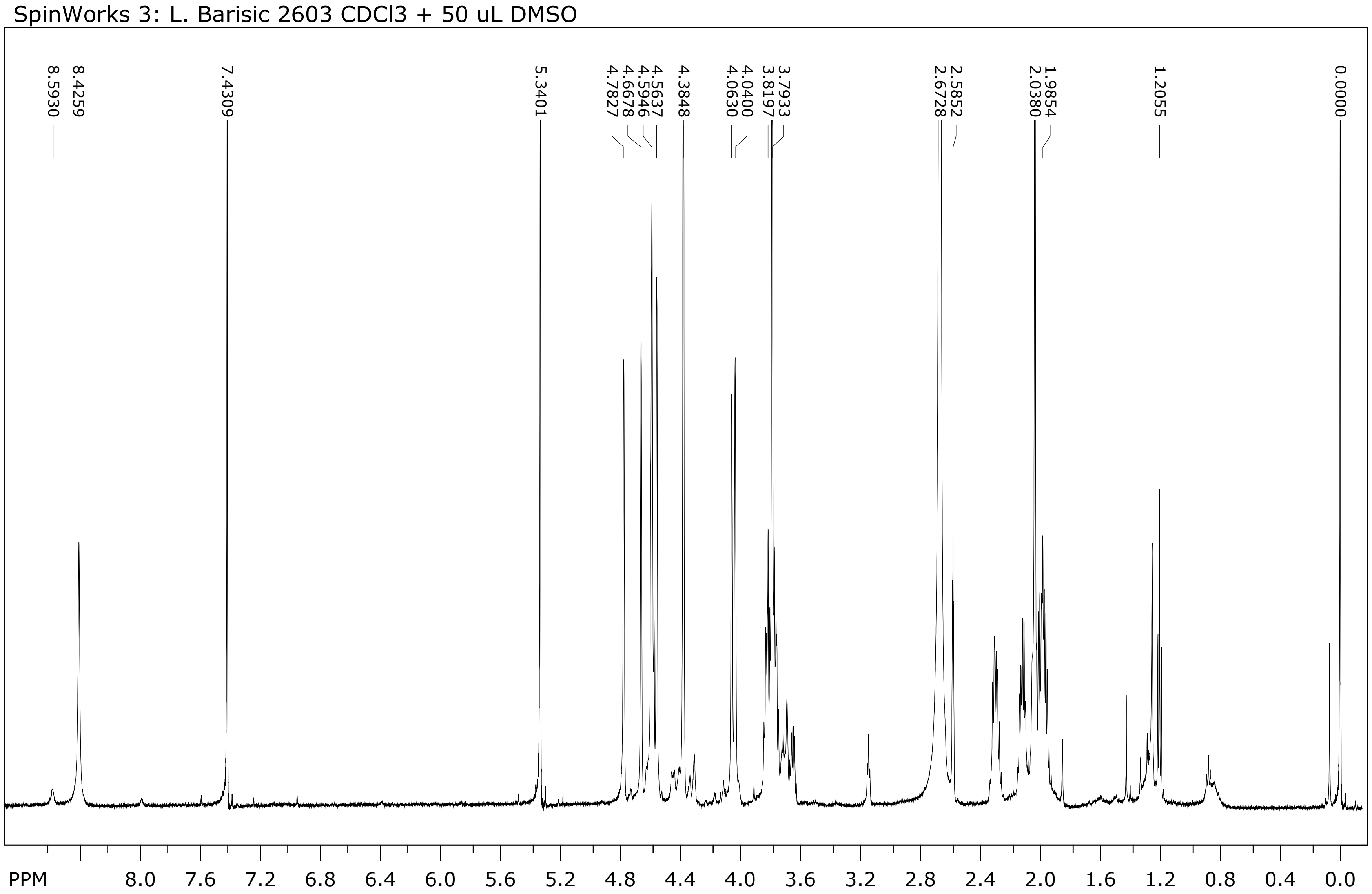
SpinWorks 3: L. Barisic 2603 CDCl3 + 50 uL DMSO
8.5930
8.4259
7.4309
5.3401
4.7827
4.6678
4.5946
4.5637
4.3848
4.0630
4.0400
3.8197
3.7933
2.6728
2.5852
2.0380
1.9854
1.2055
0.0000
PPM
8.0 7.6 7.2 6.8 6.4 6.0 5.6 5.2 4.8 4.4 4.0 3.6 3.2 2.8 2.4 2.0 1.6 1.2 0.8 0.4 0.0

SpinWorks 3: L. Barisic 2603 CDCl3 + 70 uL DMSO

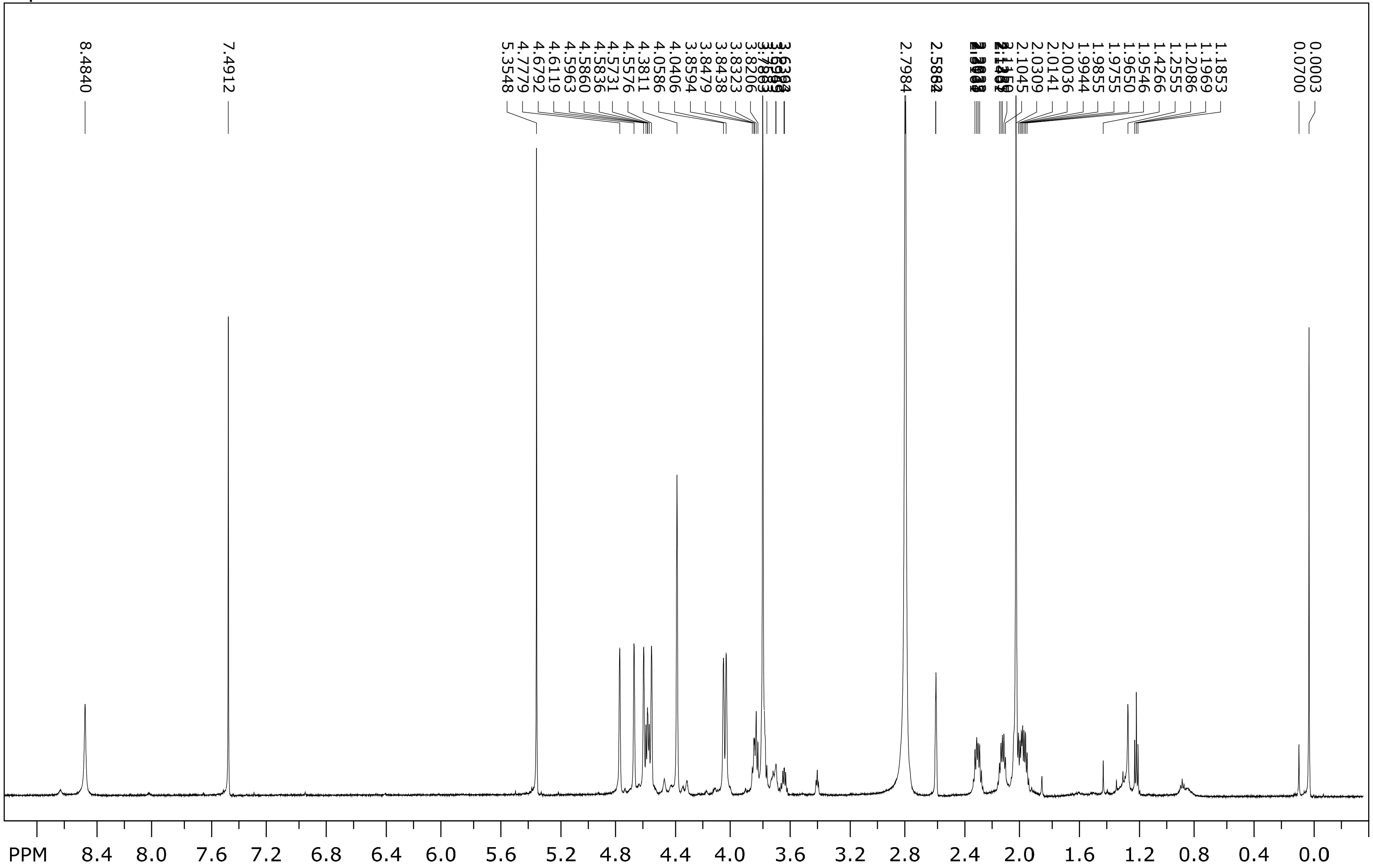

SpinWorks 3: L. Barisic 2603 CDCl3 + 90 uL DMSO

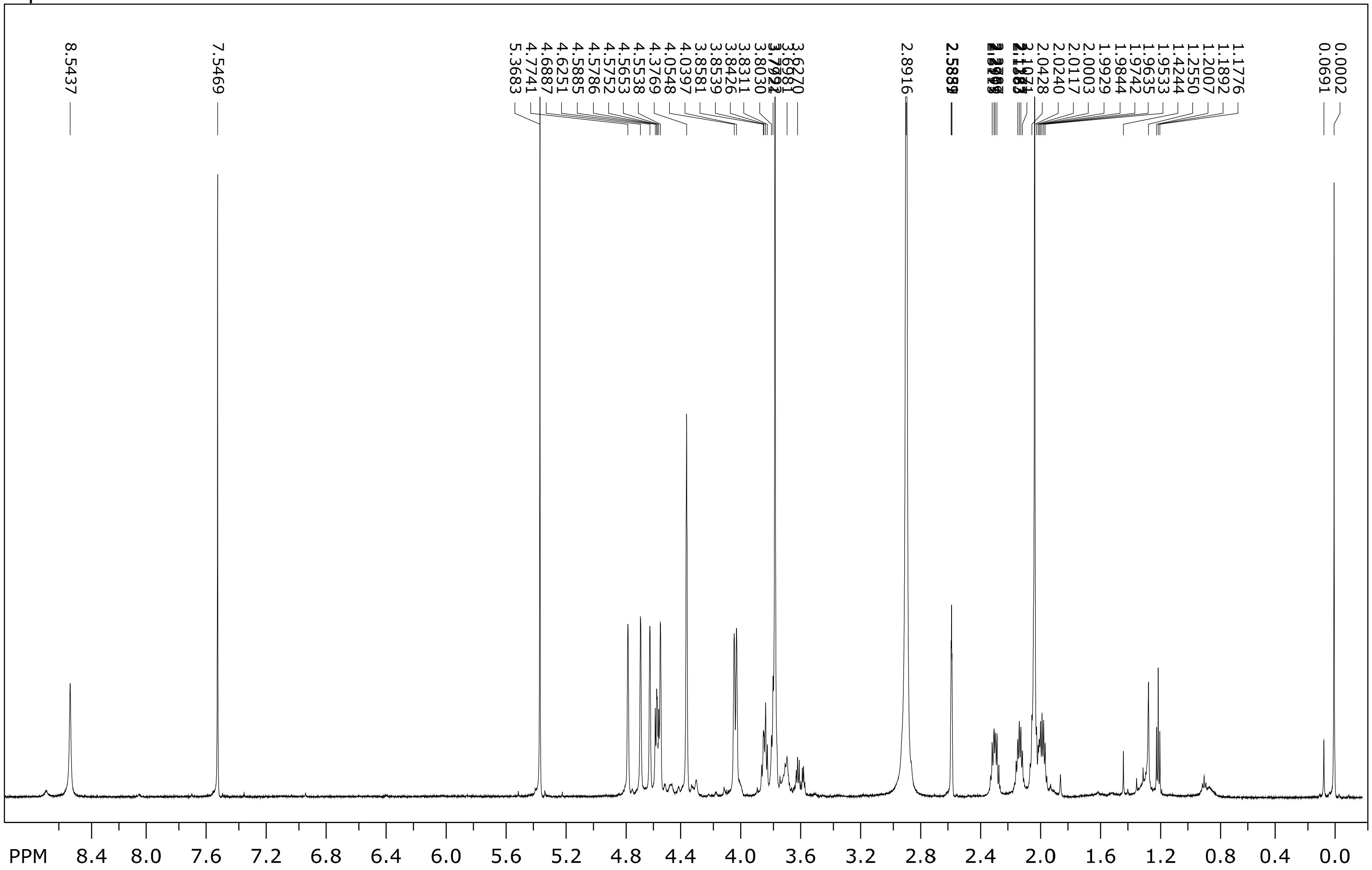

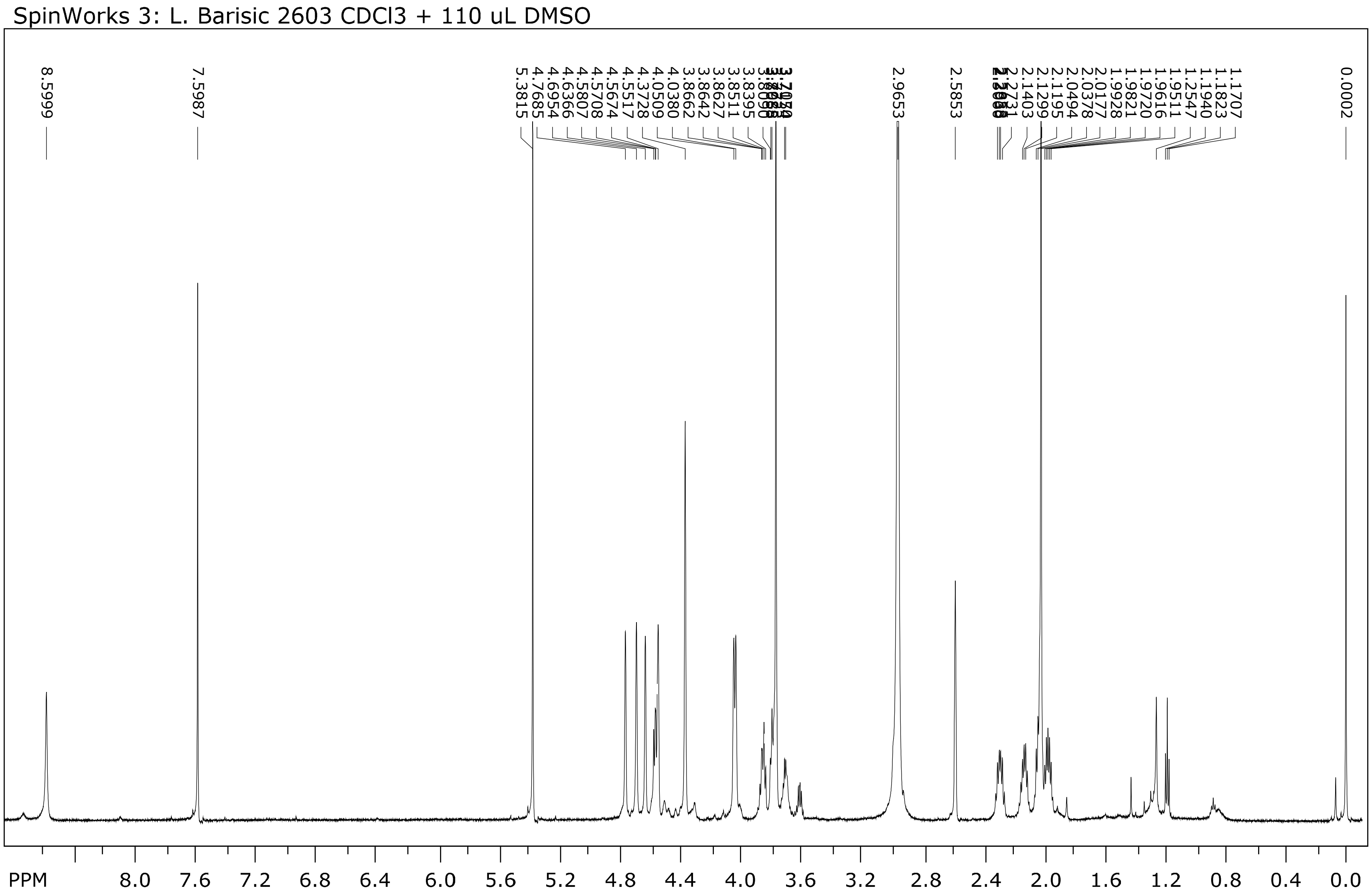
SpinWorks 3: L. Barisic 2603 CDCl3 + 110 uL DMSO
PPM
8.0 7.6 7.2 6.8 6.4 6.0 5.6 5.2 4.8 4.4 4.0 3.6 3.2 2.8 2.4 2.0 1.6 1.2 0.8 0.4 0.0

SpinWorks 3: L. Barisic 2603 CDCl3 + 130 uL DMSO

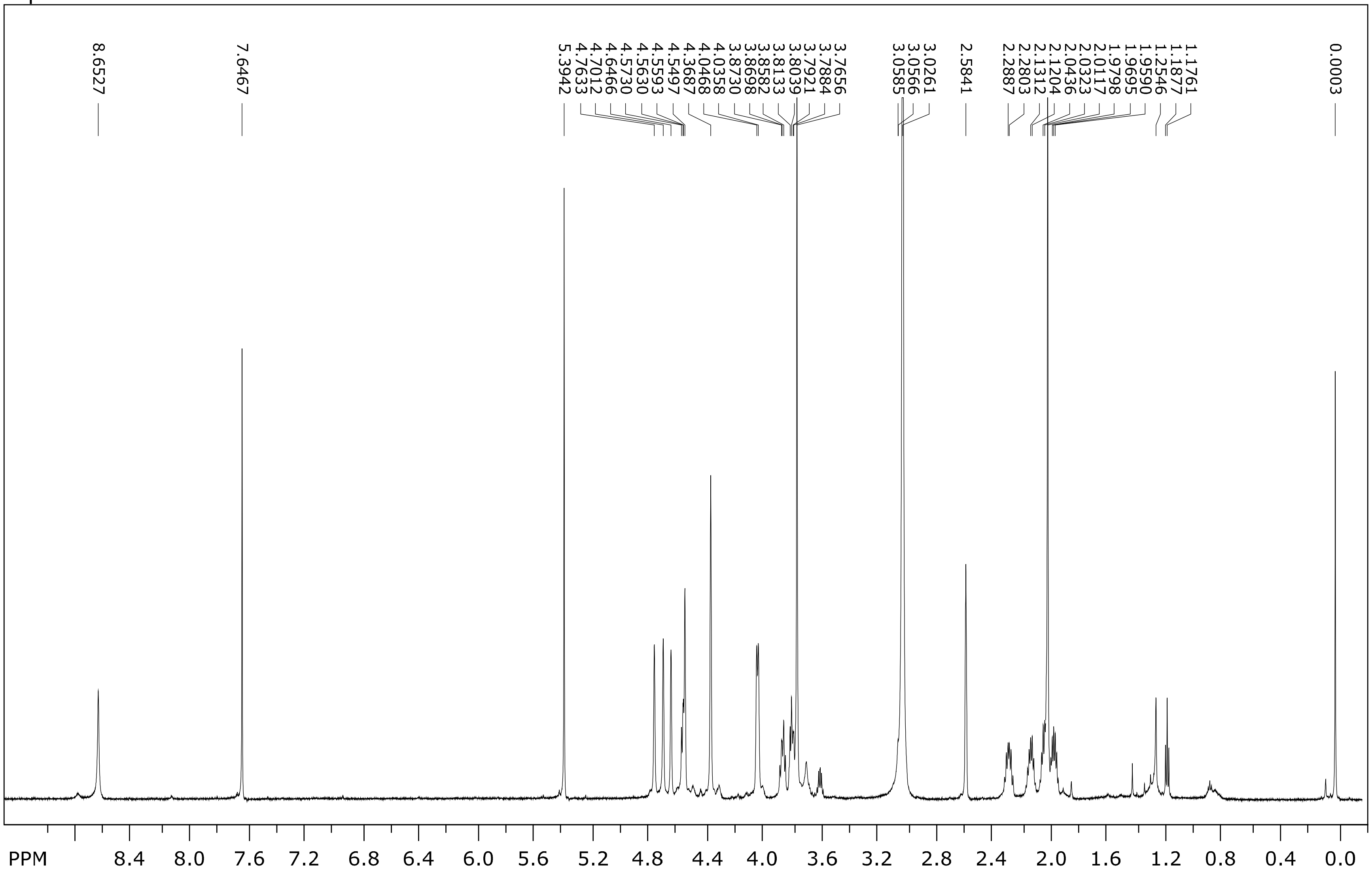

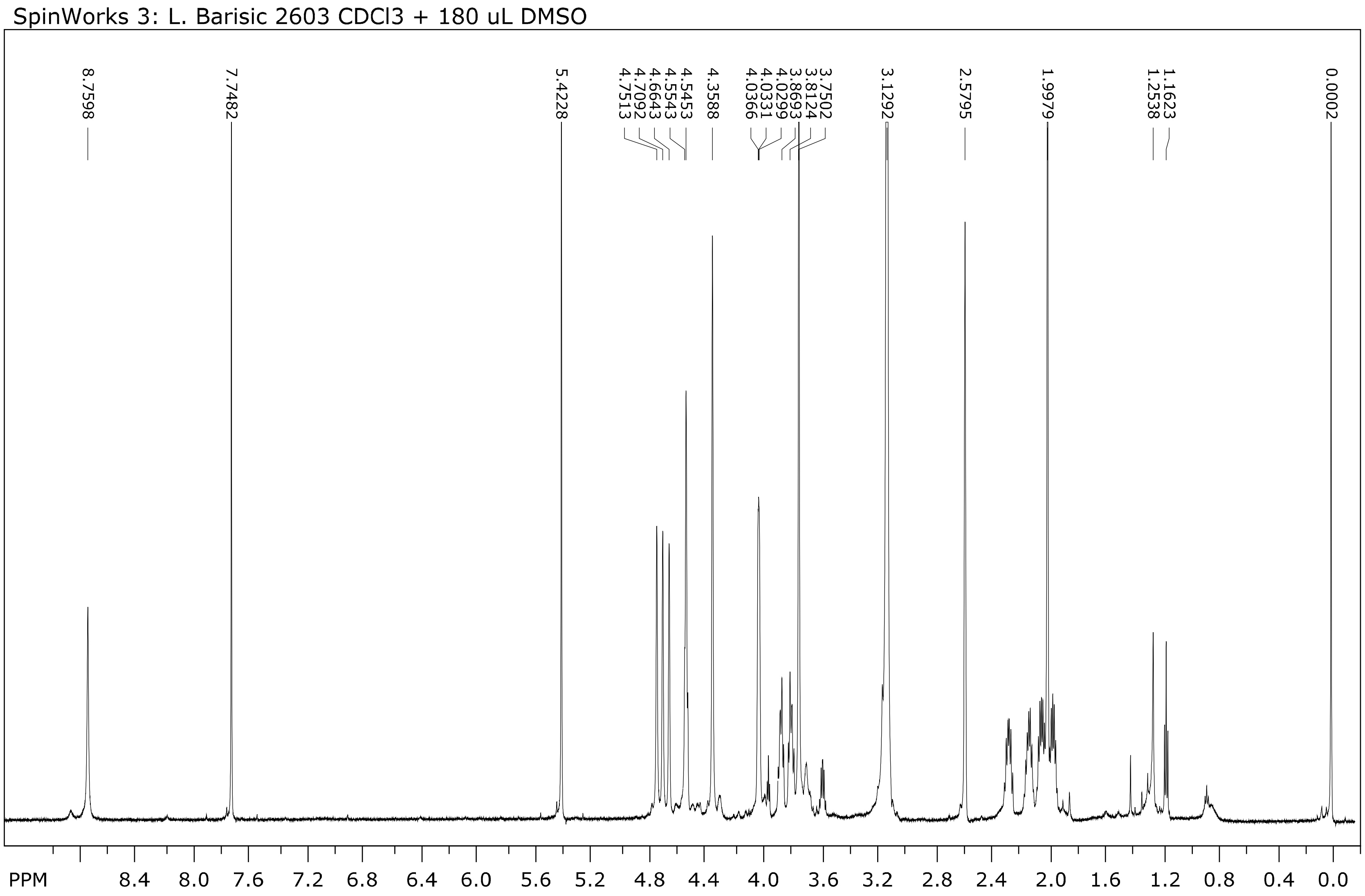
SpinWorks 3: L. Barisic 2603 CDCl3 + 180 uL DMSO
8.7598
7.7482
5.4228
4.7513
4.7092
4.6643
4.5543
4.5453
4.3588
4.0366
4.0331
4.0299
3.8693
3.8124
3.7502
3.1292
2.5795
1.9979
1.2538
1.1623
0.0002
PPM
8.4
8.0
7.6
7.2
6.8
6.4
6.0
5.6
5.2
4.8
4.4
4.0
3.6
3.2
2.8
2.4
2.0
1.6
1.2
0.8
0.4
0.0

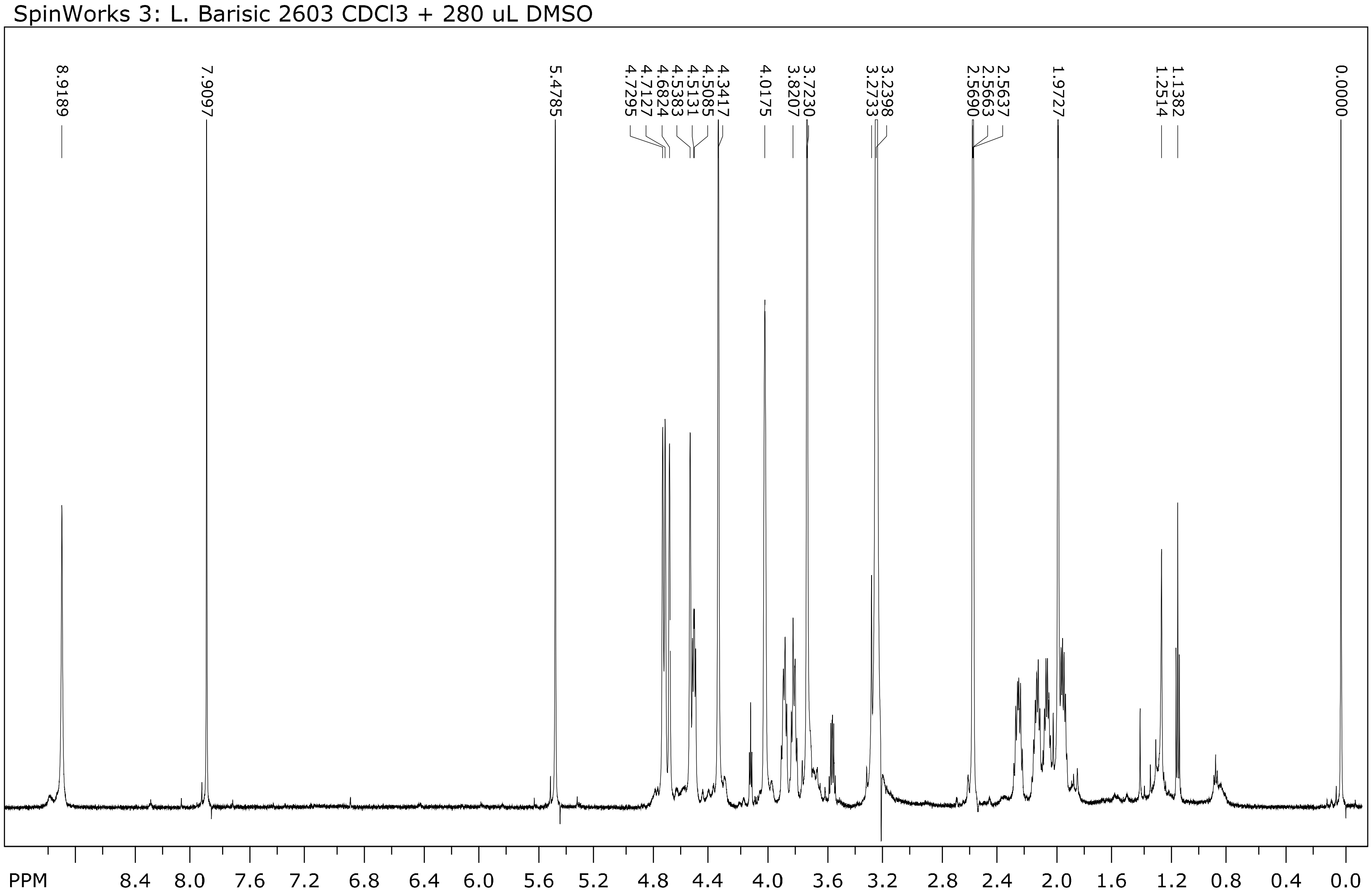

SpinWorks 3: L. Barisic 2603 CDCl3 + 280 uL DMSO
8.9189
7.9097
5.4785
4.7295
4.7127
4.6824
4.5383
4.5131
4.5085
4.3417
4.0175
3.8207
3.7230
3.2733
3.2398
2.5690
2.5663
2.5637
1.9727
1.2514
1.1382
0.0000
PPM
8.4 8.0 7.6 7.2 6.8 6.4 6.0 5.6 5.2 4.8 4.4 4.0 3.6 3.2 2.8 2.4 2.0 1.6 1.2 0.8 0.4 0.0

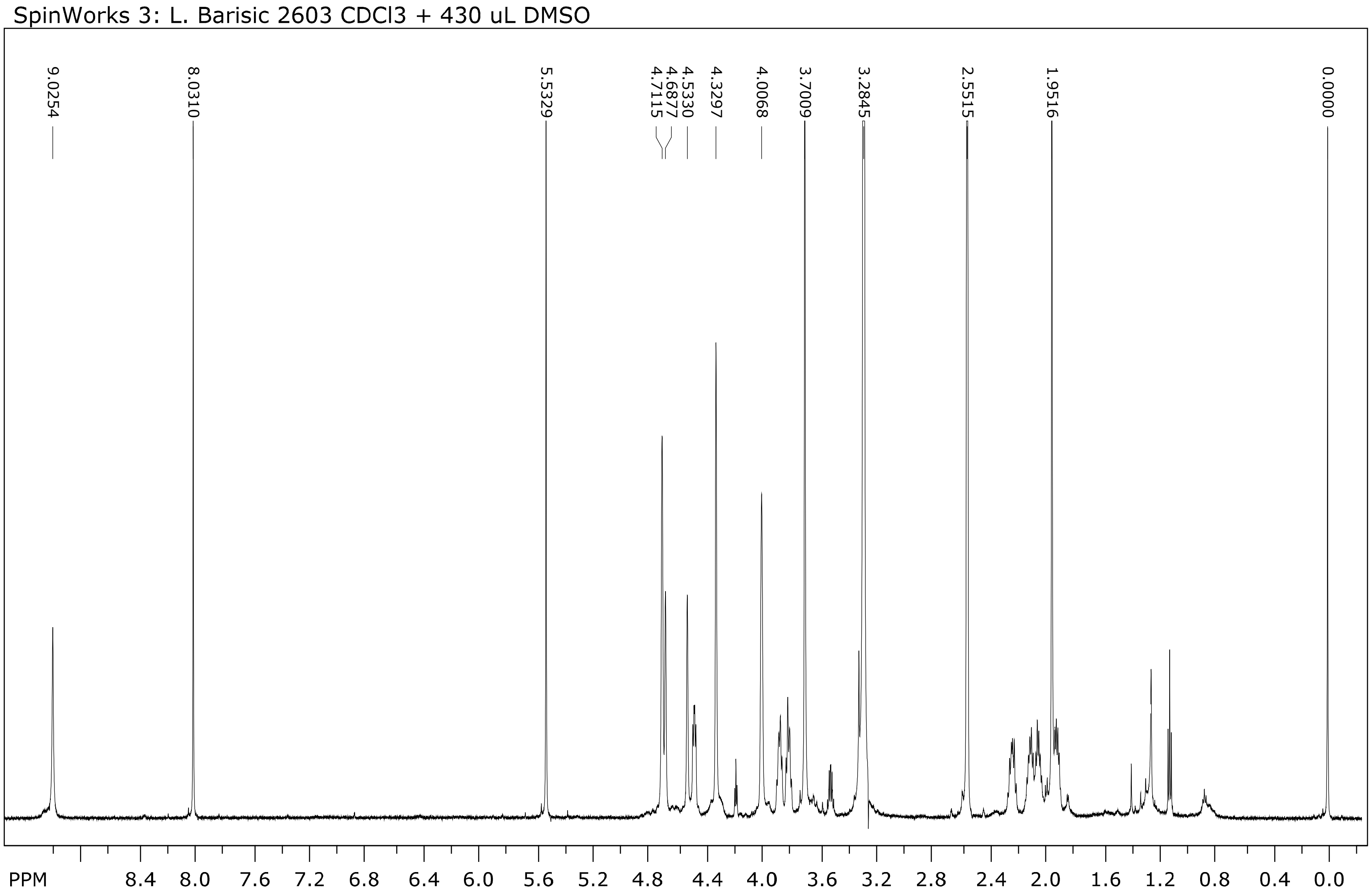
SpinWorks 3: L. Barisic 2603 CDCl3 + 430 uL DMSO
9.0254
8.0310
5.5329
4.7115
4.6877
4.5330
4.3297
4.0068
3.7009
3.2845
2.5515
1.9516
0.0000
PPM
8.4 8.0 7.6 7.2 6.8 6.4 6.0 5.6 5.2 4.8 4.4 4.0 3.6 3.2 2.8 2.4 2.0 1.6 1.2 0.8 0.4 0.0

SpinWorks 3: L. Barisic 2603 CDCl3 + 630 uL DMSO

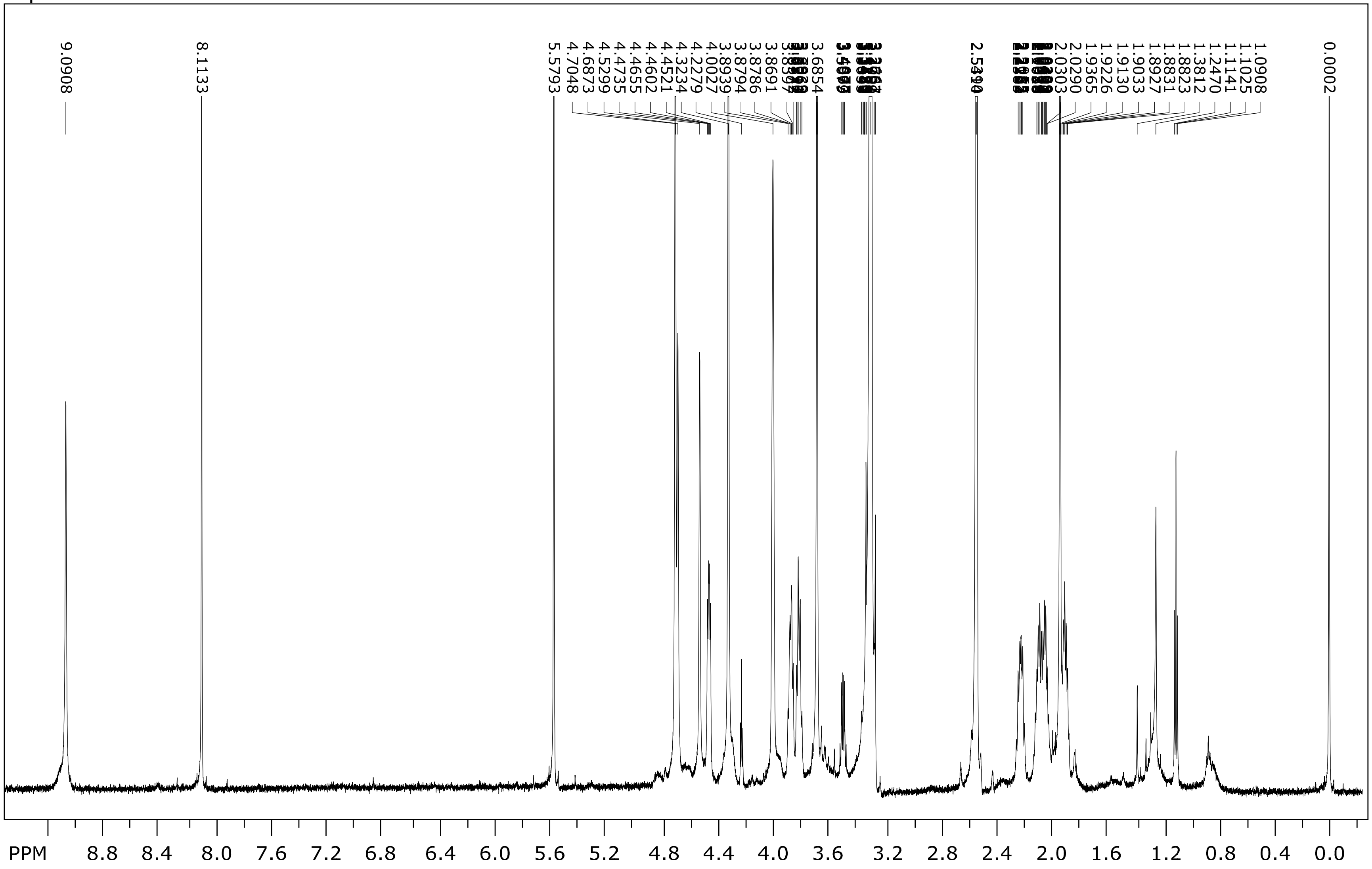

Figure S5. Variable-temperature ^{1}H NMR spectra of **1**.

SpinWorks 3: L. Barisic 2603 55 �C

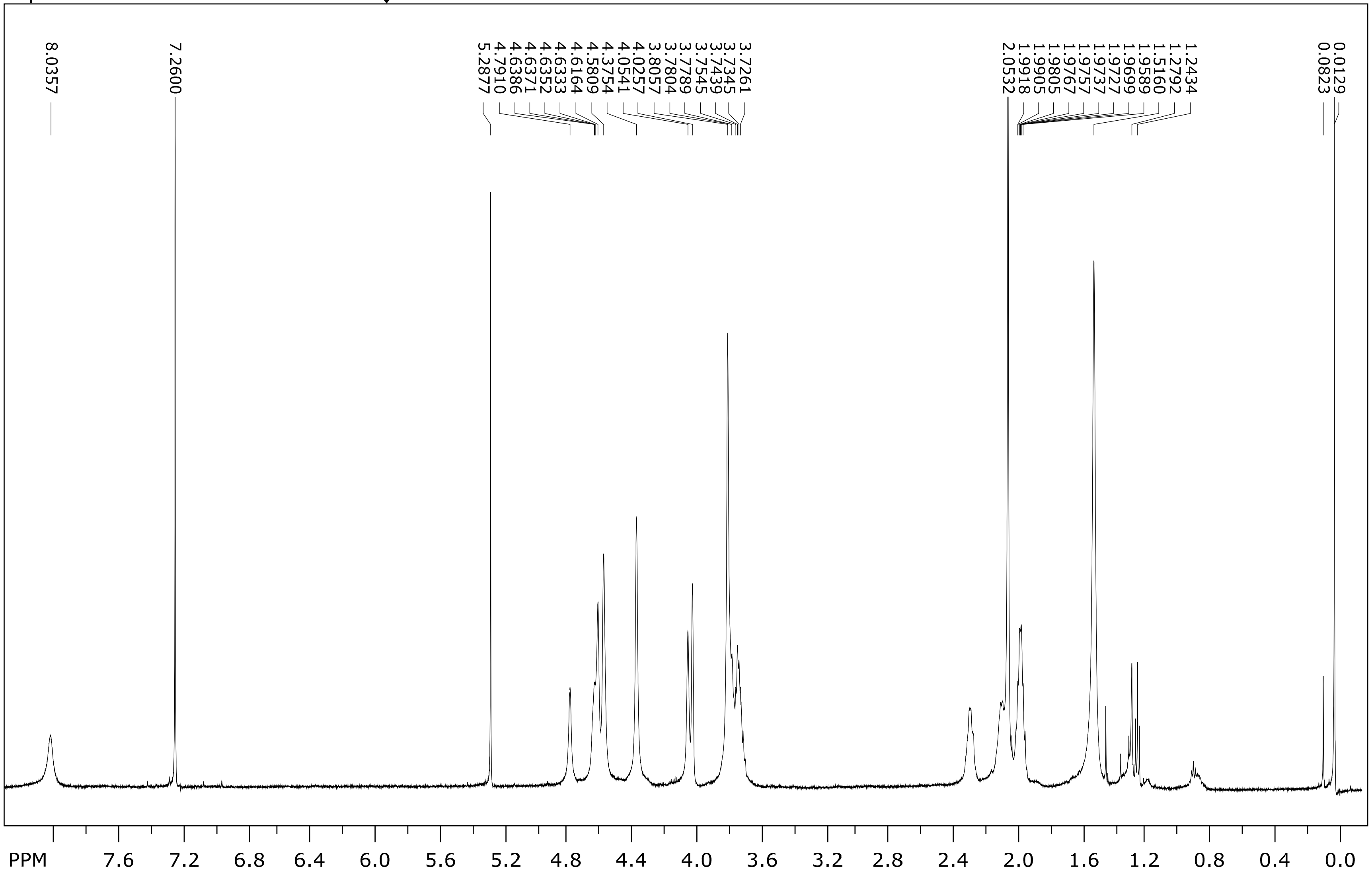

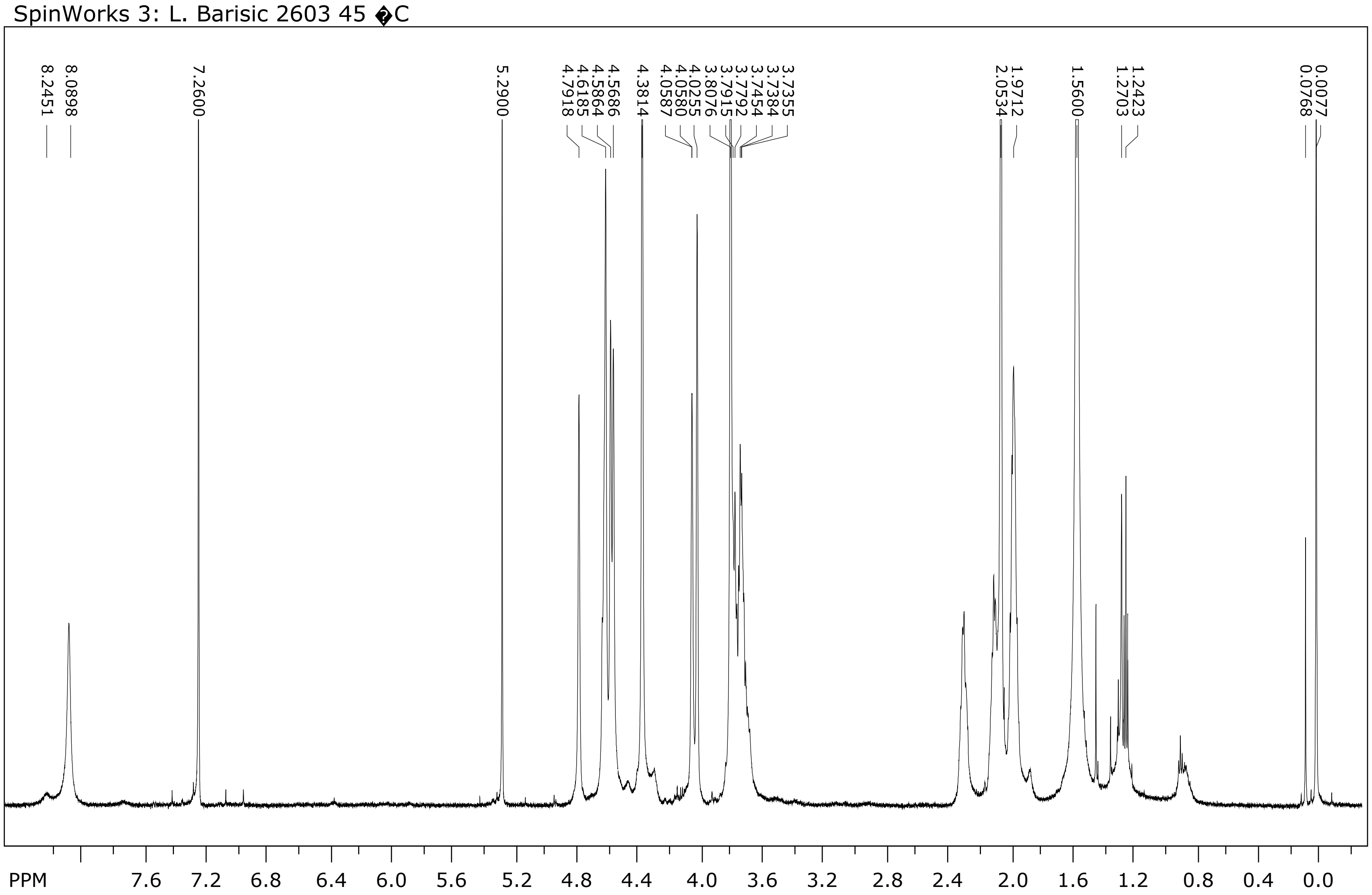
SpinWorks 3: L. Barisic 2603 45 �C
8.2451
8.0898
7.2600
5.2900
4.7918
4.6185
4.5864
4.5686
4.3814
4.0587
4.0580
4.0255
3.8076
3.7915
3.7792
3.7454
3.7384
3.7355
2.0534
1.9712
1.5600
1.2703
1.2423
0.0768
0.0077
PPM
7.6 7.2 6.8 6.4 6.0 5.6 5.2 4.8 4.4 4.0 3.6 3.2 2.8 2.4 2.0 1.6 1.2 0.8 0.4 0.0

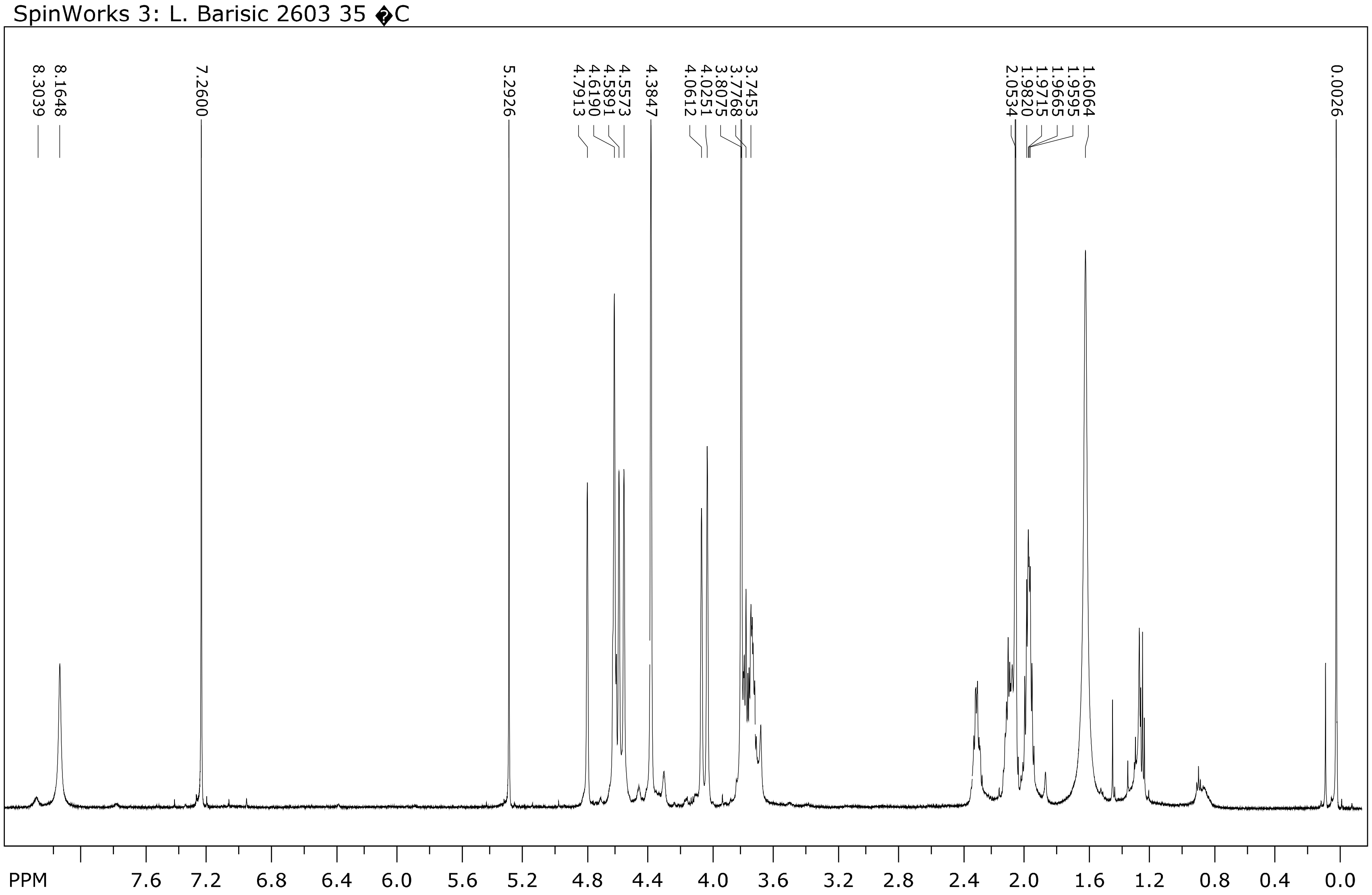
SpinWorks 3: L. Barisic 2603 35 �C
8.3039
8.1648
7.2600
5.2926
4.7913
4.6190
4.5891
4.5573
4.3847
4.0612
4.0251
3.8075
3.7768
3.7453
2.0534
1.9820
1.9715
1.9665
1.9595
1.6064
0.0026
PPM
7.6 7.2 6.8 6.4 6.0 5.6 5.2 4.8 4.4 4.0 3.6 3.2 2.8 2.4 2.0 1.6 1.2 0.8 0.4 0.0

SpinWorks 3: L. Barisic 2603 25 �C

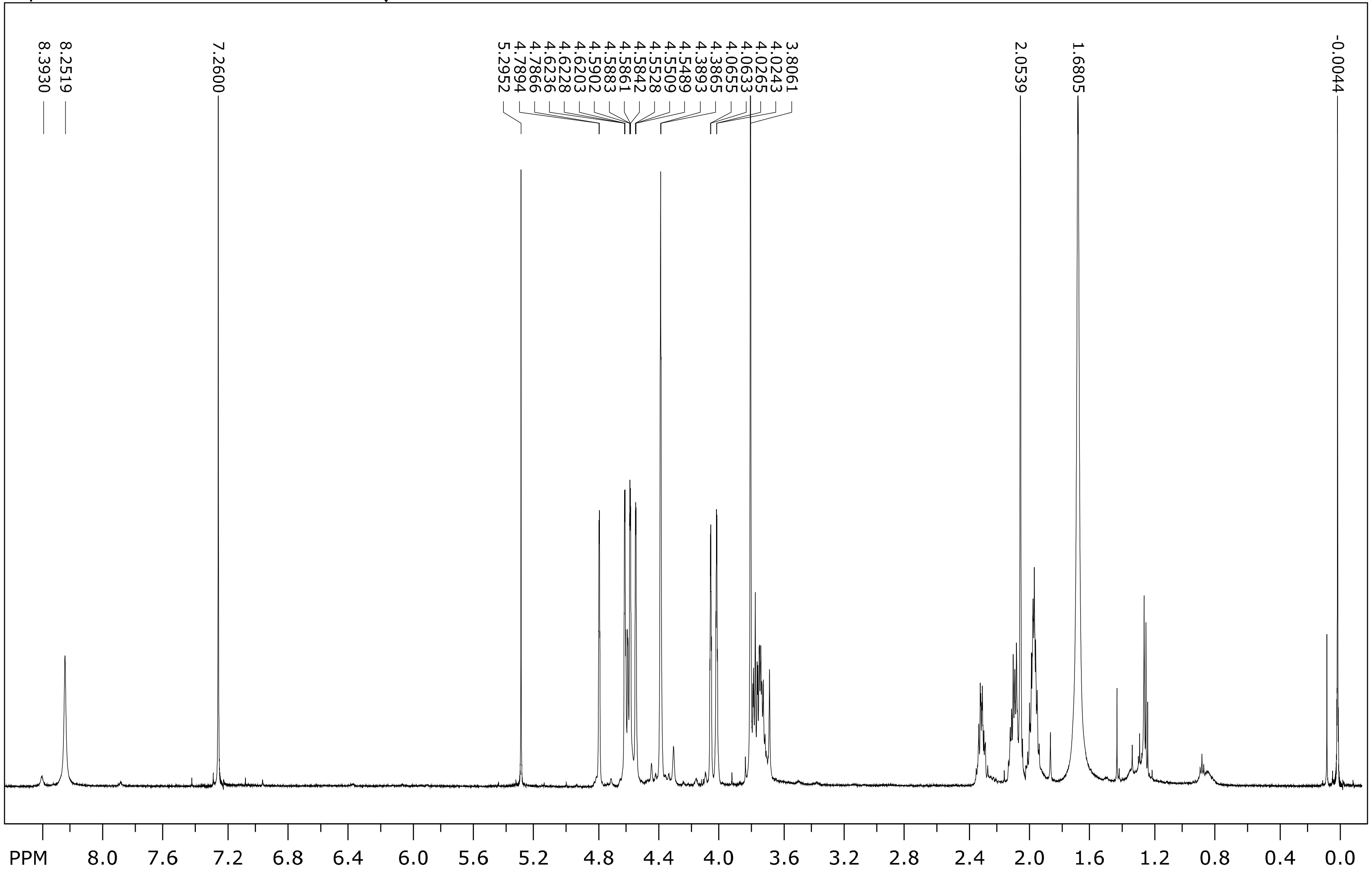

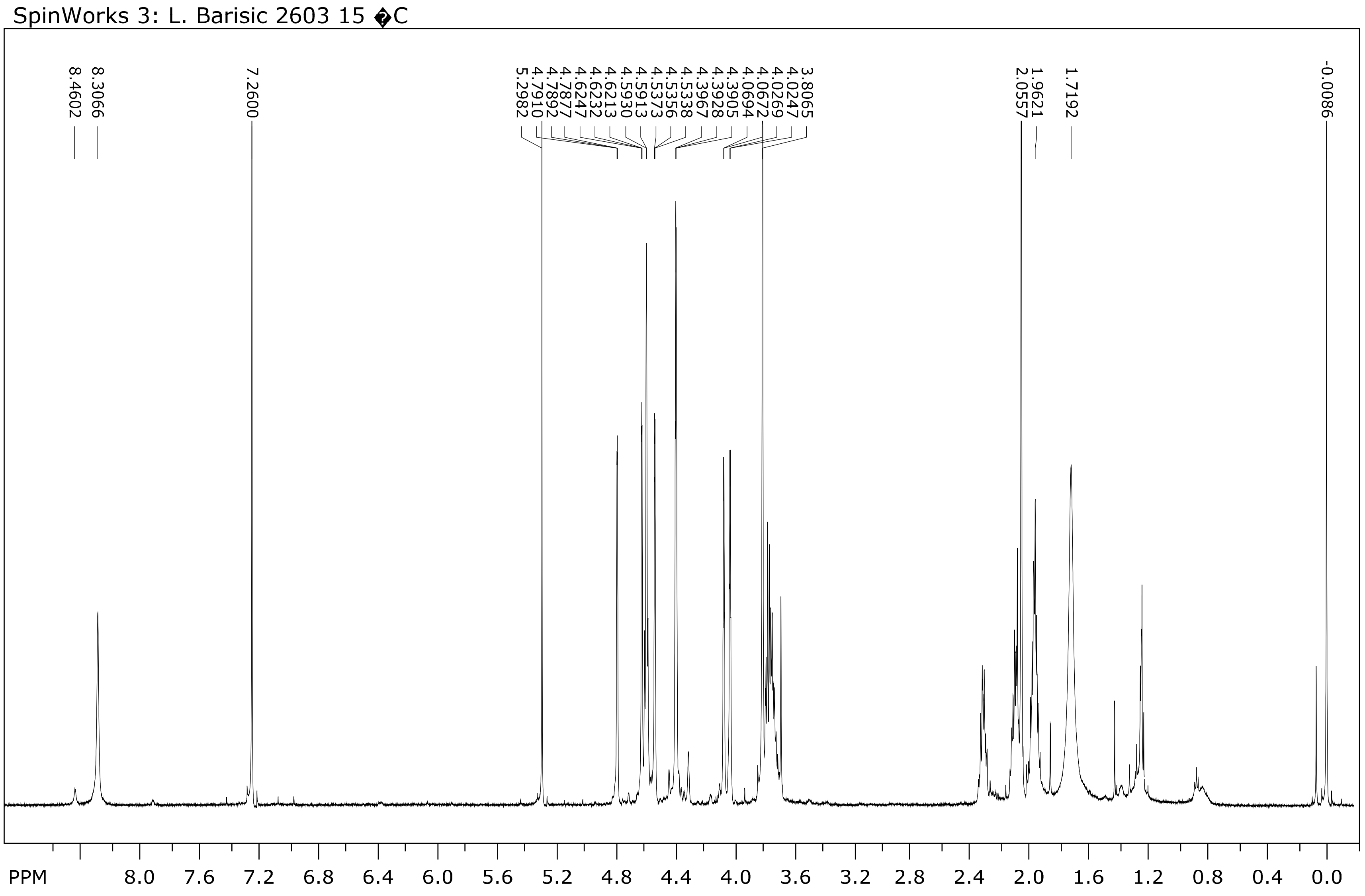
SpinWorks 3: L. Barisic 2603 15 �C
8.4602
8.3066
7.2600
5.2982
4.7910
4.7892
4.7877
4.6247
4.6232
4.6213
4.5930
4.5913
4.5373
4.5356
4.5338
4.3967
4.3928
4.3905
4.0694
4.0672
4.0269
4.0247
3.8065
2.0557
1.9621
1.7192
-0.0086
PPM
8.0 7.6 7.2 6.8 6.4 6.0 5.6 5.2 4.8 4.4 4.0 3.6 3.2 2.8 2.4 2.0 1.6 1.2 0.8 0.4 0.0

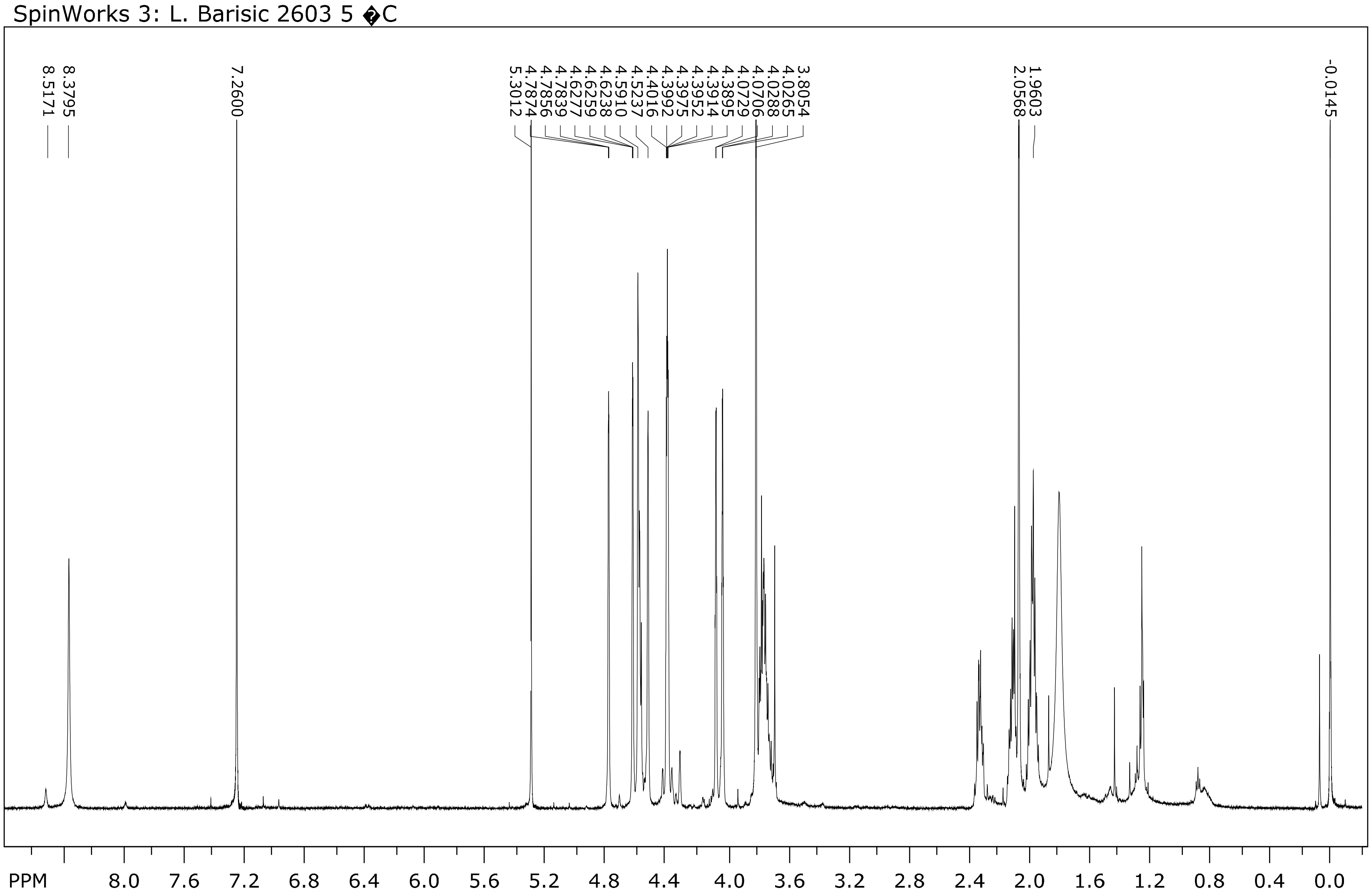
SpinWorks 3: L. Barisic 2603 5 �C
8.5171
8.3795
7.2600
5.3012
4.7874
4.7856
4.7839
4.6277
4.6259
4.6238
4.5910
4.5237
4.4016
4.3992
4.3975
4.3952
4.3914
4.3895
4.0729
4.0706
4.0288
4.0265
3.8054
2.0568
1.9603
-0.0145
PPM
8.0
7.6
7.2
6.8
6.4
6.0
5.6
5.2
4.8
4.4
4.0
3.6
3.2
2.8
2.4
2.0
1.6
1.2
0.8
0.4
0.0

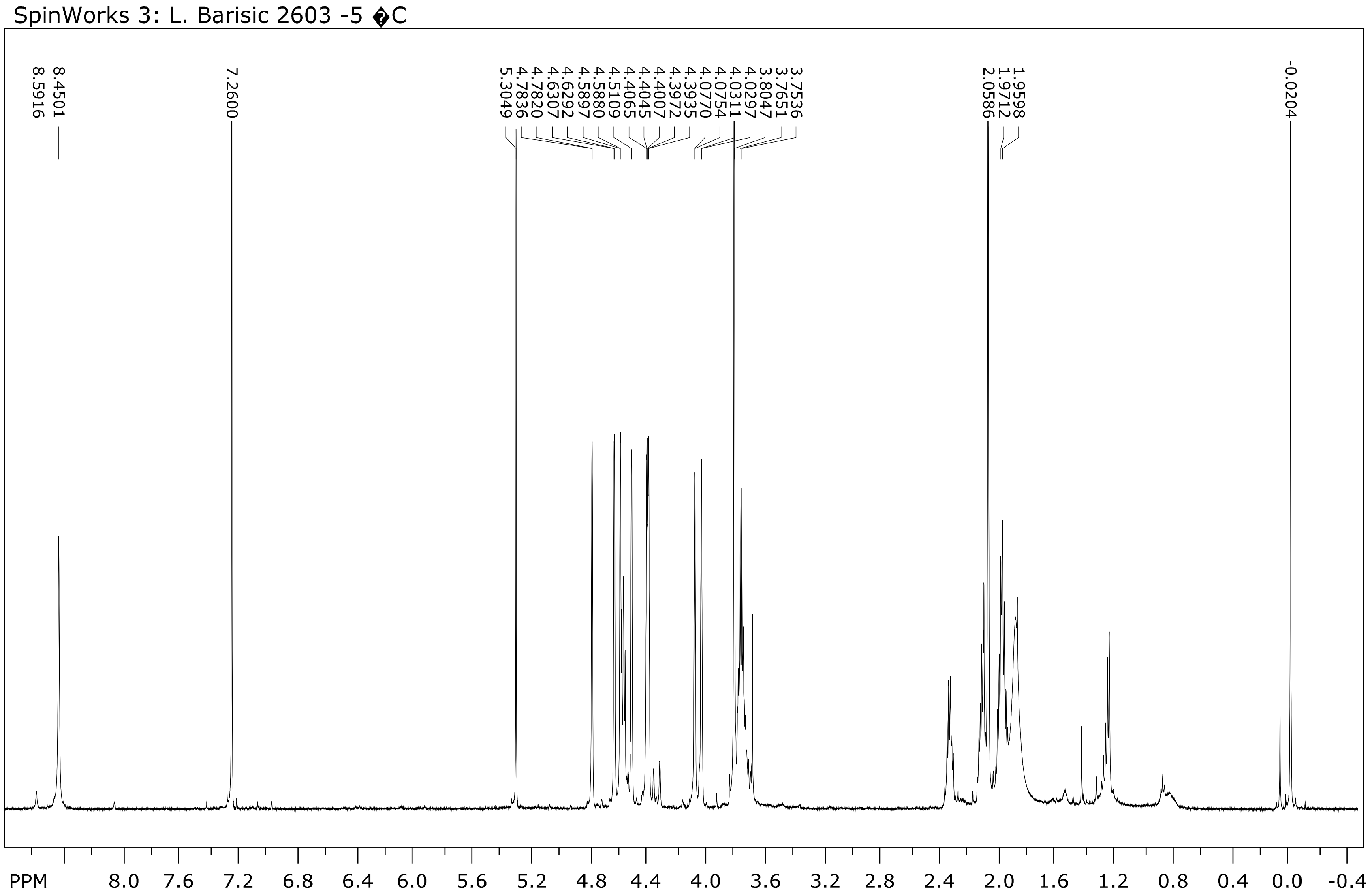
SpinWorks 3: L. Barisic 2603 -5 �C
8.5916
8.4501
7.2600
5.3049
4.7836
4.7820
4.6307
4.6292
4.5897
4.5880
4.5109
4.4065
4.4045
4.4007
4.3972
4.3935
4.0770
4.0754
4.0311
4.0297
3.8047
3.7651
3.7536
2.0586
1.9712
1.9598
-0.0204
PPM
8.0 7.6 7.2 6.8 6.4 6.0 5.6 5.2 4.8 4.4 4.0 3.6 3.2 2.8 2.4 2.0 1.6 1.2 0.8 0.4 0.0 -0.4

SpinWorks 3: L. Barisic 2603 -15 �C

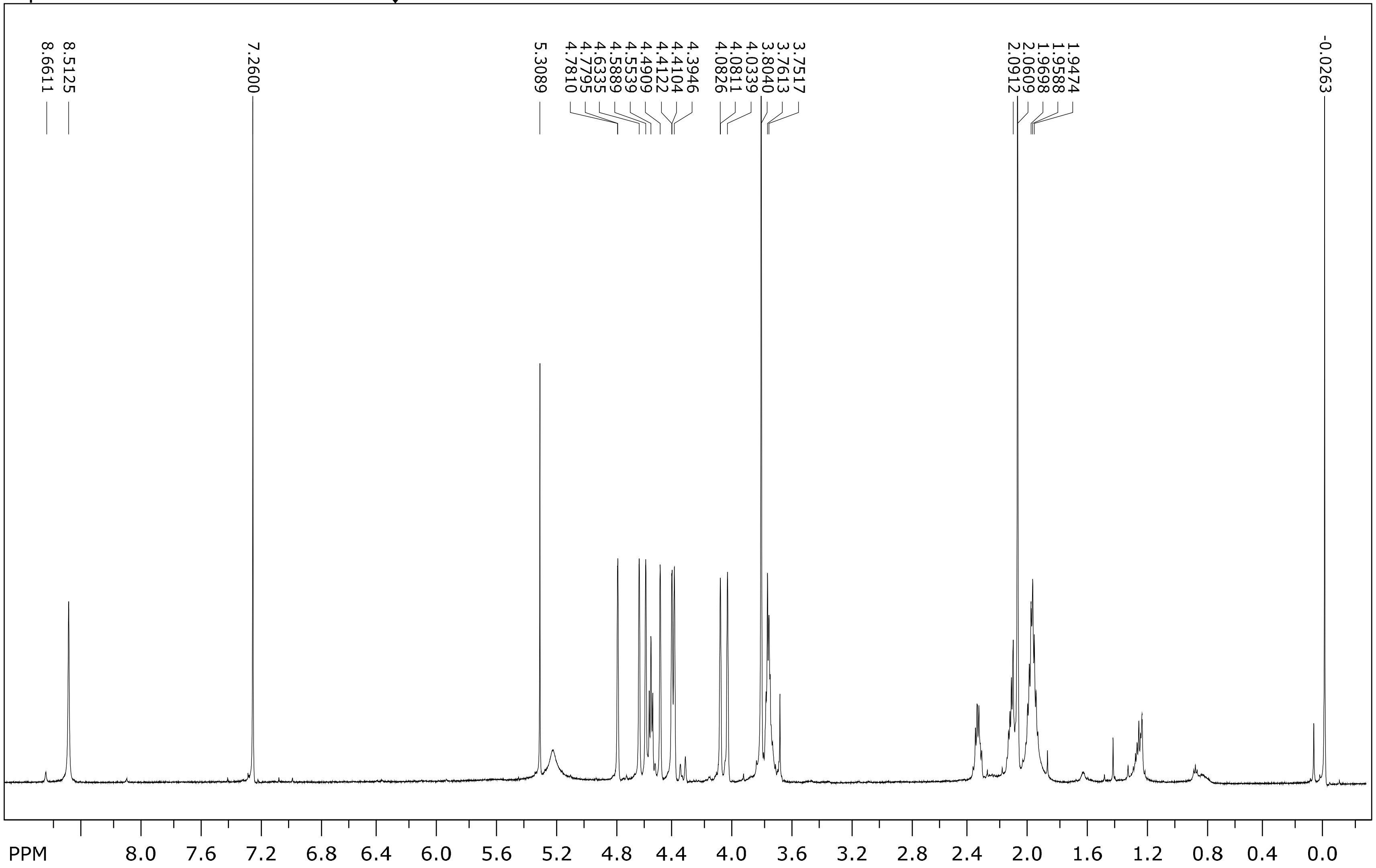

Compound 2

Figure S6. MALDI TOF/TOF-MS spectrum of **2**.

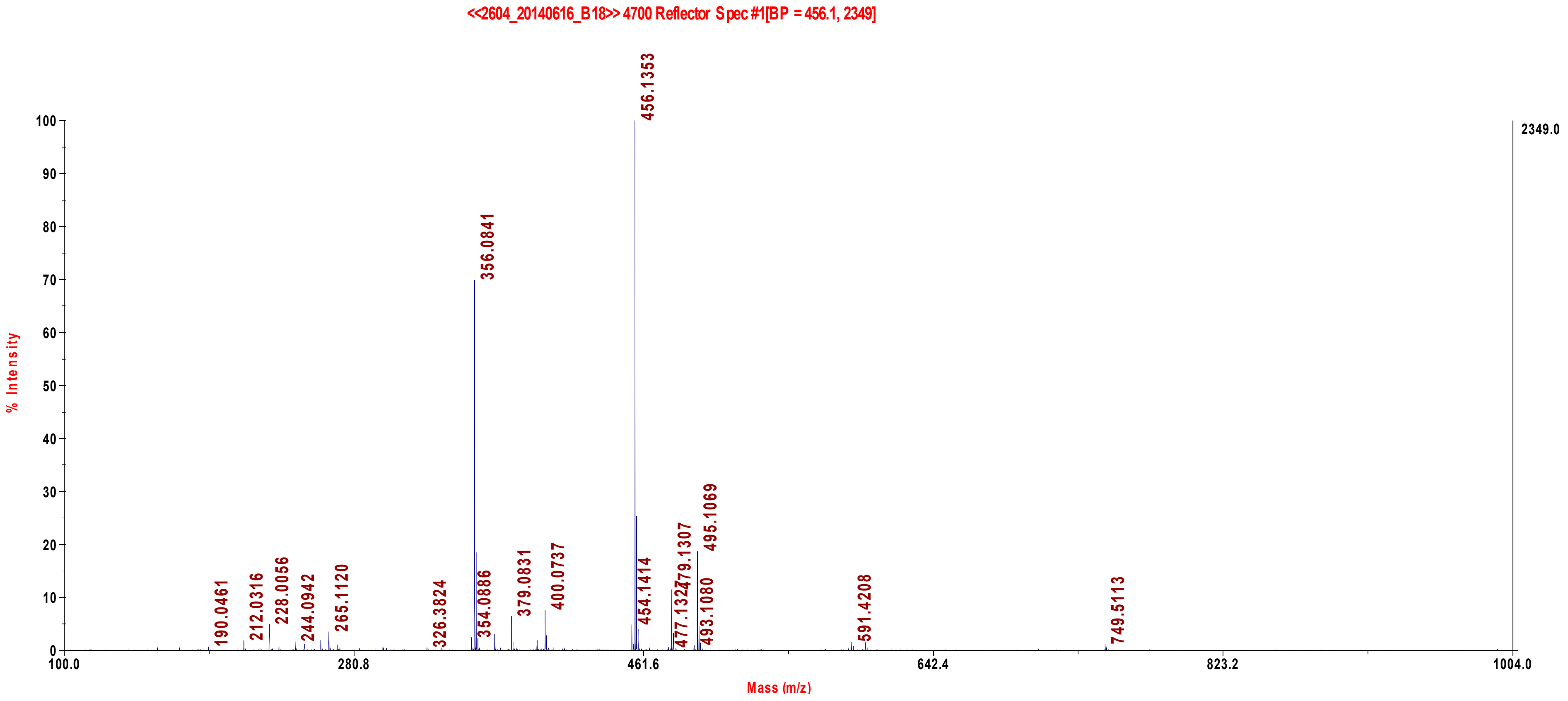

Figure S7. ^{1}H NMR spectra of **2**.

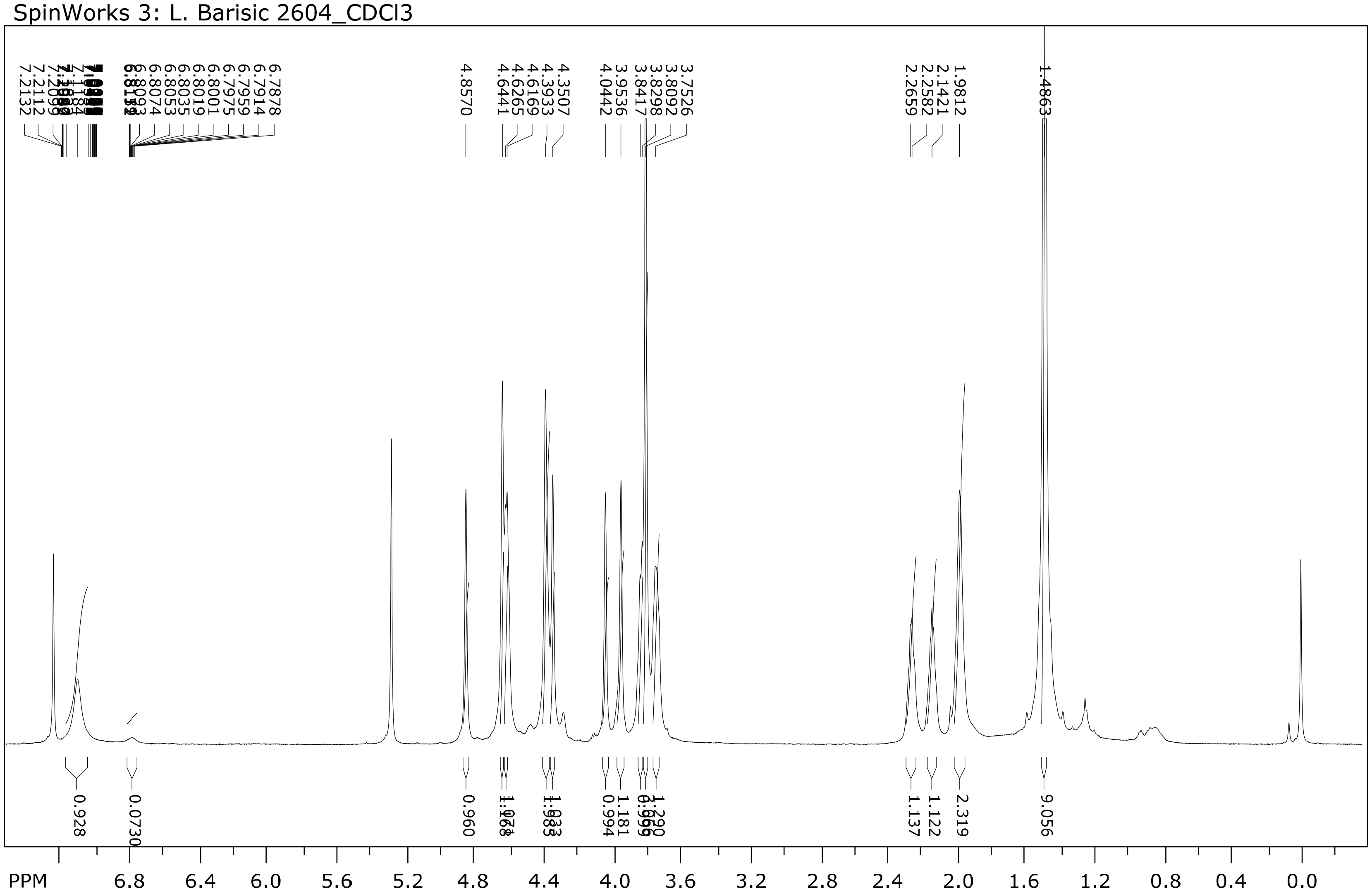

SpinWorks 3: L. Barisic 2604_CDCl3

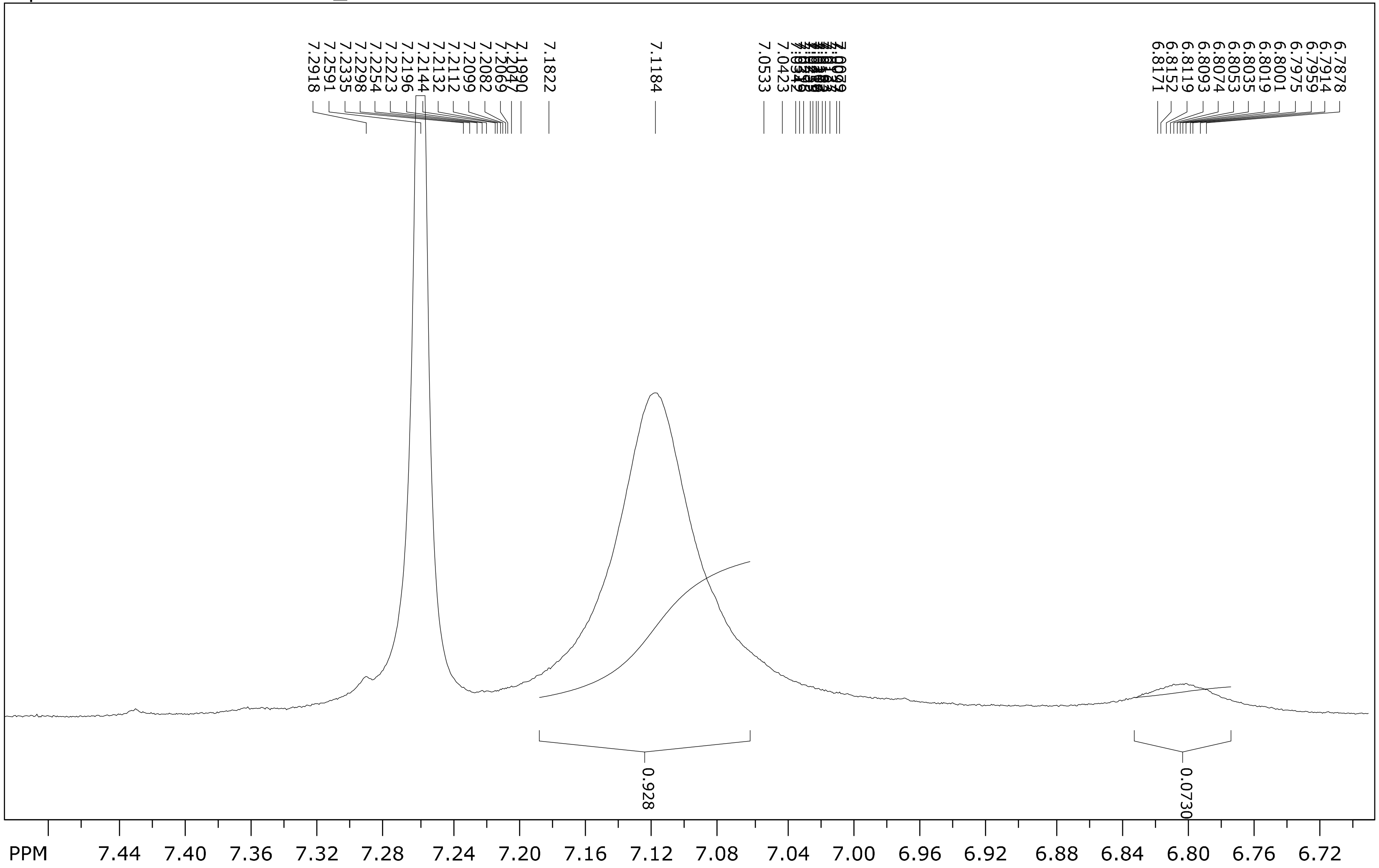

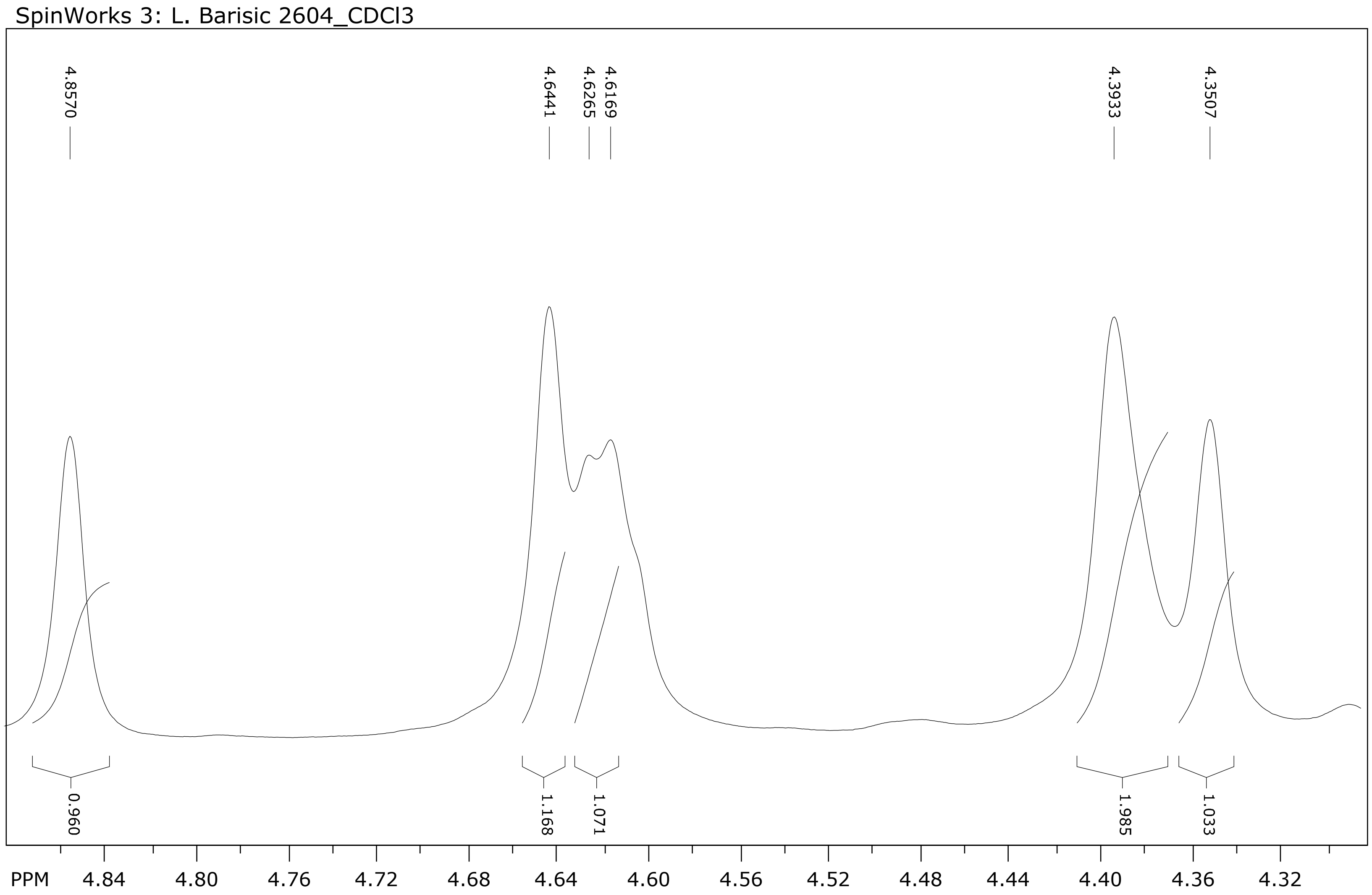
SpinWorks 3: L. Barisic 2604_CDCl3
4.8570
4.6441
4.6265
4.6169
4.3933
4.3507
0.960
1.168
1.071
1.985
1.033
PPM
4.84
4.80
4.76
4.72
4.68
4.64
4.60
4.56
4.52
4.48
4.44
4.40
4.36
4.32

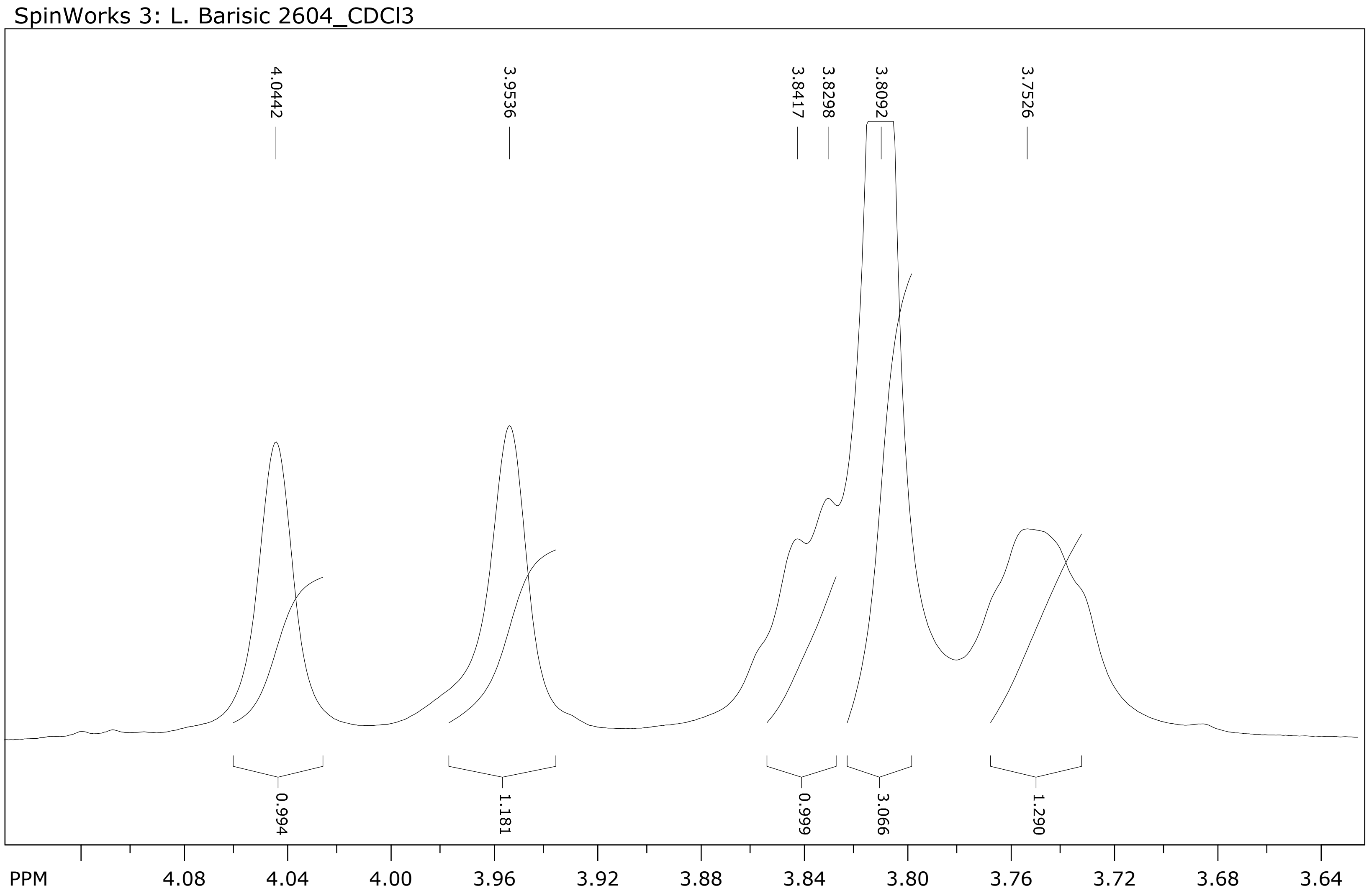
SpinWorks 3: L. Barisic 2604_CDCl3
4.0442
3.9536
3.8417
3.8298
3.8092
3.7526
0.994
1.181
0.999
3.066
1.290
PPM
4.08
4.04
4.00
3.96
3.92
3.88
3.84
3.80
3.76
3.72
3.68
3.64

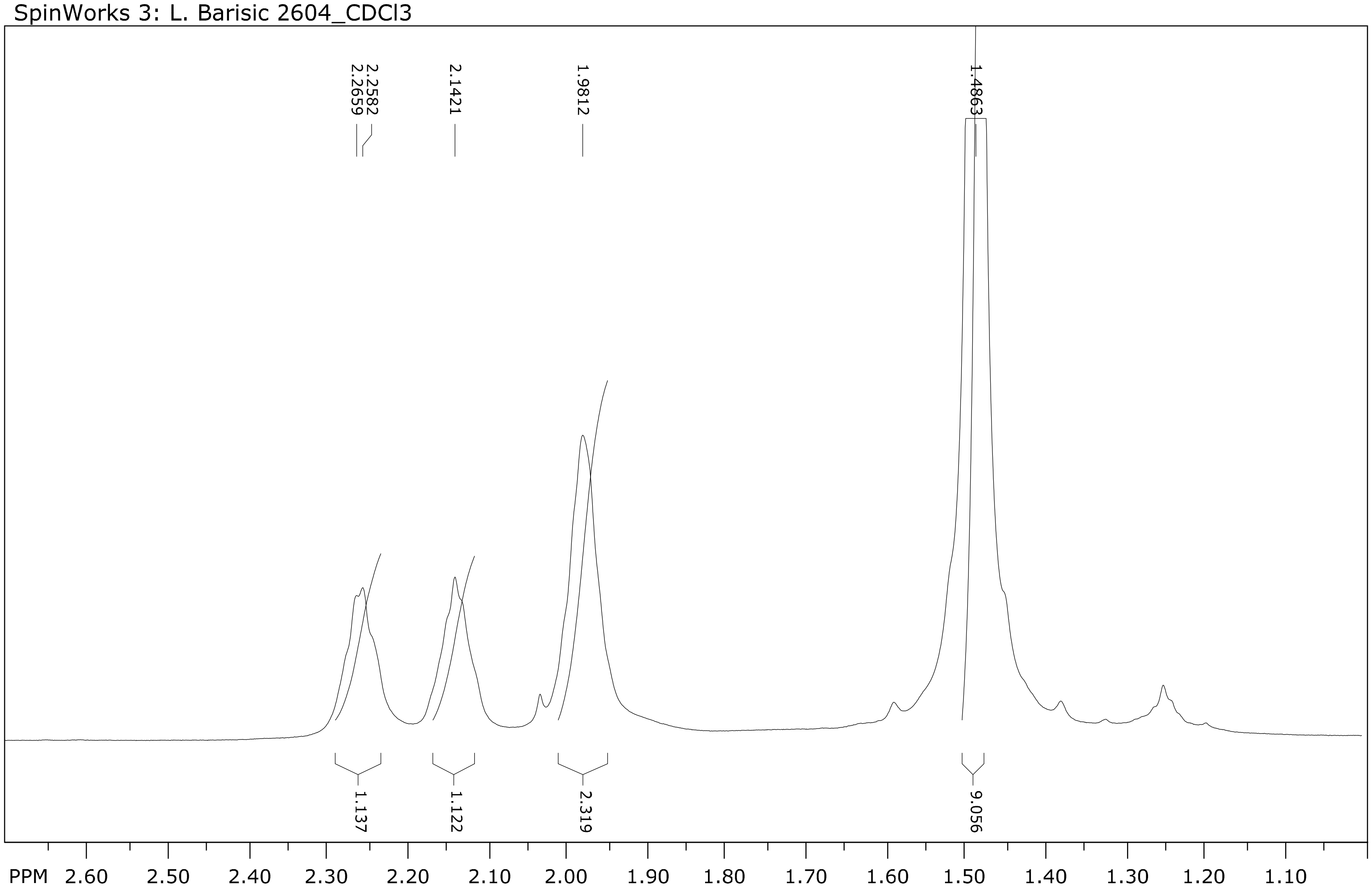
SpinWorks 3: L. Barisic 2604_CDCl3
2.2582
2.2659
2.1421
1.9812
1.4863
1.137
1.122
2.319
9.056
PPM 2.60 2.50 2.40 2.30 2.20 2.10 2.00 1.90 1.80 1.70 1.60 1.50 1.40 1.30 1.20 1.10

Figure S8. ^{13}C NMR spectra of **2.**

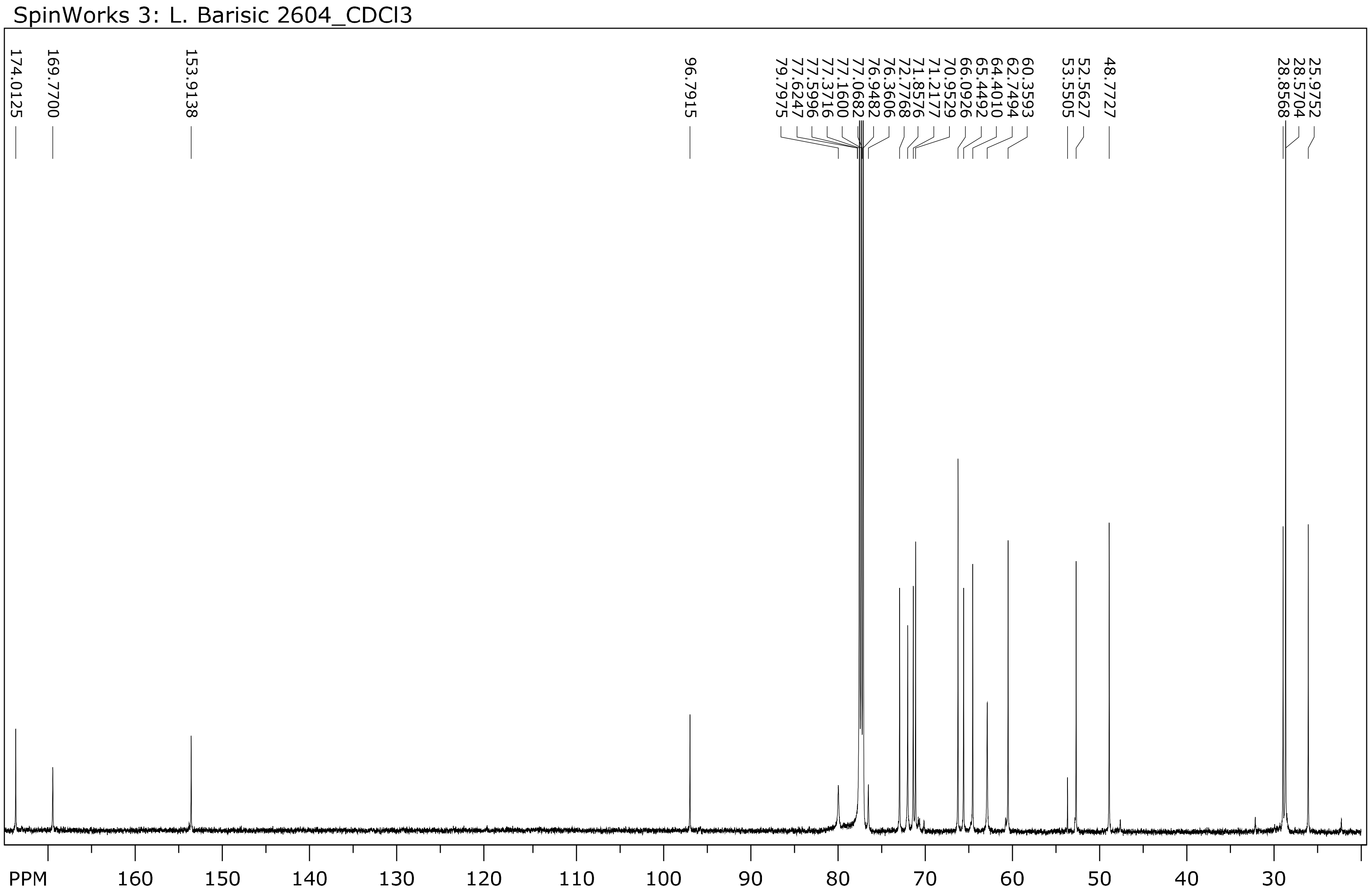

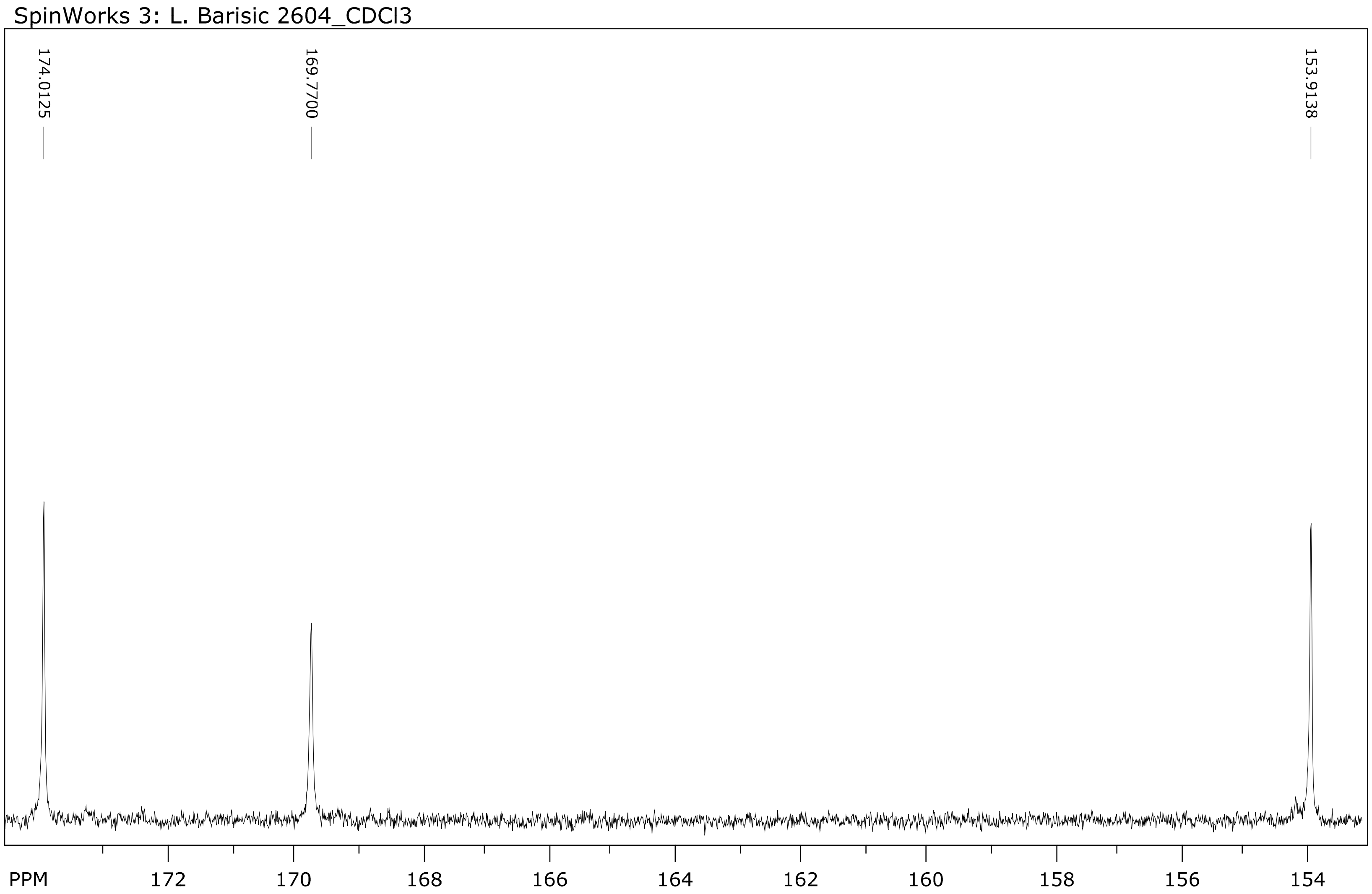
SpinWorks 3: L. Barisic 2604_CDCl3
174.0125
169.7700
153.9138
PPM
172
170
168
166
164
162
160
158
156
154

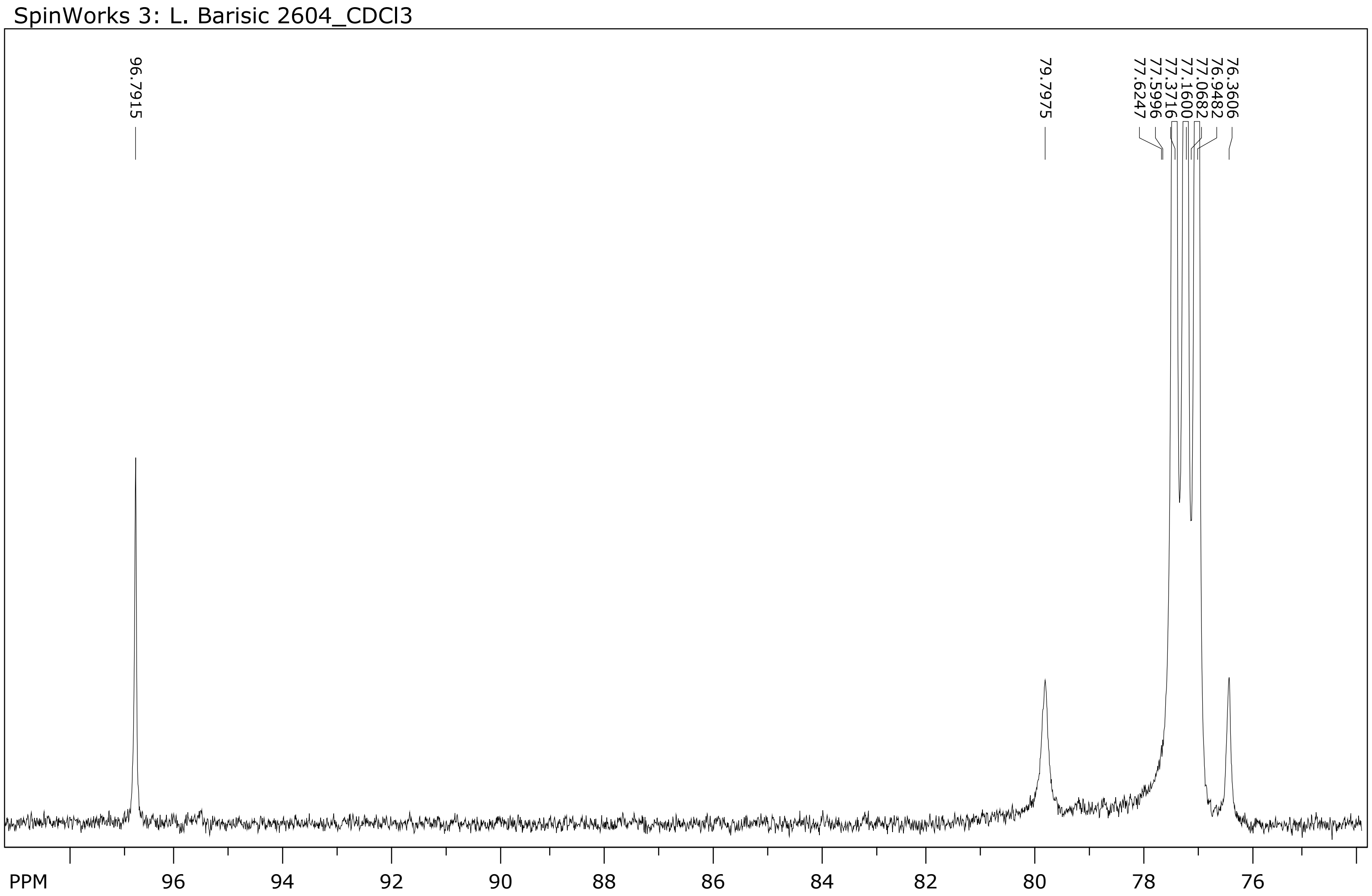
SpinWorks 3: L. Barisic 2604_CDCl3
96.7915
79.7975
77.6247
77.5996
77.3716
77.1600
77.0682
76.9482
76.3606
PPM
96
94
92
90
88
86
84
82
80
78
76

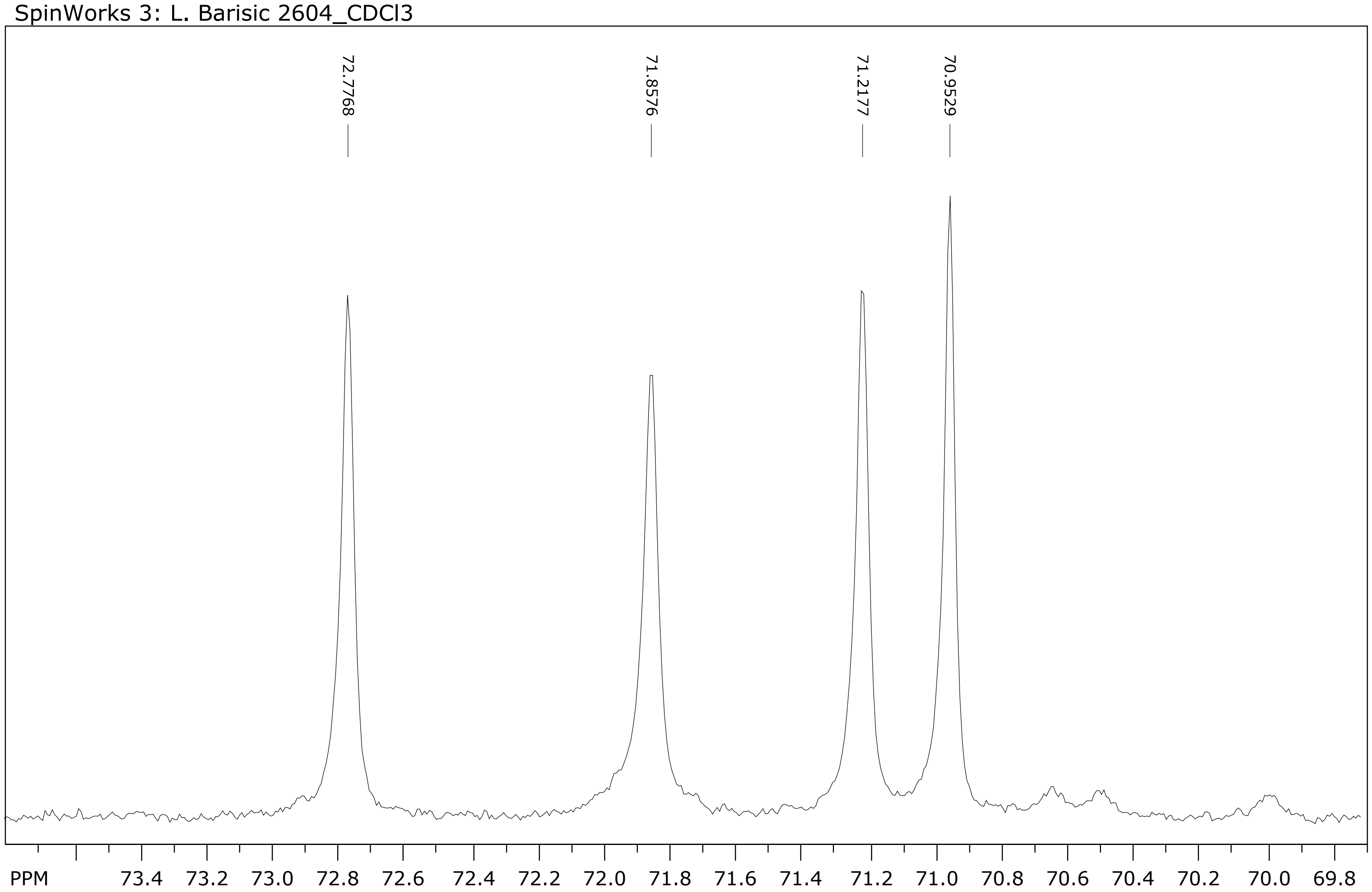

SpinWorks 3: L. Barisic 2604_CDCl3
72.7768
71.8576
71.2177
70.9529
PPM
73.4
73.2
73.0
72.8
72.6
72.4
72.2
72.0
71.8
71.6
71.4
71.2
71.0
70.8
70.6
70.4
70.2
70.0
69.8

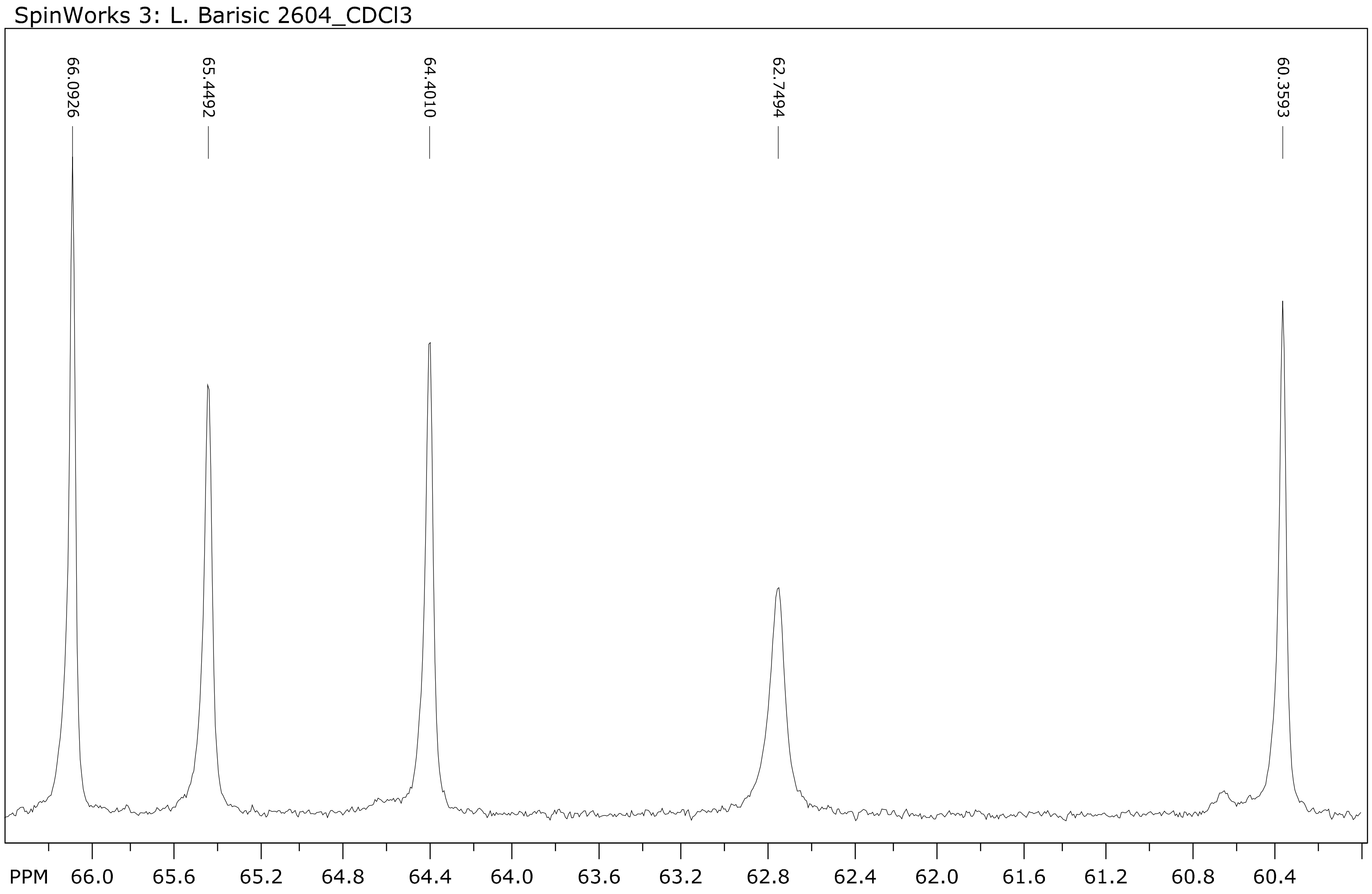
SpinWorks 3: L. Barisic 2604_CDCl3
66.0926
65.4492
64.4010
62.7494
60.3593
PPM
66.0
65.6
65.2
64.8
64.4
64.0
63.6
63.2
62.8
62.4
62.0
61.6
61.2
60.8
60.4

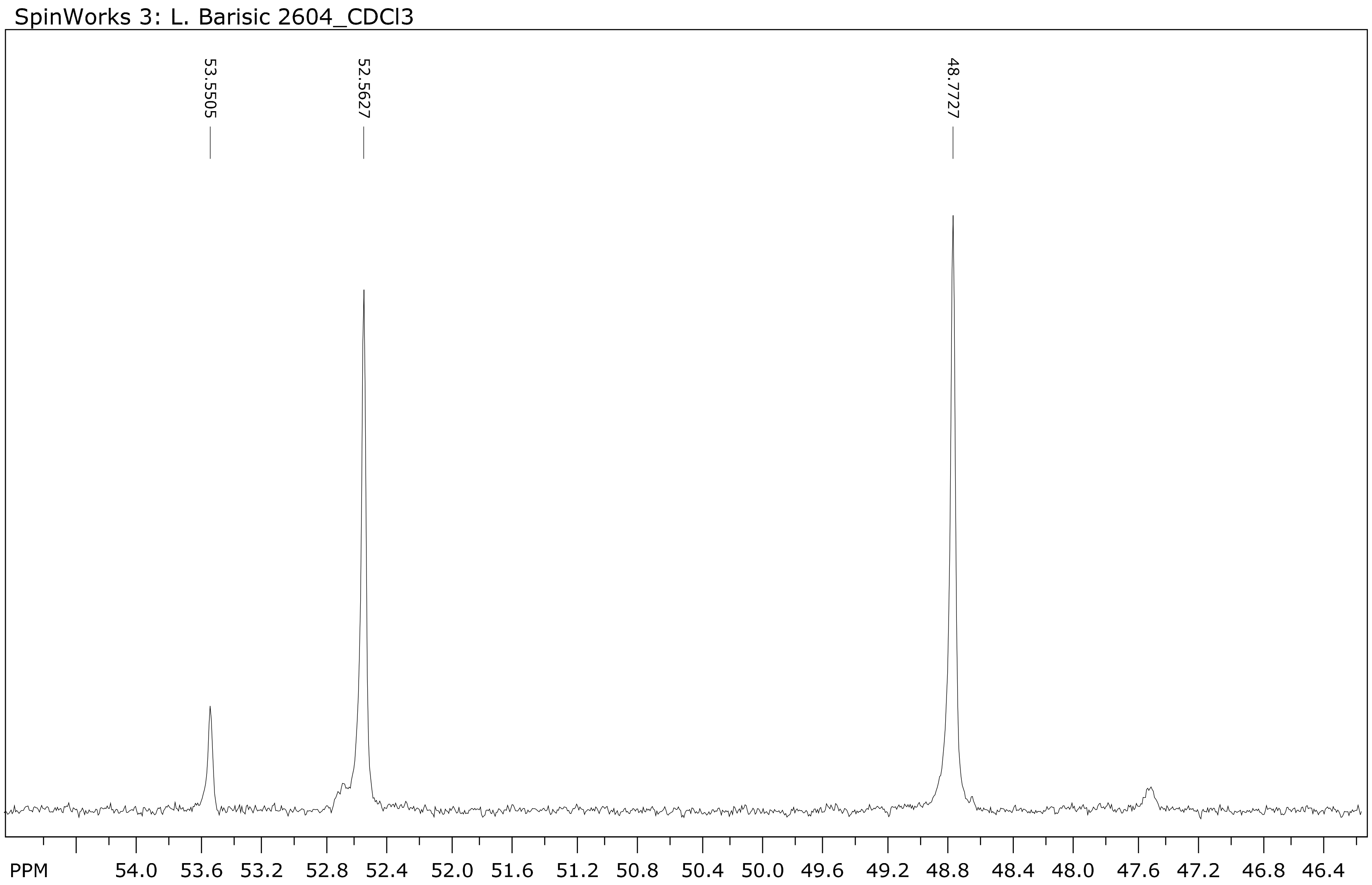
SpinWorks 3: L. Barisic 2604_CDCl3
53.5505
52.5627
48.7727
PPM
54.0 53.6 53.2 52.8 52.4 52.0 51.6 51.2 50.8 50.4 50.0 49.6 49.2 48.8 48.4 48.0 47.6 47.2 46.8 46.4

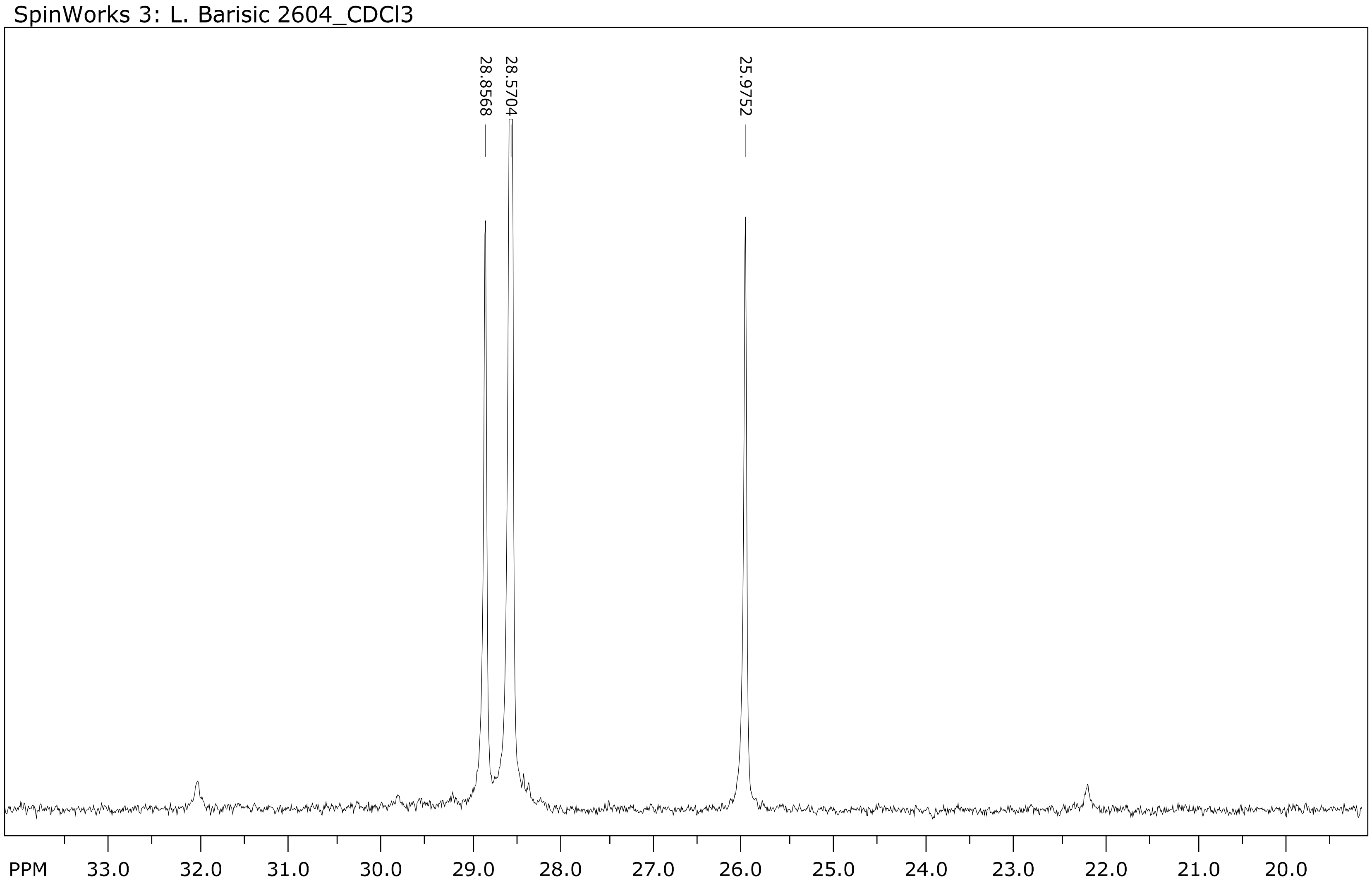
SpinWorks 3: L. Barisic 2604_CDCl3
28.8568
28.5704
25.9752
PPM
33.0
32.0
31.0
30.0
29.0
28.0
27.0
26.0
25.0
24.0
23.0
22.0
21.0
20.0

Figure S9. ^{1}H NMR titration of compound **2** with DMSO in $CDCl_3$.

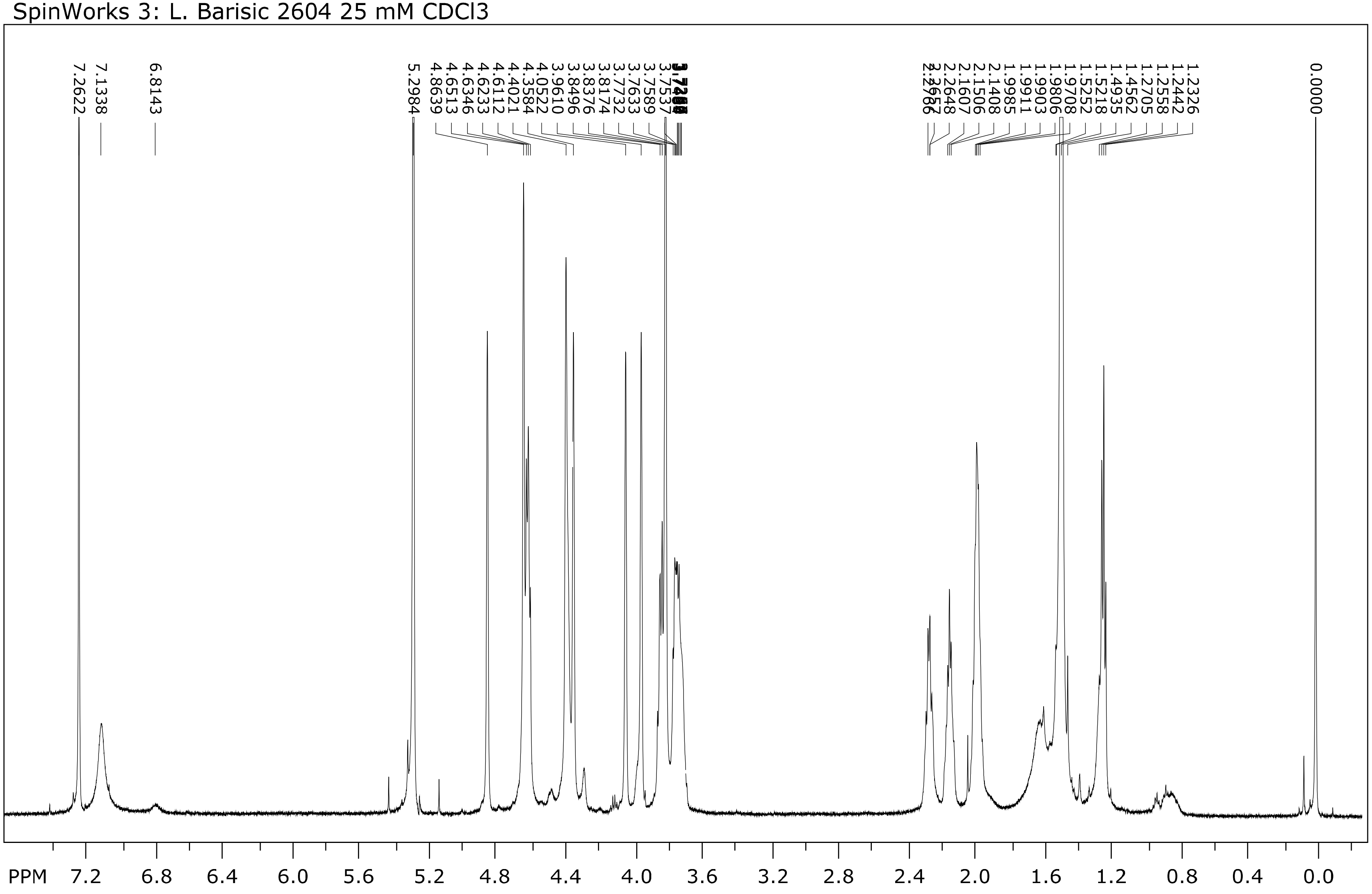

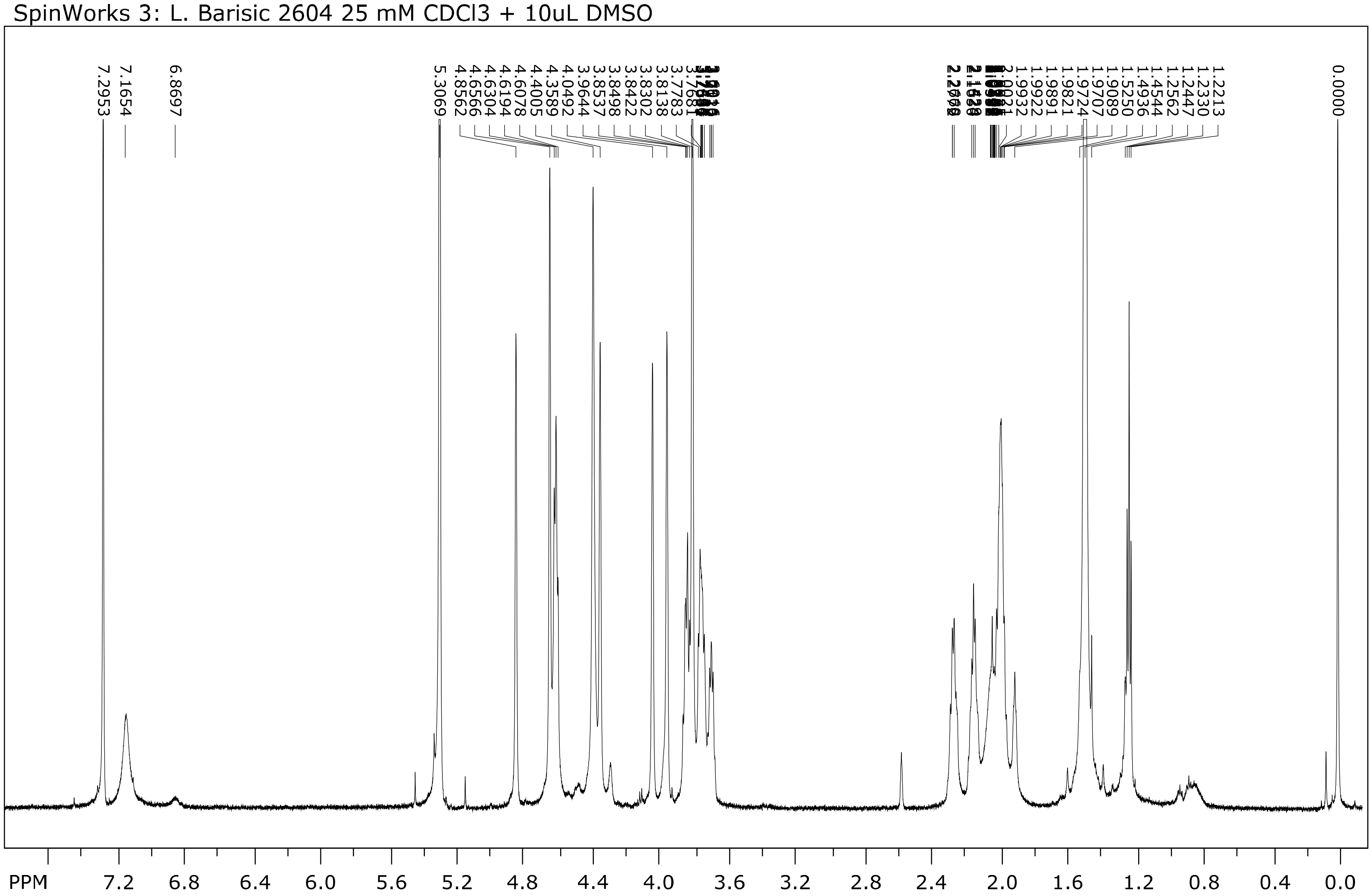
SpinWorks 3: L. Barisic 2604 25 mM CDCl3 + 10uL DMSO
PPM
7.2 6.8 6.4 6.0 5.6 5.2 4.8 4.4 4.0 3.6 3.2 2.8 2.4 2.0 1.6 1.2 0.8 0.4 0.0

SpinWorks 3: L. Barisic 2604 25 mM CDCl3 + 20uL DMSO

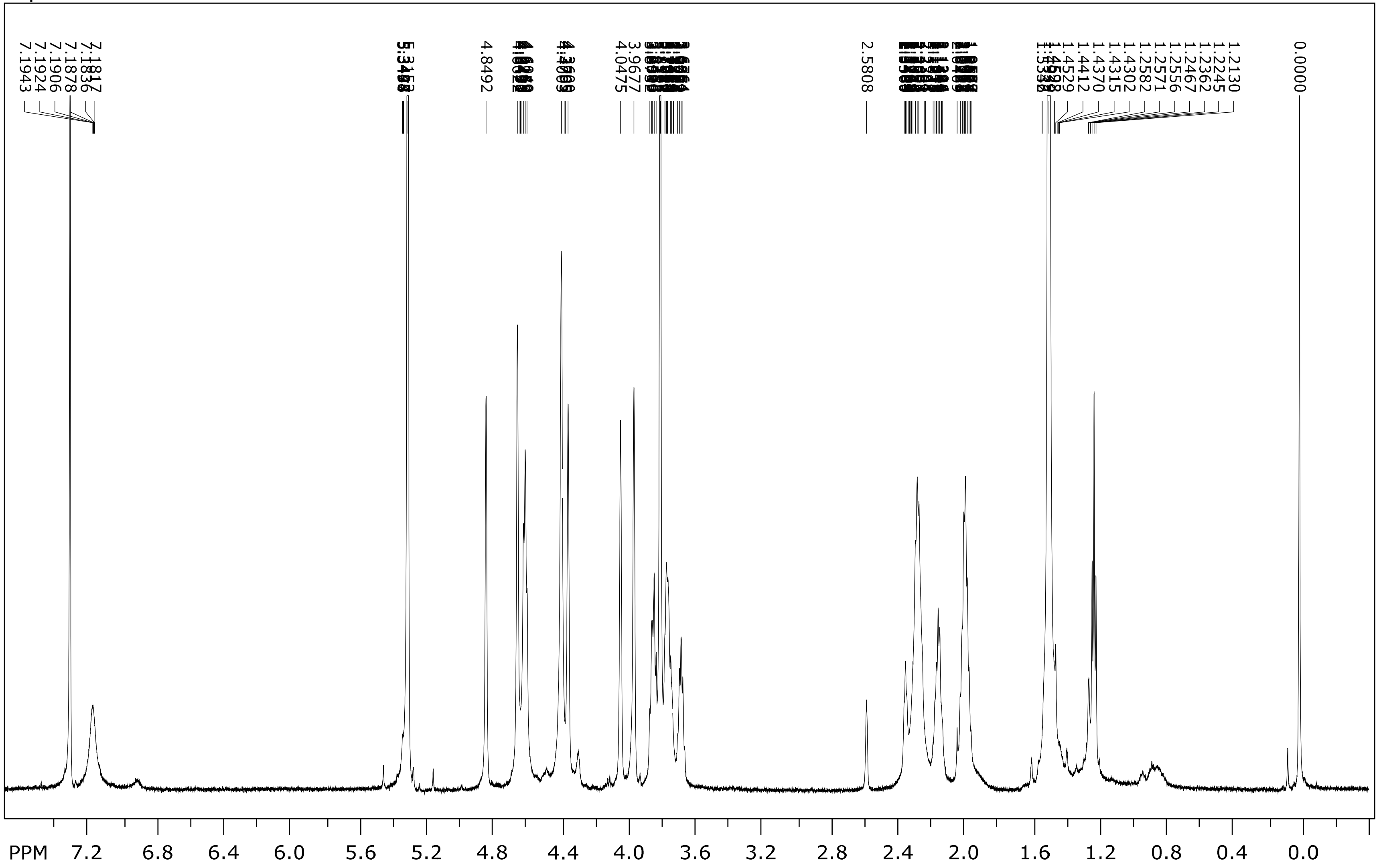

SpinWorks 3: L. Barisic 2604 25 mM CDCl3 + 30uL DMSO

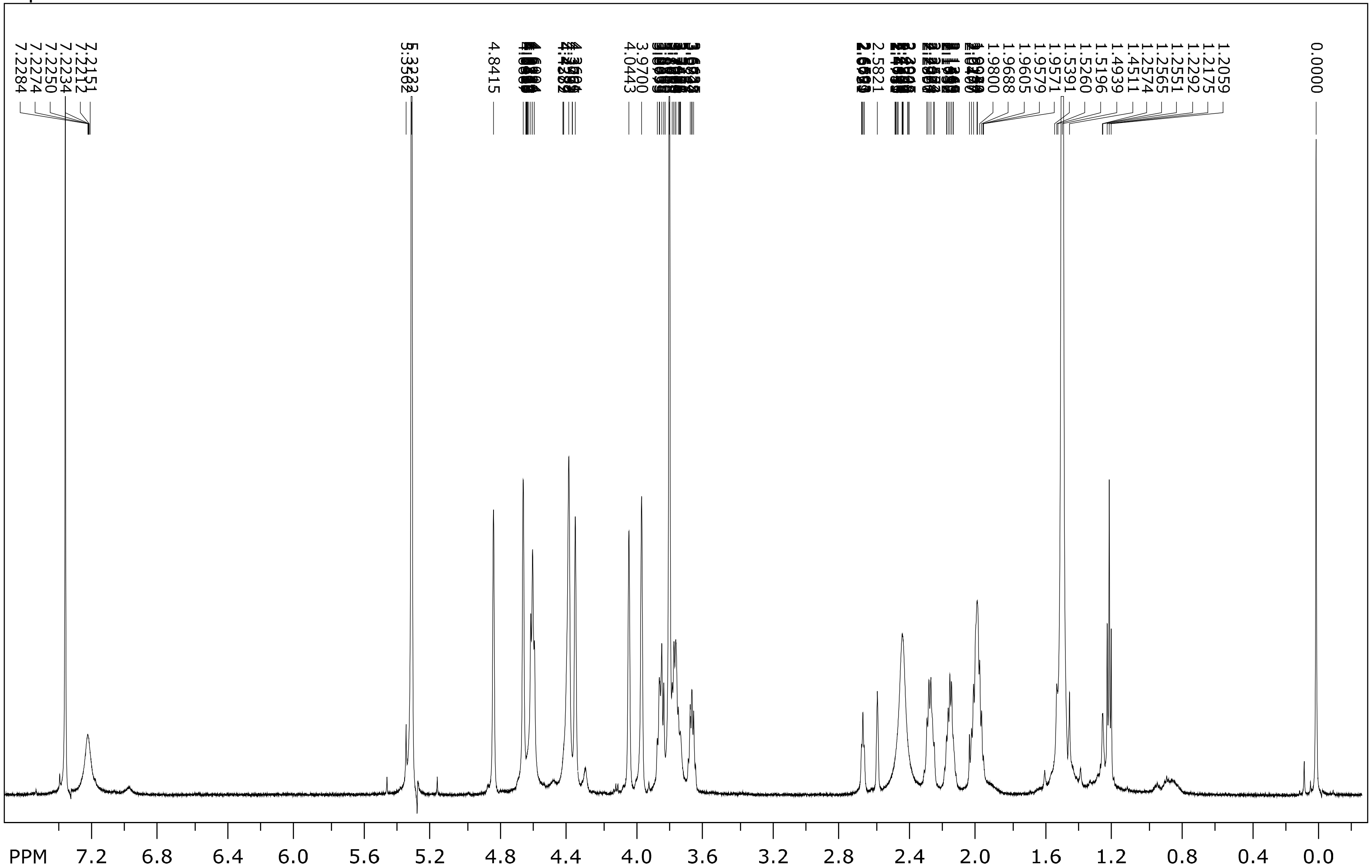

SpinWorks 3: L. Barisic 2604 25 mM CDCl3 + 40uL DMSO

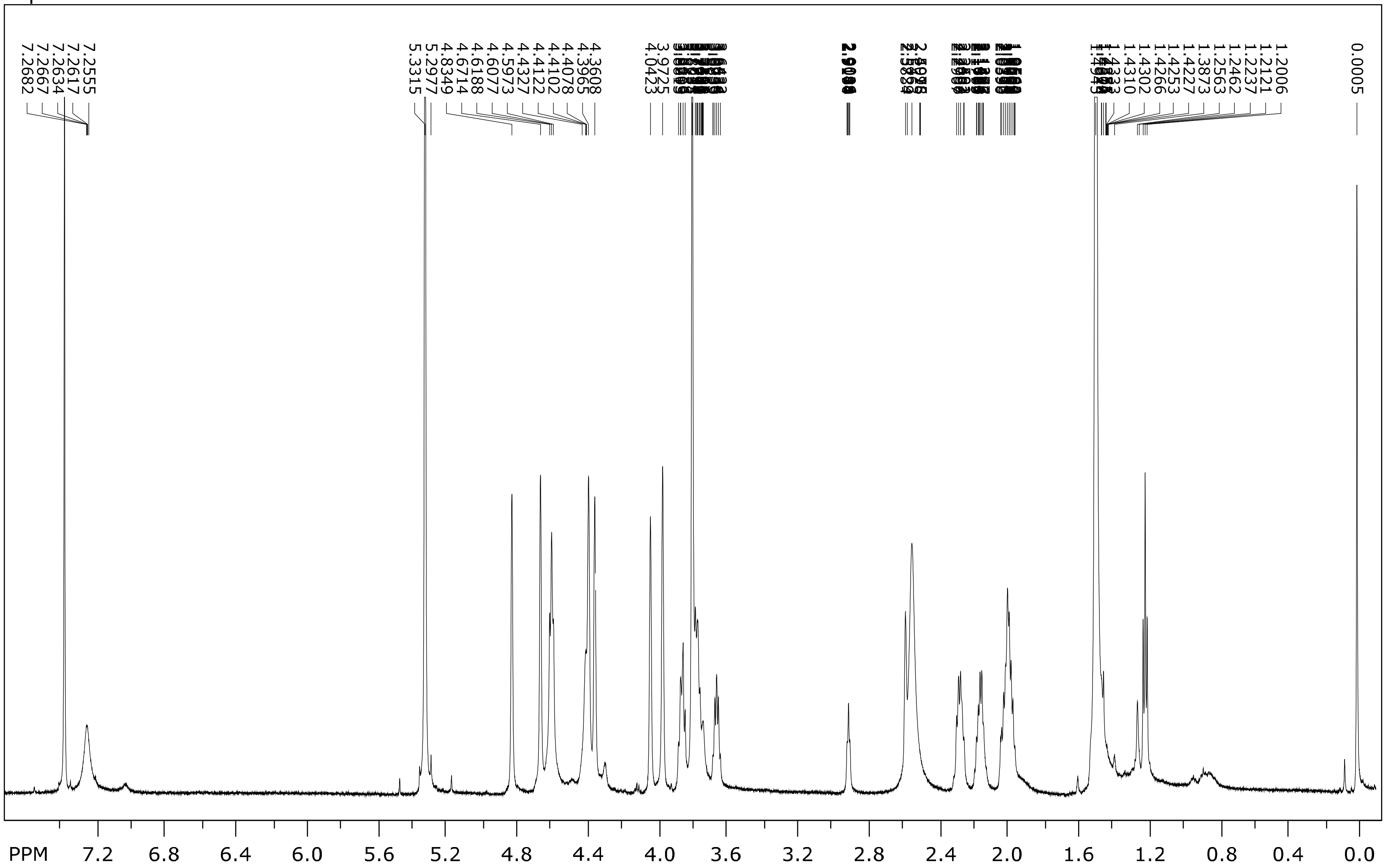

SpinWorks 3: L. Barisic 2604 25 mM CDCl3 + 50uL DMSO

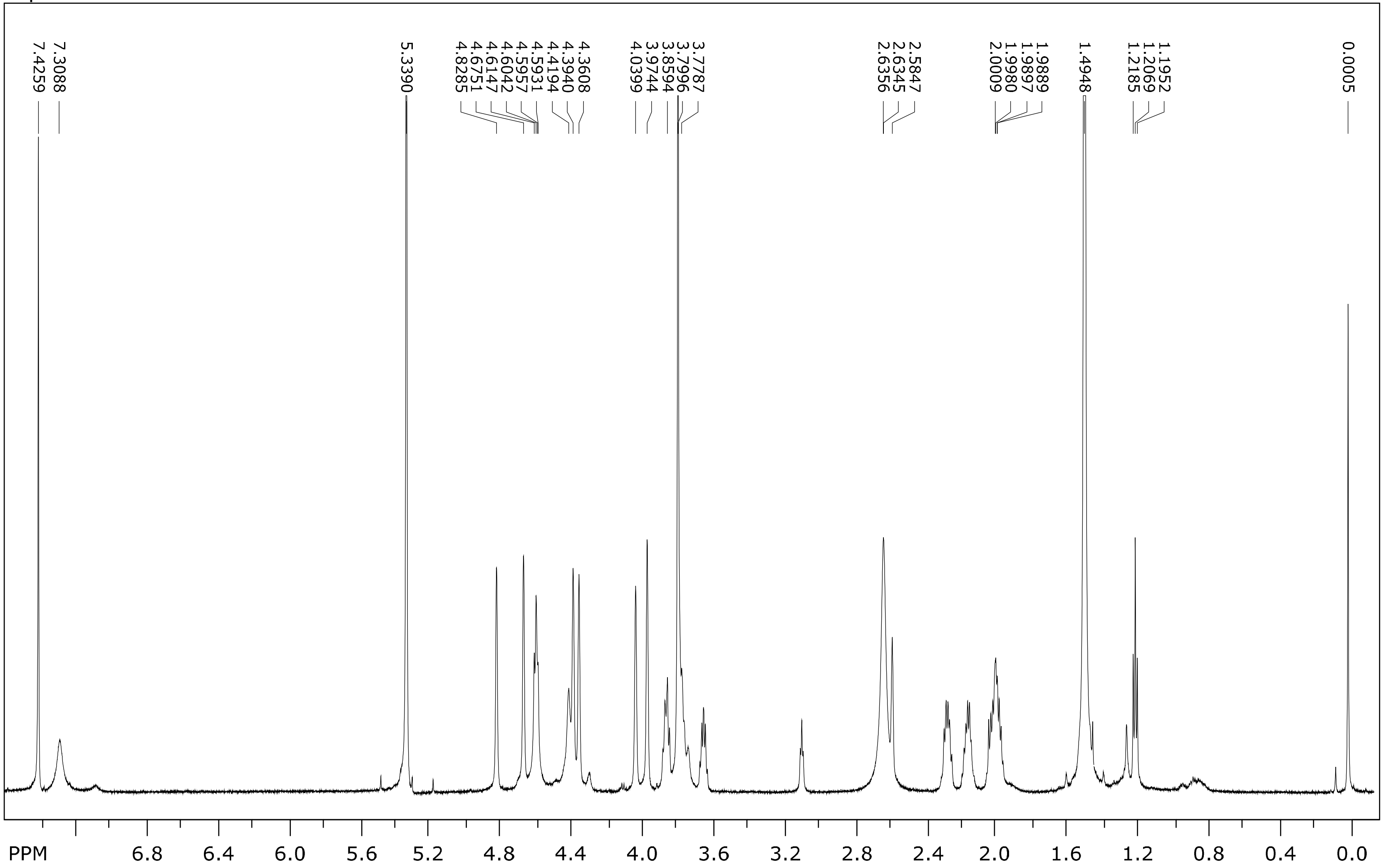

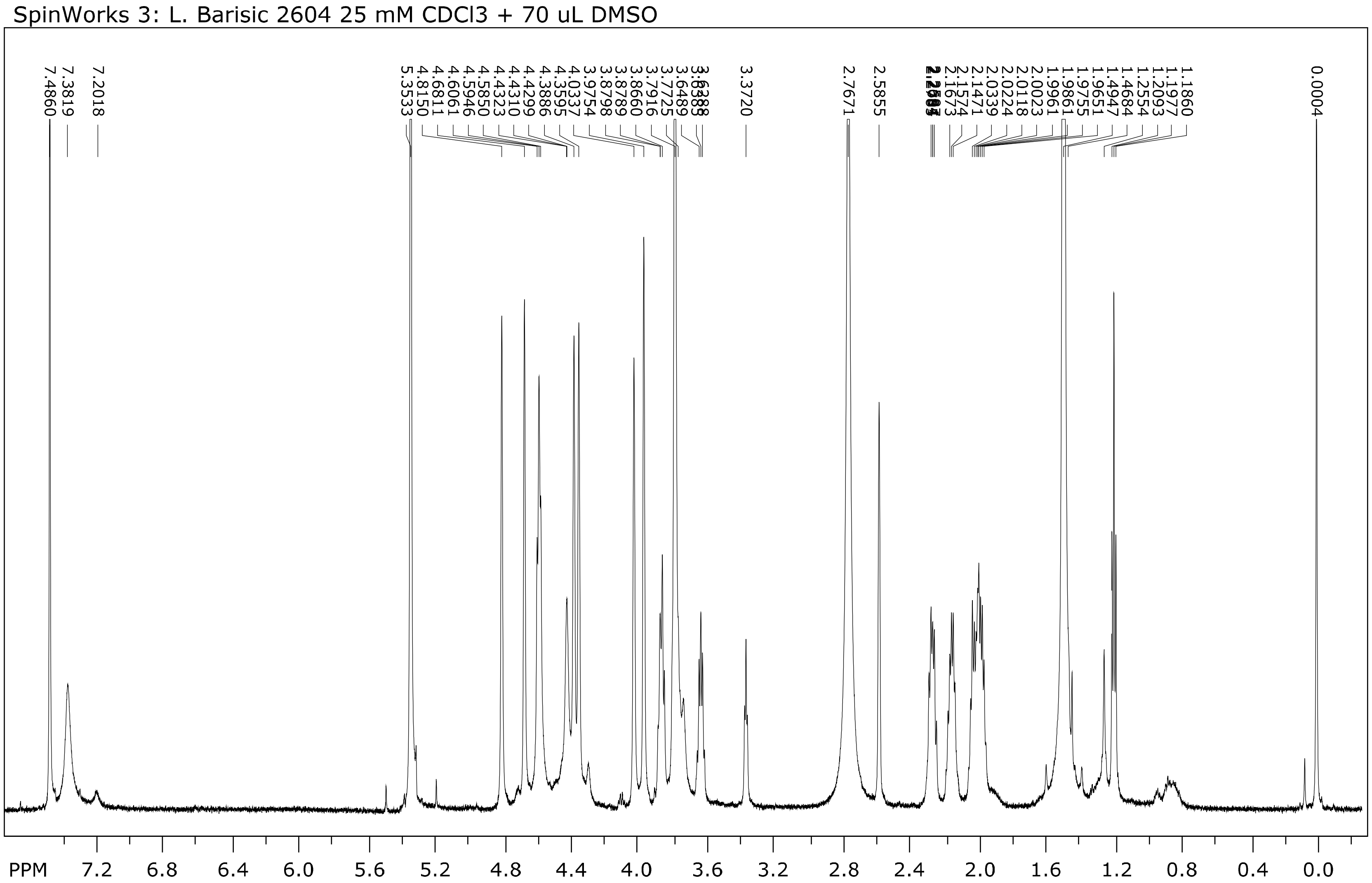
SpinWorks 3: L. Barisic 2604 25 mM CDCl3 + 70 uL DMSO
7.4860
7.3819
7.2018
5.3533
4.8150
4.6811
4.6061
4.5946
4.5850
4.4323
4.4310
4.4299
4.3886
4.3595
4.0337
3.9754
3.8798
3.8789
3.8660
3.7916
3.7725
3.6489
3.3720
2.7671
2.5855
2.1673
2.1574
2.1471
2.0339
2.0224
2.0118
2.0023
1.9961
1.9861
1.9755
1.9651
1.4947
1.4684
1.2554
1.2093
1.1977
1.1860
0.0004
PPM
7.2
6.8
6.4
6.0
5.6
5.2
4.8
4.4
4.0
3.6
3.2
2.8
2.4
2.0
1.6
1.2
0.8
0.4
0.0

SpinWorks 3: L. Barisic 2604 25 mM CDCl3 + 90 uL DMSO

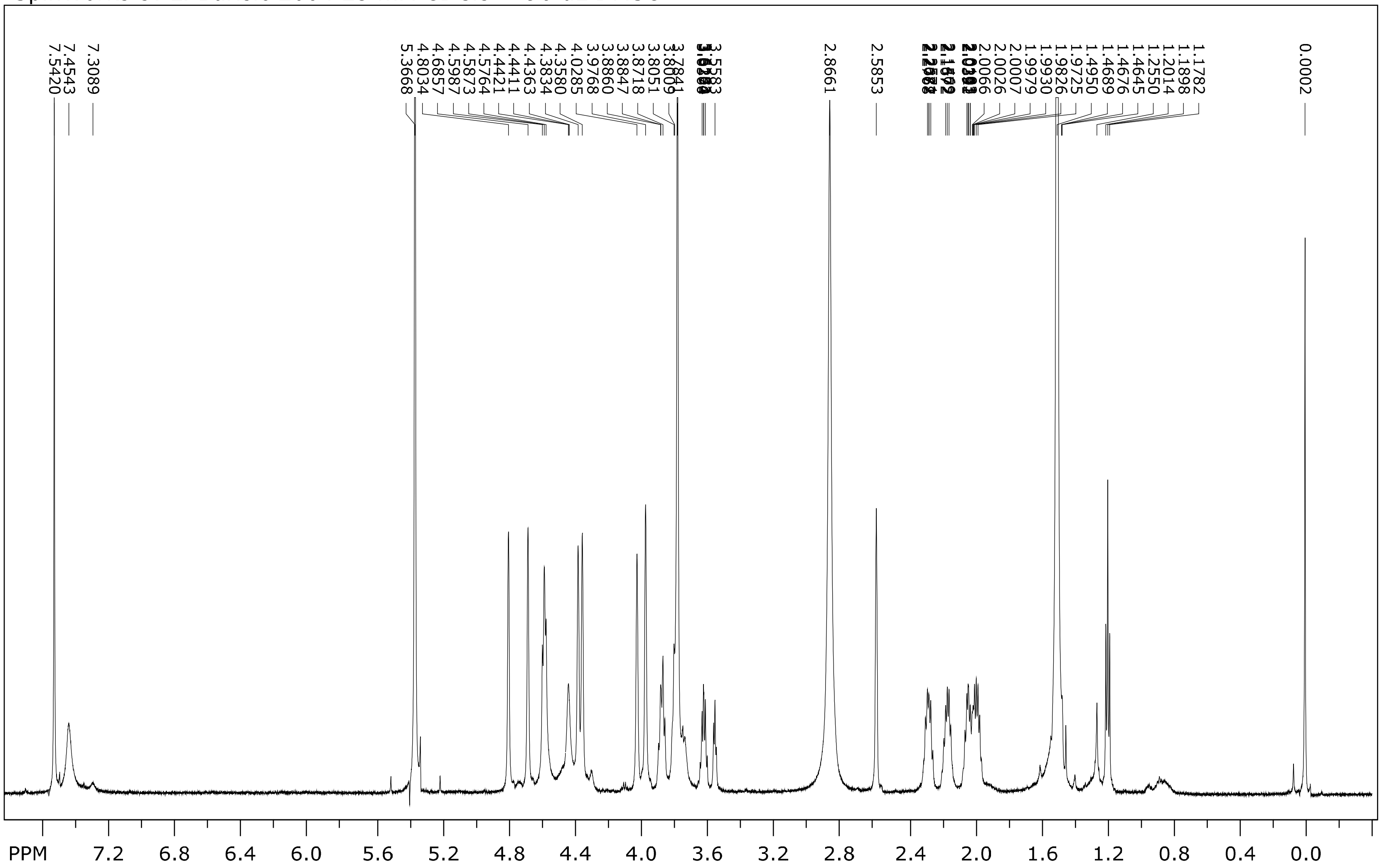

SpinWorks 3: L. Barisic 2604 25 mM CDCl3 + 110 uL DMSO

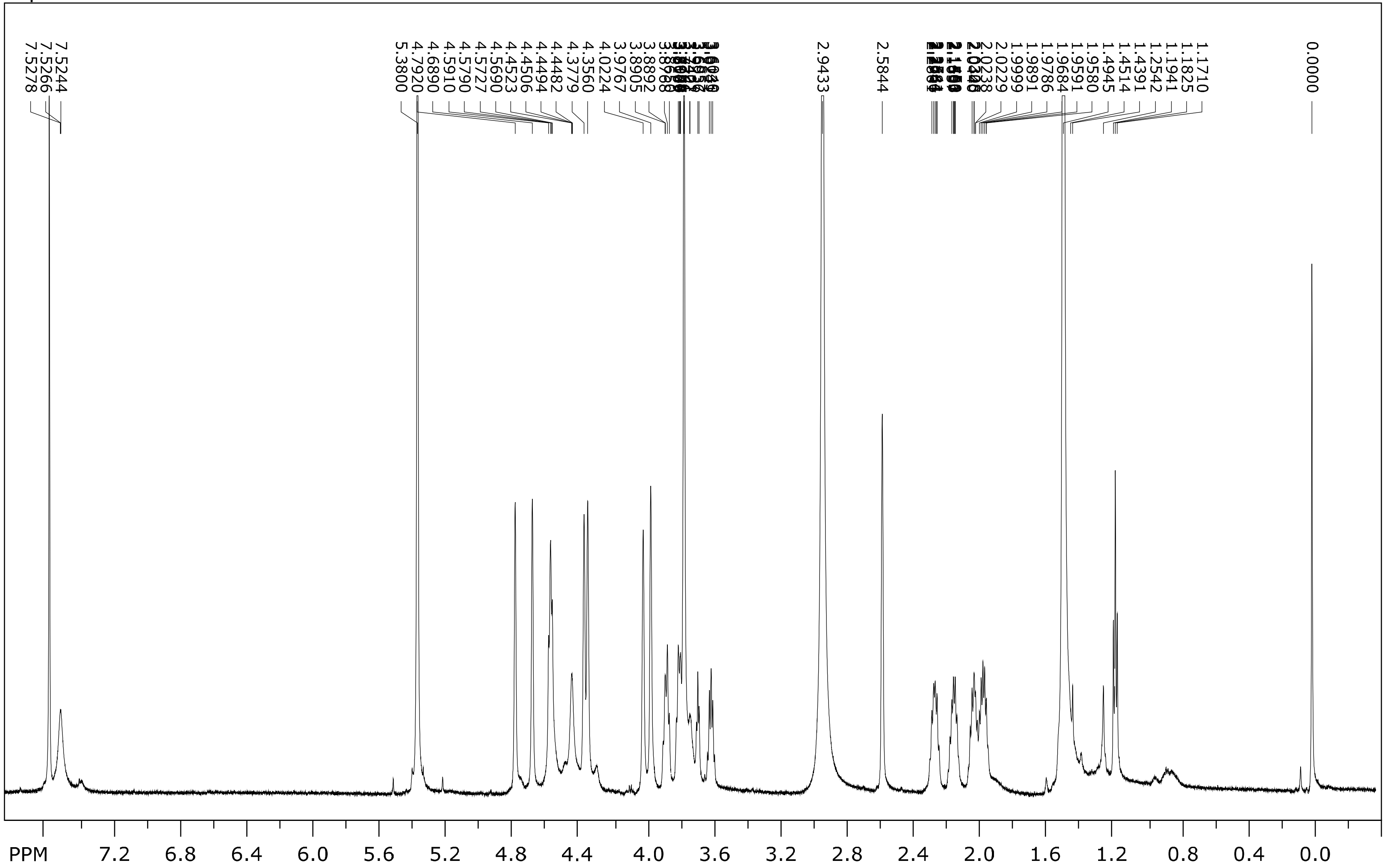

SpinWorks 3: L. Barisic 2604 25 mM CDCl3 + 130 uL DMSO

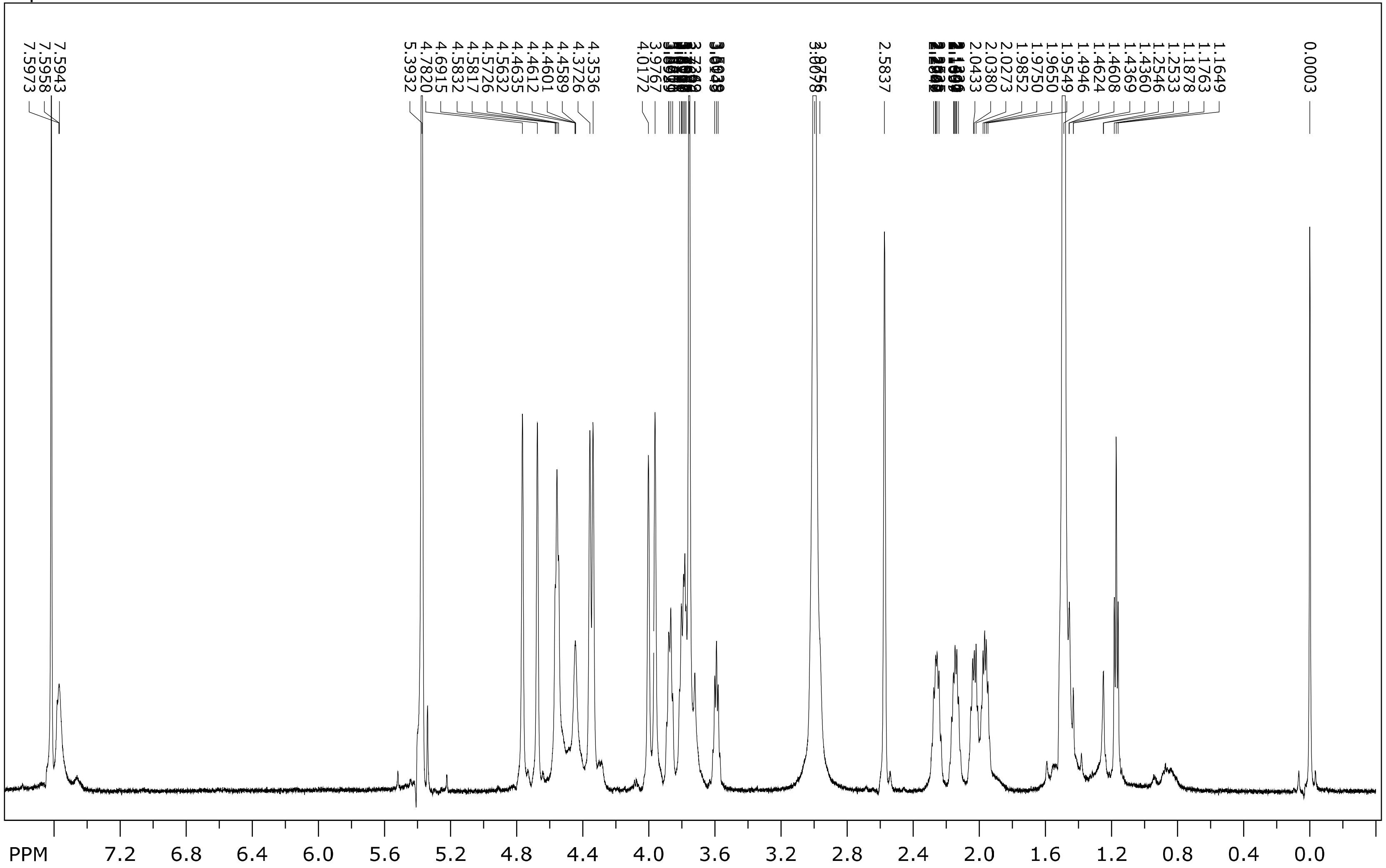

SpinWorks 3: L. Barisic 2604 25 mM CDCl3 + 180 uL DMSO

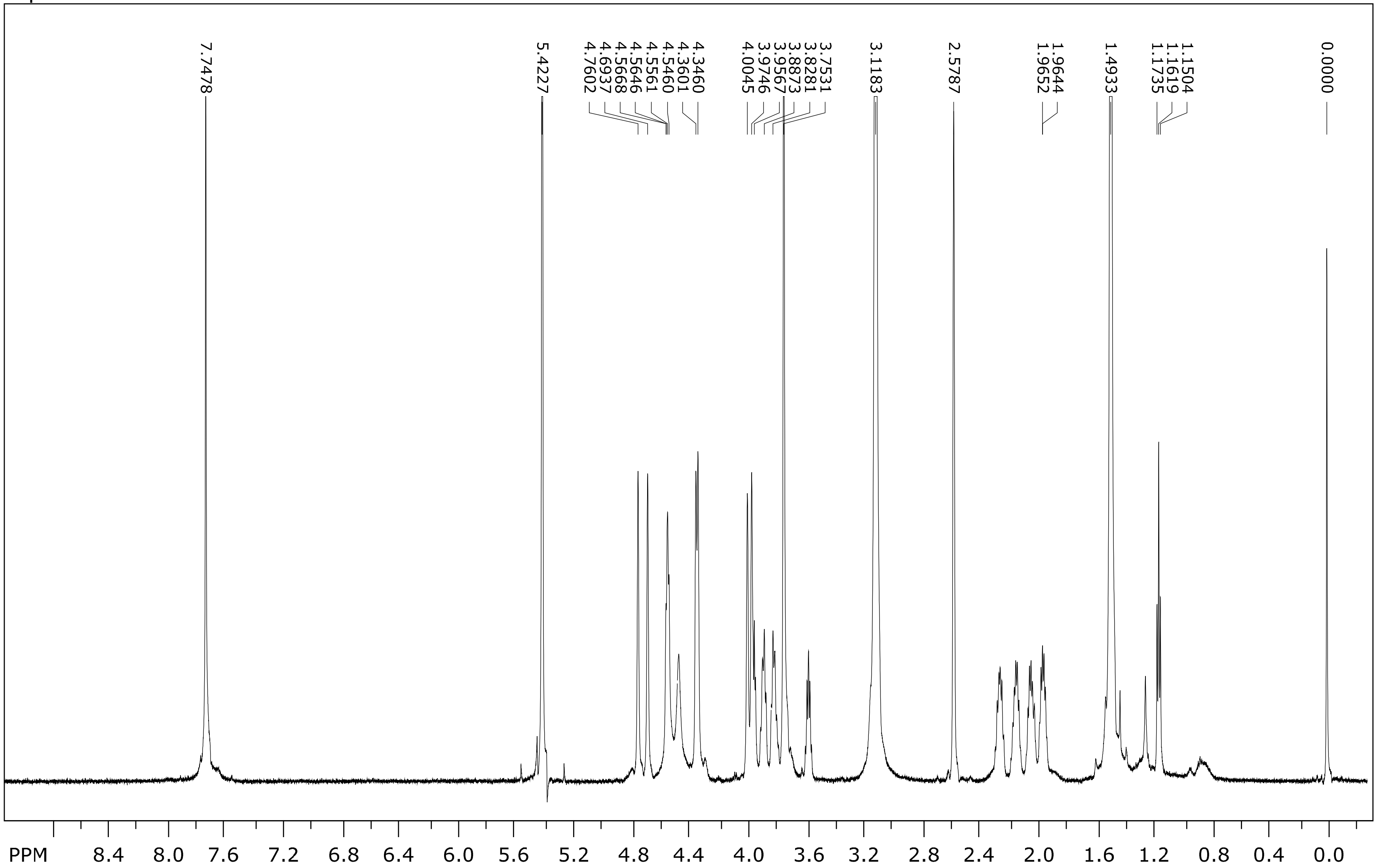

SpinWorks 3: L. Barisic 2604 25 mM CDCl3 + 280 uL DMSO

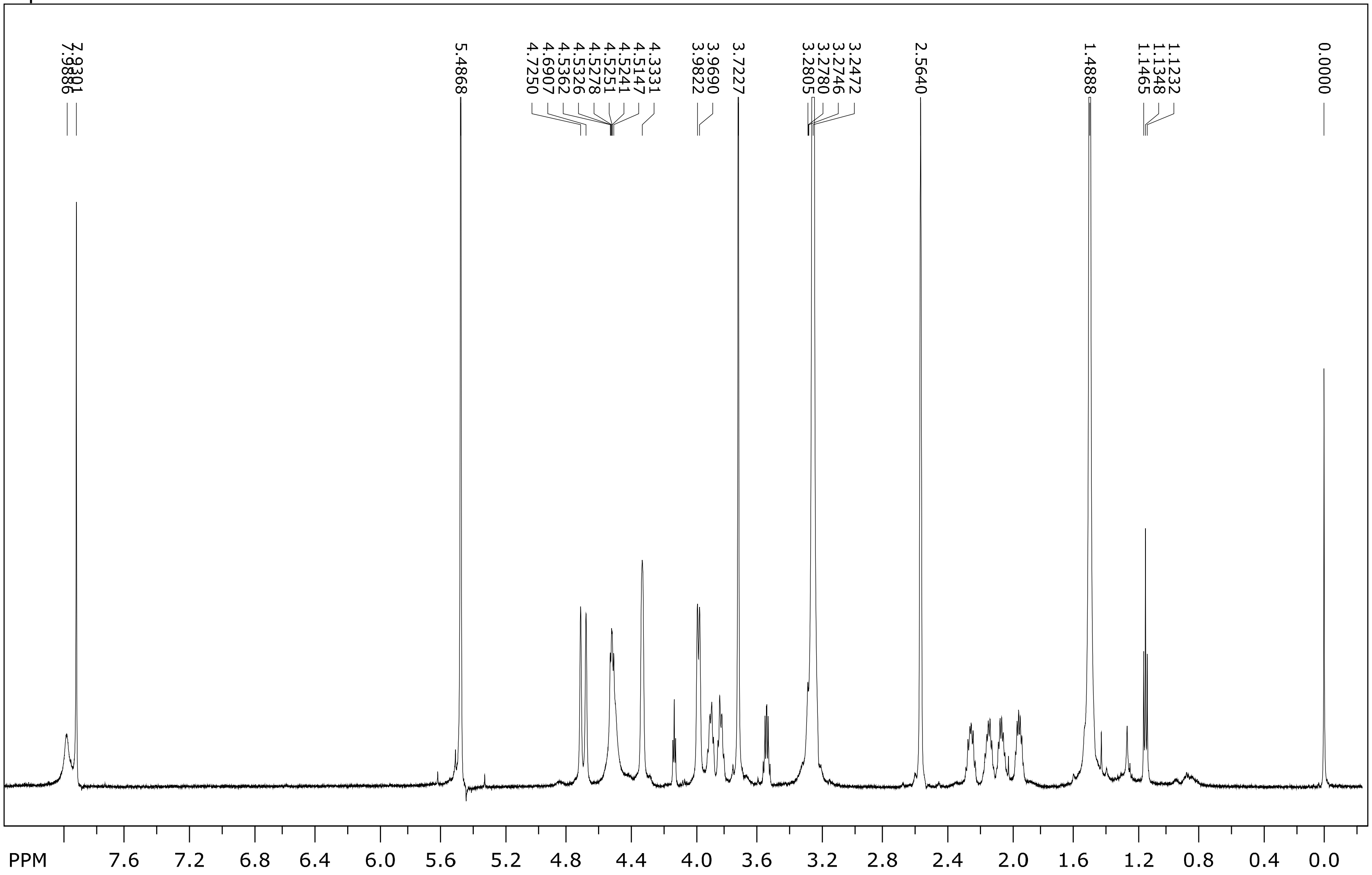

SpinWorks 3: L. Barisic 2604 25 mM CDCl3 + 430 uL DMSO

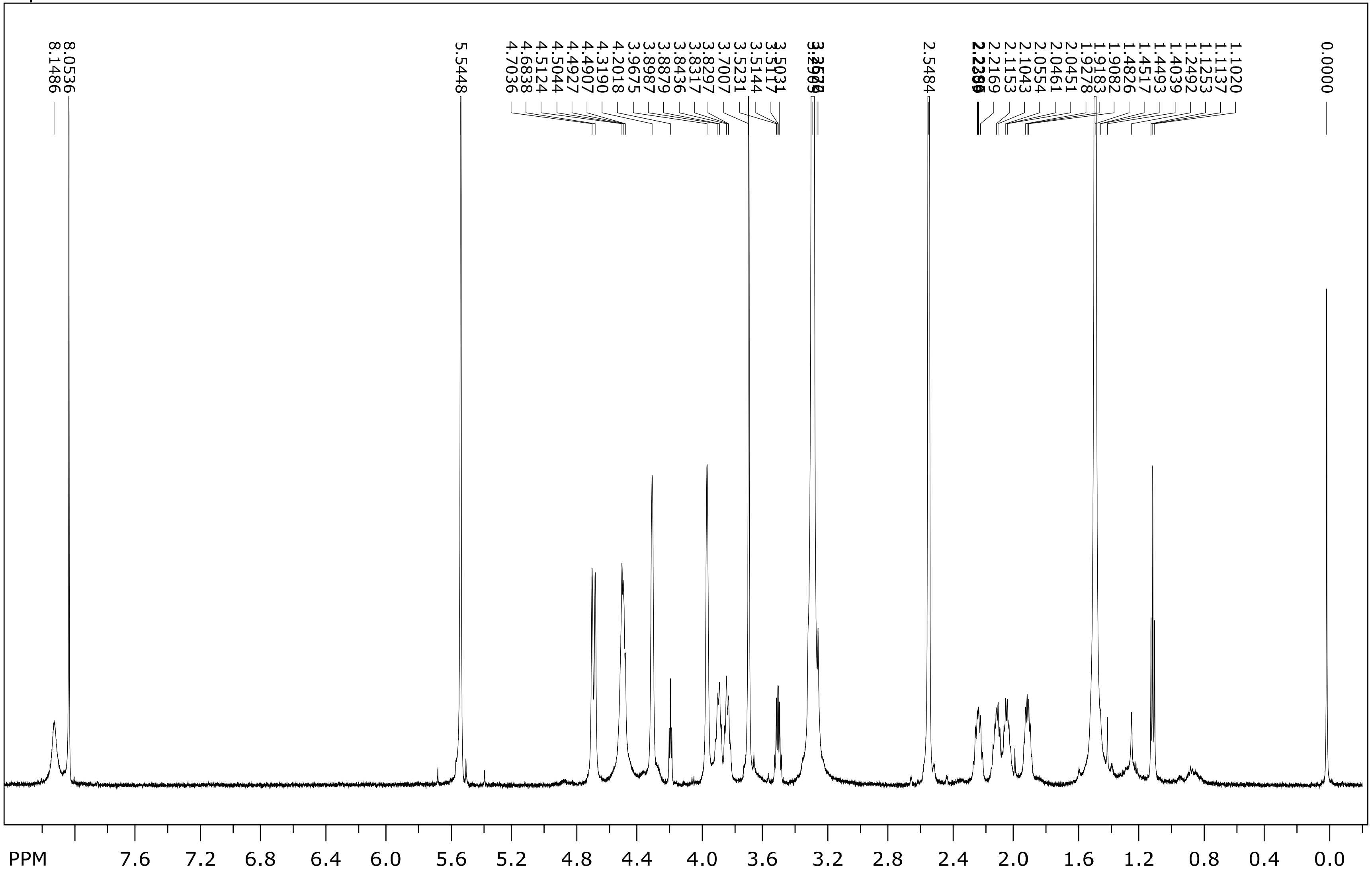

SpinWorks 3: L. Barisic 2604 25 mM CDCl3 + 630 uL DMSO

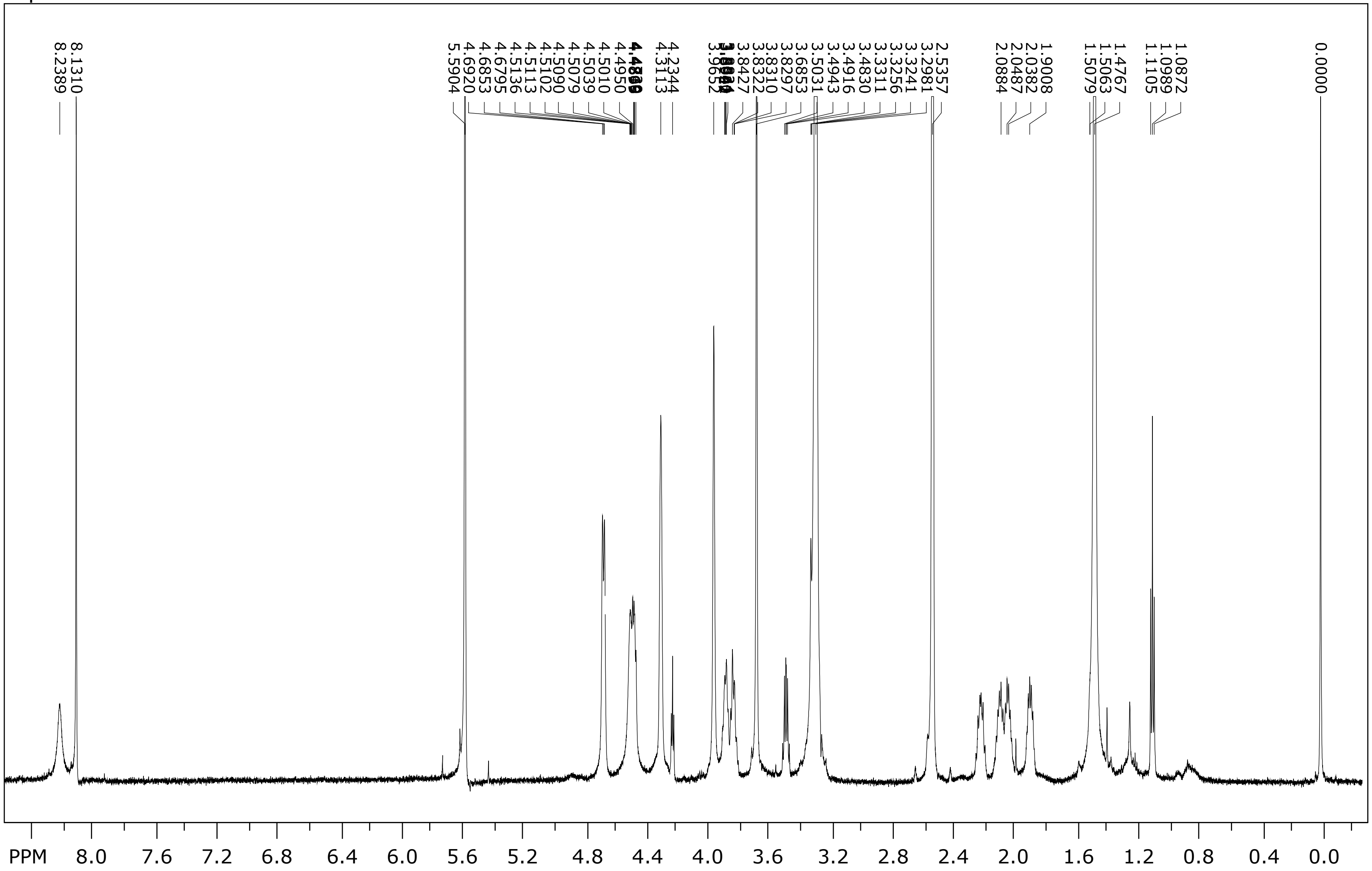

Figure S10. Variable-temperature ^{1}H NMR spectra of **2**.

SpinWorks 3: L. Barisic 2604 55 �C

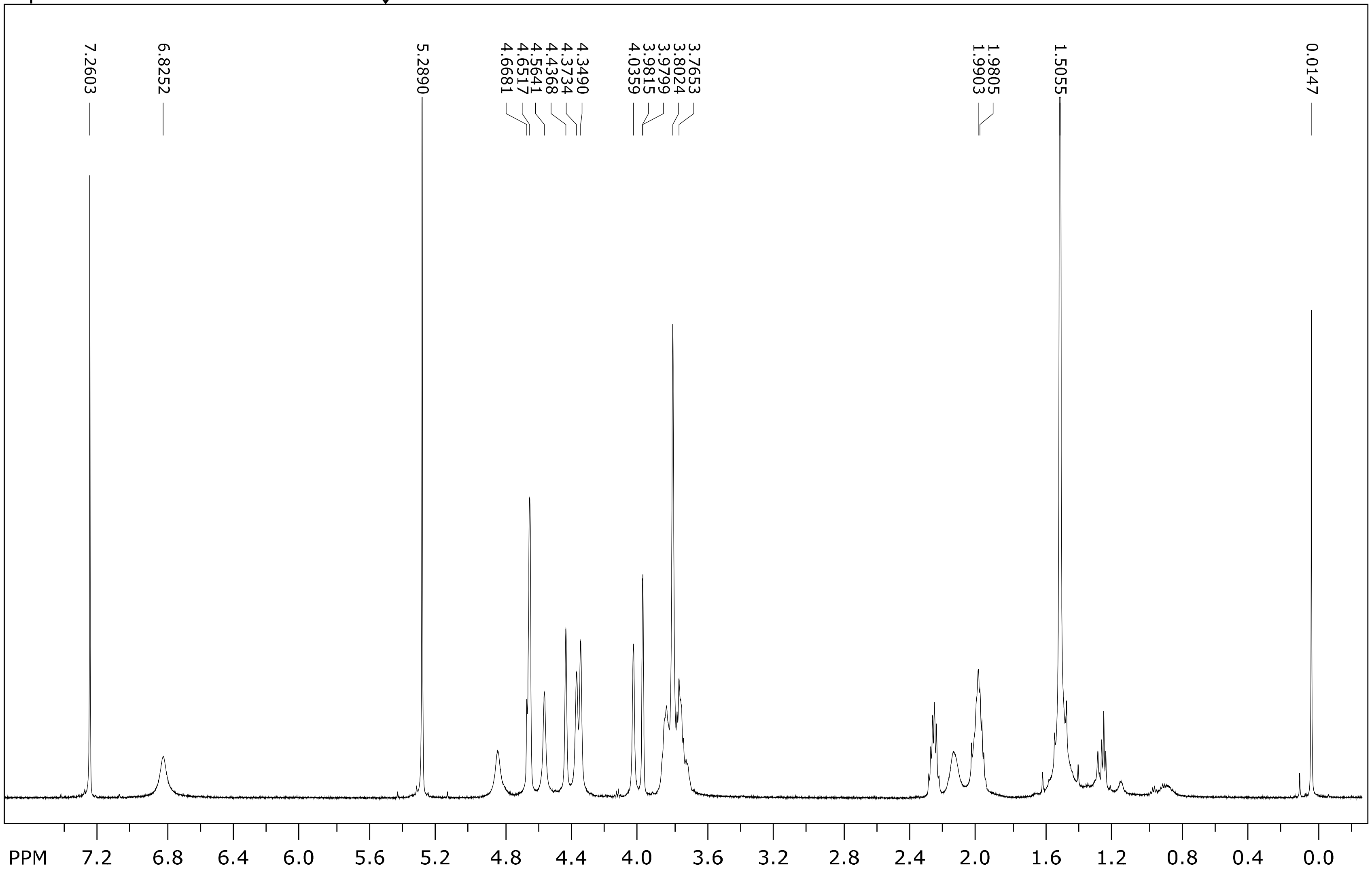

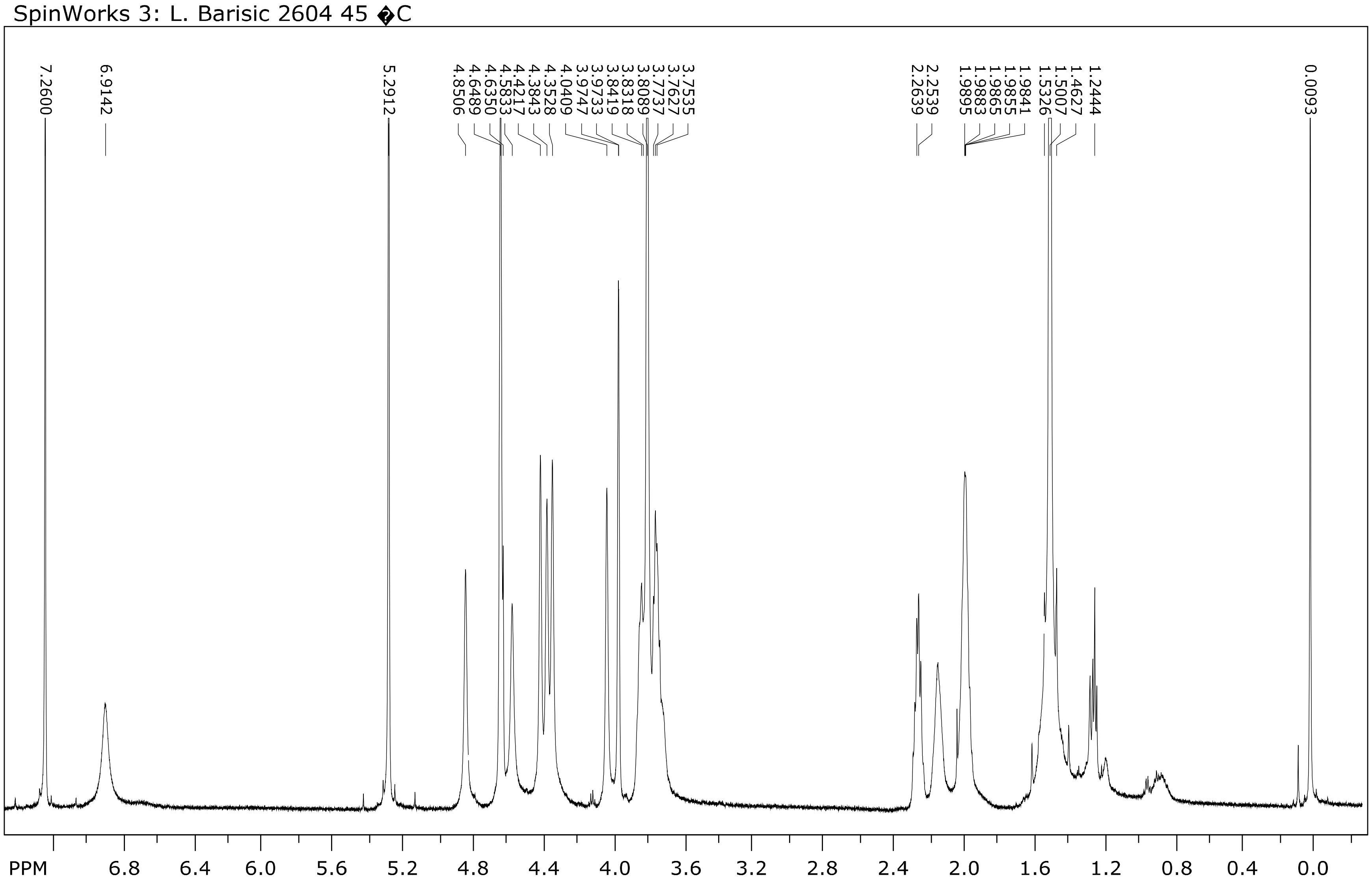
SpinWorks 3: L. Barisic 2604 45 �C
7.2600
6.9142
5.2912
4.8506
4.6489
4.6350
4.5833
4.4217
4.3843
4.3528
4.0409
3.9747
3.9733
3.8419
3.8318
3.8089
3.7737
3.7627
3.7535
2.2639
2.2539
1.9895
1.9883
1.9865
1.9855
1.9841
1.5326
1.5007
1.4627
1.2444
0.0093
PPM
6.8
6.4
6.0
5.6
5.2
4.8
4.4
4.0
3.6
3.2
2.8
2.4
2.0
1.6
1.2
0.8
0.4
0.0

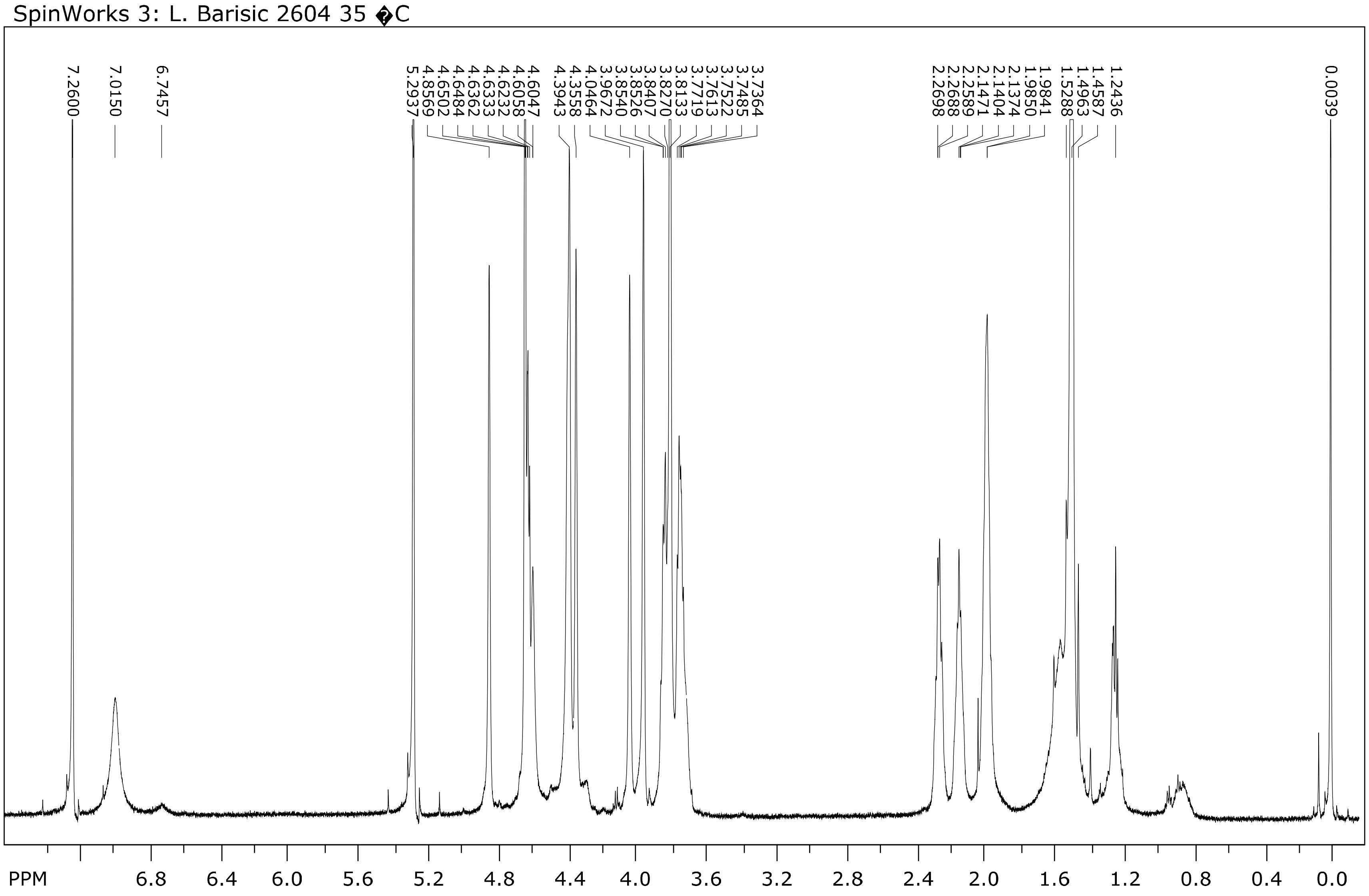
SpinWorks 3: L. Barisic 2604 35 �C
7.2600
7.0150
6.7457
5.2937
4.8569
4.6502
4.6484
4.6362
4.6333
4.6232
4.6058
4.6047
4.3943
4.3558
4.0464
3.9672
3.8540
3.8526
3.8407
3.8270
3.8133
3.7719
3.7613
3.7522
3.7485
3.7364
2.2698
2.2688
2.2589
2.1471
2.1404
2.1374
1.9850
1.9841
1.5288
1.4963
1.4587
1.2436
0.0039
PPM
6.8
6.4
6.0
5.6
5.2
4.8
4.4
4.0
3.6
3.2
2.8
2.4
2.0
1.6
1.2
0.8
0.4
0.0

SpinWorks 3: L. Barisic 2604 25 �C

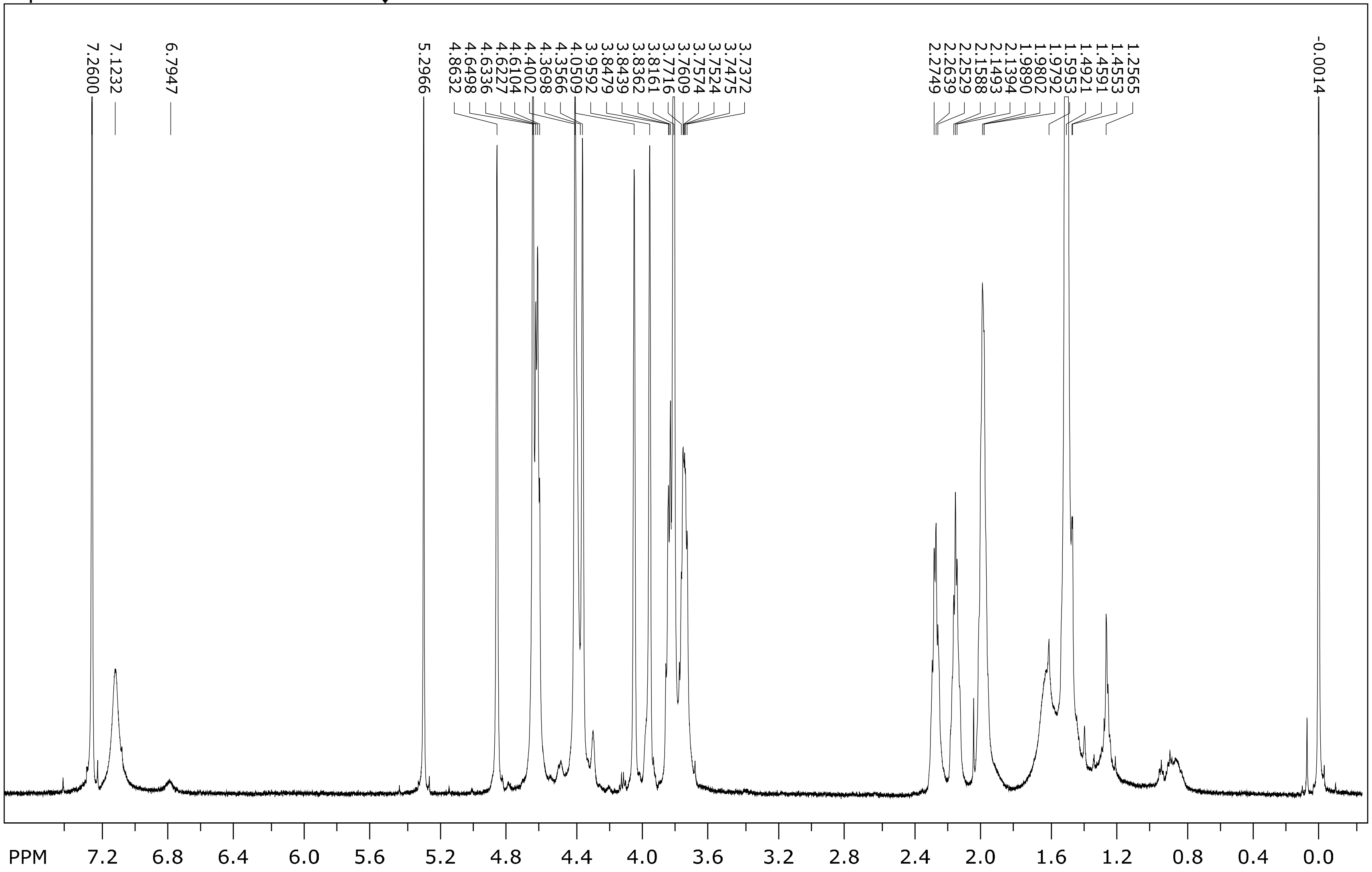

SpinWorks 3: L. Barisic 2604 15 �C

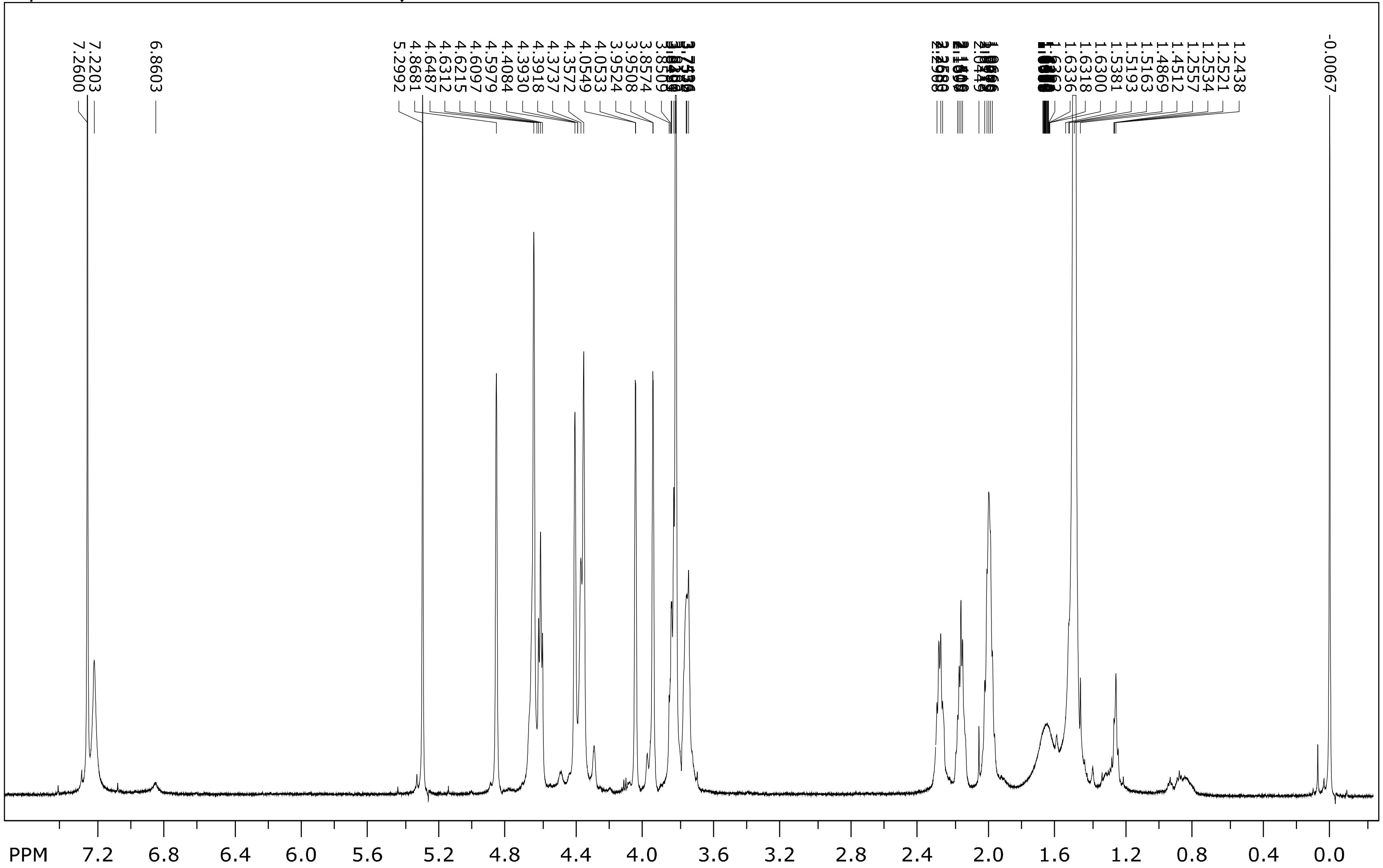

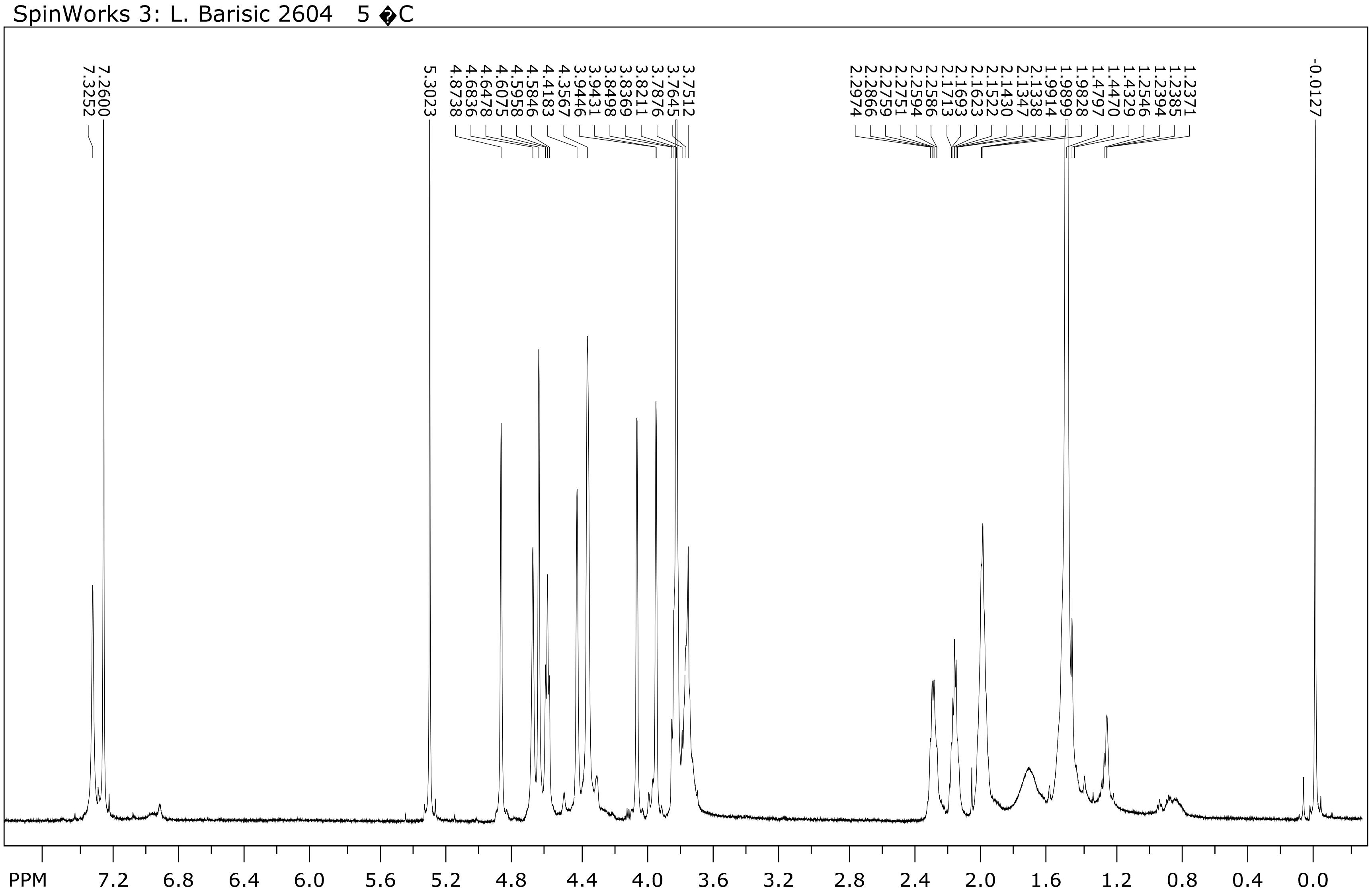
SpinWorks 3: L. Barisic 2604 5 �C
7.3252
7.2600
5.3023
4.8738
4.6836
4.6478
4.6075
4.5958
4.5846
4.4183
4.3567
3.9446
3.9431
3.8498
3.8369
3.8211
3.7876
3.7645
3.7512
2.2974
2.2866
2.2759
2.2751
2.2594
2.2586
2.1713
2.1693
2.1623
2.1522
2.1430
2.1347
2.1338
1.9914
1.9899
1.9828
1.4797
1.4470
1.4329
1.2546
1.2394
1.2385
1.2371
-0.0127
PPM
7.2
6.8
6.4
6.0
5.6
5.2
4.8
4.4
4.0
3.6
3.2
2.8
2.4
2.0
1.6
1.2
0.8
0.4
0.0

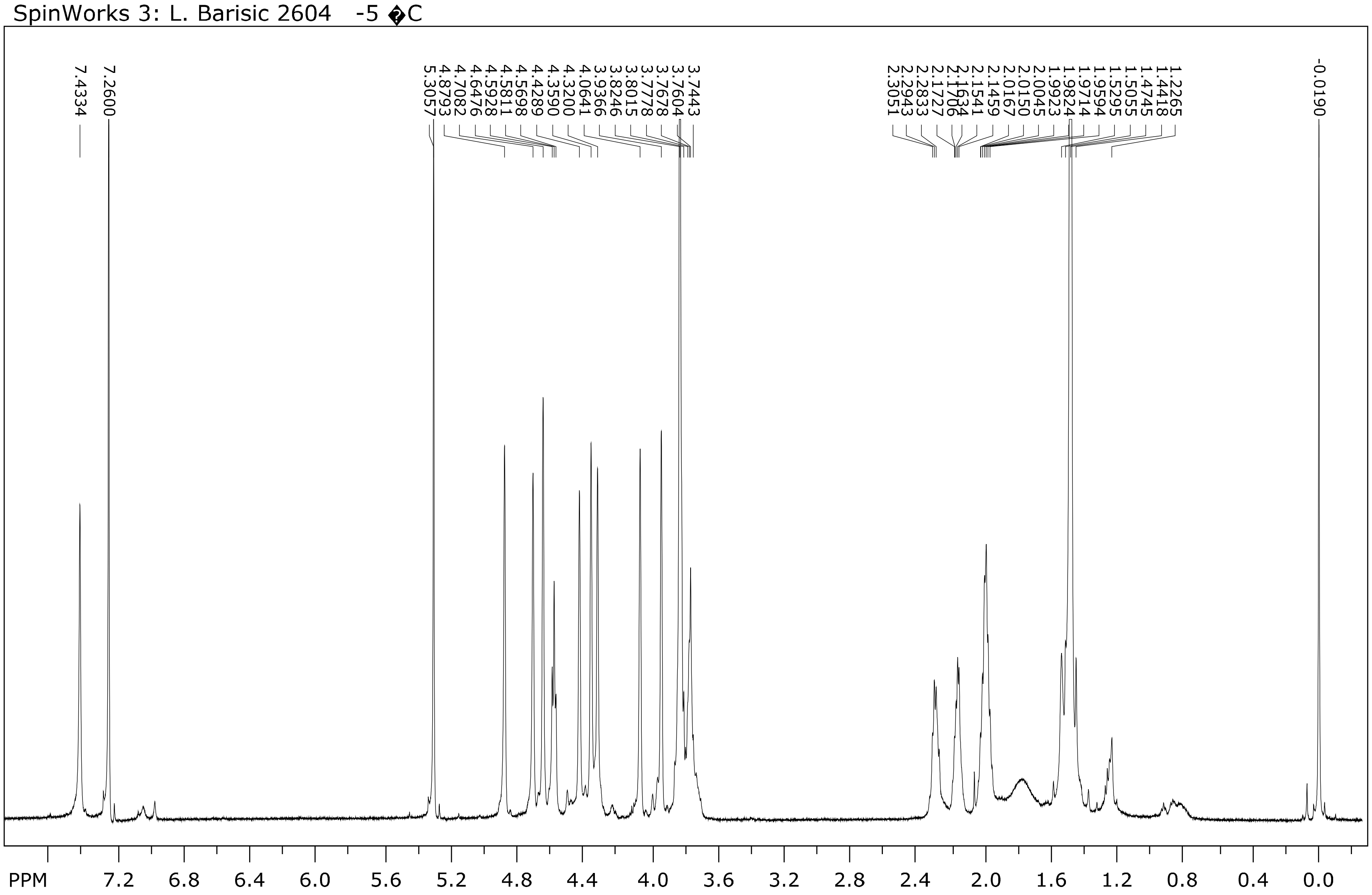
SpinWorks 3: L. Barisic 2604 -5 �C
7.4334
7.2600
5.3057
4.8793
4.7082
4.6476
4.5928
4.5811
4.5698
4.4289
4.3590
4.3200
4.0641
3.9366
3.8246
3.8015
3.7778
3.7678
3.7604
3.7443
2.3051
2.2943
2.2833
2.1727
2.1706
2.1634
2.1541
2.1459
2.0167
2.0150
2.0045
1.9923
1.9824
1.9714
1.9594
1.5295
1.5055
1.4745
1.4418
1.2265
-0.0190
PPM
7.2
6.8
6.4
6.0
5.6
5.2
4.8
4.4
4.0
3.6
3.2
2.8
2.4
2.0
1.6
1.2
0.8
0.4
0.0

SpinWorks 3: L. Barisic 2604 -15 �C

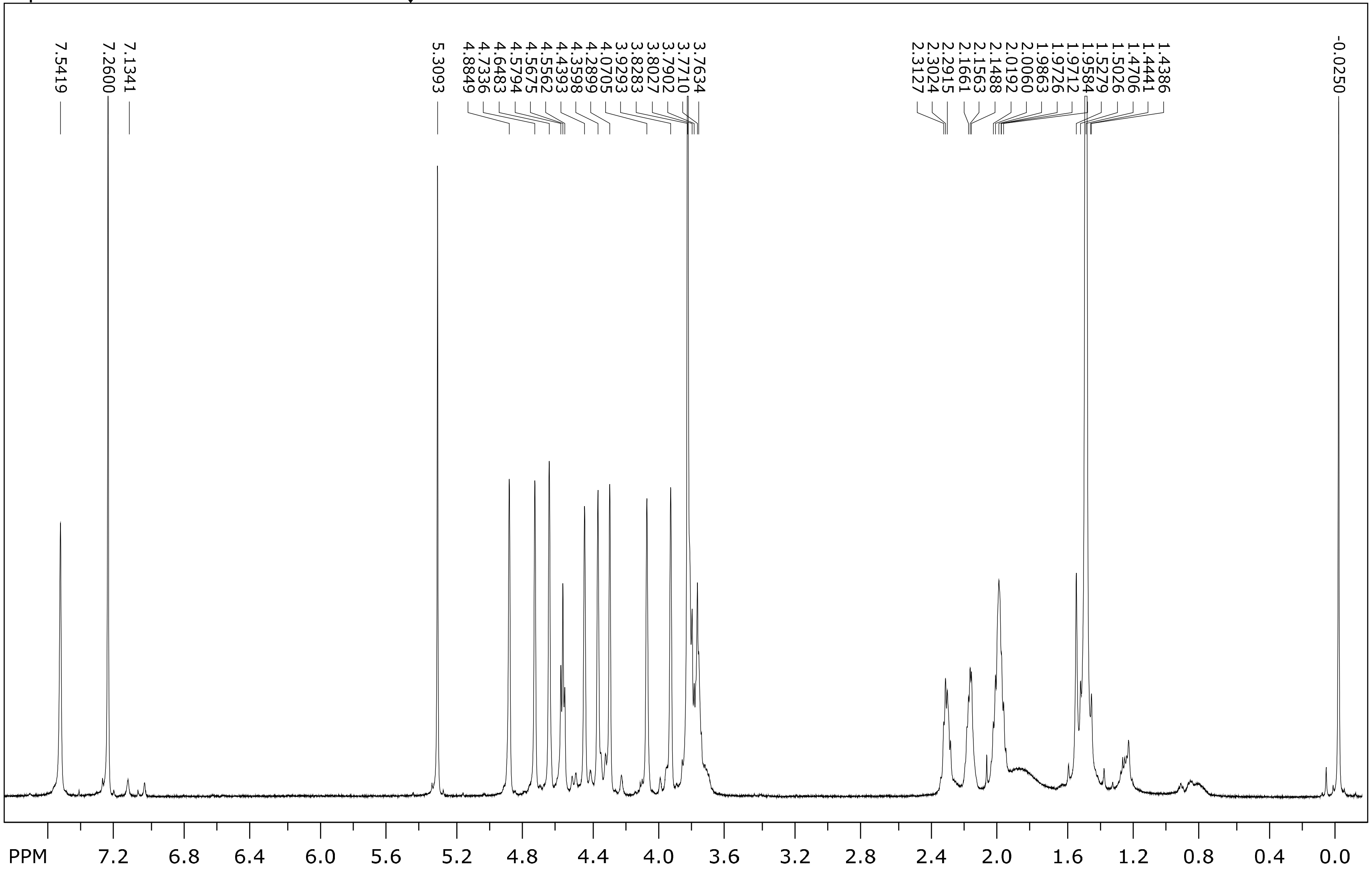

Compound 3

Figure S11. MALDI TOF/TOF-MS spectrum of **3**.

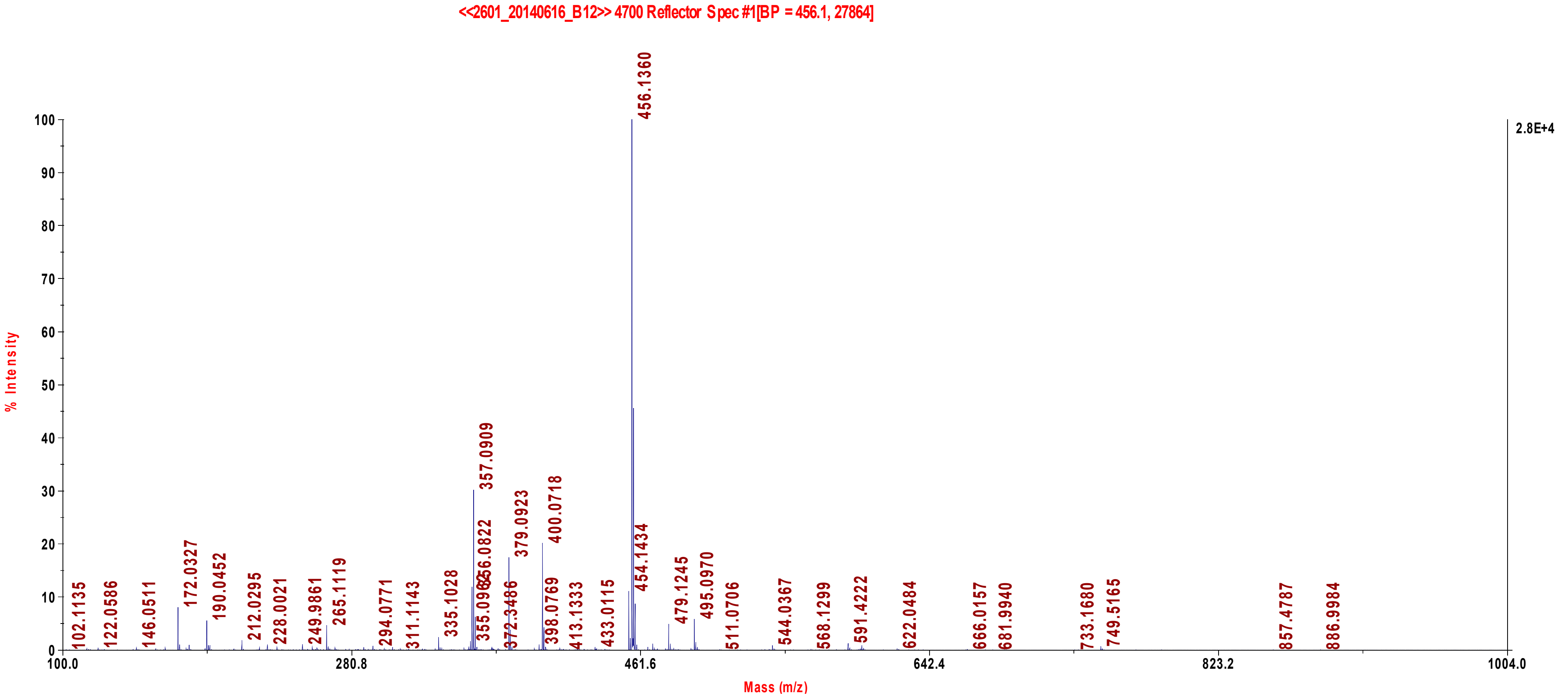

Figure S12. ¹H NMR spectra of **3**.

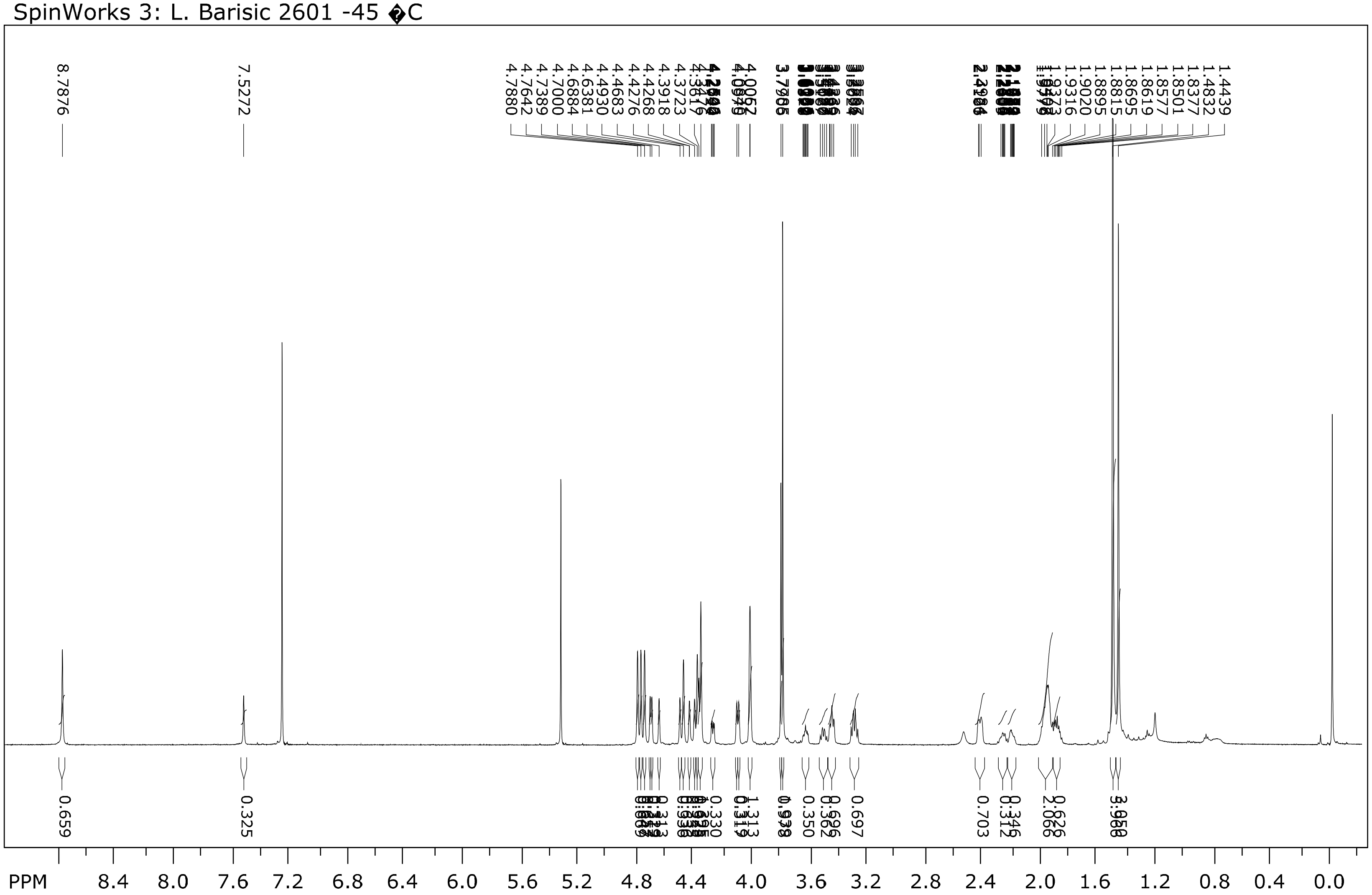

SpinWorks 3: L. Barisic 2601 -45 �C

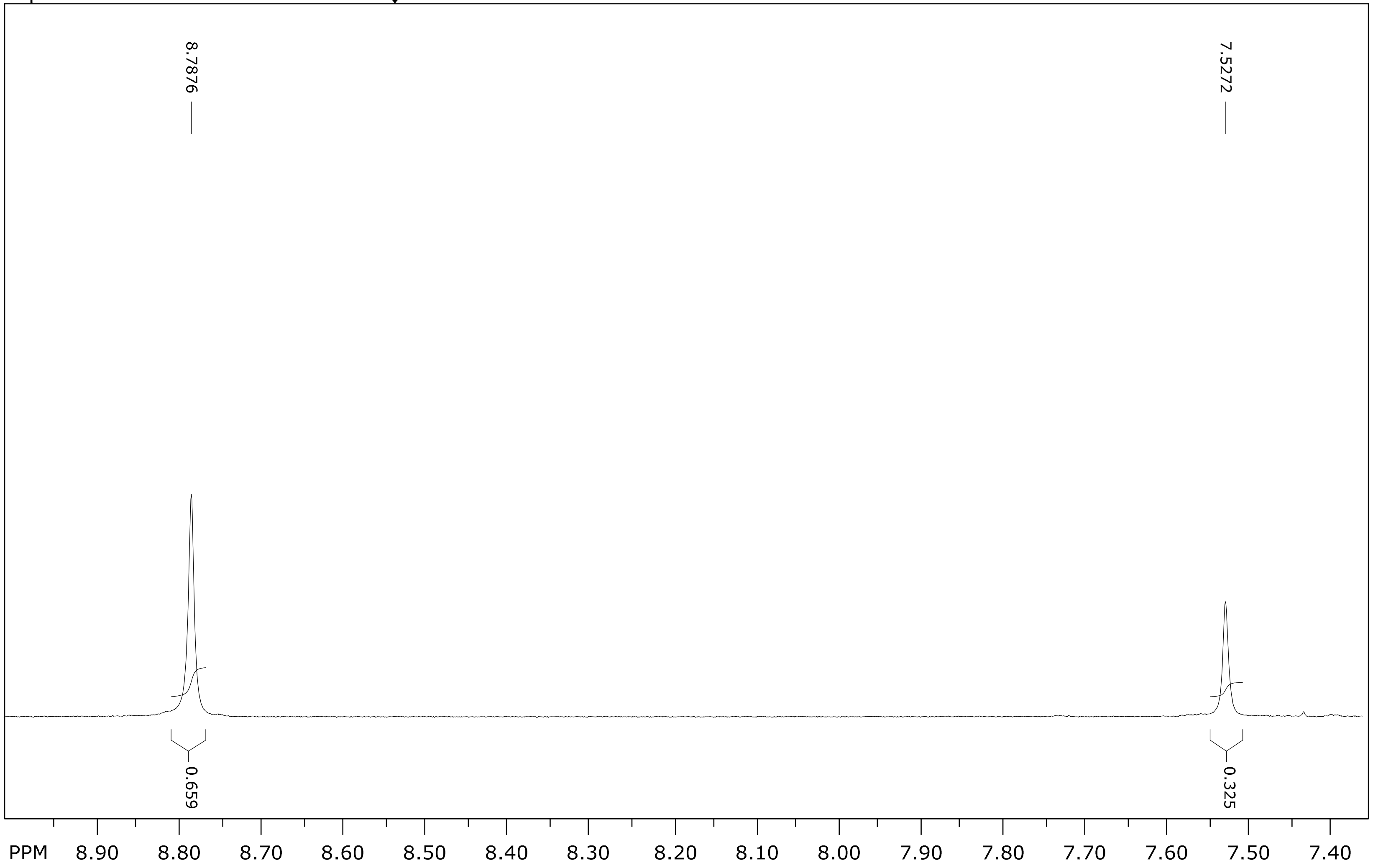

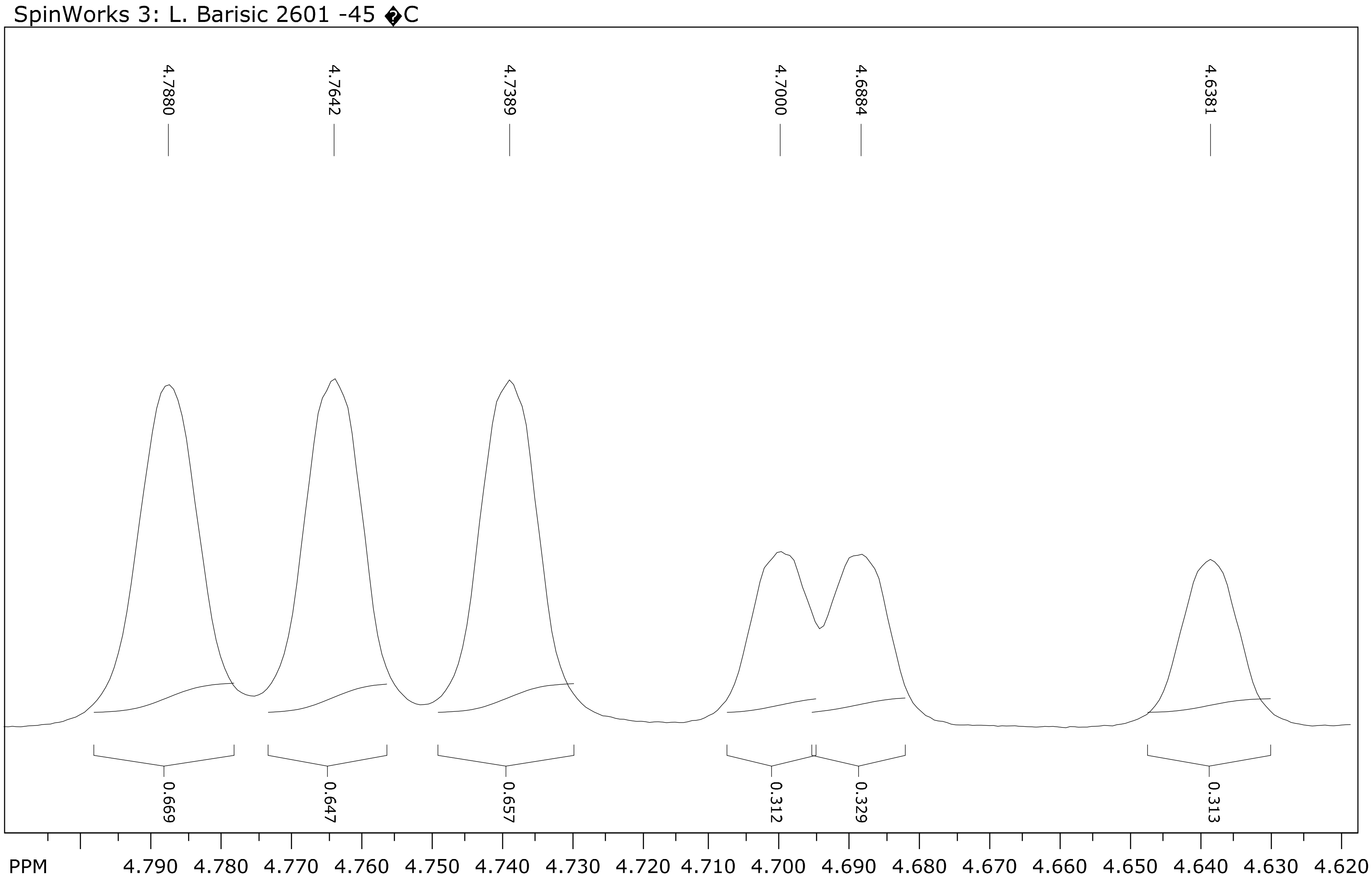
SpinWorks 3: L. Barisic 2601 -45 �C
4.7880
4.7642
4.7389
4.7000
4.6884
4.6381
0.669
0.647
0.657
0.312
0.329
0.313
PPM
4.790 4.780 4.770 4.760 4.750 4.740 4.730 4.720 4.710 4.700 4.690 4.680 4.670 4.660 4.650 4.640 4.630 4.620

SpinWorks 3: L. Barisic 2601 -45 �C

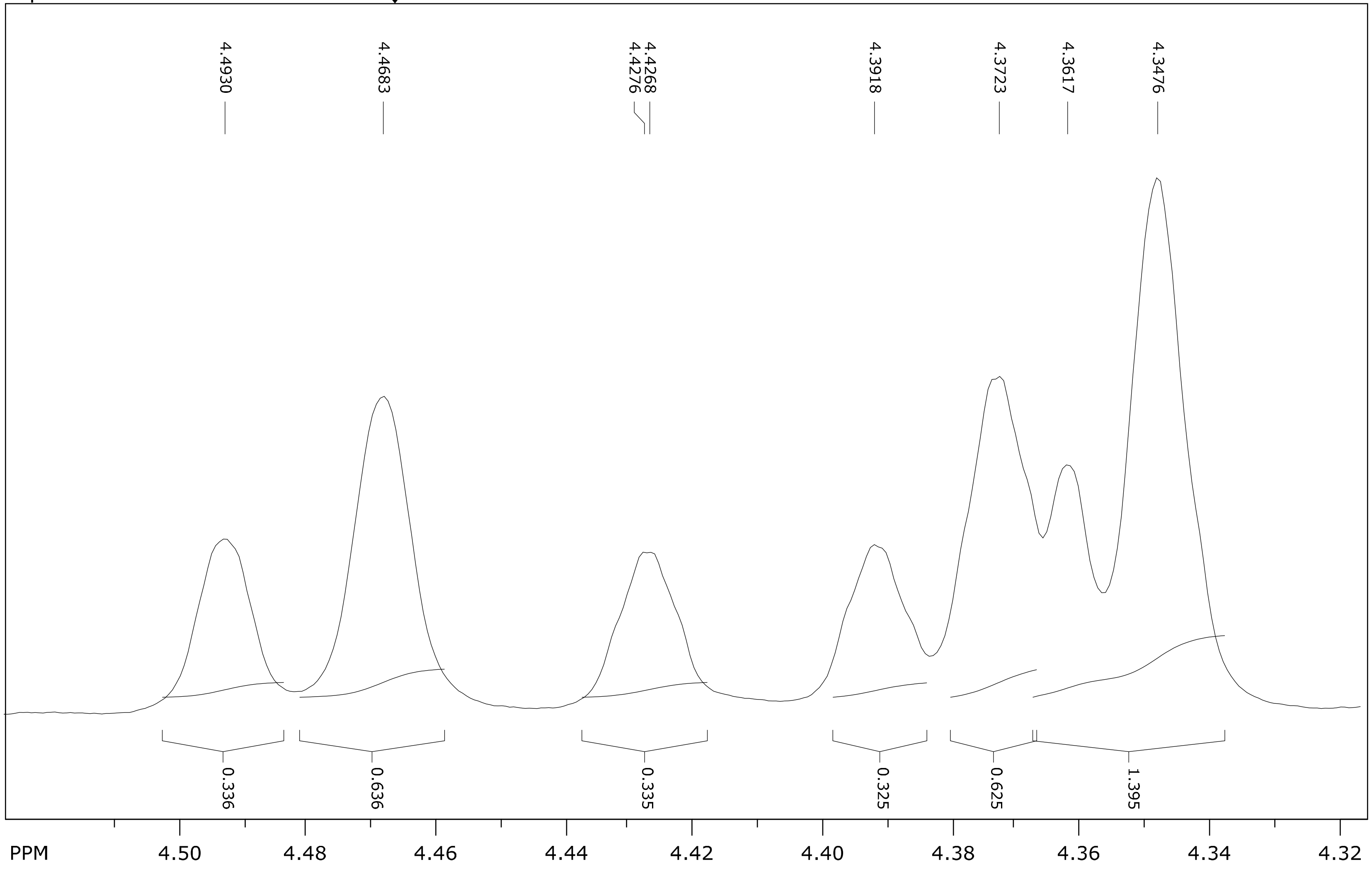

SpinWorks 3: L. Barisic 2601 -45 �C

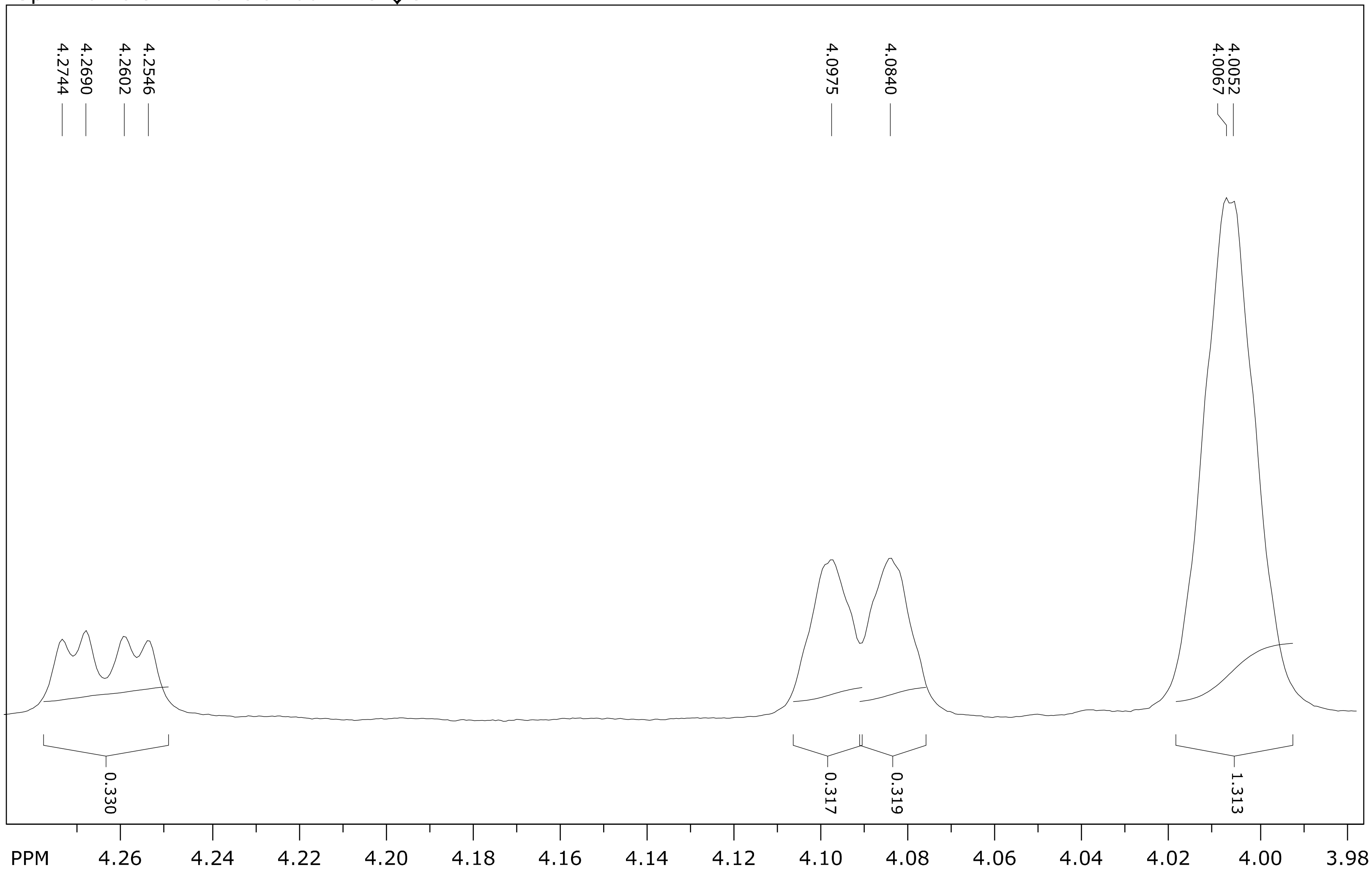

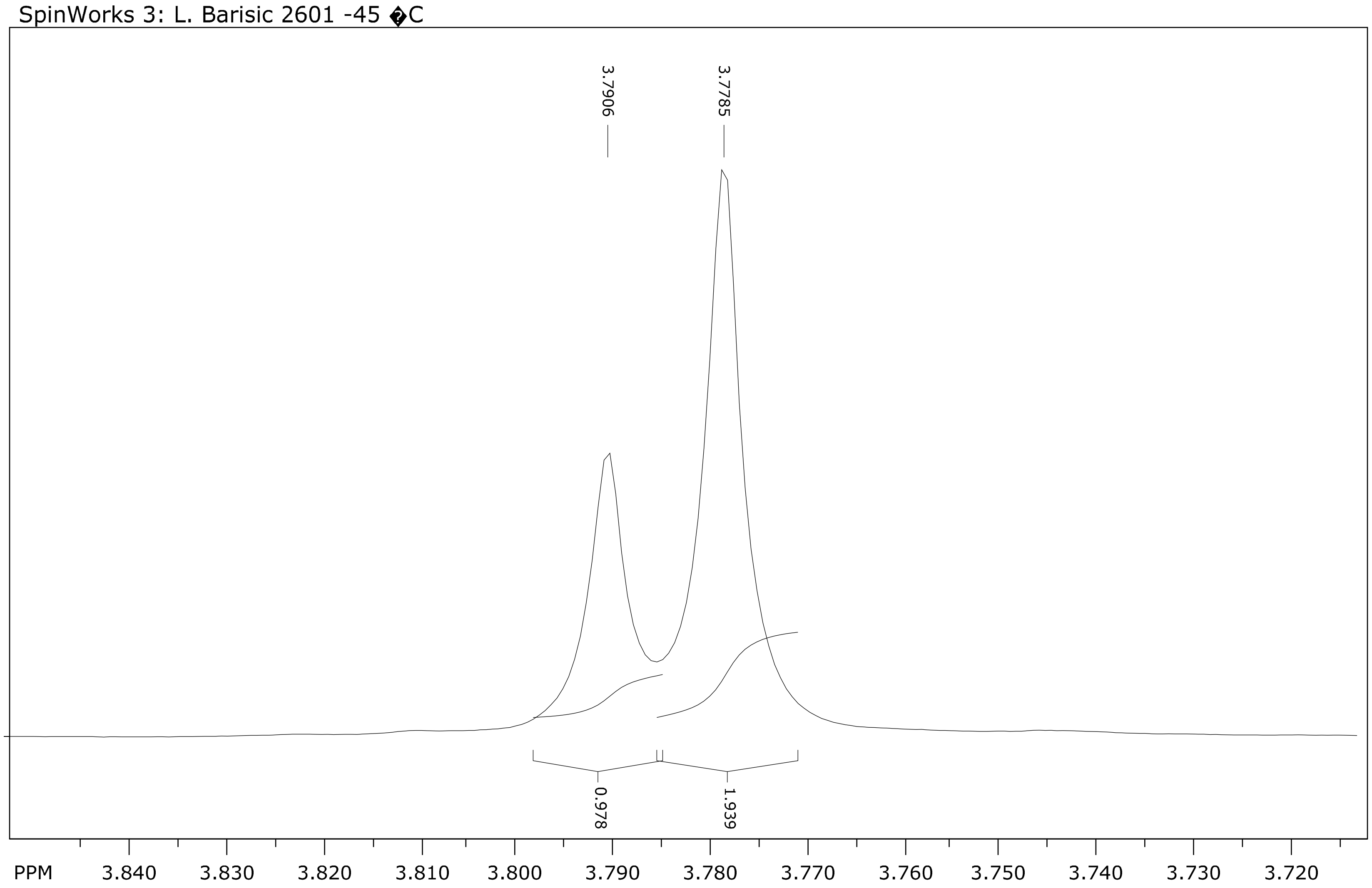
SpinWorks 3: L. Barisic 2601 -45 �C
3.7906
3.7785
0.978
1.939
PPM
3.840
3.830
3.820
3.810
3.800
3.790
3.780
3.770
3.760
3.750
3.740
3.730
3.720

SpinWorks 3: L. Barisic 2601 -45 �C

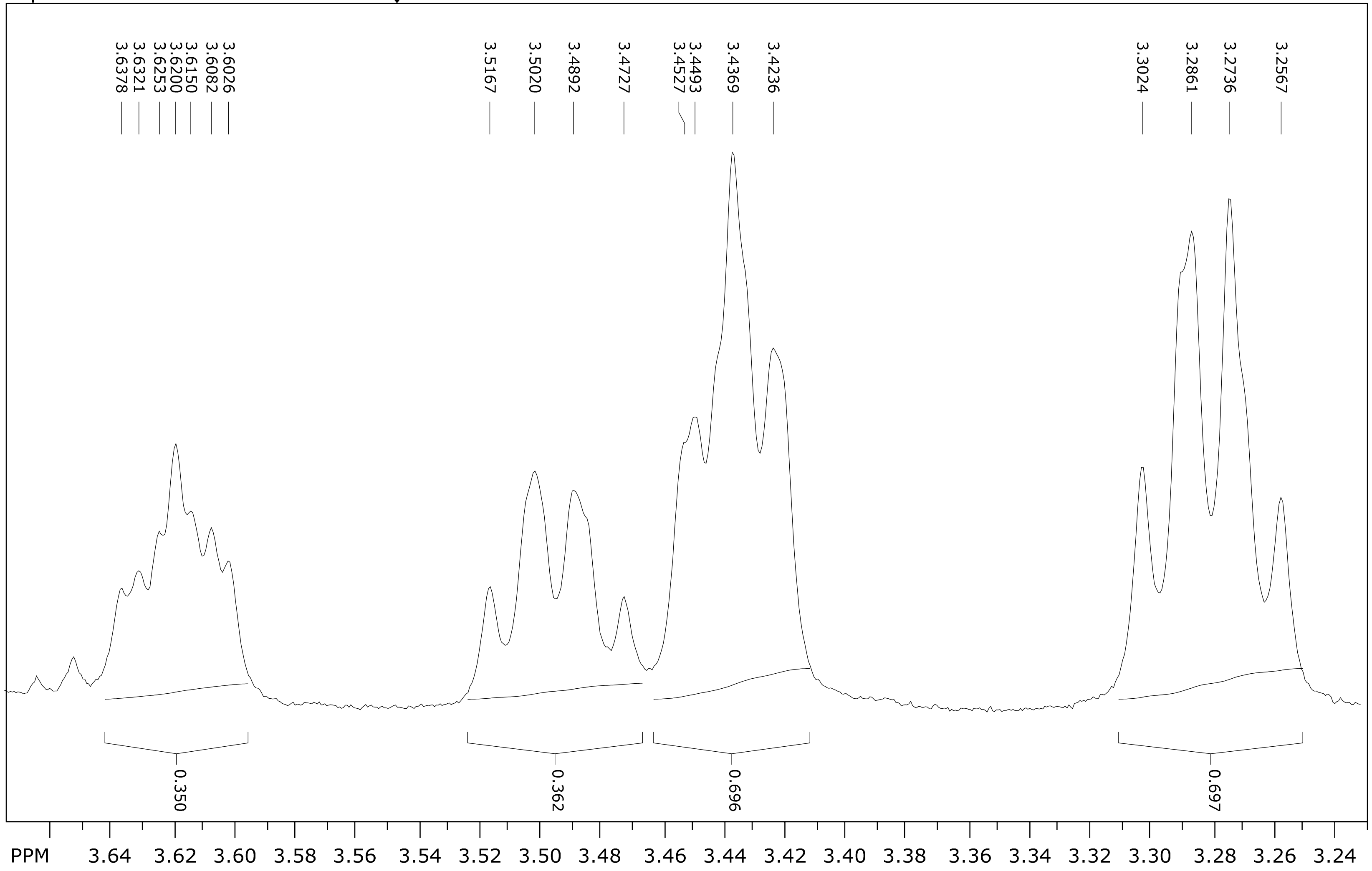

SpinWorks 3: L. Barisic 2601 -45 �C

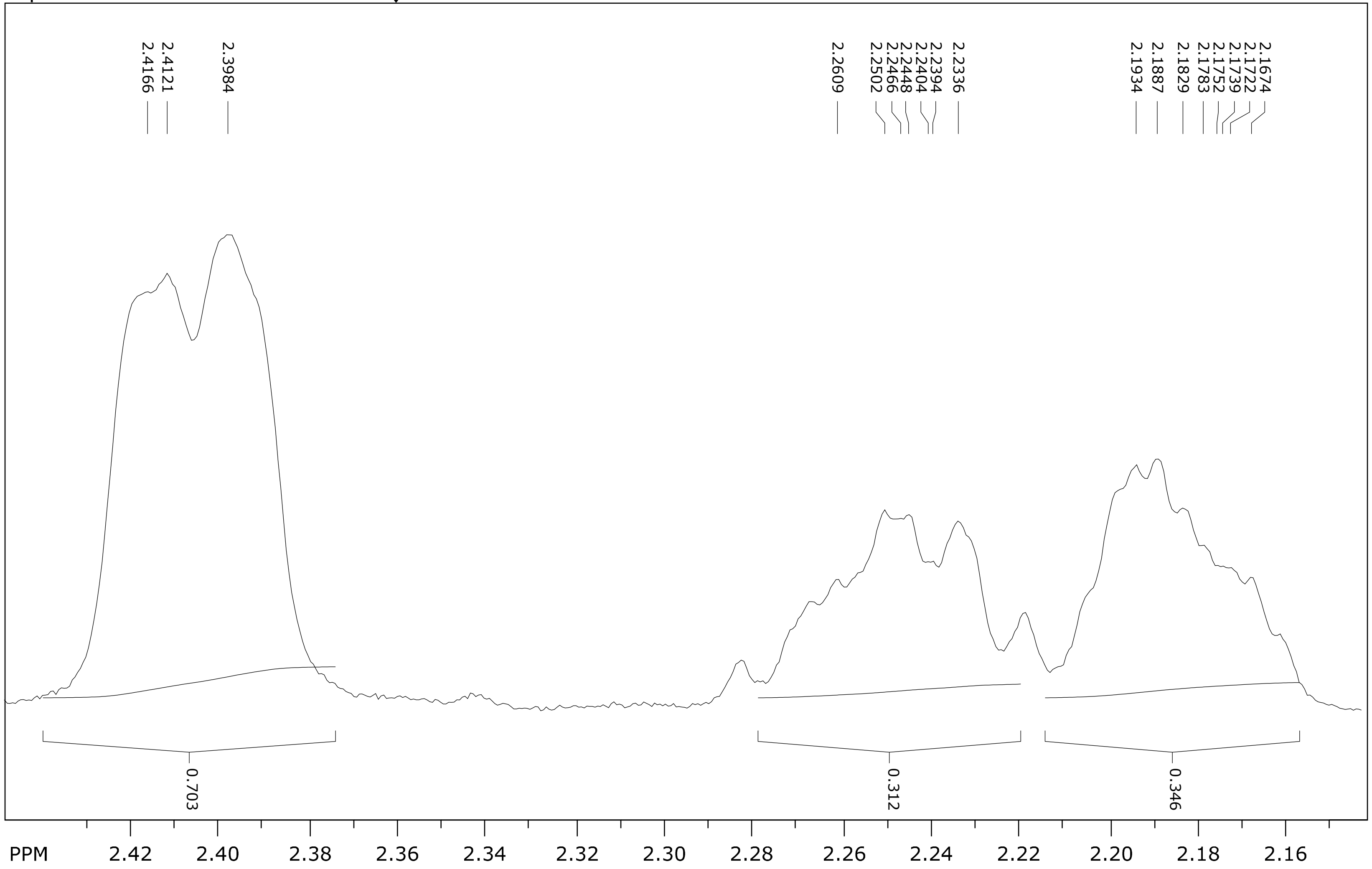

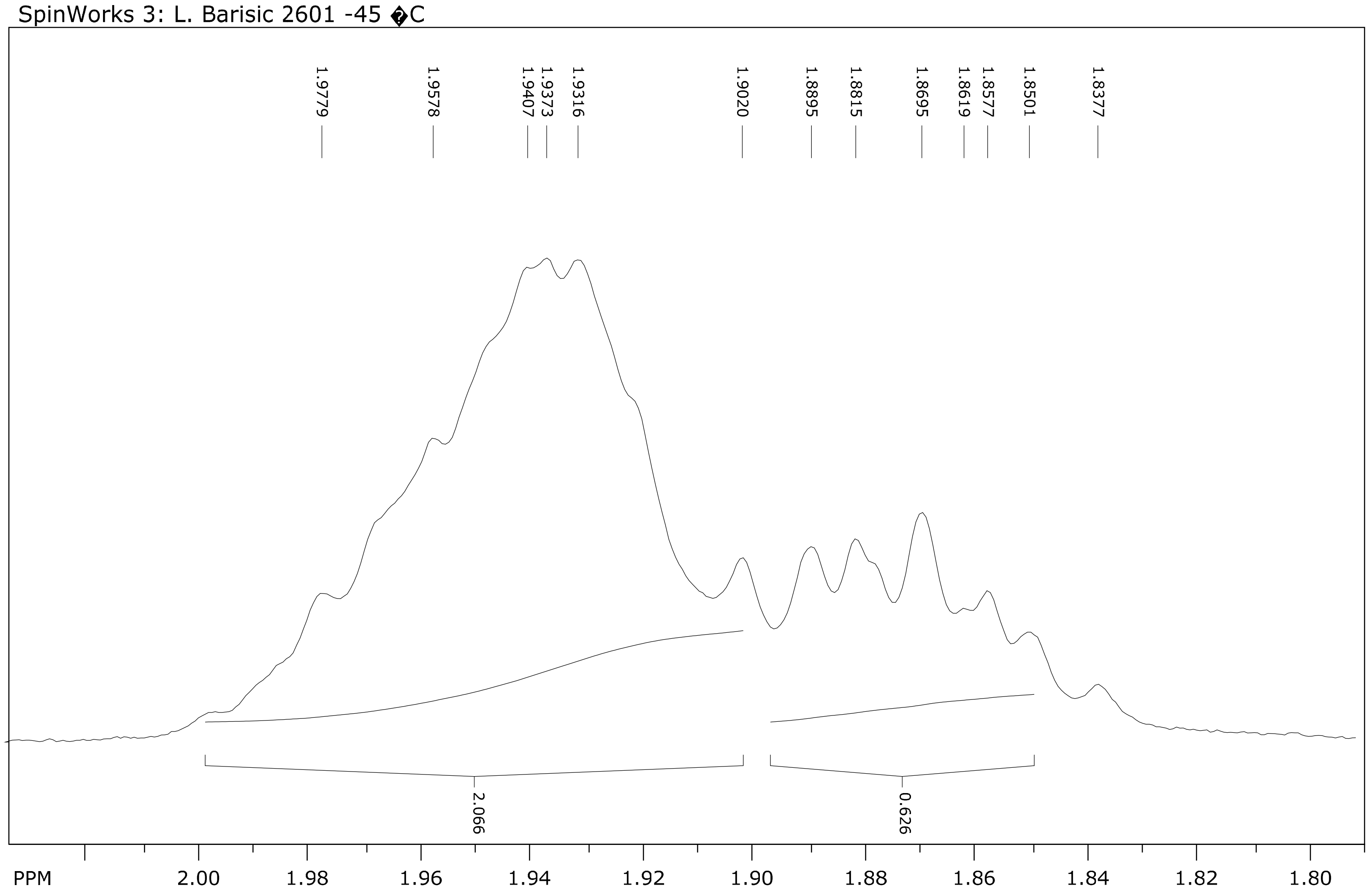
SpinWorks 3: L. Barisic 2601 -45 �C
1.9779
1.9578
1.9407
1.9373
1.9316
1.9020
1.8895
1.8815
1.8695
1.8619
1.8577
1.8501
1.8377
2.066
0.626
PPM
2.00
1.98
1.96
1.94
1.92
1.90
1.88
1.86
1.84
1.82
1.80

SpinWorks 3: L. Barisic 2601 -45 �C

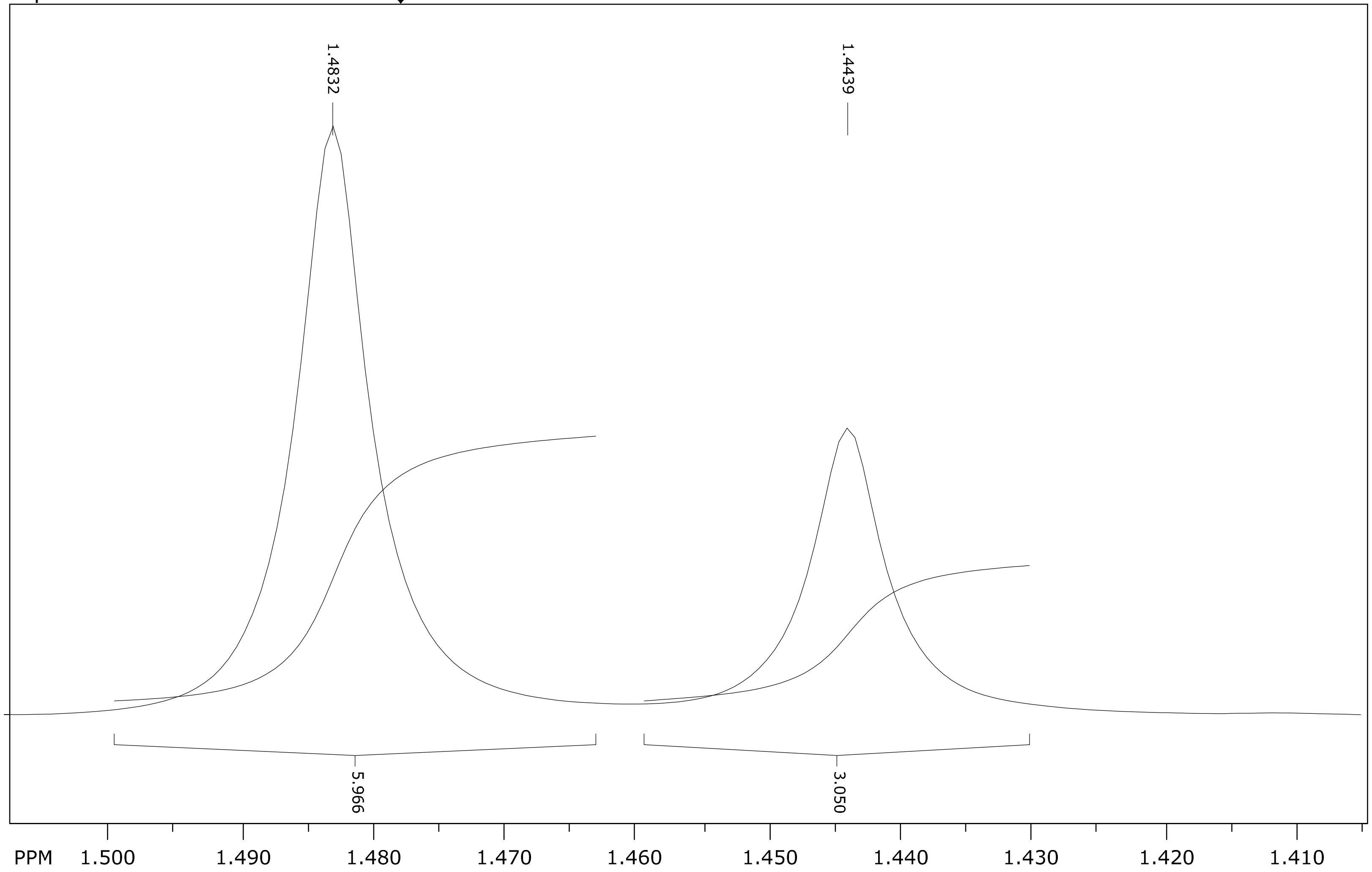

Figure S13. ^{13}C NMR spectra of **3**.

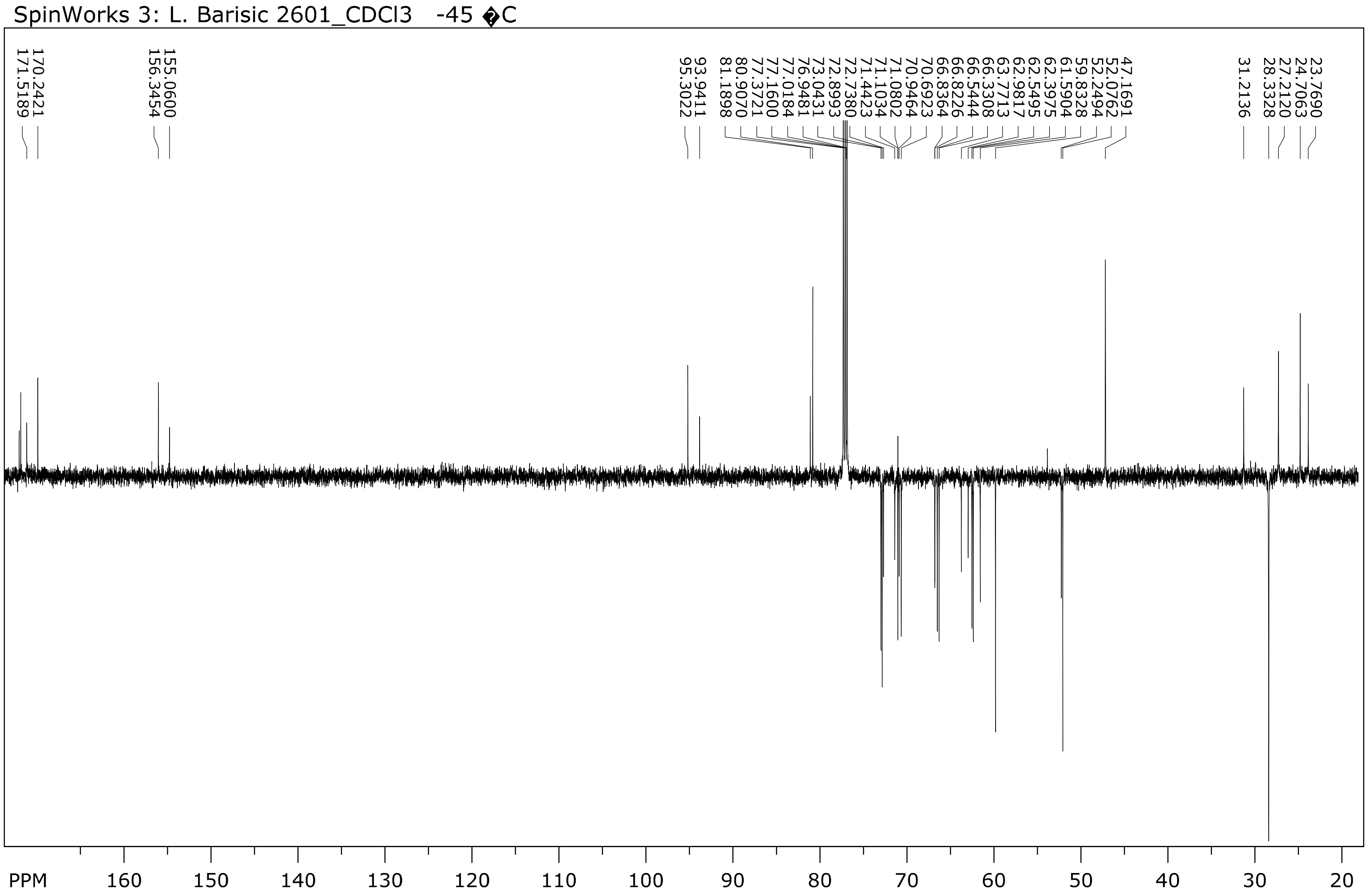

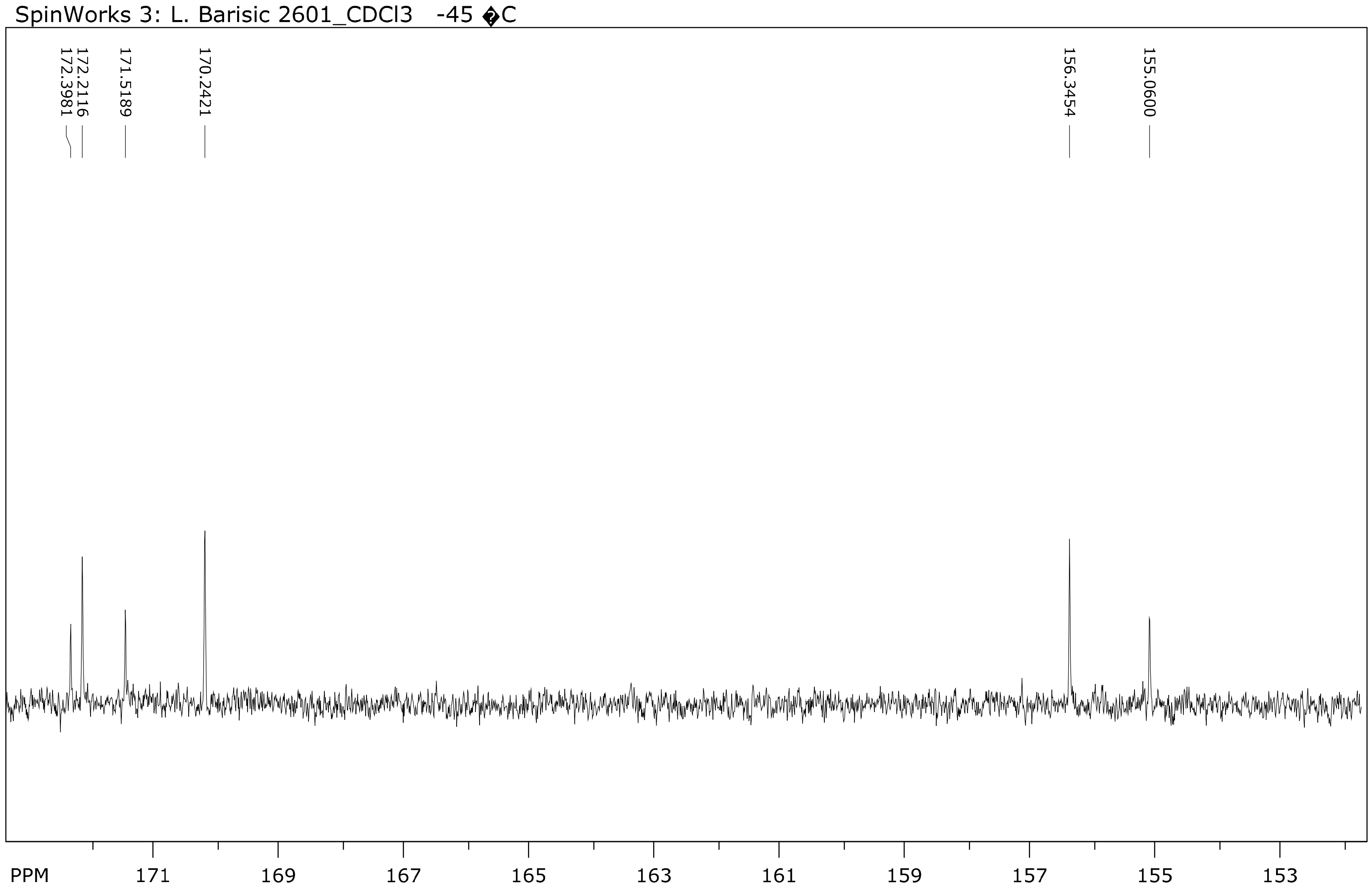
SpinWorks 3: L. Barisic 2601_CDCl3 -45 �C
172.3981
172.2116
171.5189
170.2421
156.3454
155.0600
PPM
171
169
167
165
163
161
159
157
155
153

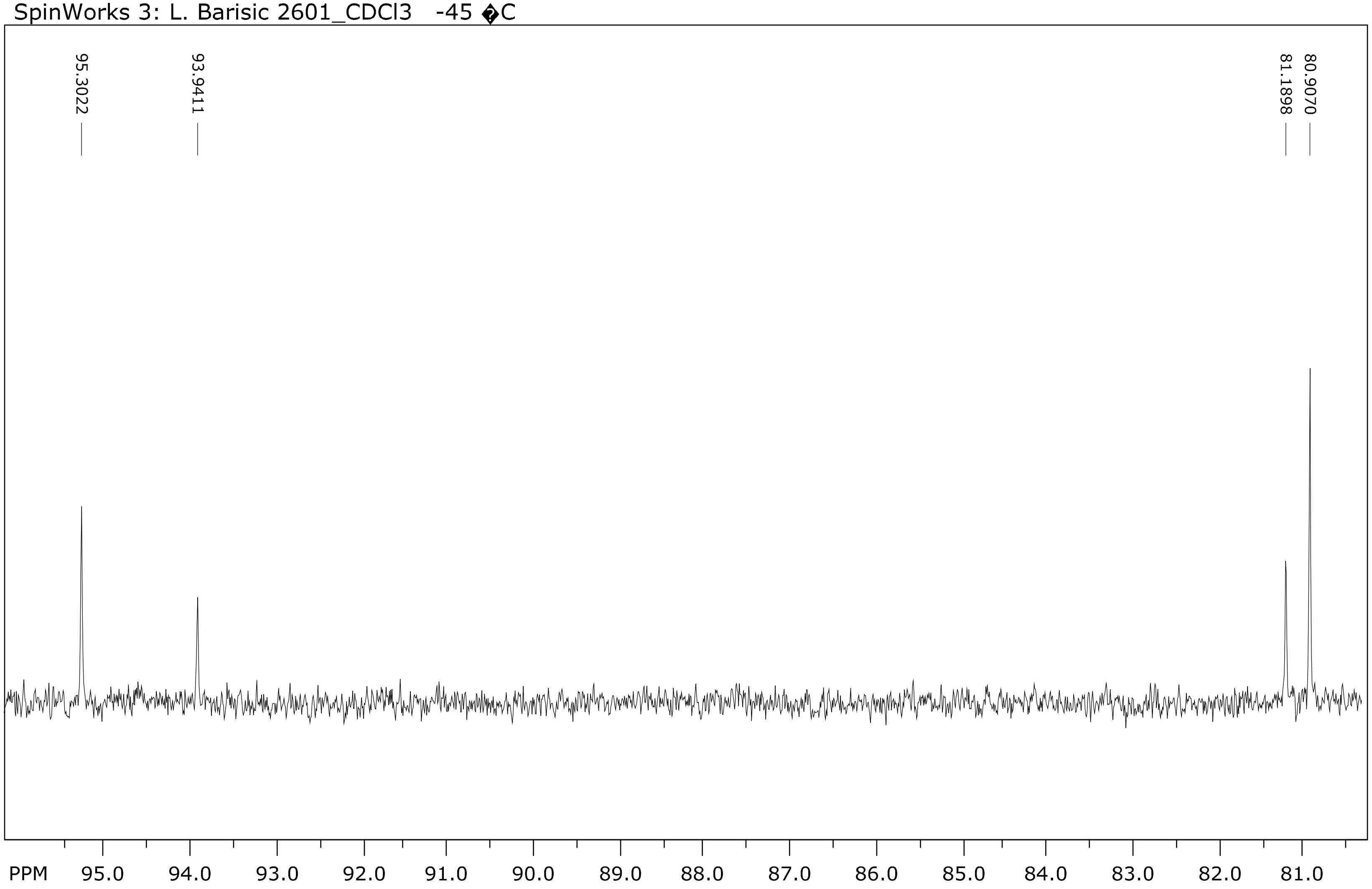
SpinWorks 3: L. Barisic 2601_CDCl3 -45 �C
95.3022
93.9411
81.1898
80.9070
PPM
95.0
94.0
93.0
92.0
91.0
90.0
89.0
88.0
87.0
86.0
85.0
84.0
83.0
82.0
81.0

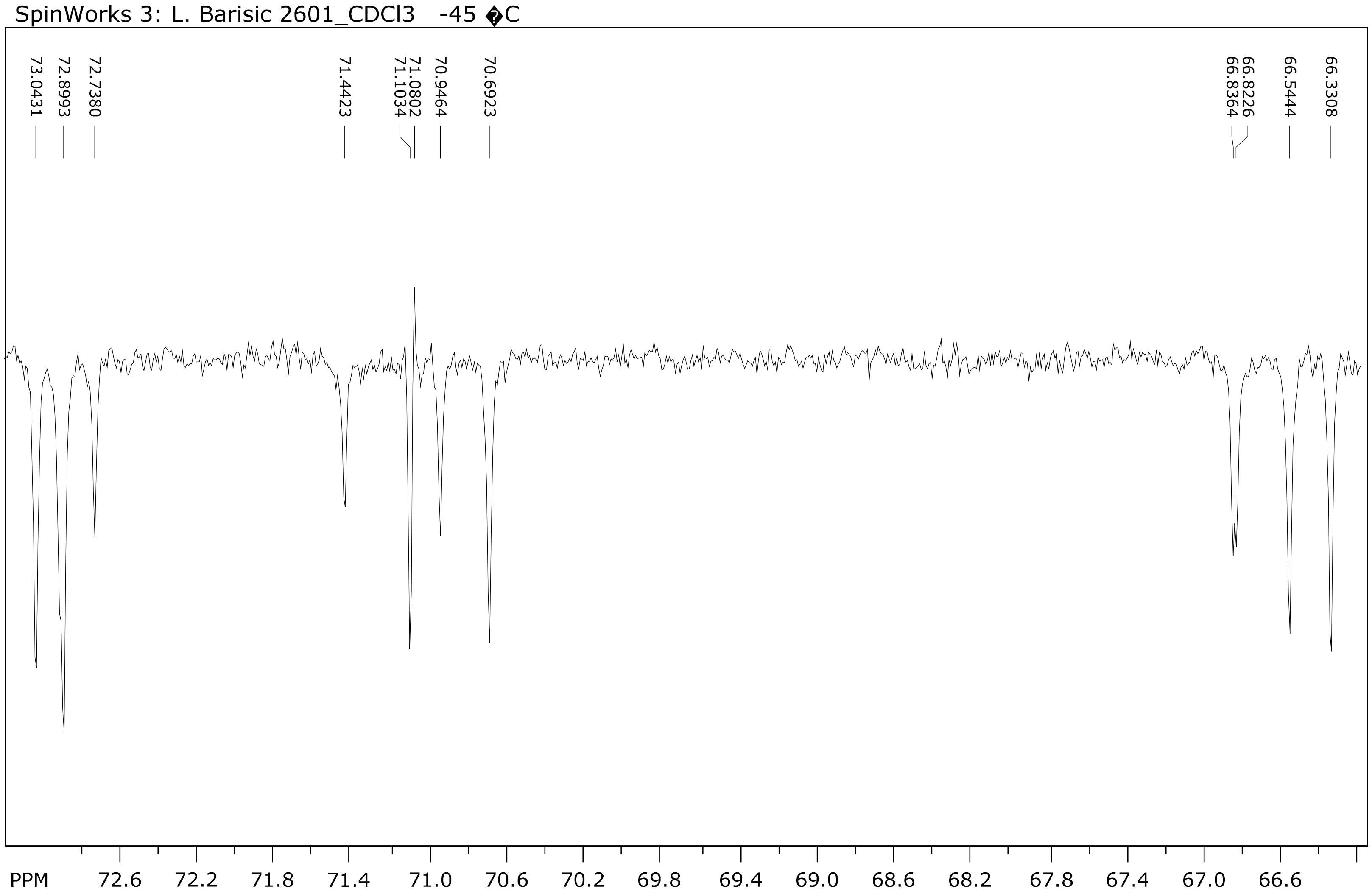
SpinWorks 3: L. Barisic 2601_CDCl3 -45 �C
73.0431
72.8993
72.7380
71.4423
71.0802
71.1034
70.9464
70.6923
66.8226
66.8364
66.5444
66.3308
PPM
72.6
72.2
71.8
71.4
71.0
70.6
70.2
69.8
69.4
69.0
68.6
68.2
67.8
67.4
67.0
66.6

SpinWorks 3: L. Barisic 2601_CDCl3 -45 �C

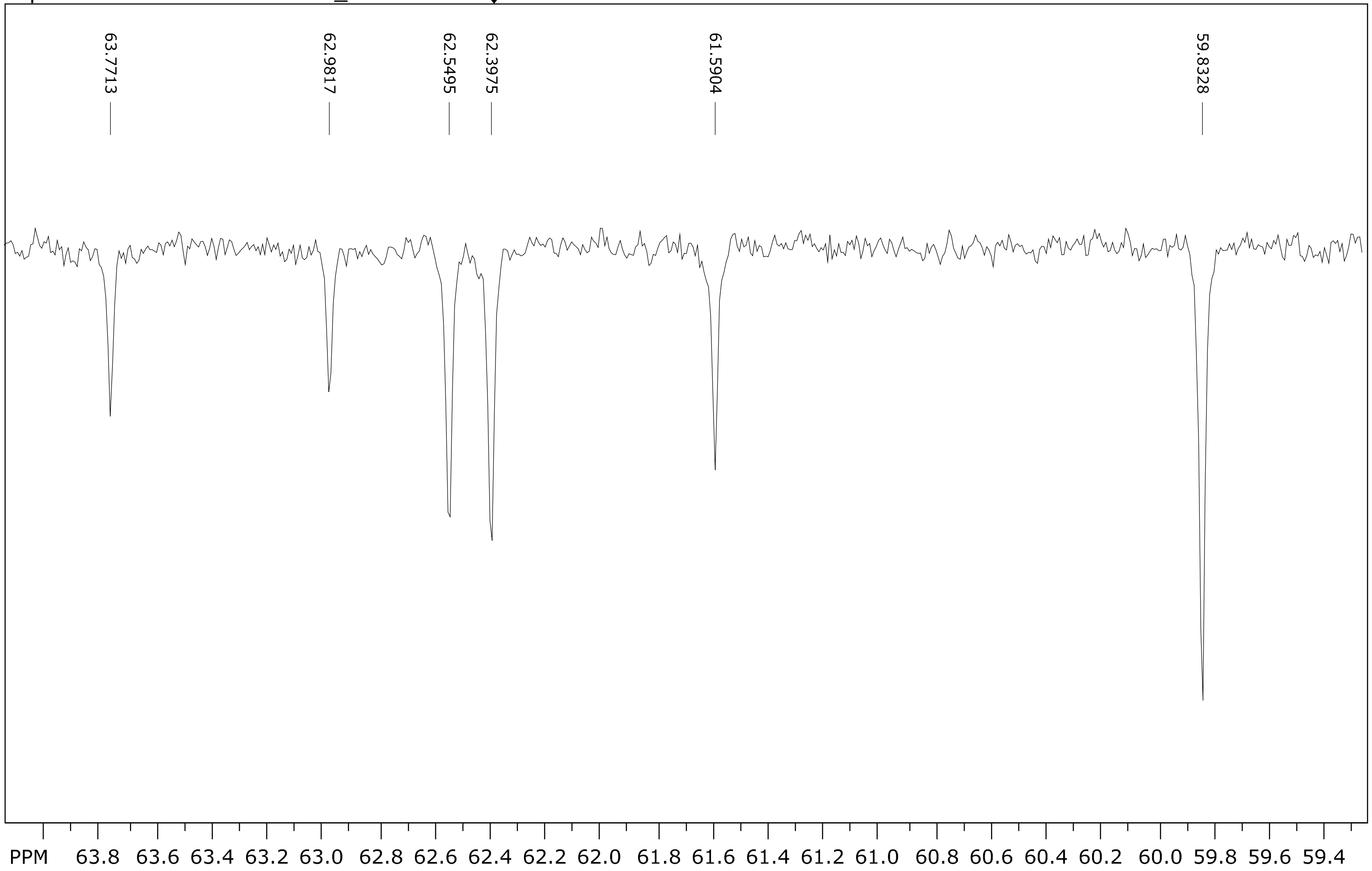

SpinWorks 3: L. Barisic 2601_CDCl3 -45 �C

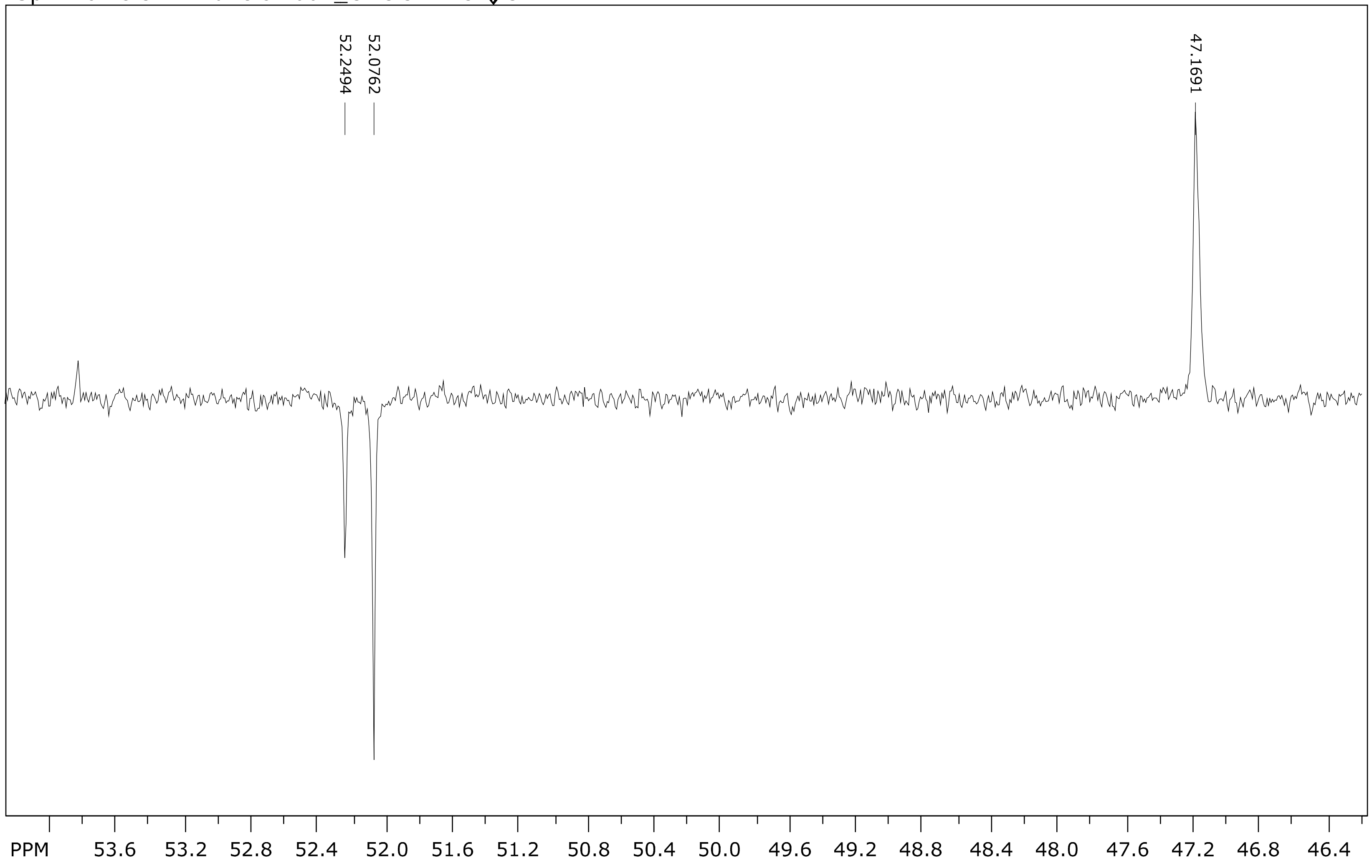

SpinWorks 3: L. Barisic 2601_CDCl3 -45 �C

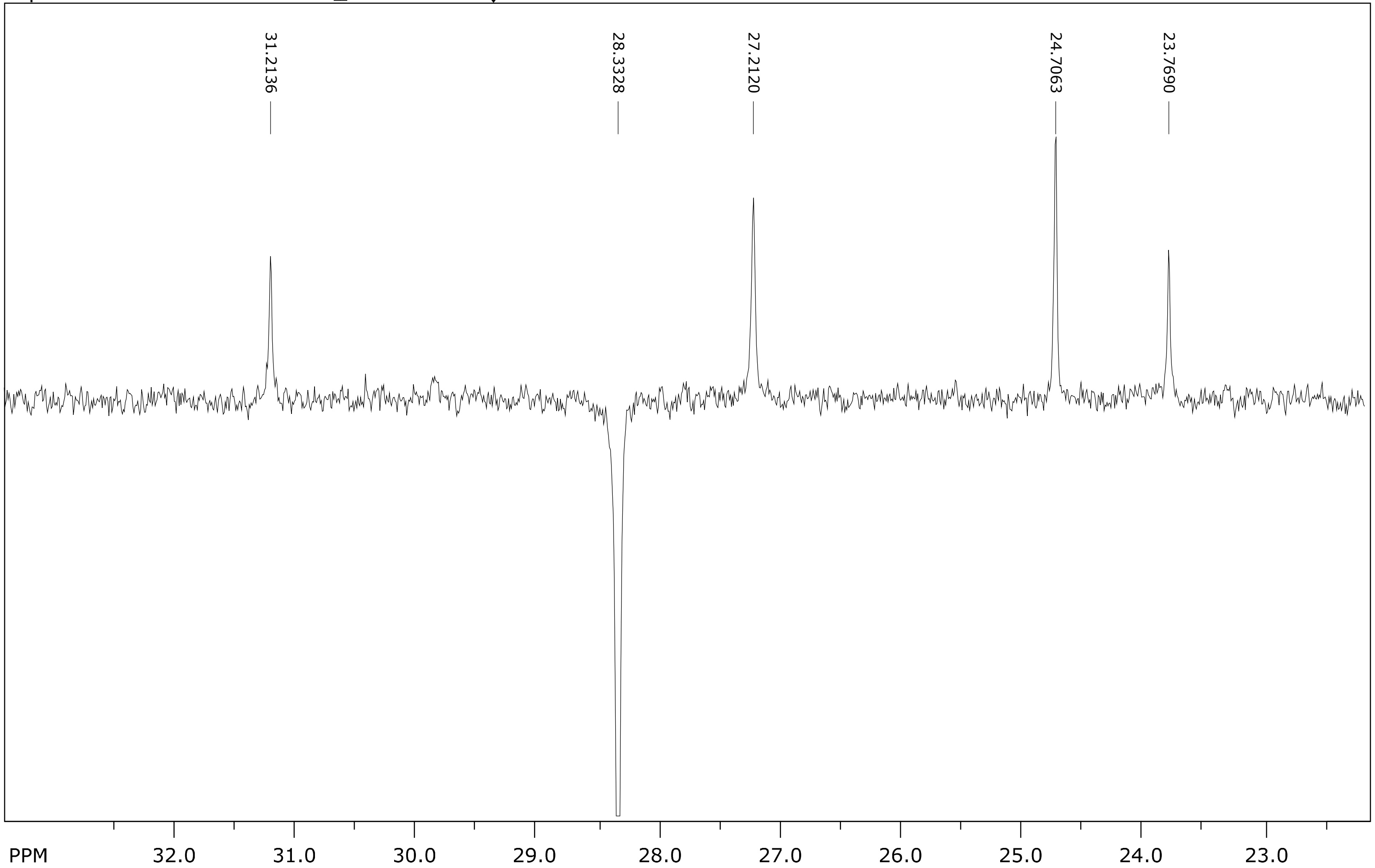

Figure S14. ^1H NMR titration of compound **3** with DMSO in $CDCl_3$.

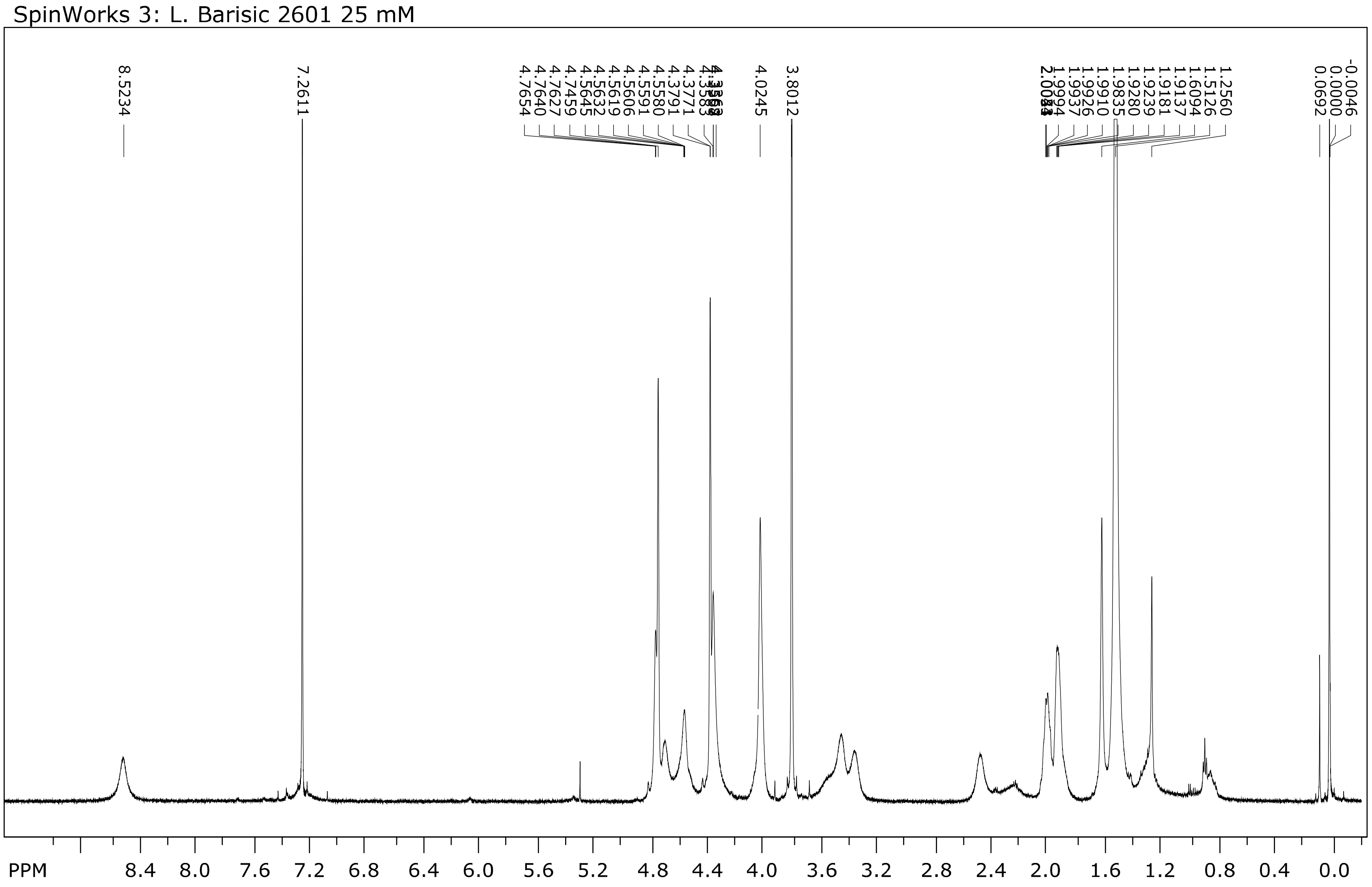

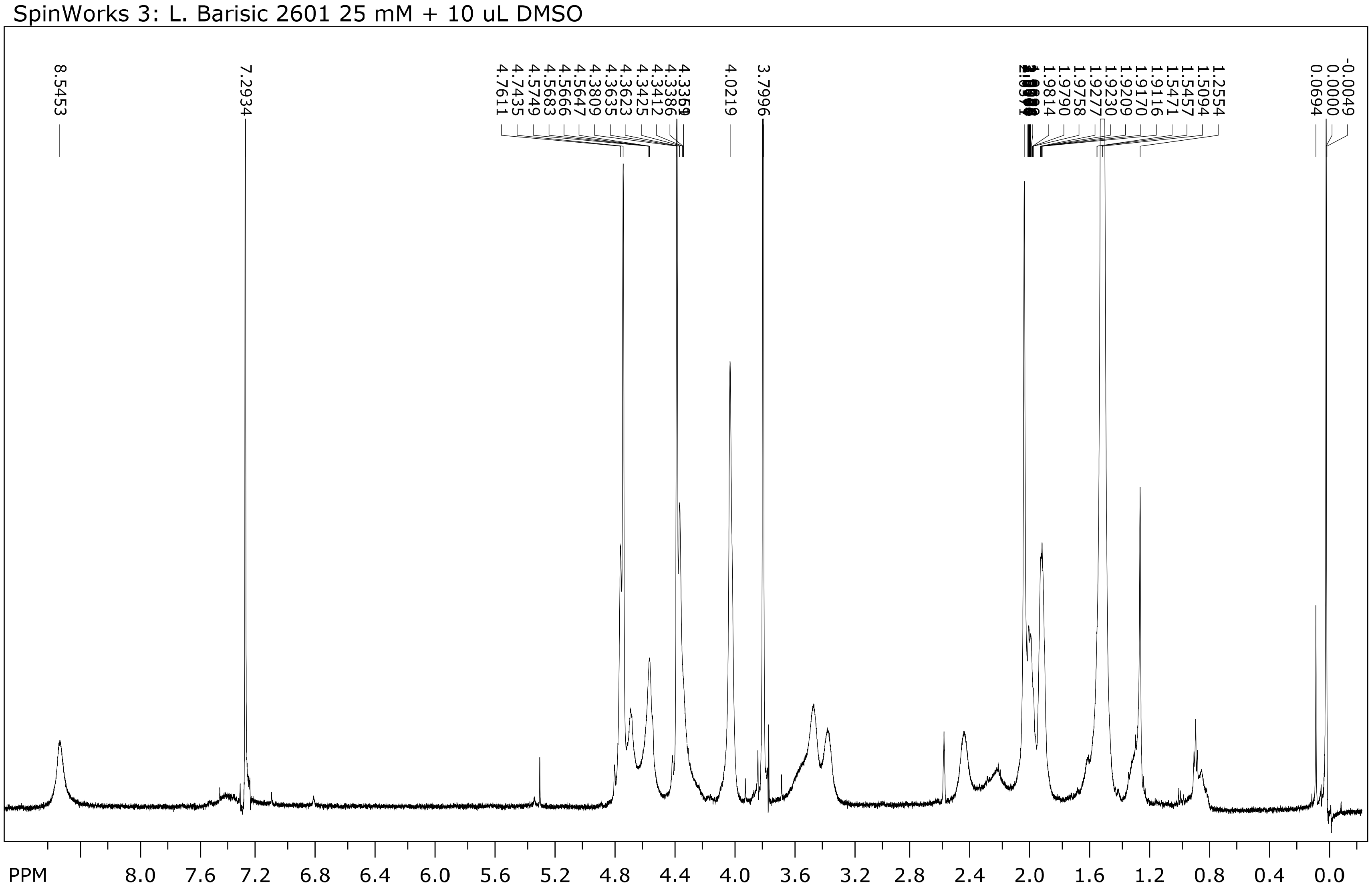
SpinWorks 3: L. Barisic 2601 25 mM + 10 uL DMSO
PPM
8.0 7.6 7.2 6.8 6.4 6.0 5.6 5.2 4.8 4.4 4.0 3.6 3.2 2.8 2.4 2.0 1.6 1.2 0.8 0.4 0.0
8.5453
7.2934
4.7611
4.7435
4.5749
4.5683
4.5666
4.5647
4.3809
4.3635
4.3623
4.3425
4.3412
4.3386
4.0219
3.7996
1.9814
1.9790
1.9758
1.9277
1.9230
1.9209
1.9170
1.9116
1.5471
1.5457
1.5094
1.2554
0.0694
0.0000
-0.0049

SpinWorks 3: L. Barisic 2601 25 mM + 20 uL DMSO

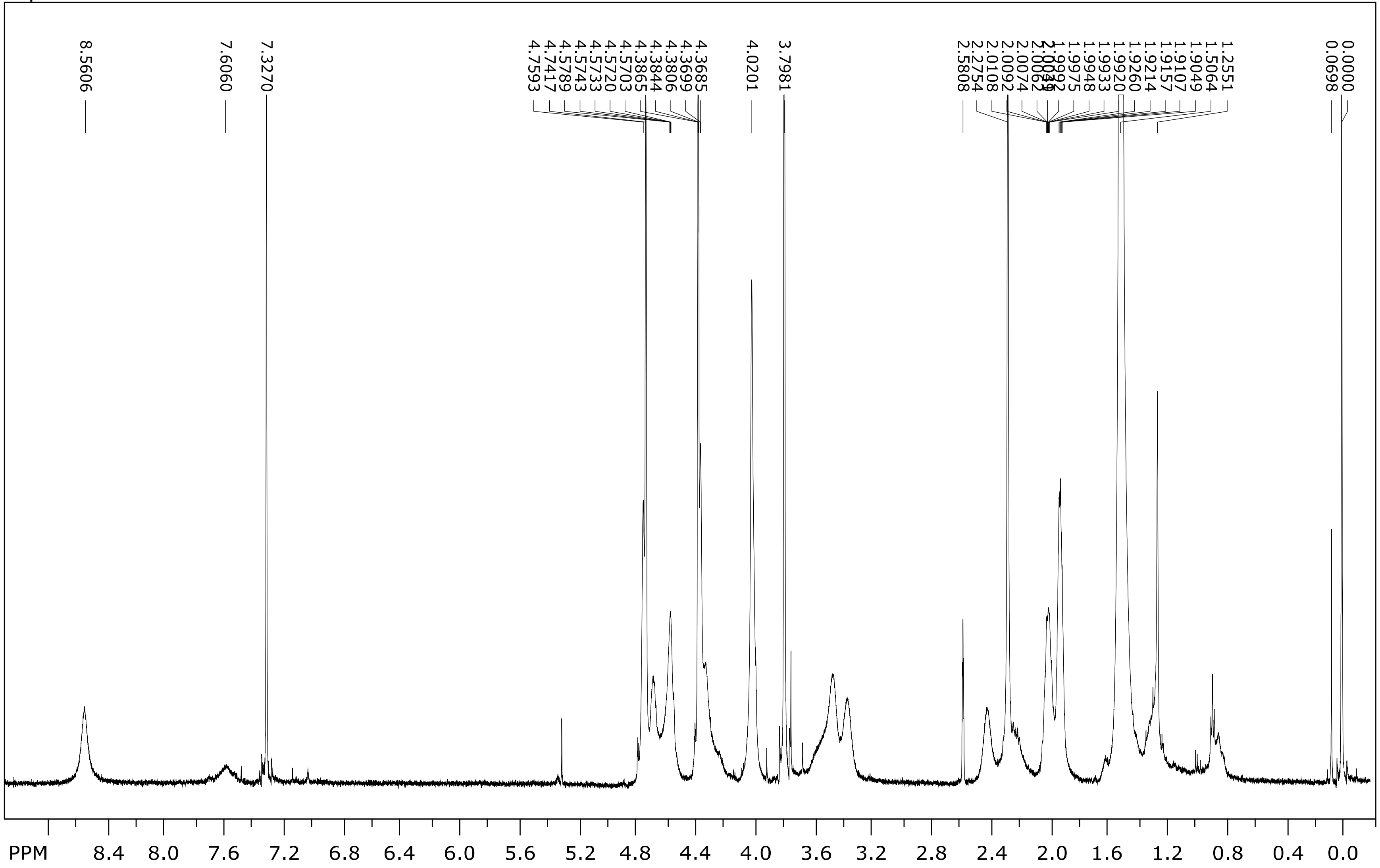

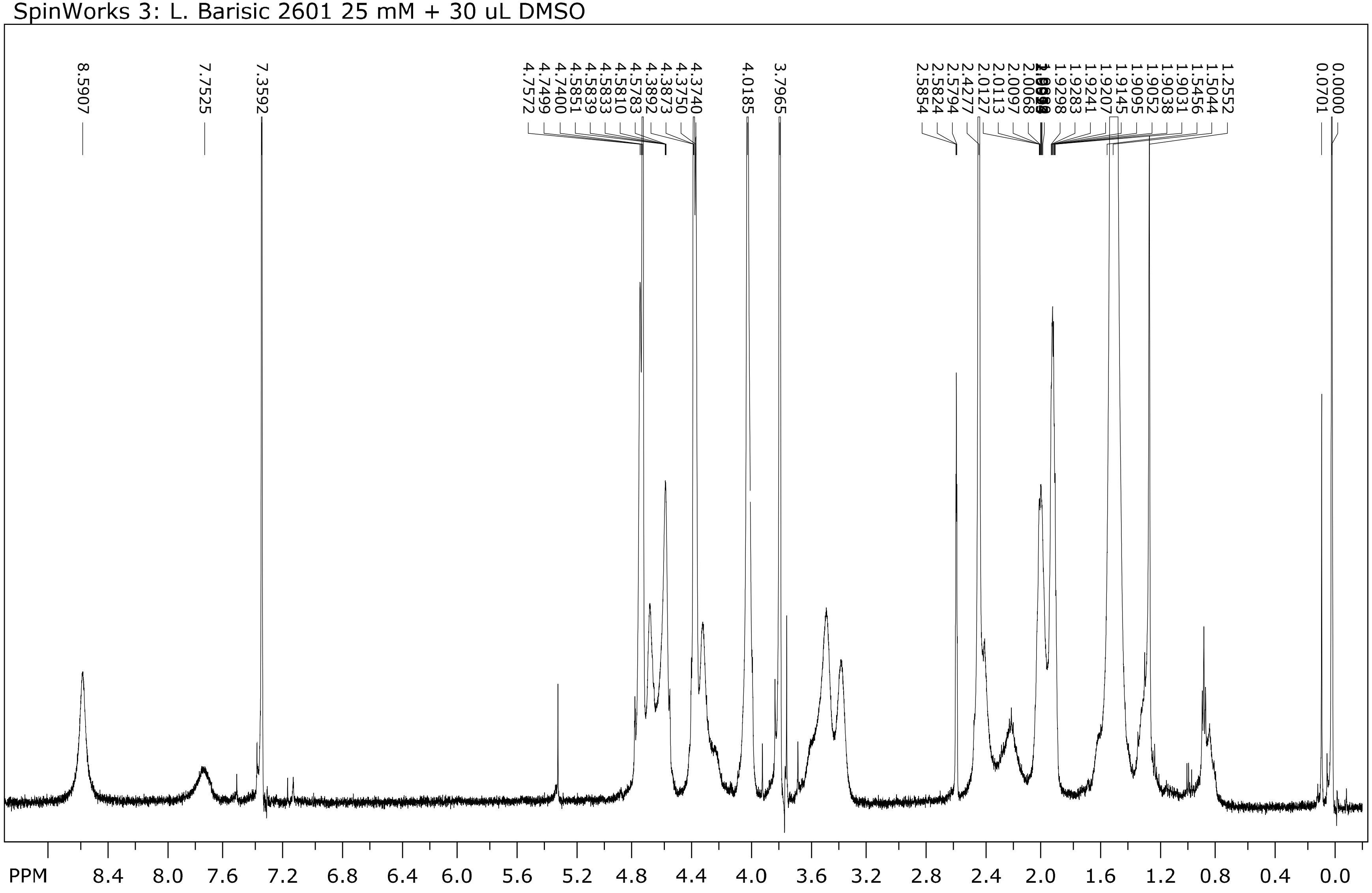
SpinWorks 3: L. Barisic 2601 25 mM + 30 uL DMSO
8.5907
7.7525
7.3592
4.7572
4.7499
4.7400
4.5851
4.5839
4.5833
4.5810
4.5783
4.3892
4.3873
4.3750
4.3740
4.0185
3.7965
2.5854
2.5824
2.5794
2.4277
2.0127
2.0113
2.0097
2.0068
1.9298
1.9283
1.9241
1.9207
1.9145
1.9095
1.9052
1.9038
1.9031
1.5456
1.5044
1.2552
0.0701
0.0000
PPM
8.4
8.0
7.6
7.2
6.8
6.4
6.0
5.6
5.2
4.8
4.4
4.0
3.6
3.2
2.8
2.4
2.0
1.6
1.2
0.8
0.4
0.0

SpinWorks 3: L. Barisic 2601 25 mM + 40 uL DMSO

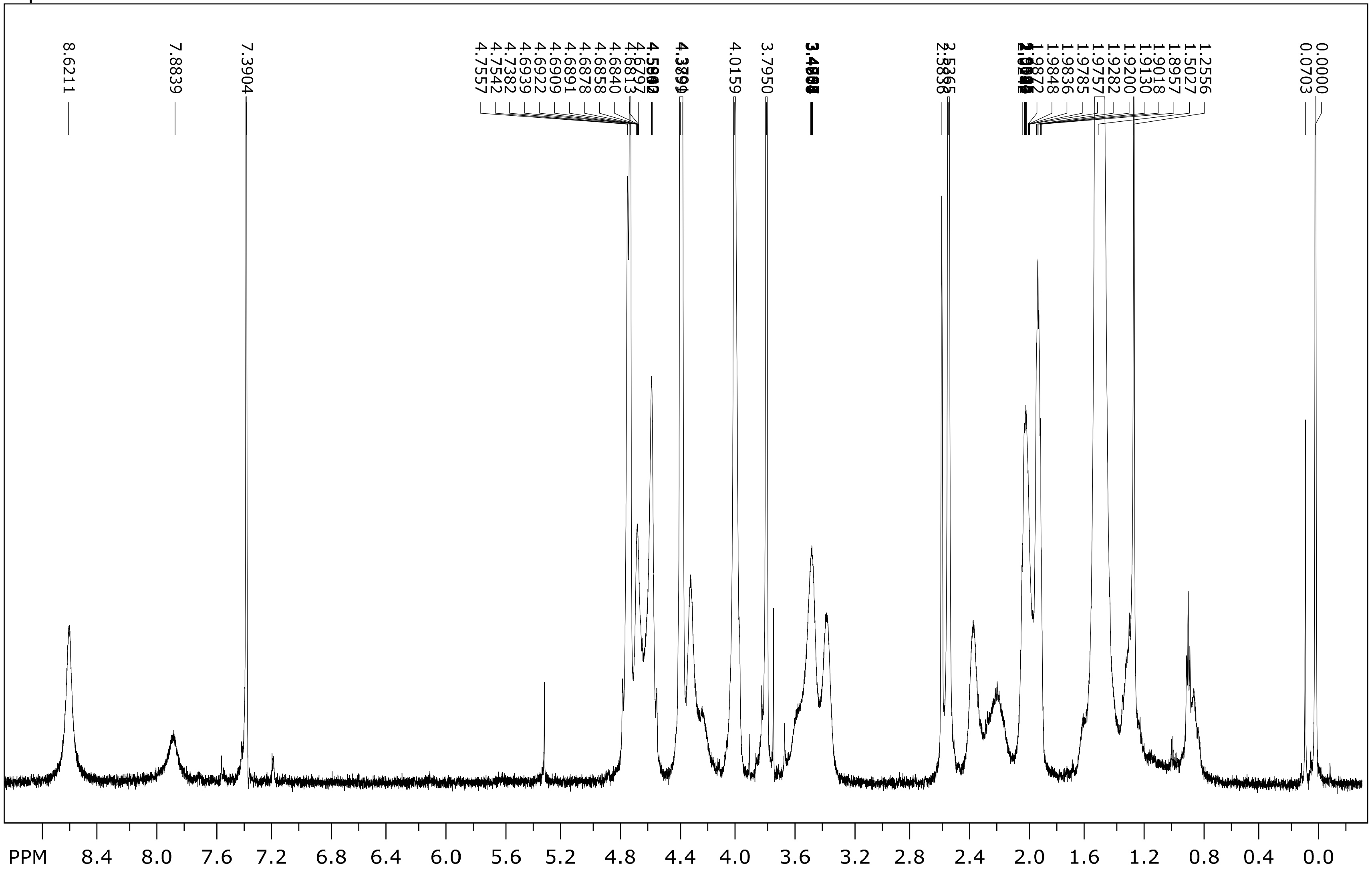

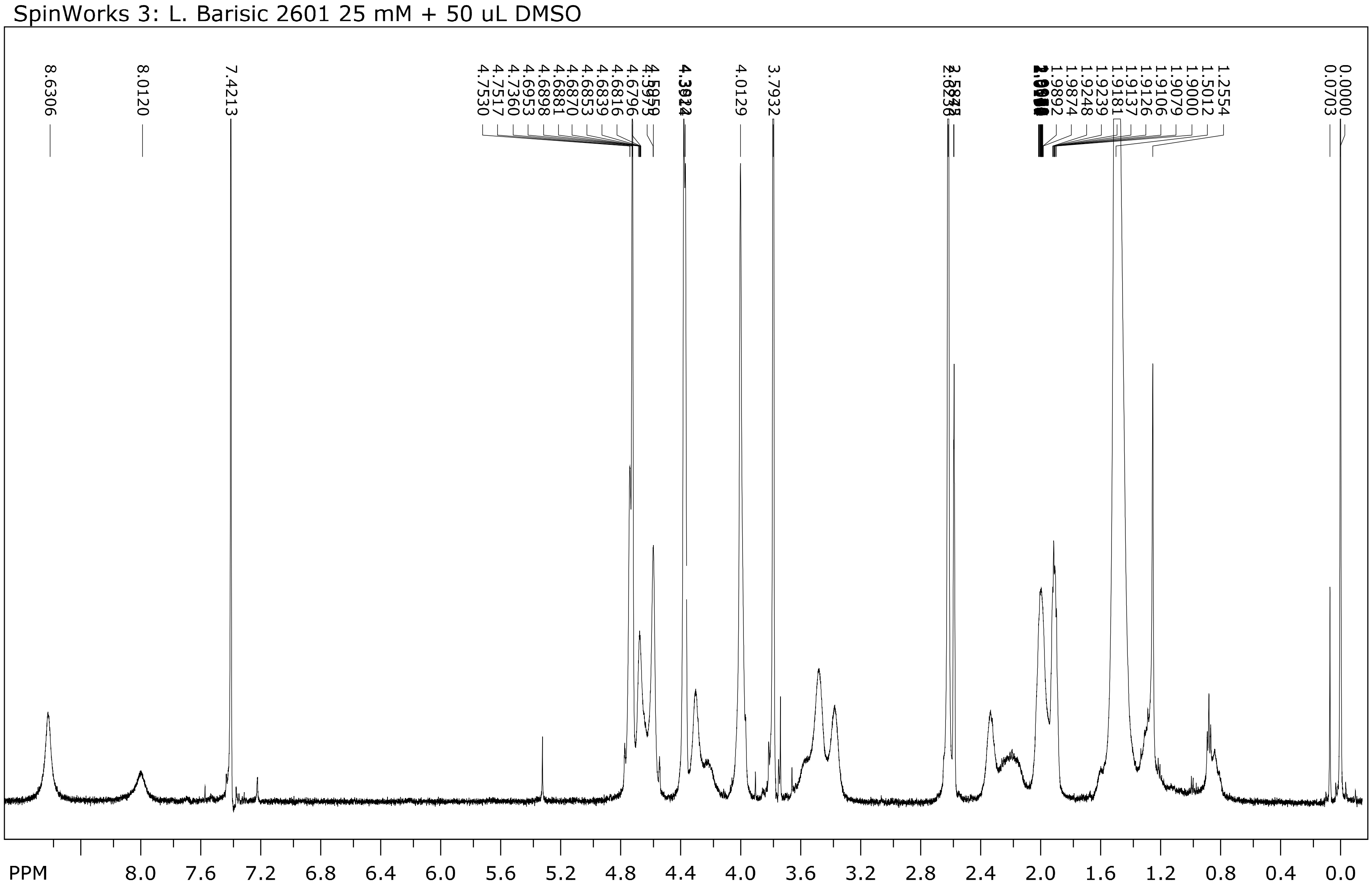
SpinWorks 3: L. Barisic 2601 25 mM + 50 uL DMSO
8.6306
8.0120
7.4213
4.7530
4.7517
4.7360
4.6953
4.6898
4.6881
4.6870
4.6853
4.6839
4.6816
4.6796
4.0129
3.7932
1.9892
1.9874
1.9248
1.9239
1.9181
1.9137
1.9126
1.9106
1.9079
1.9000
1.5012
1.2554
0.0703
0.0000
PPM
8.0
7.6
7.2
6.8
6.4
6.0
5.6
5.2
4.8
4.4
4.0
3.6
3.2
2.8
2.4
2.0
1.6
1.2
0.8
0.4
0.0

SpinWorks 3: L. Barisic 2601 25 mM + 70 uL DMSO

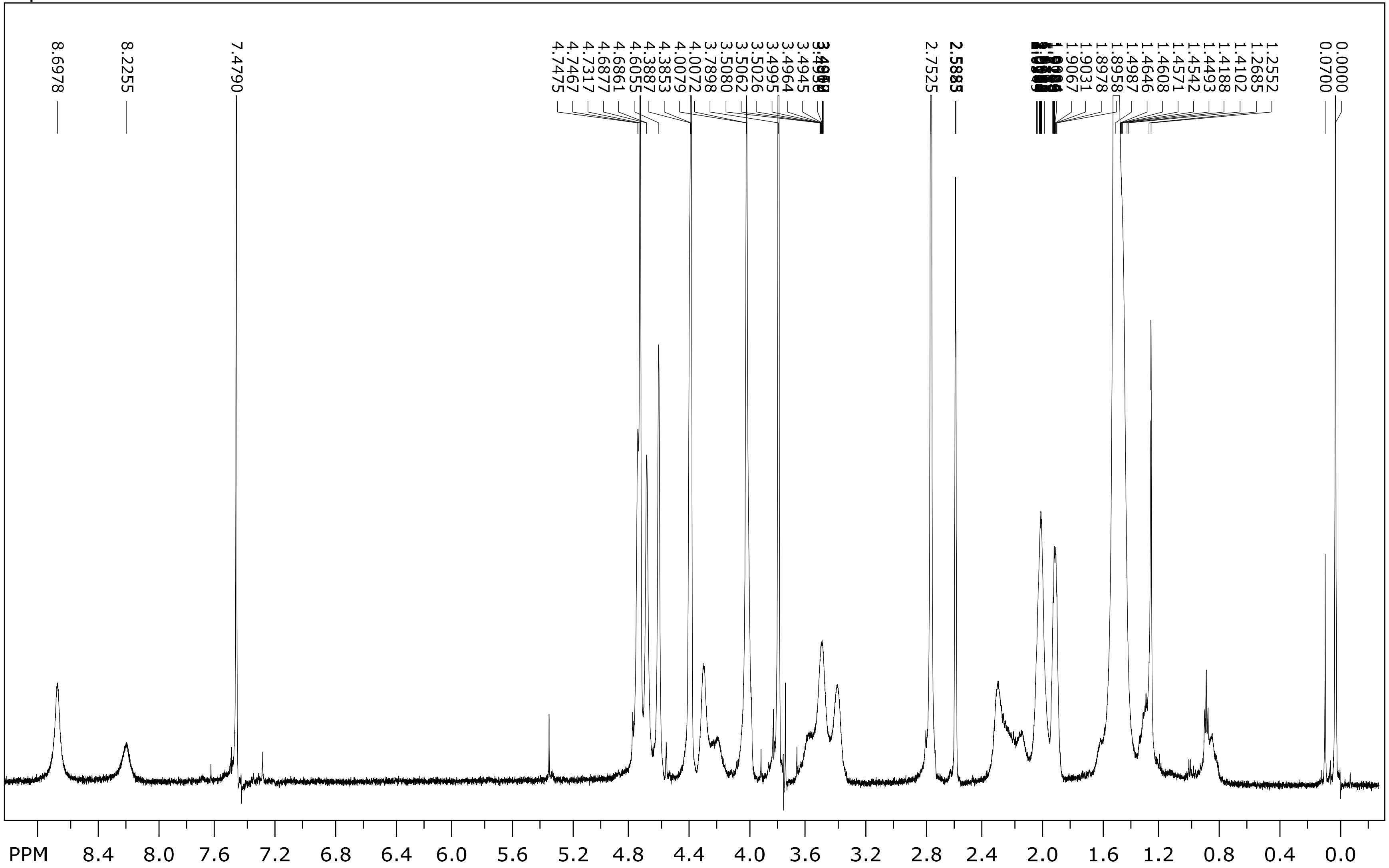

SpinWorks 3: L. Barisic 2601 25 mM + 90 uL DMSO

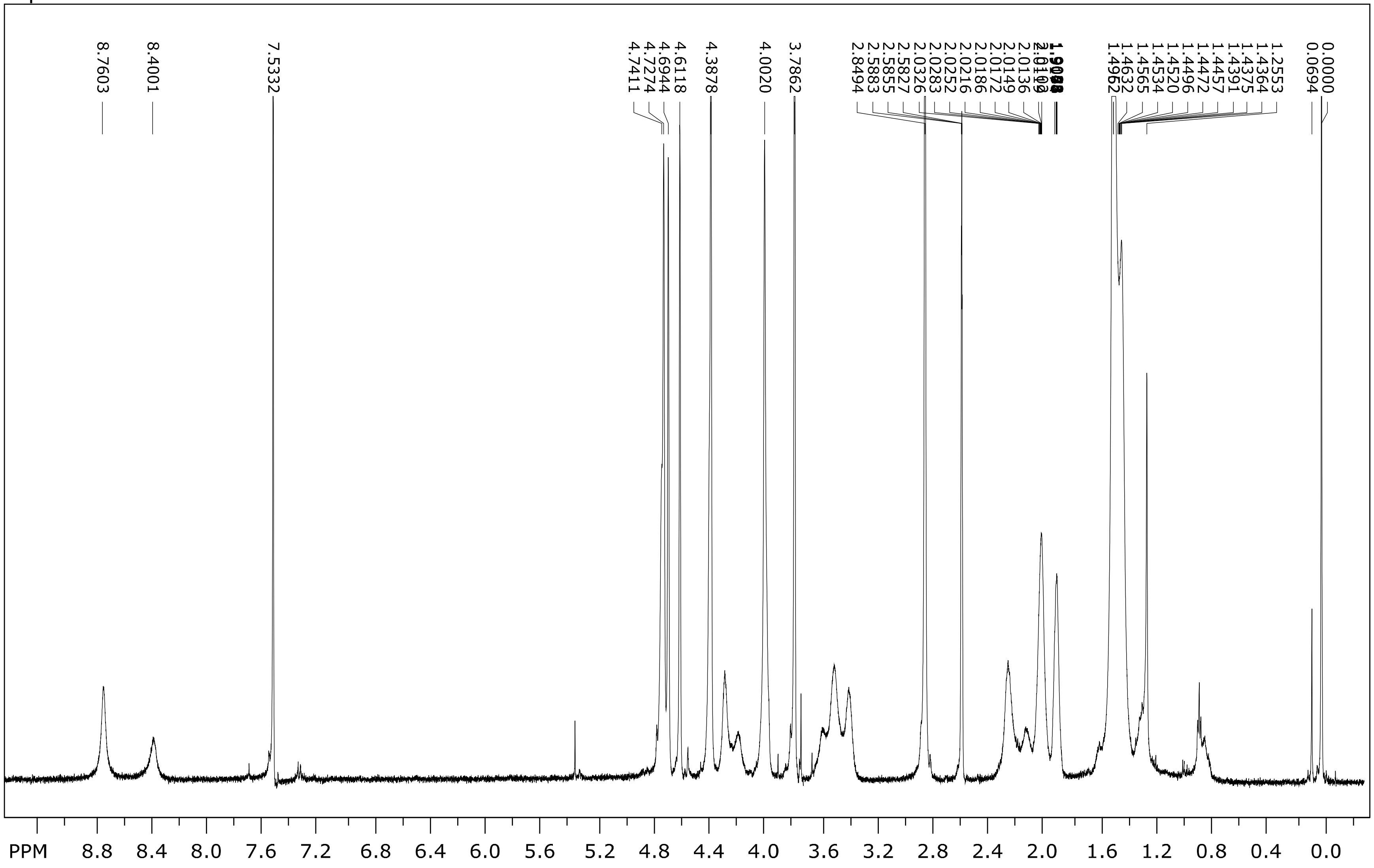

SpinWorks 3: L. Barisic 2601 25 mM + 110 uL DMSO

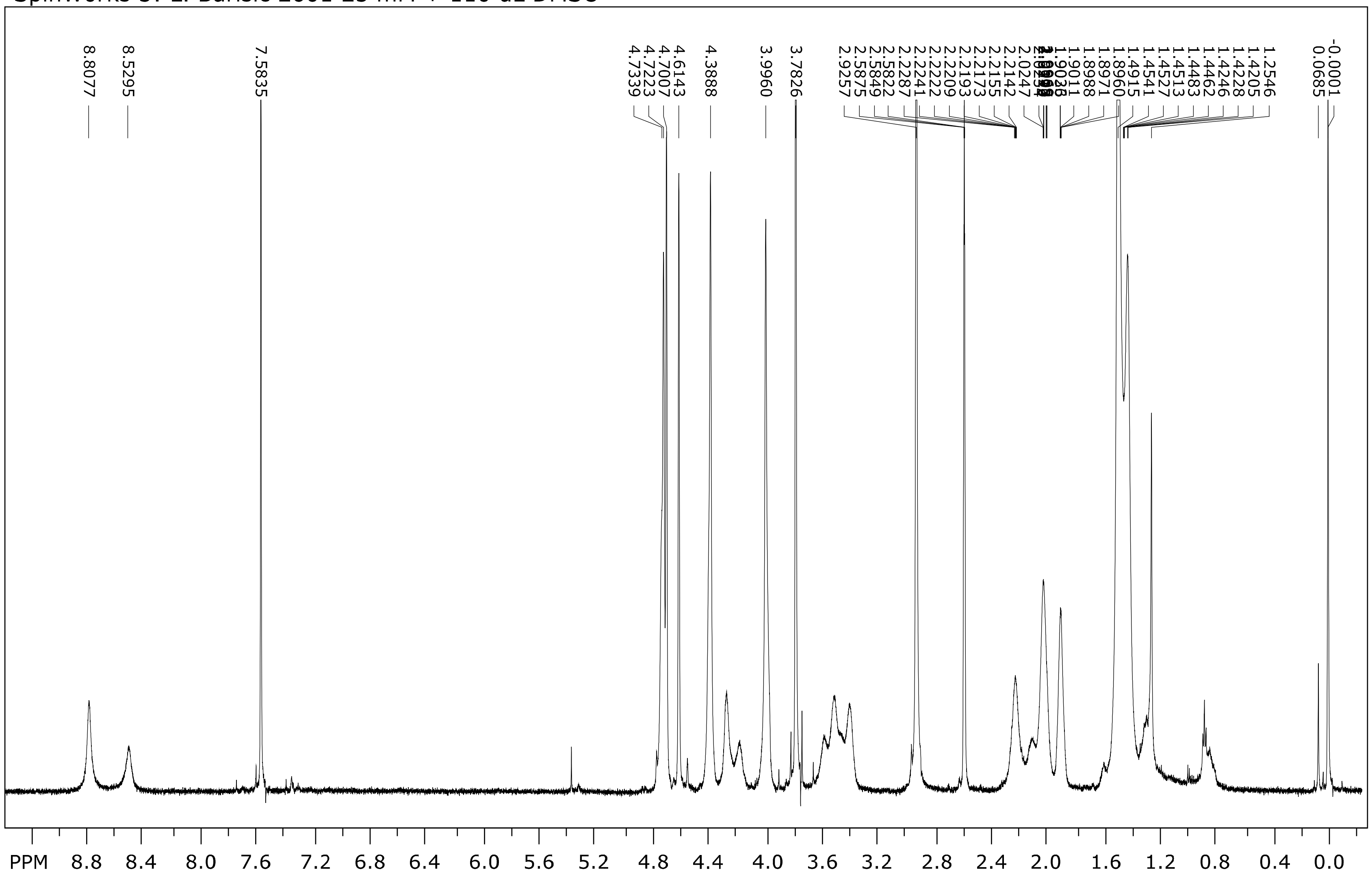

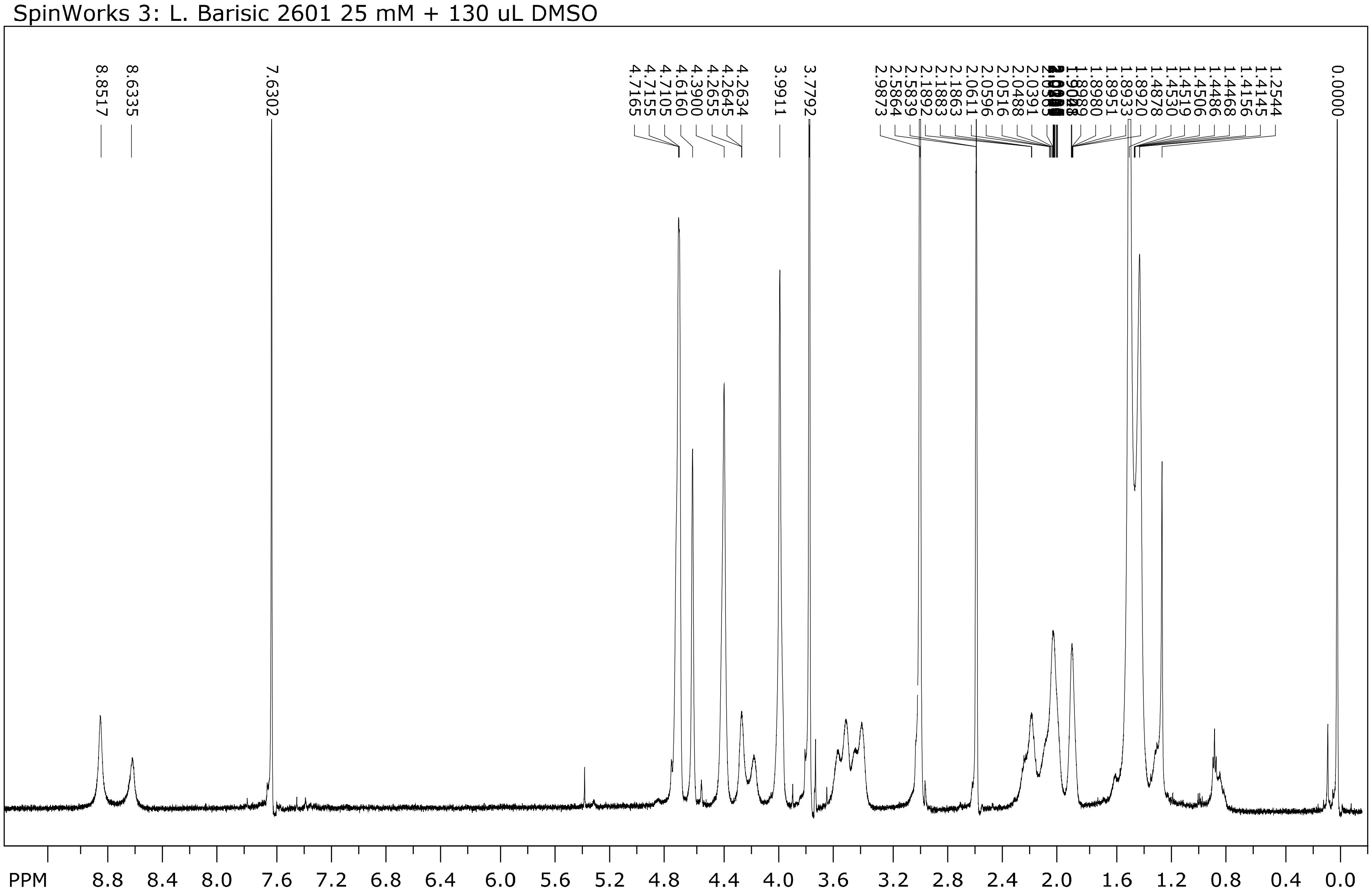
SpinWorks 3: L. Barisic 2601 25 mM + 130 uL DMSO
8.8517
8.6335
7.6302
4.7165
4.7155
4.7105
4.6160
4.3900
4.2655
4.2645
4.2634
3.9911
3.7792
2.9873
2.5864
2.5839
2.1892
2.1883
2.1863
2.0611
2.0596
2.0516
2.0488
2.0391
1.8989
1.8980
1.8951
1.8933
1.8920
1.4878
1.4530
1.4519
1.4506
1.4486
1.4468
1.4156
1.4145
1.2544
0.0000
PPM
8.8
8.4
8.0
7.6
7.2
6.8
6.4
6.0
5.6
5.2
4.8
4.4
4.0
3.6
3.2
2.8
2.4
2.0
1.6
1.2
0.8
0.4
0.0

SpinWorks 3: L. Barisic 2601 25 mM + 180 uL DMSO

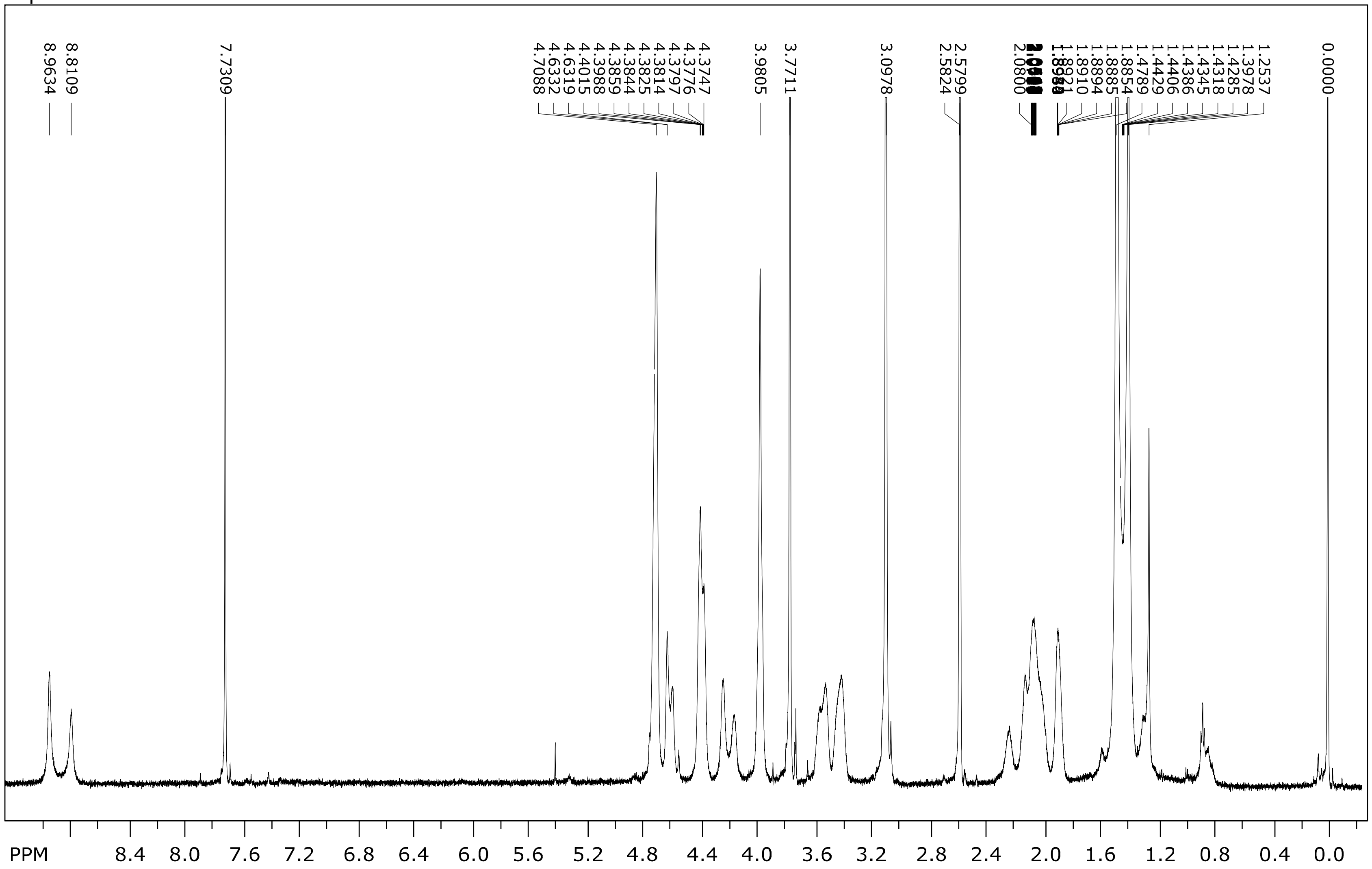

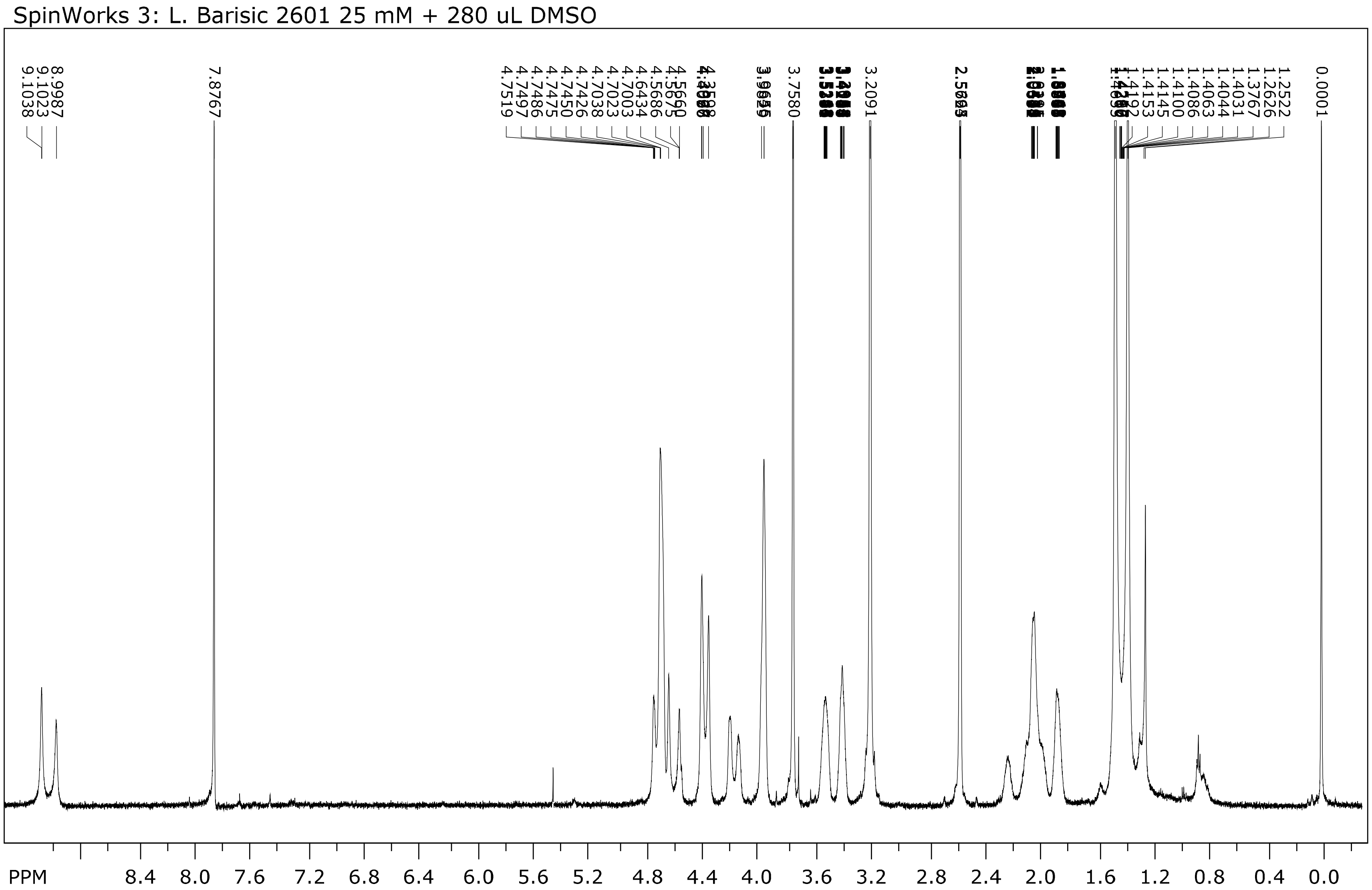
SpinWorks 3: L. Barisic 2601 25 mM + 280 uL DMSO
PPM

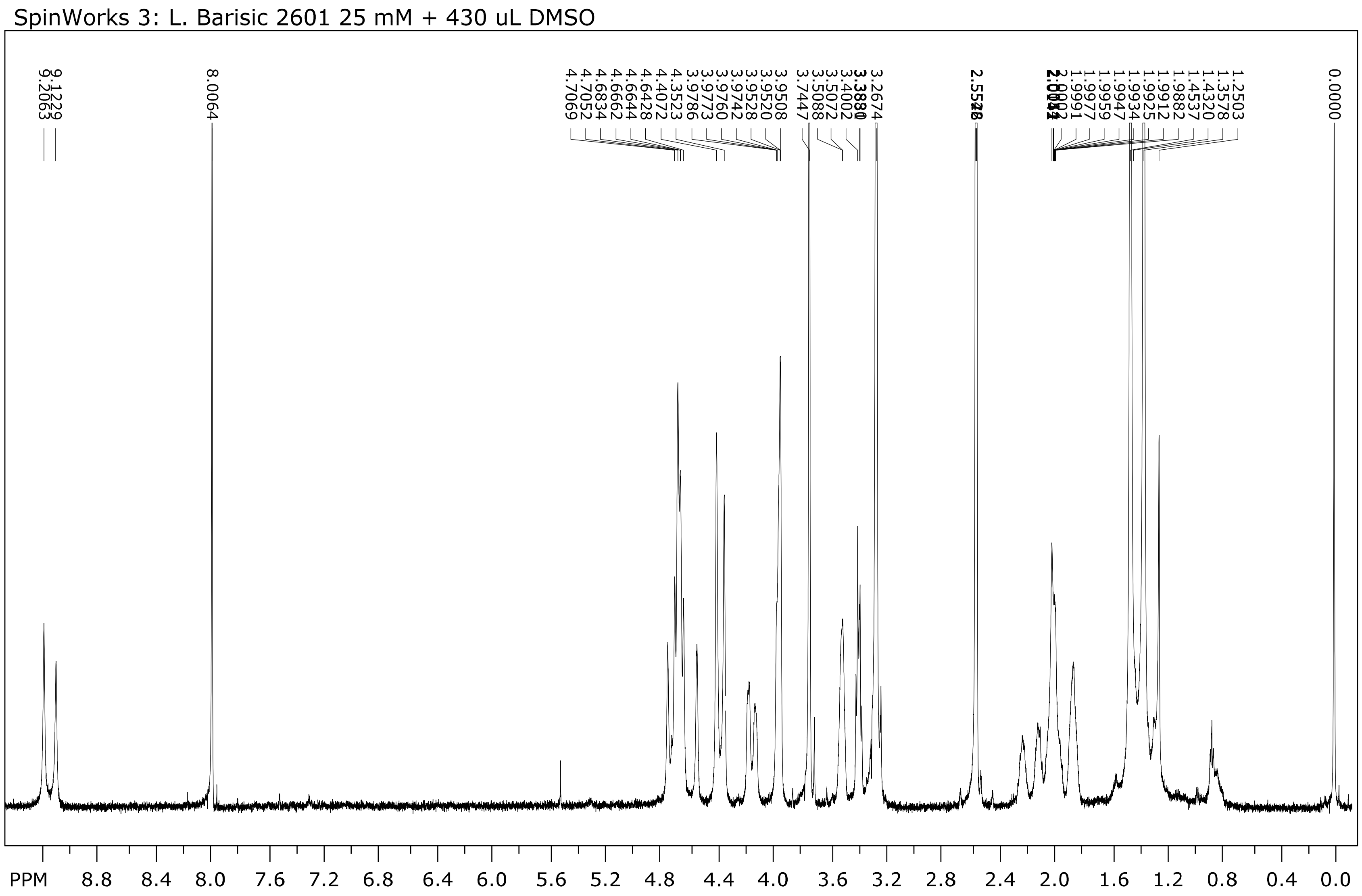
SpinWorks 3: L. Barisic 2601 25 mM + 430 uL DMSO
PPM

SpinWorks 3: L. Barisic 2601 25 mM + 630 uL DMSO

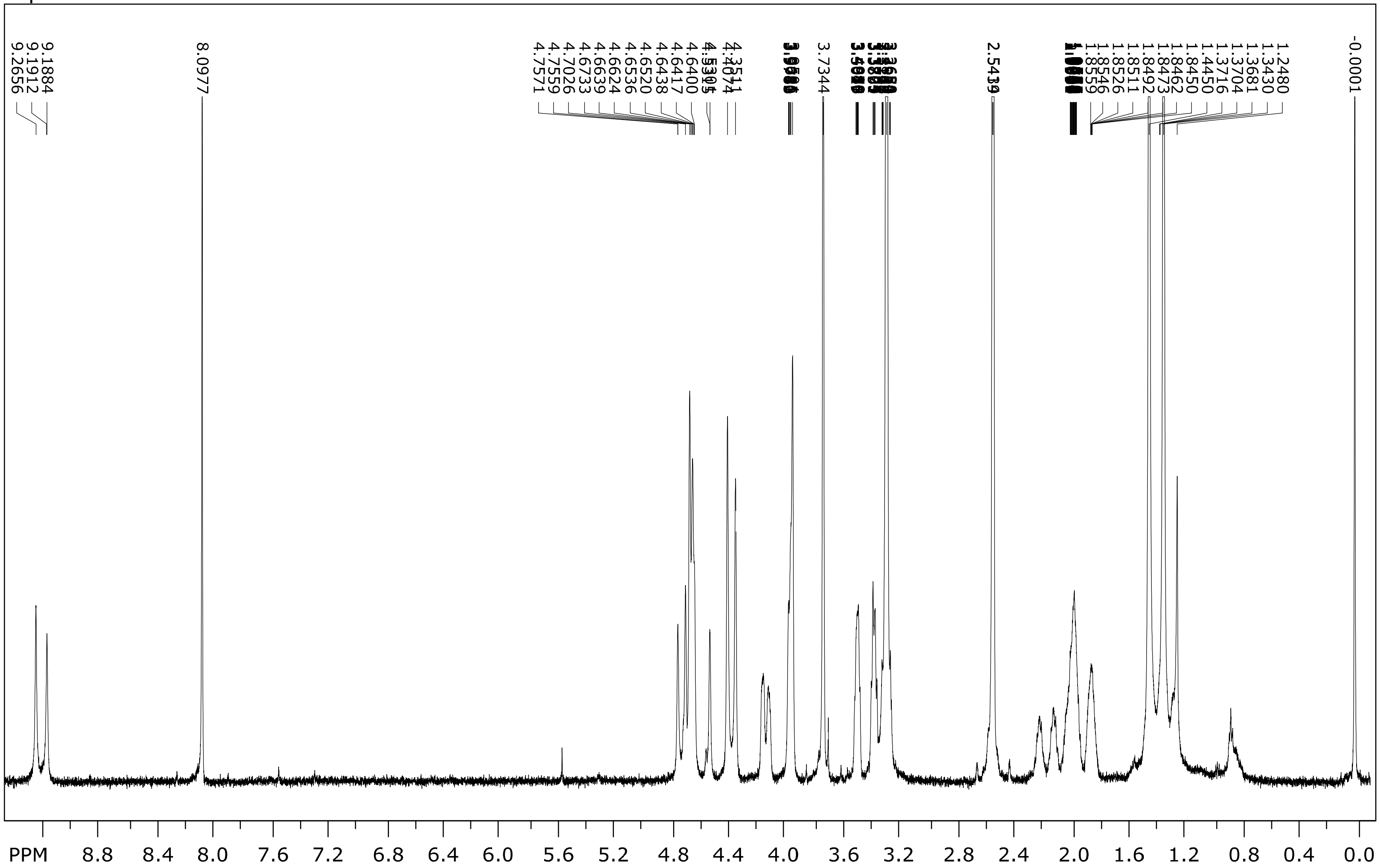

Figure S15. Variable-temperature ^{1}H NMR spectra of **3**.

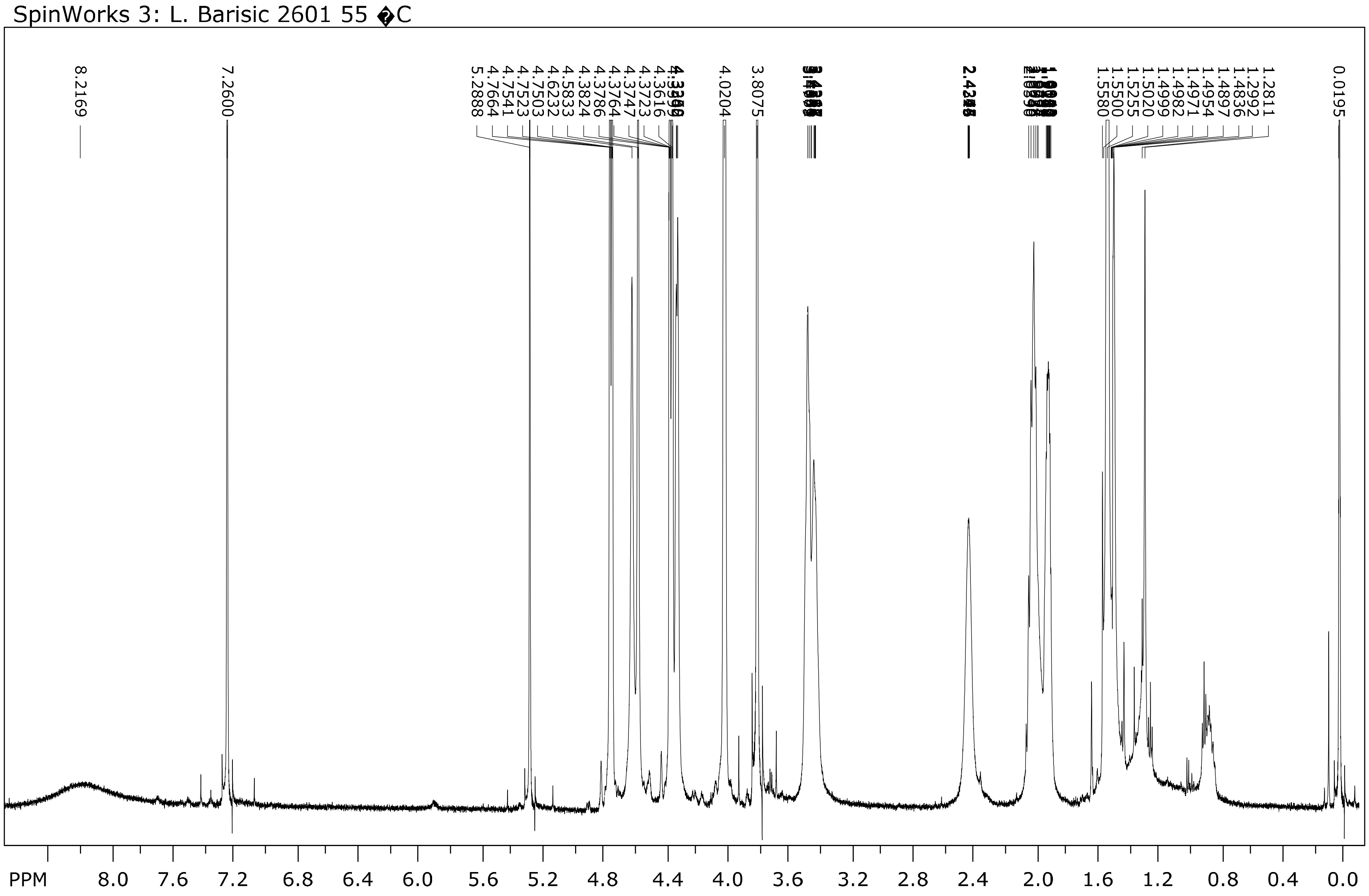

SpinWorks 3: L. Barisic 2601 45 �C

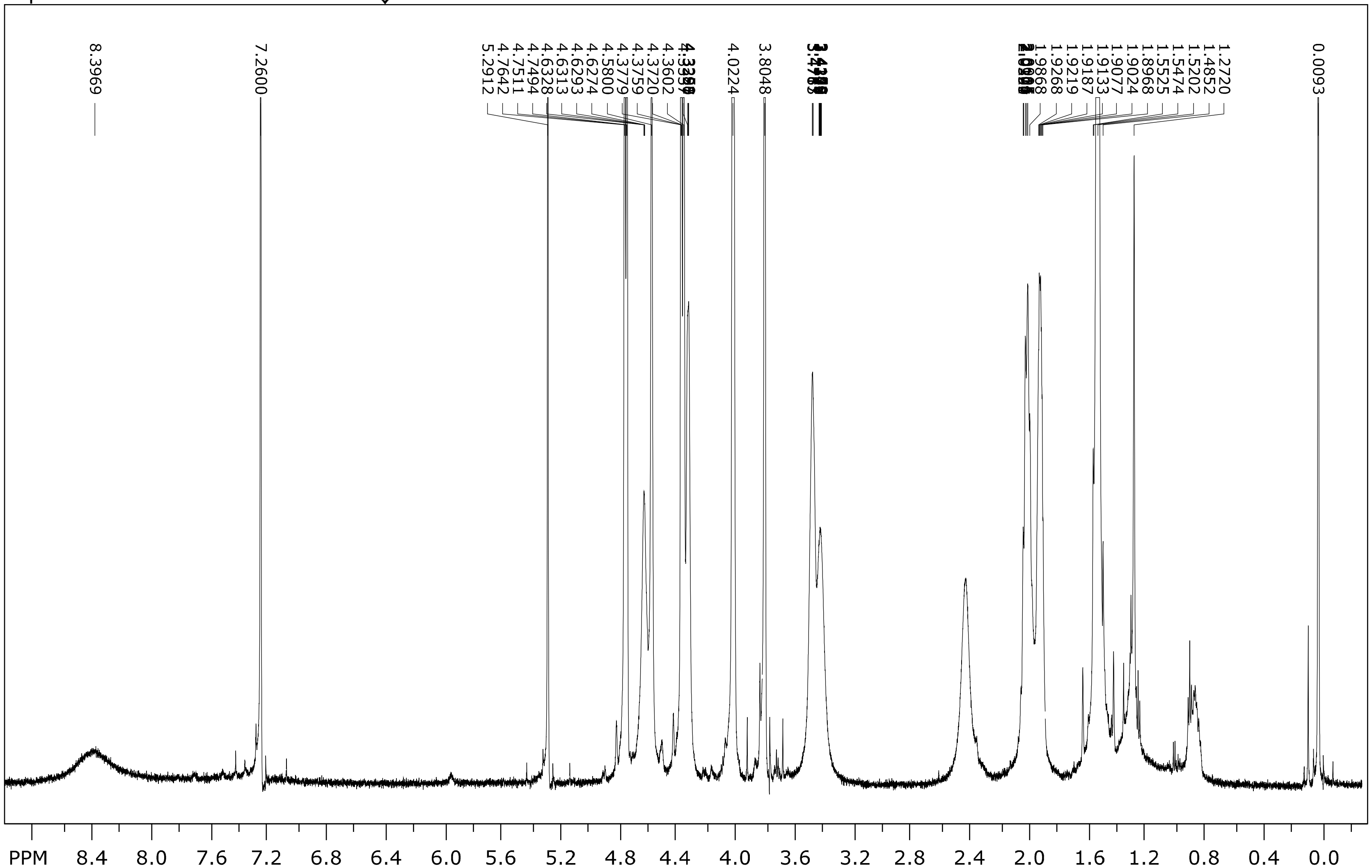

SpinWorks 3: L. Barisic 2601 35 �C

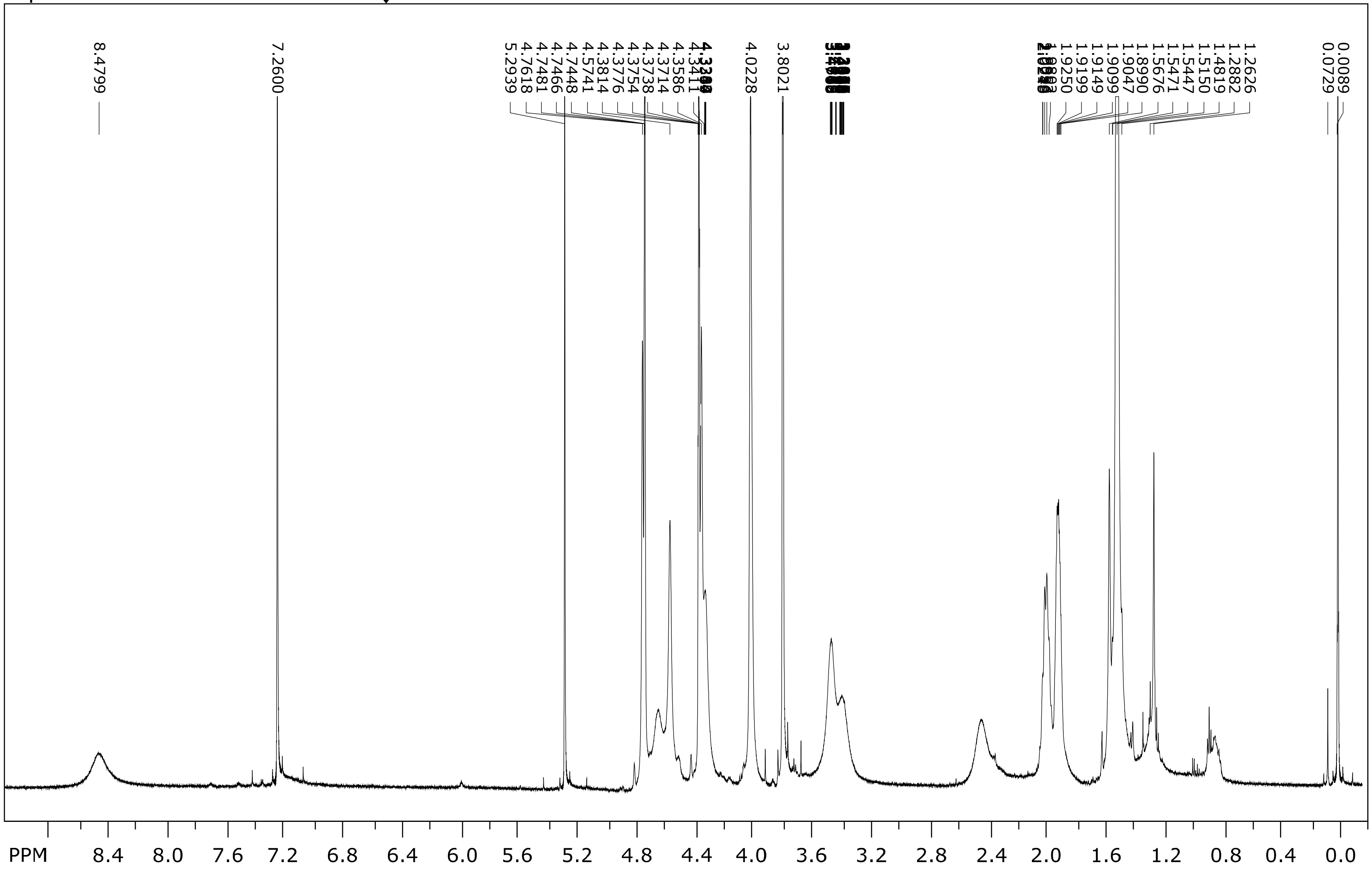

SpinWorks 3: L. Barisic 2601 25 �C

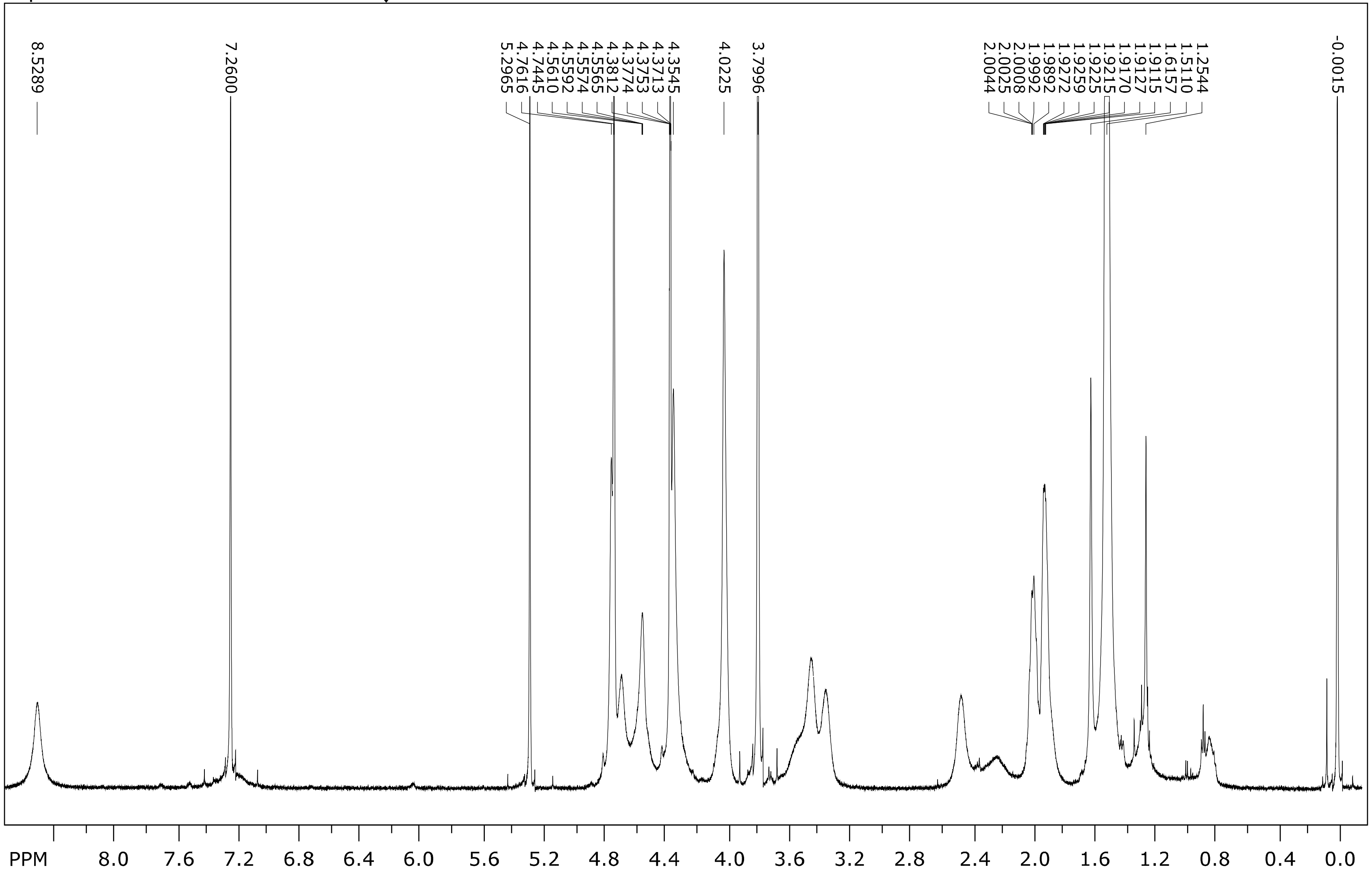

SpinWorks 3: L. Barisic 2601 15 �C

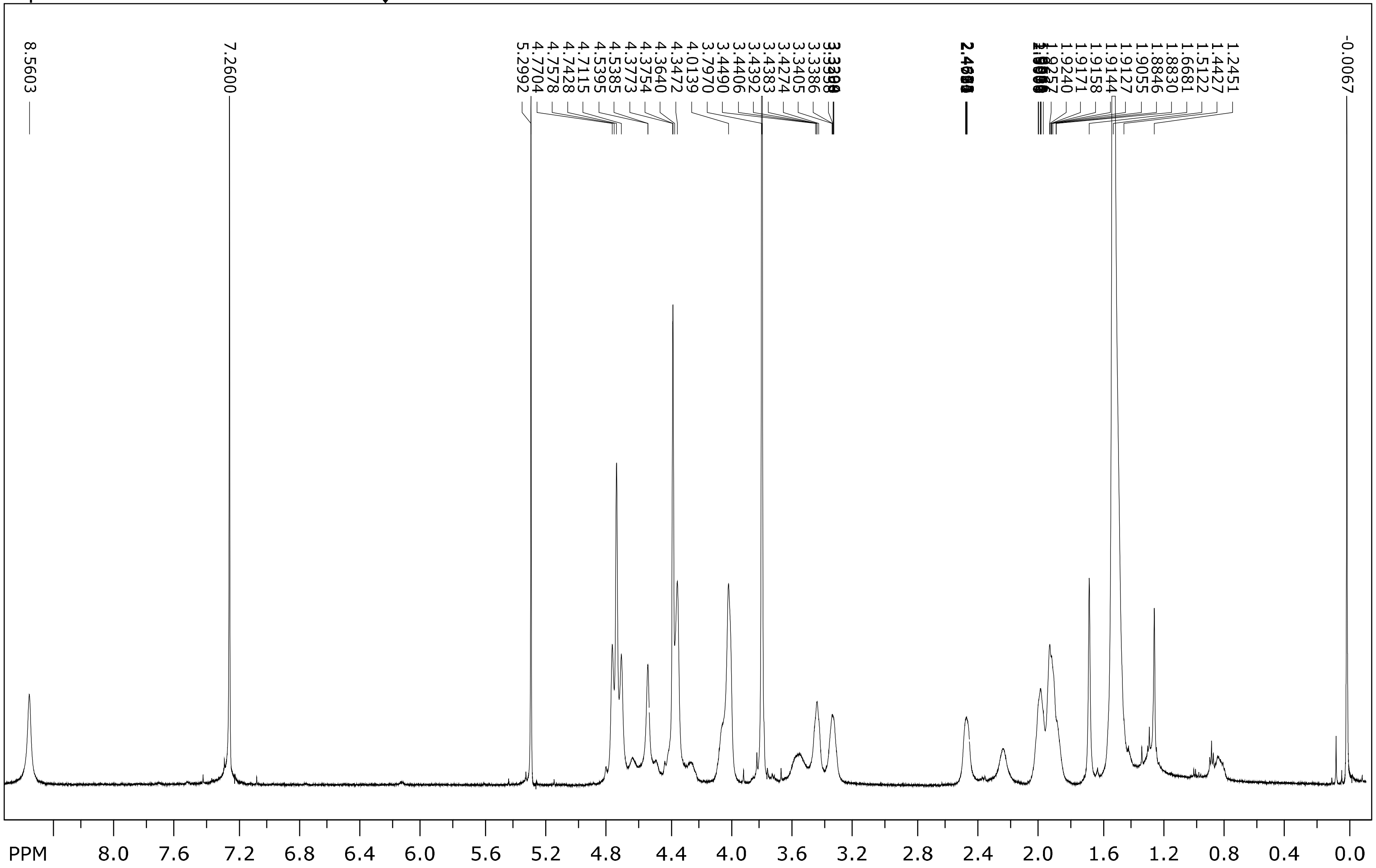

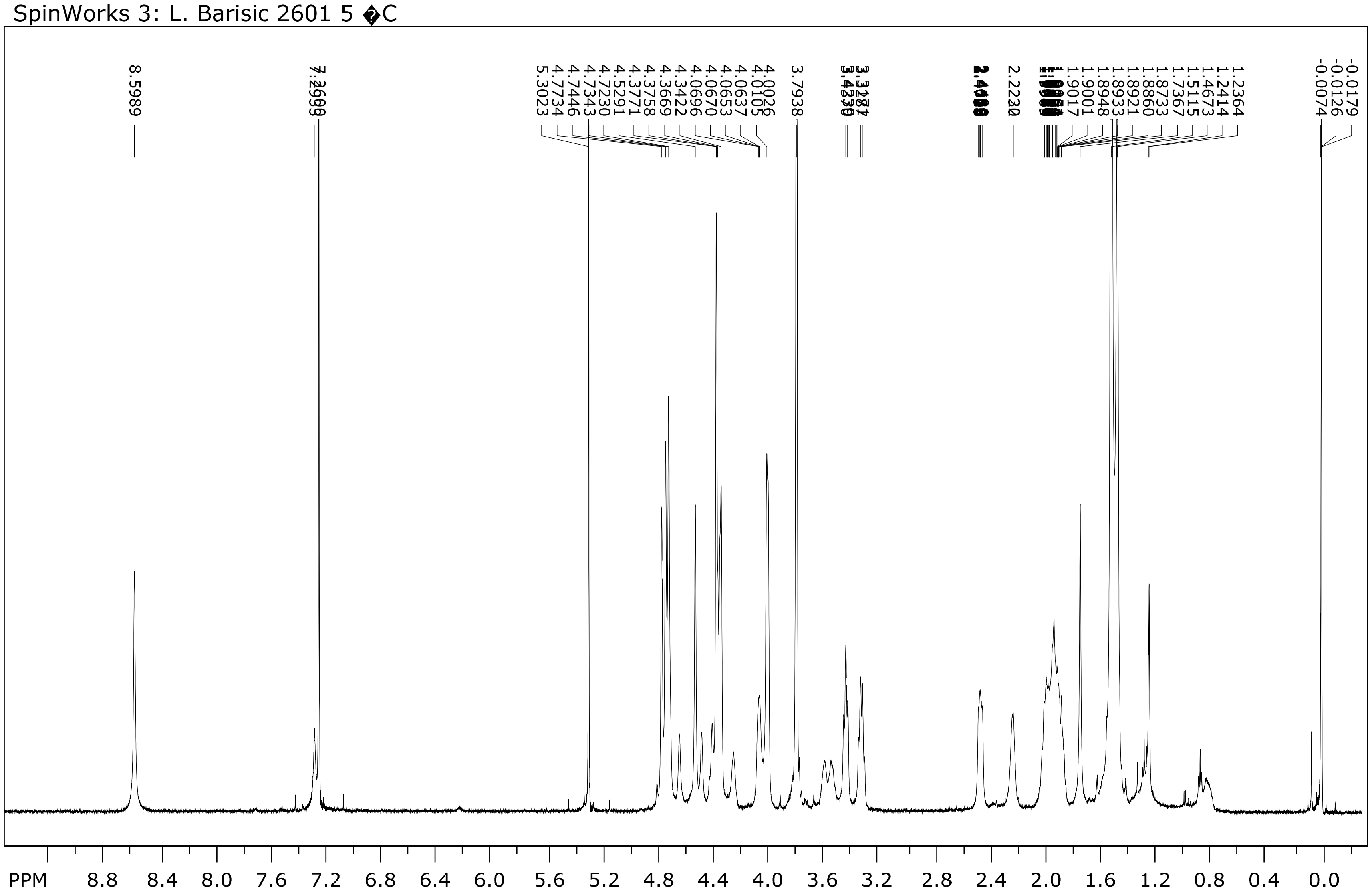
SpinWorks 3: L. Barisic 2601 5 �C
8.5989
5.3023
4.7734
4.7446
4.7343
4.7230
4.5291
4.3771
4.3758
4.3669
4.3422
4.0696
4.0670
4.0653
4.0637
4.0105
4.0026
3.7938
2.2220
1.9017
1.9001
1.8948
1.8933
1.8921
1.8860
1.8733
1.7367
1.5115
1.4673
1.2414
1.2364
-0.0074
-0.0126
-0.0179
PPM
8.8
8.4
8.0
7.6
7.2
6.8
6.4
6.0
5.6
5.2
4.8
4.4
4.0
3.6
3.2
2.8
2.4
2.0
1.6
1.2
0.8
0.4
0.0

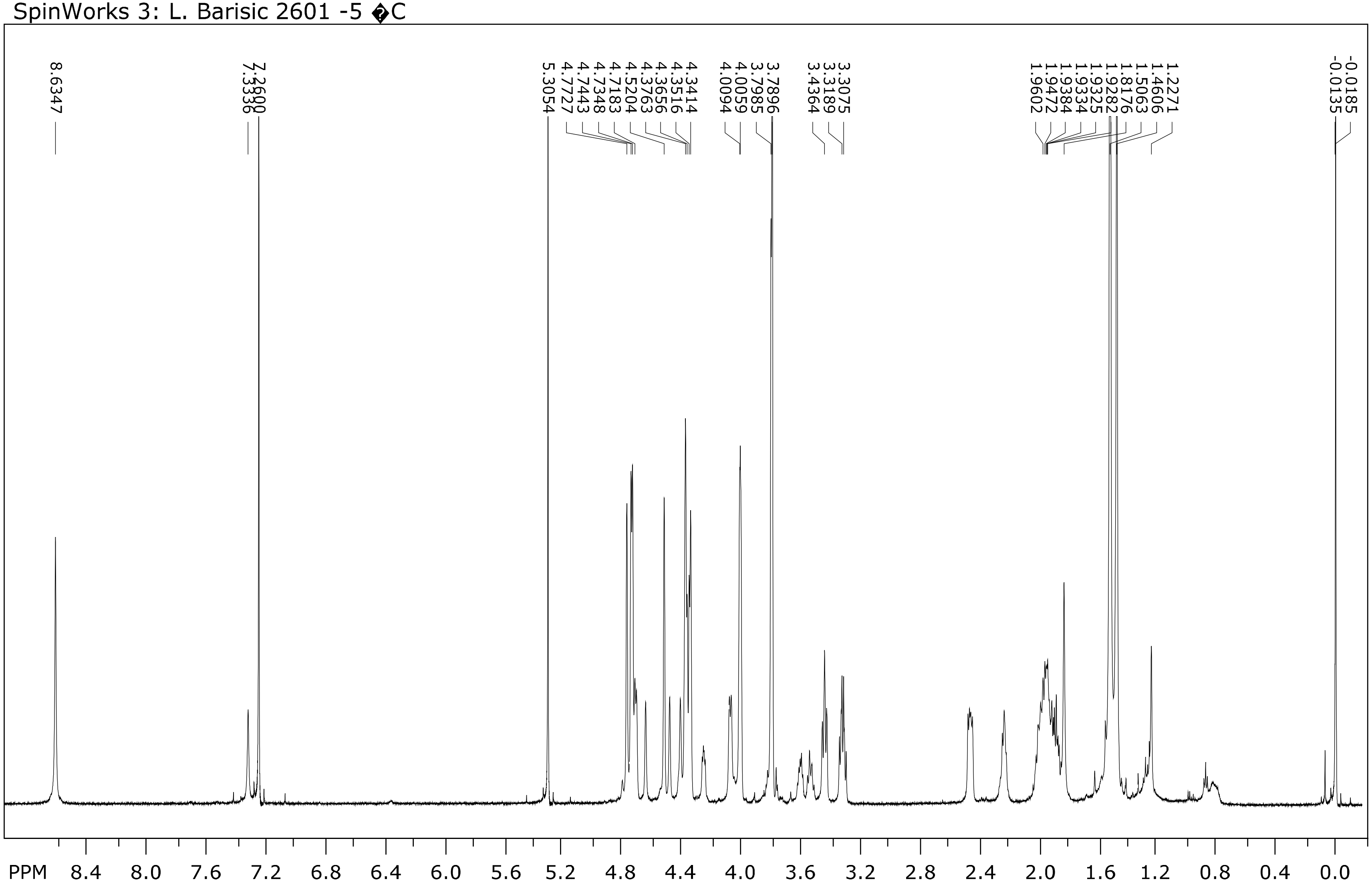
SpinWorks 3: L. Barisic 2601 -5 �C
8.6347
7.3336
7.2600
5.3054
4.7727
4.7443
4.7348
4.7183
4.5204
4.3763
4.3656
4.3516
4.3414
4.0094
4.0059
3.7985
3.7896
3.4364
3.3189
3.3075
1.9602
1.9472
1.9384
1.9334
1.9325
1.9282
1.8176
1.5063
1.4606
1.2271
-0.0135
-0.0185
PPM 8.4 8.0 7.6 7.2 6.8 6.4 6.0 5.6 5.2 4.8 4.4 4.0 3.6 3.2 2.8 2.4 2.0 1.6 1.2 0.8 0.4 0.0

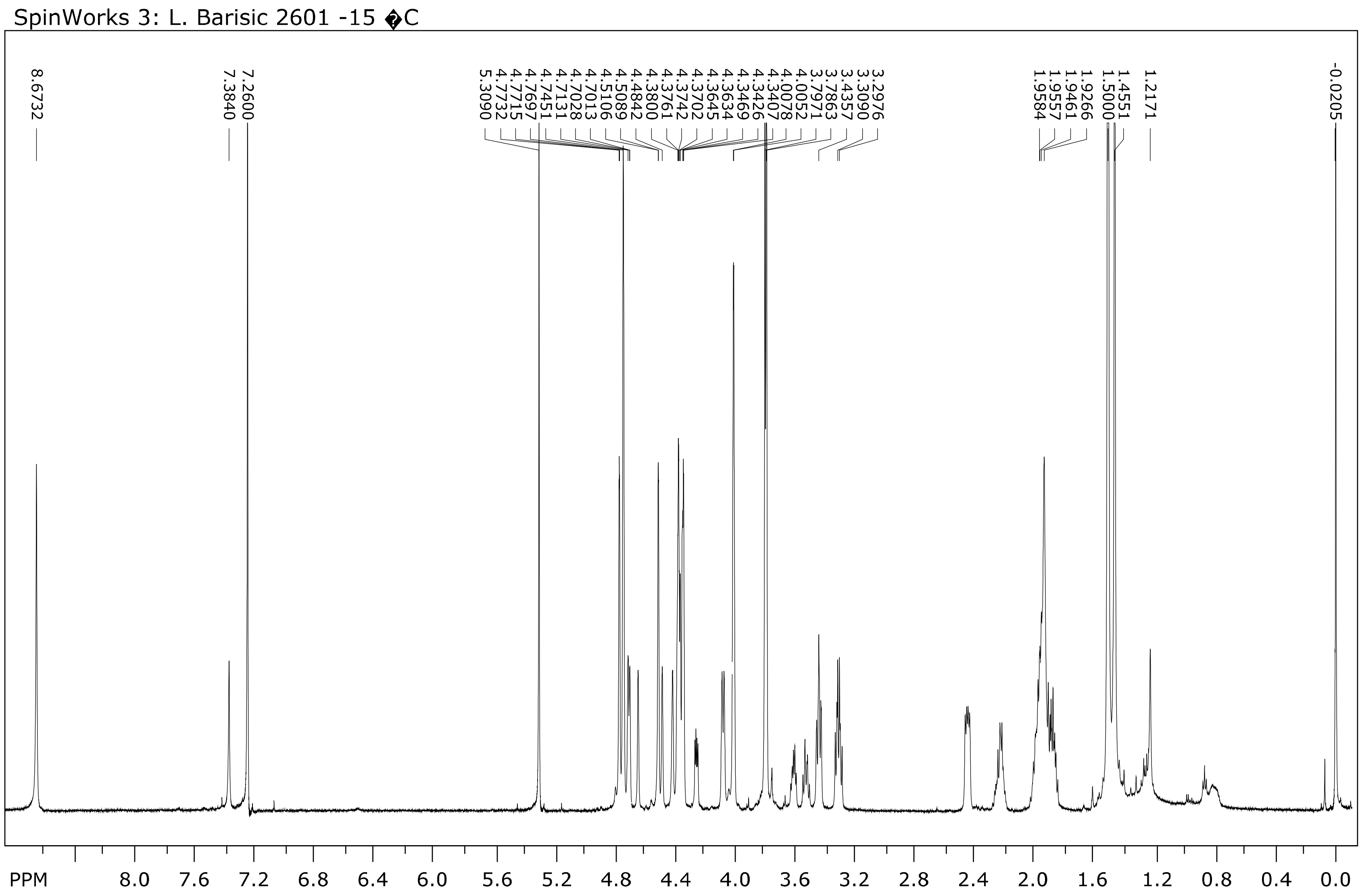
SpinWorks 3: L. Barisic 2601 -15 �C
8.6732
7.3840
7.2600
5.3090
4.7732
4.7715
4.7697
4.7451
4.7131
4.7028
4.7013
4.5106
4.5089
4.4842
4.3800
4.3761
4.3742
4.3702
4.3645
4.3634
4.3469
4.3426
4.3407
4.0078
4.0052
3.7971
3.7863
3.4357
3.3090
3.2976
1.9584
1.9557
1.9461
1.9266
1.5000
1.4551
1.2171
-0.0205
PPM
8.0
7.6
7.2
6.8
6.4
6.0
5.6
5.2
4.8
4.4
4.0
3.6
3.2
2.8
2.4
2.0
1.6
1.2
0.8
0.4
0.0

Compound 4

Figure S16. MALDI TOF/TOF-MS spectrum of **4**.

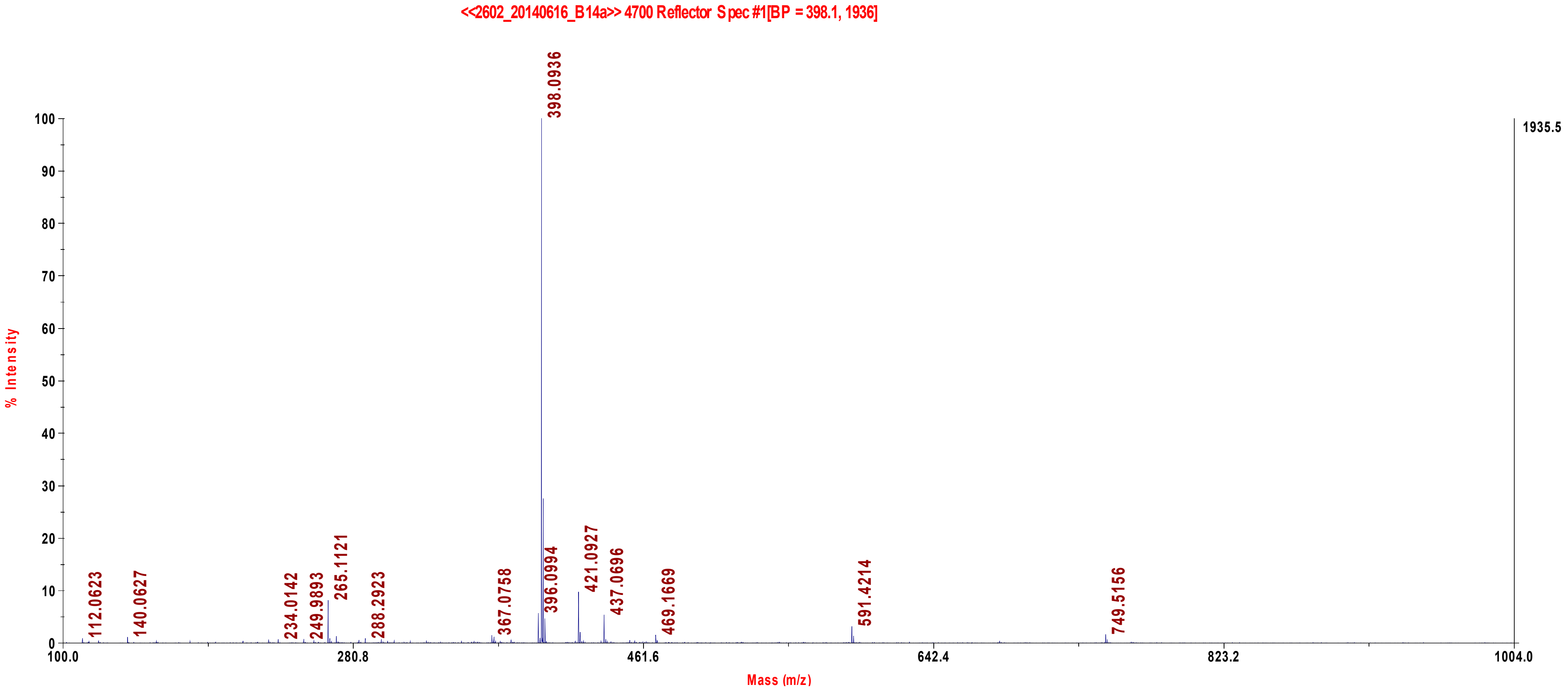

Figure S17. ^{1}H NMR spectra of **4.**

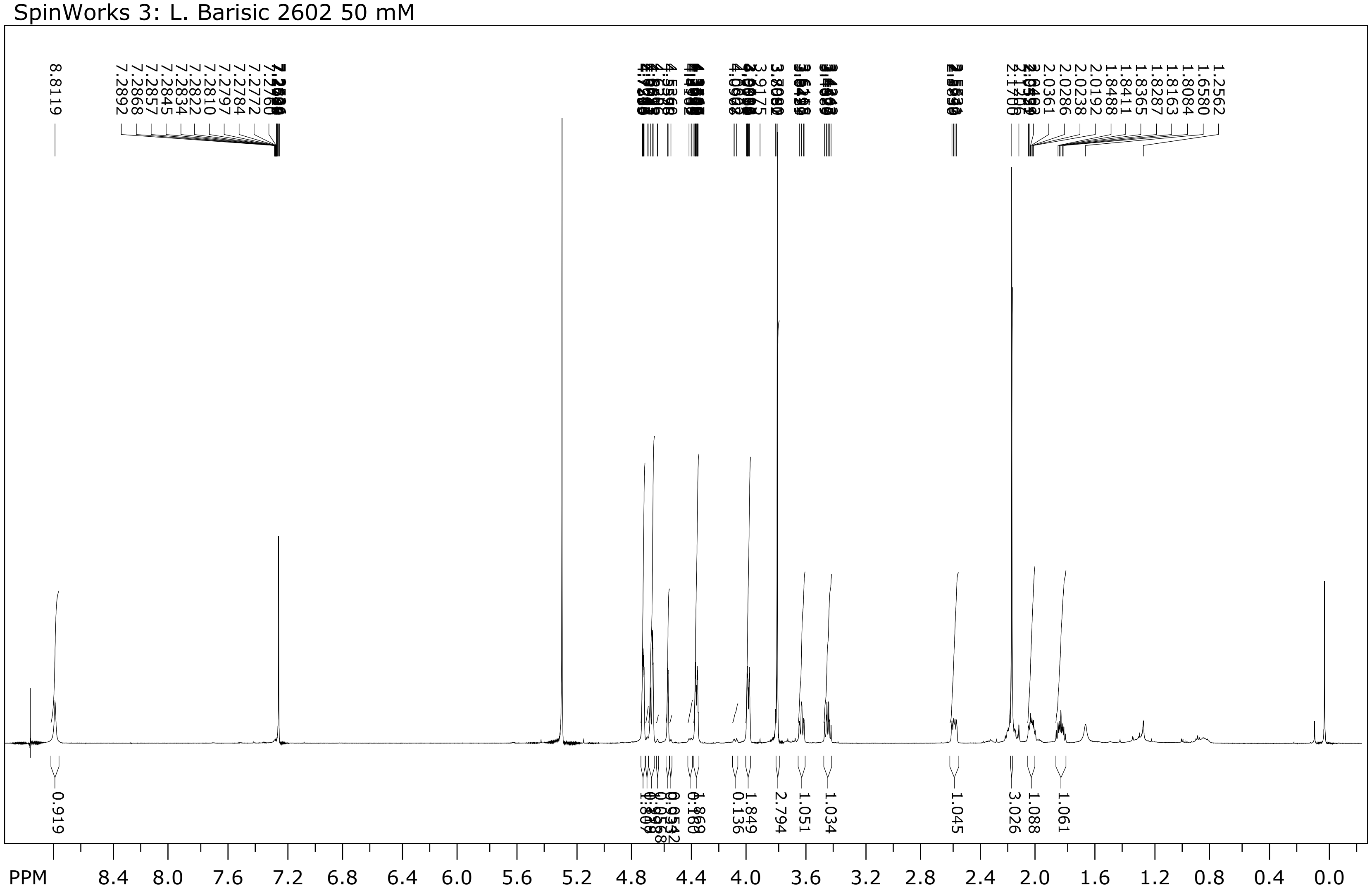

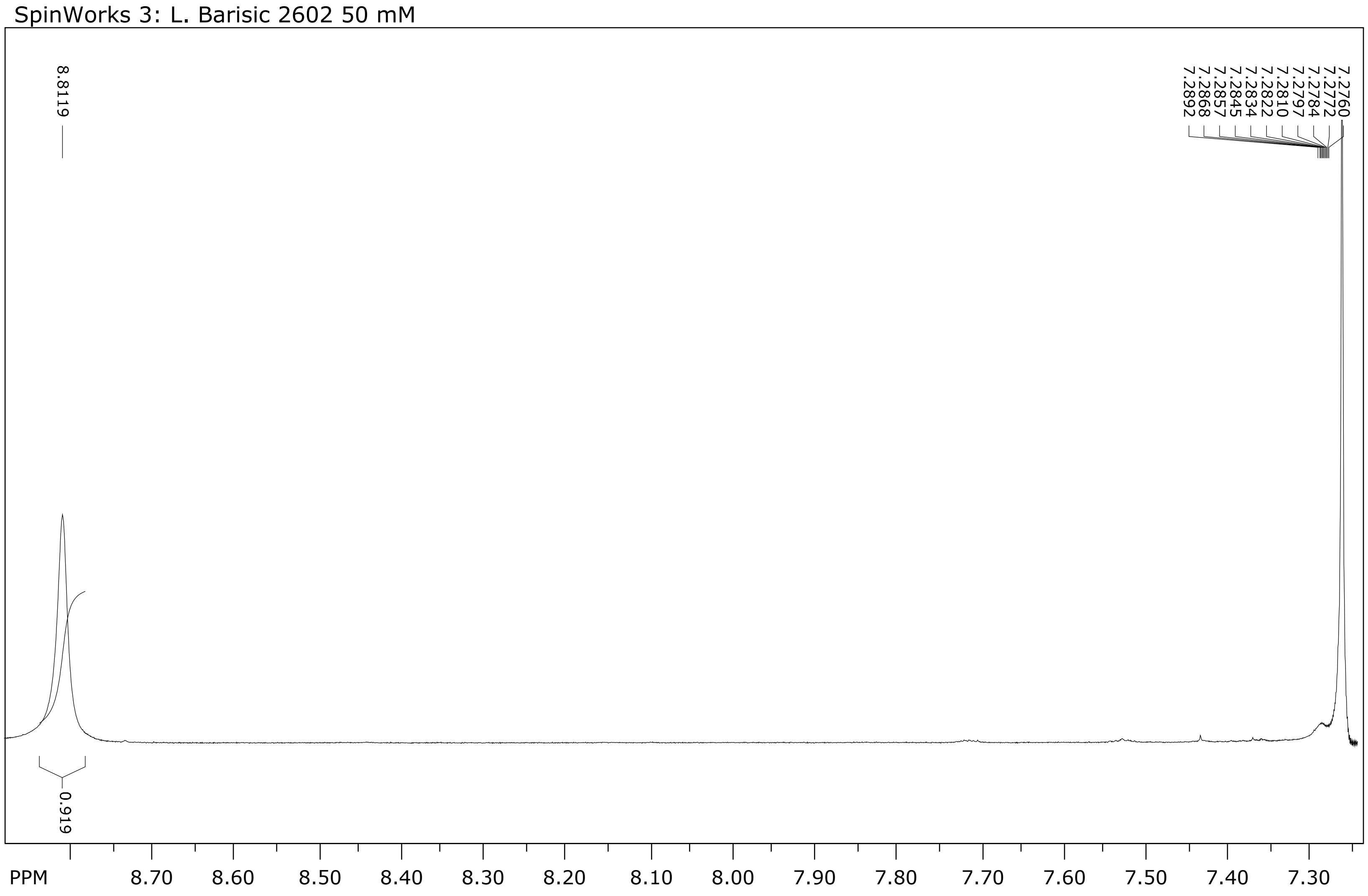
SpinWorks 3: L. Barisic 2602 50 mM
8.8119
7.2892
7.2868
7.2857
7.2845
7.2834
7.2822
7.2810
7.2797
7.2784
7.2772
7.2760
0.919
PPM
8.70
8.60
8.50
8.40
8.30
8.20
8.10
8.00
7.90
7.80
7.70
7.60
7.50
7.40
7.30

SpinWorks 3: L. Barisic 2602 50 mM

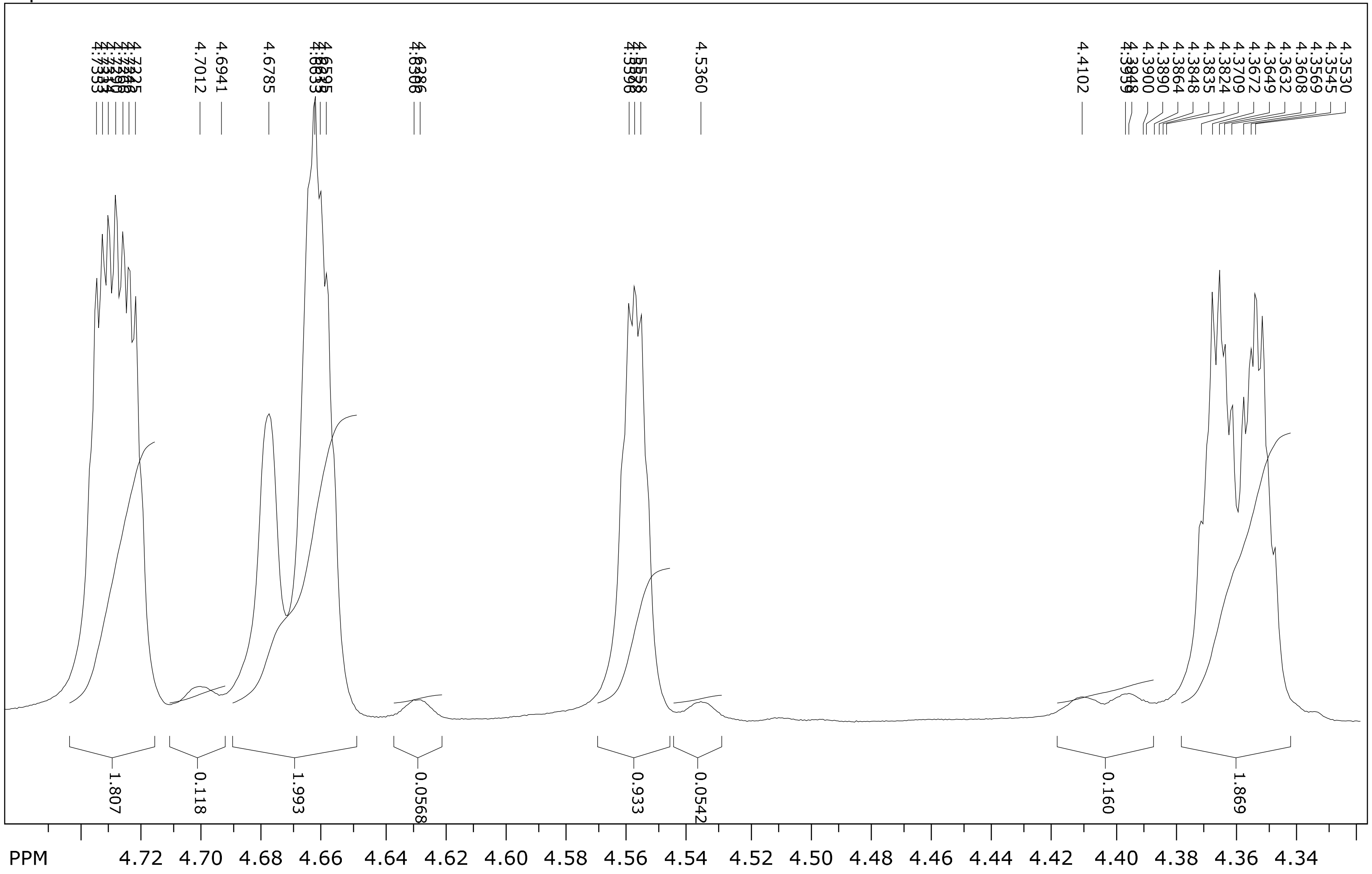

SpinWorks 3: L. Barisic 2602 50 mM

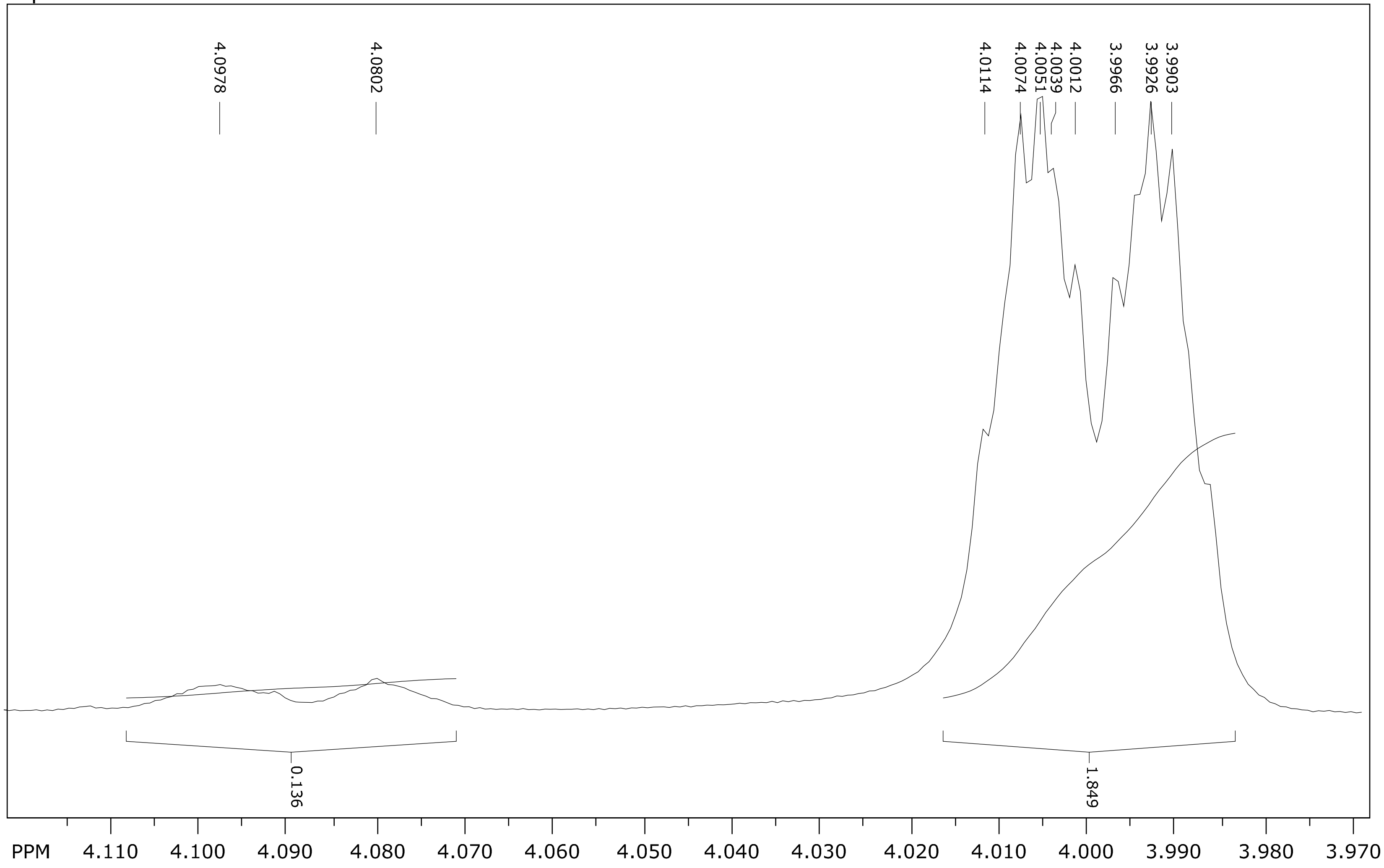

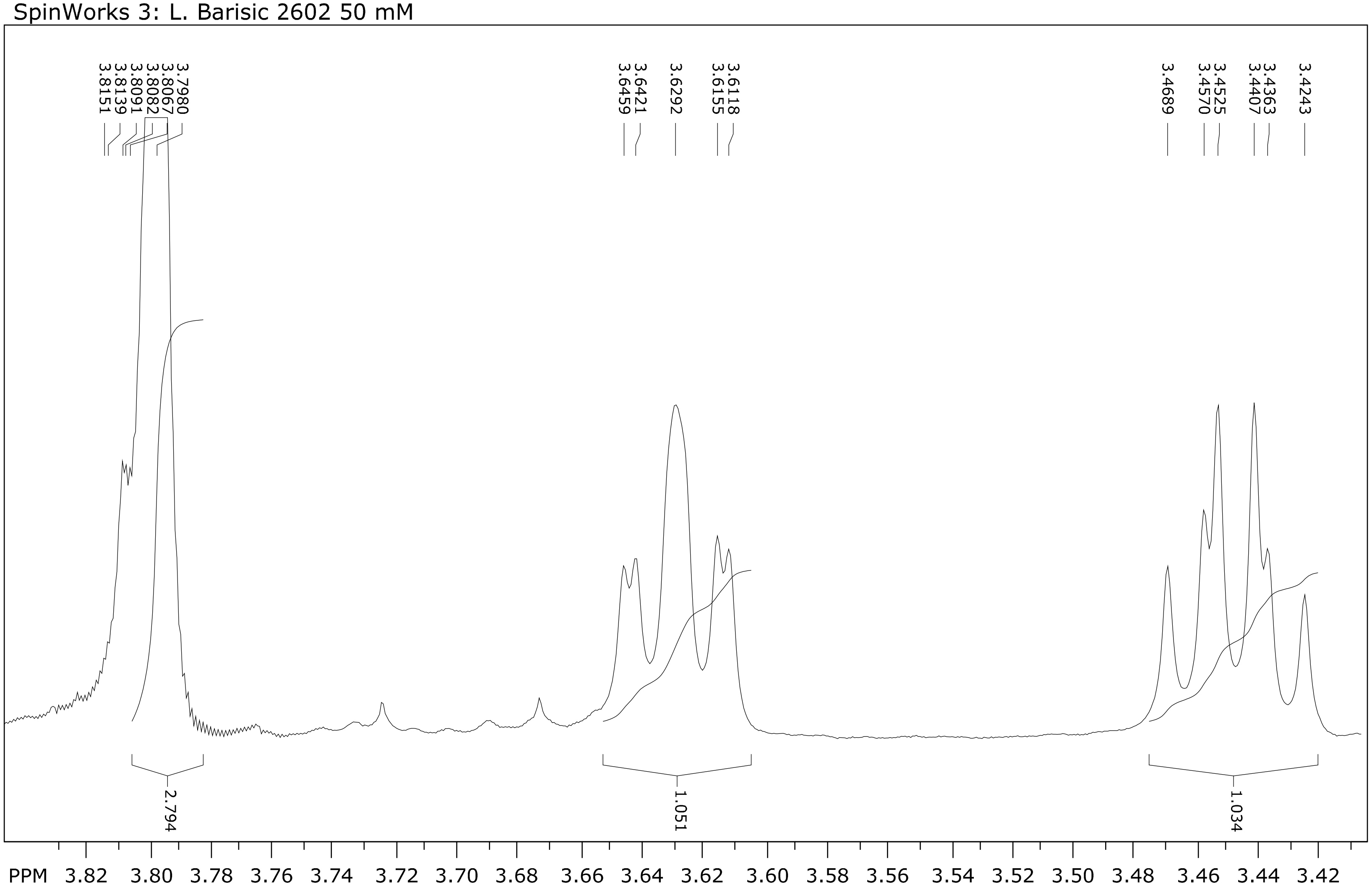
SpinWorks 3: L. Barisic 2602 50 mM
3.8151
3.8139
3.8091
3.8082
3.8067
3.7980
3.6459
3.6421
3.6292
3.6155
3.6118
3.4689
3.4570
3.4525
3.4407
3.4363
3.4243
2.794
1.051
1.034
PPM 3.82 3.80 3.78 3.76 3.74 3.72 3.70 3.68 3.66 3.64 3.62 3.60 3.58 3.56 3.54 3.52 3.50 3.48 3.46 3.44 3.42

Figure S18. ^{13}C NMR spectra of **4**.

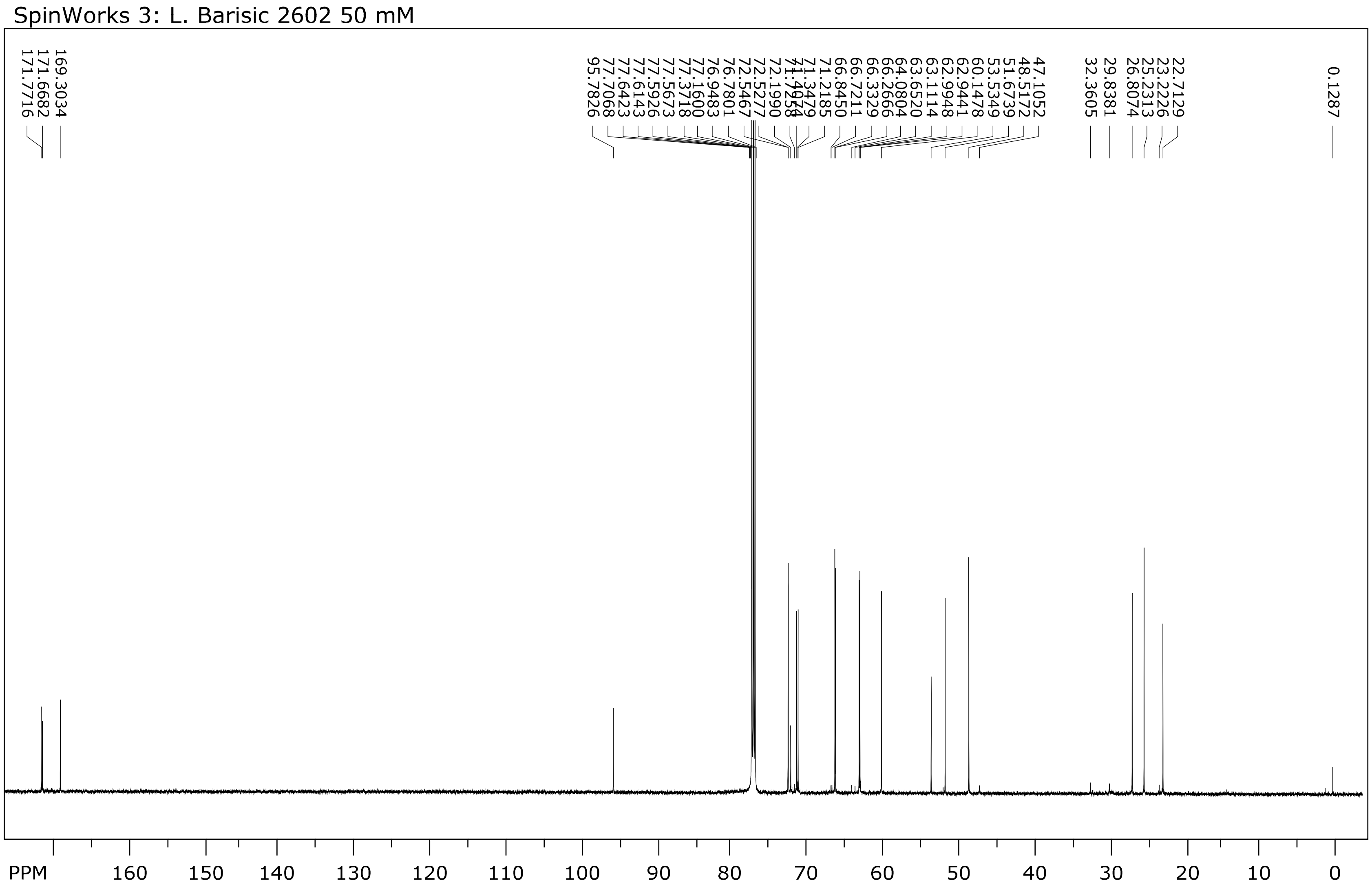

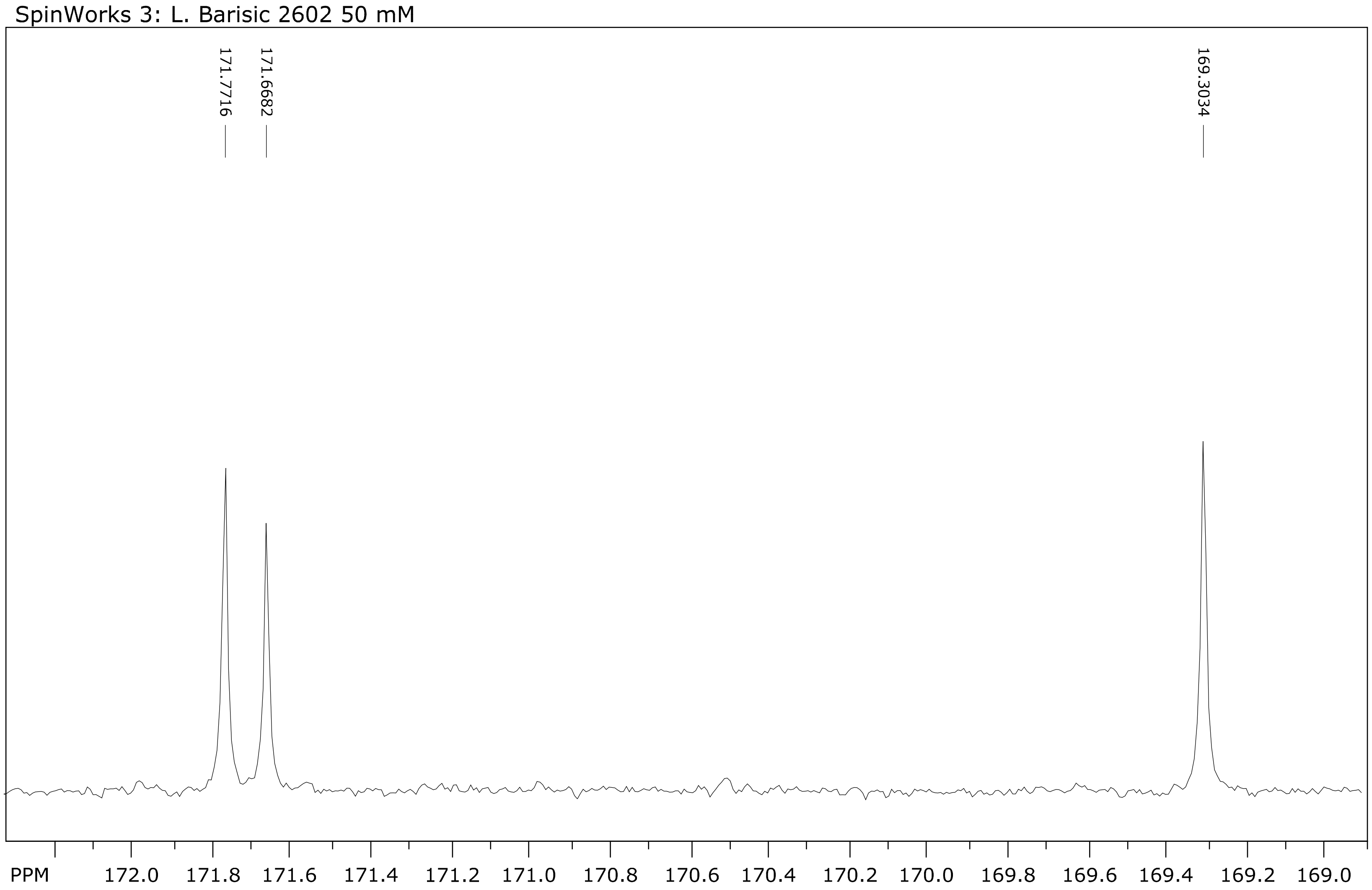
SpinWorks 3: L. Barisic 2602 50 mM
171.7716
171.6682
169.3034
PPM
172.0
171.8
171.6
171.4
171.2
171.0
170.8
170.6
170.4
170.2
170.0
169.8
169.6
169.4
169.2
169.0

SpinWorks 3: L. Barisic 2602 50 mM

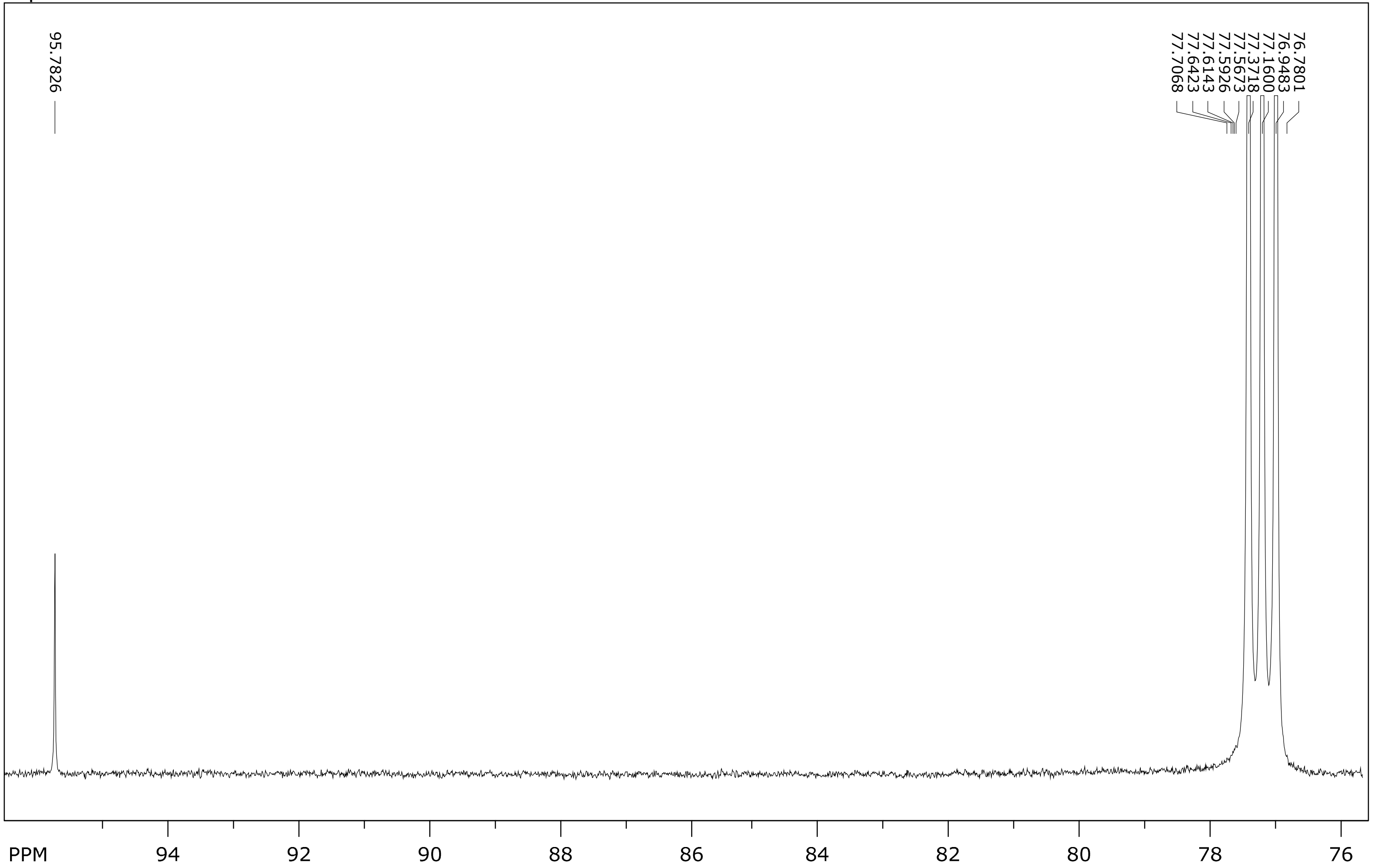

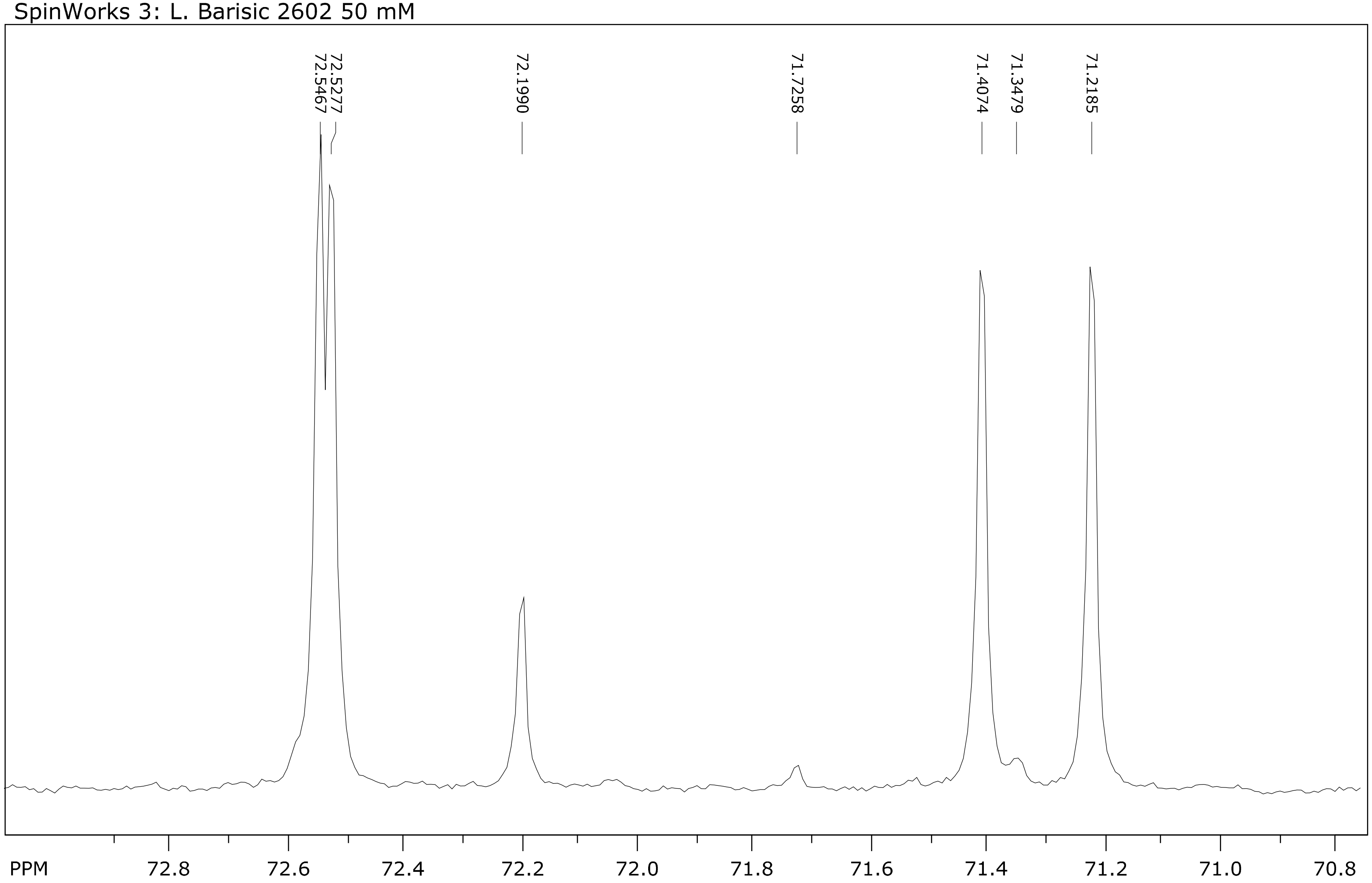
SpinWorks 3: L. Barisic 2602 50 mM
72.5467
72.5277
72.1990
71.7258
71.4074
71.3479
71.2185
PPM
72.8
72.6
72.4
72.2
72.0
71.8
71.6
71.4
71.2
71.0
70.8

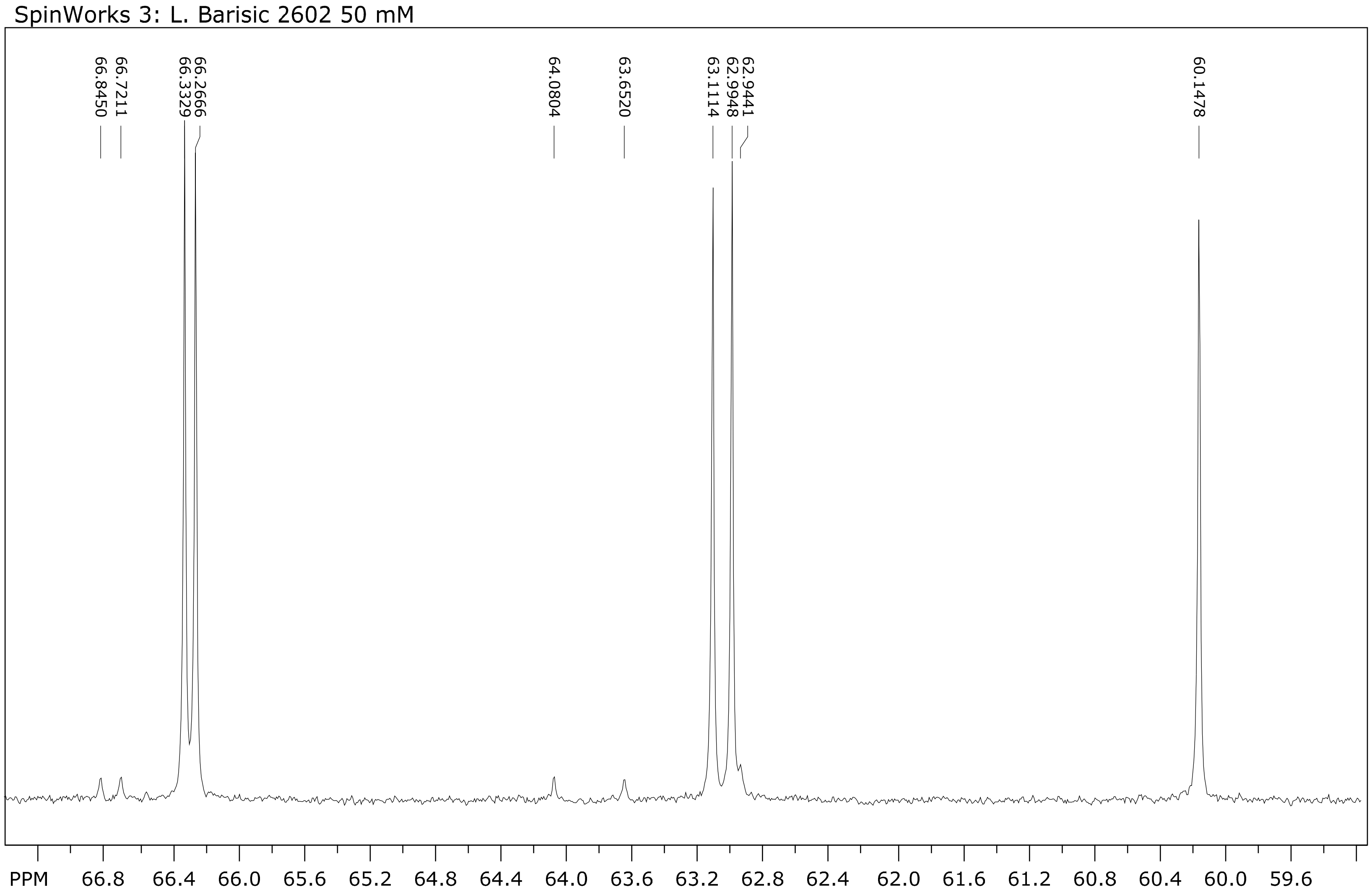
SpinWorks 3: L. Barisic 2602 50 mM
66.8450
66.7211
66.3329
66.2666
64.0804
63.6520
63.1114
62.9948
62.9441
60.1478
PPM
66.8
66.4
66.0
65.6
65.2
64.8
64.4
64.0
63.6
63.2
62.8
62.4
62.0
61.6
61.2
60.8
60.4
60.0
59.6

SpinWorks 3: L. Barisic 2602 50 mM

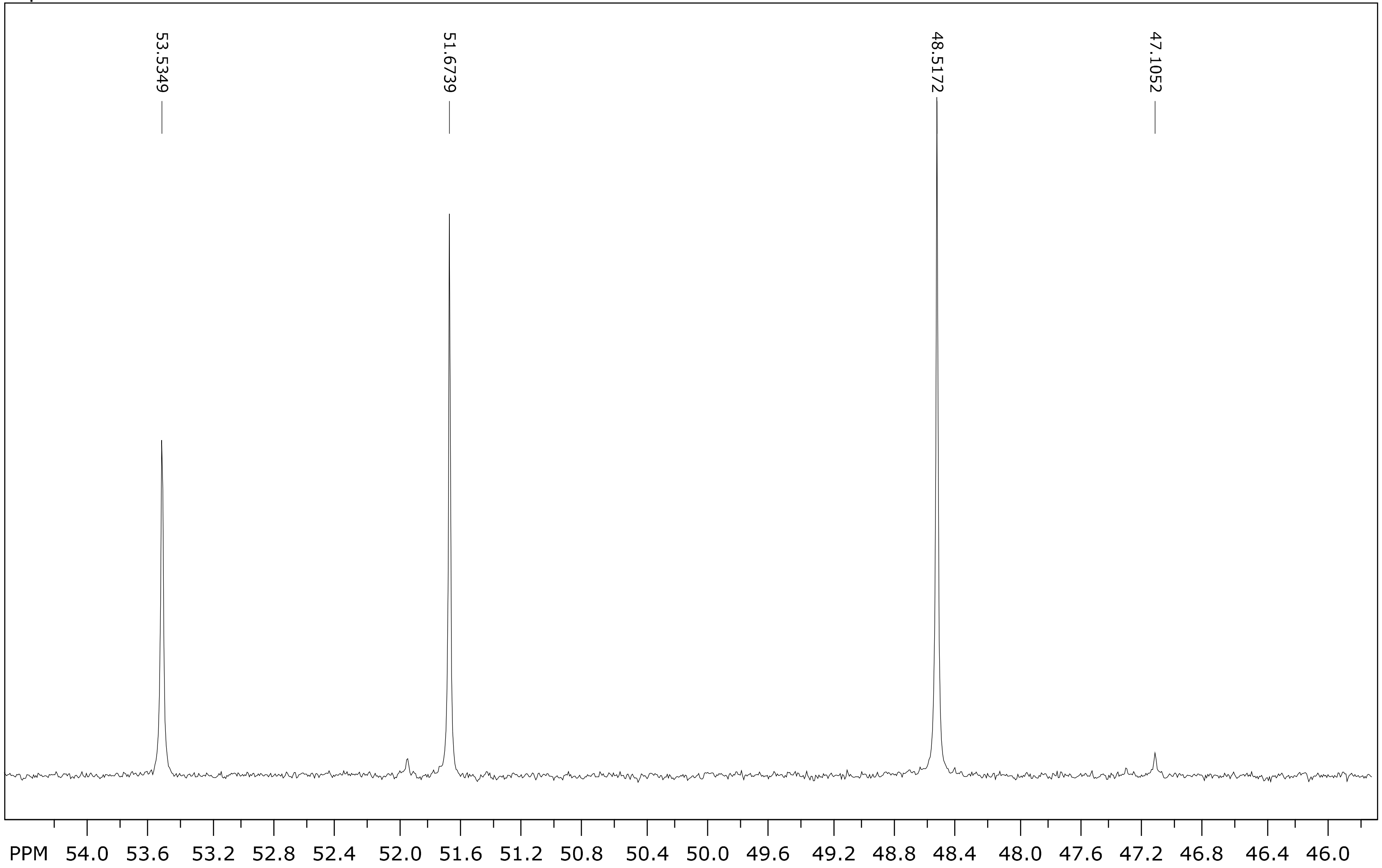

SpinWorks 3: L. Barisic 2602 50 mM

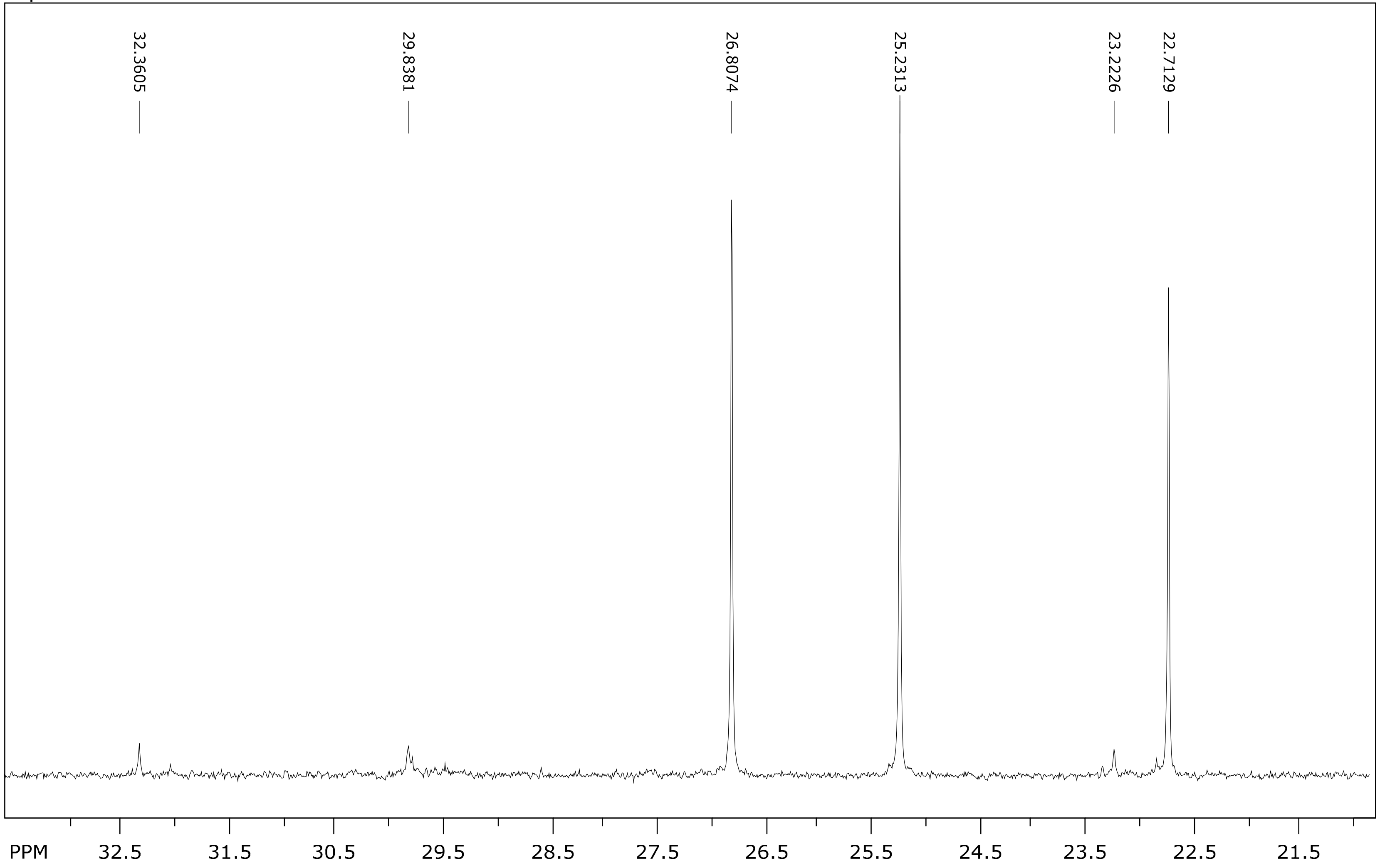

Figure S19. ^{1}H NMR titration of compound **1** with DMSO in $CDCl_3$.

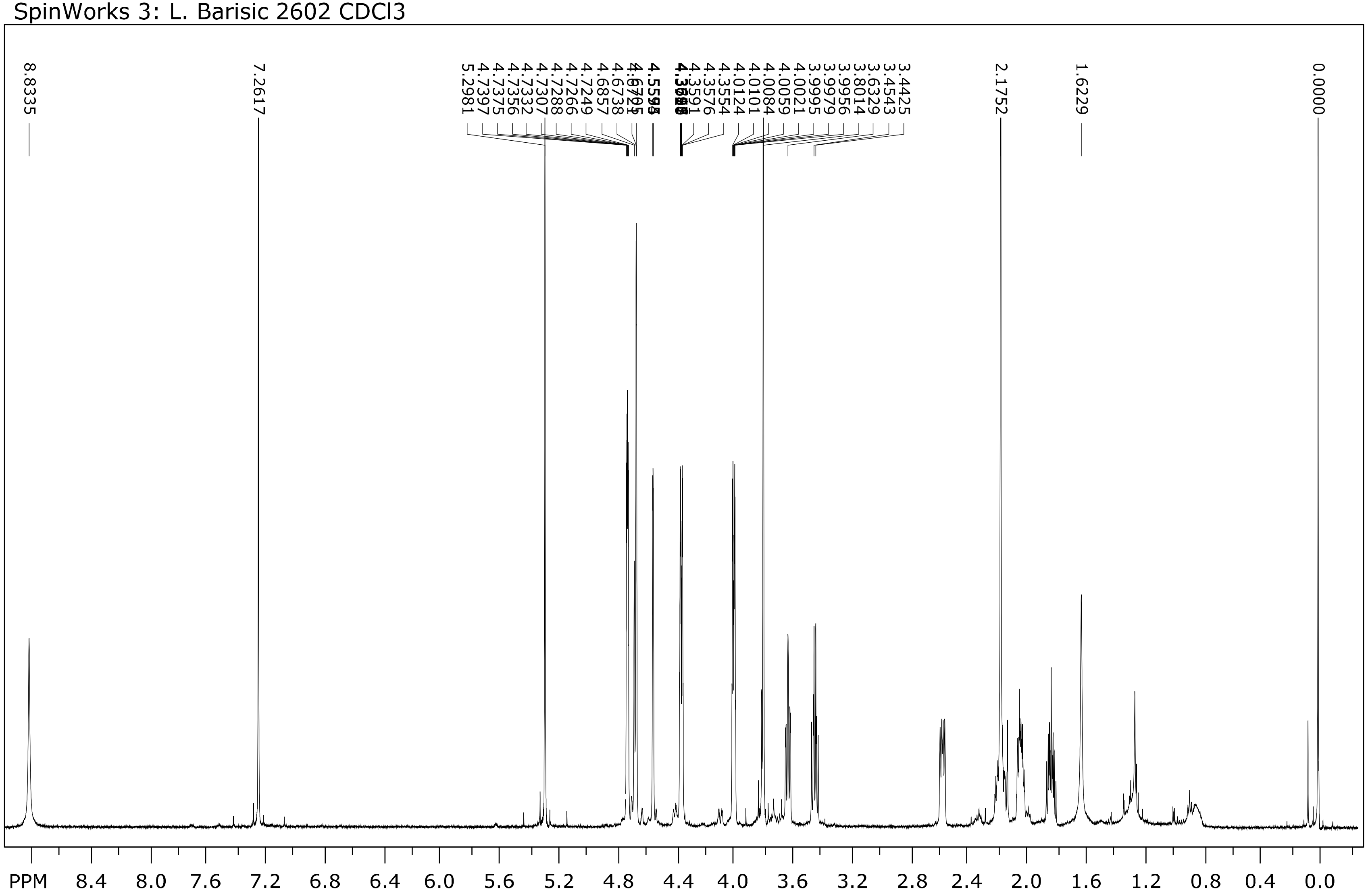

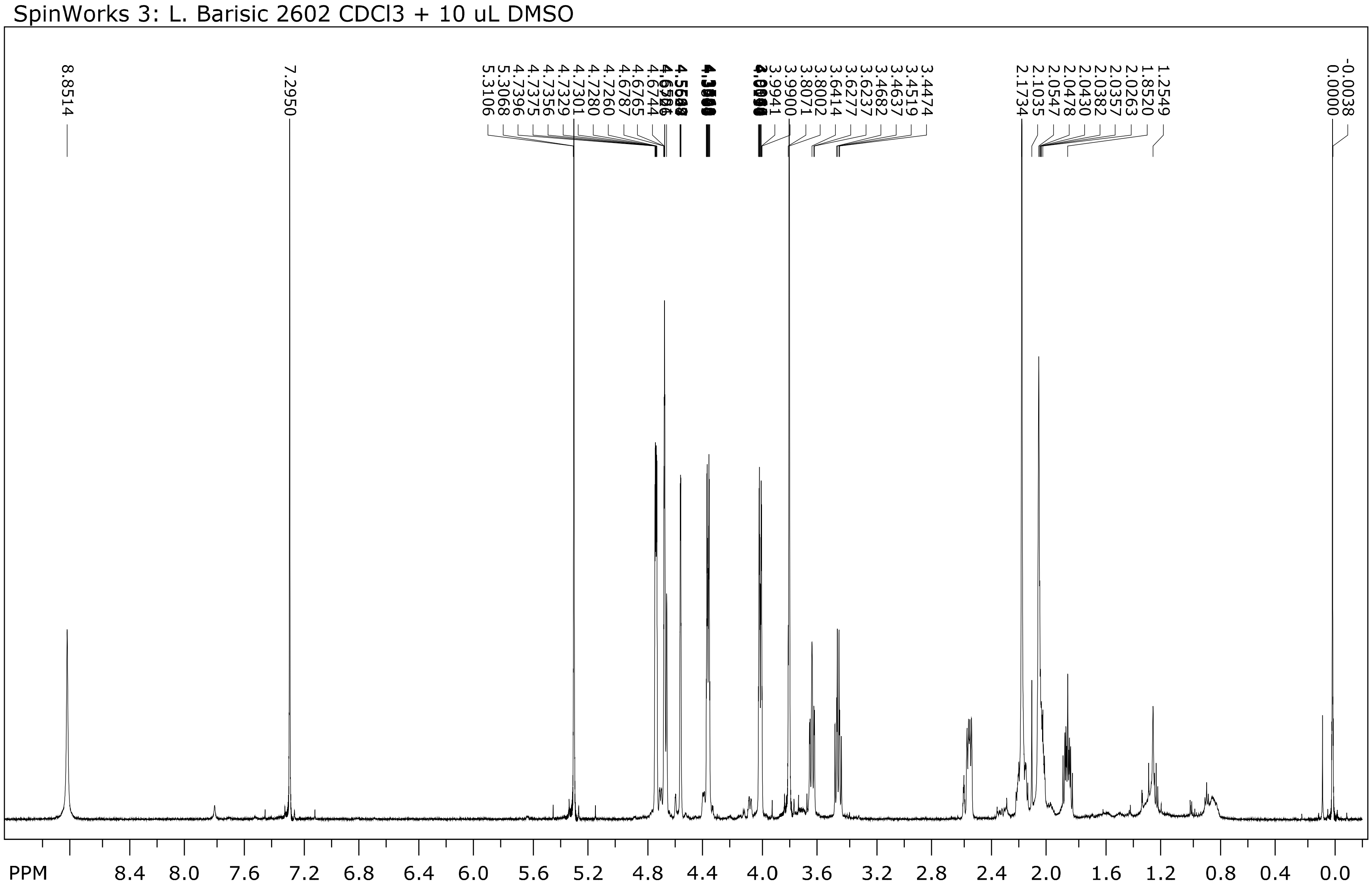
SpinWorks 3: L. Barisic 2602 CDCl3 + 10 uL DMSO
8.8514
7.2950
5.3106
5.3068
4.7396
4.7375
4.7356
4.7329
4.7301
4.7280
4.7260
4.6787
4.6765
4.6744
3.9941
3.9900
3.8071
3.8002
3.6414
3.6277
3.6237
3.4682
3.4637
3.4519
3.4474
2.1734
2.1035
2.0547
2.0478
2.0430
2.0382
2.0357
2.0263
1.8520
1.2549
0.0000
-0.0038
PPM
8.4 8.0 7.6 7.2 6.8 6.4 6.0 5.6 5.2 4.8 4.4 4.0 3.6 3.2 2.8 2.4 2.0 1.6 1.2 0.8 0.4 0.0

SpinWorks 3: L. Barisic 2602 CDCl3 + 20 uL DMSO

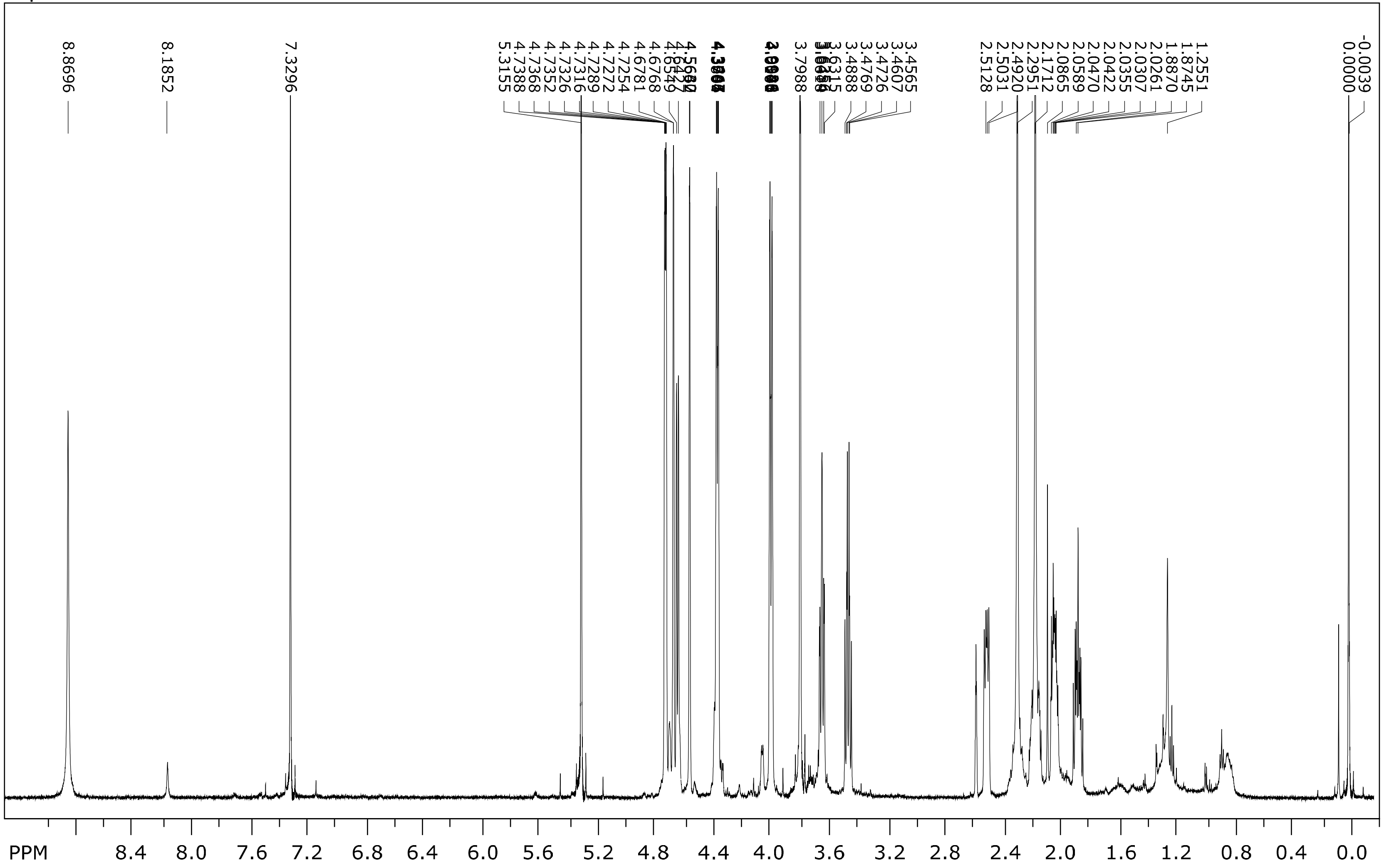

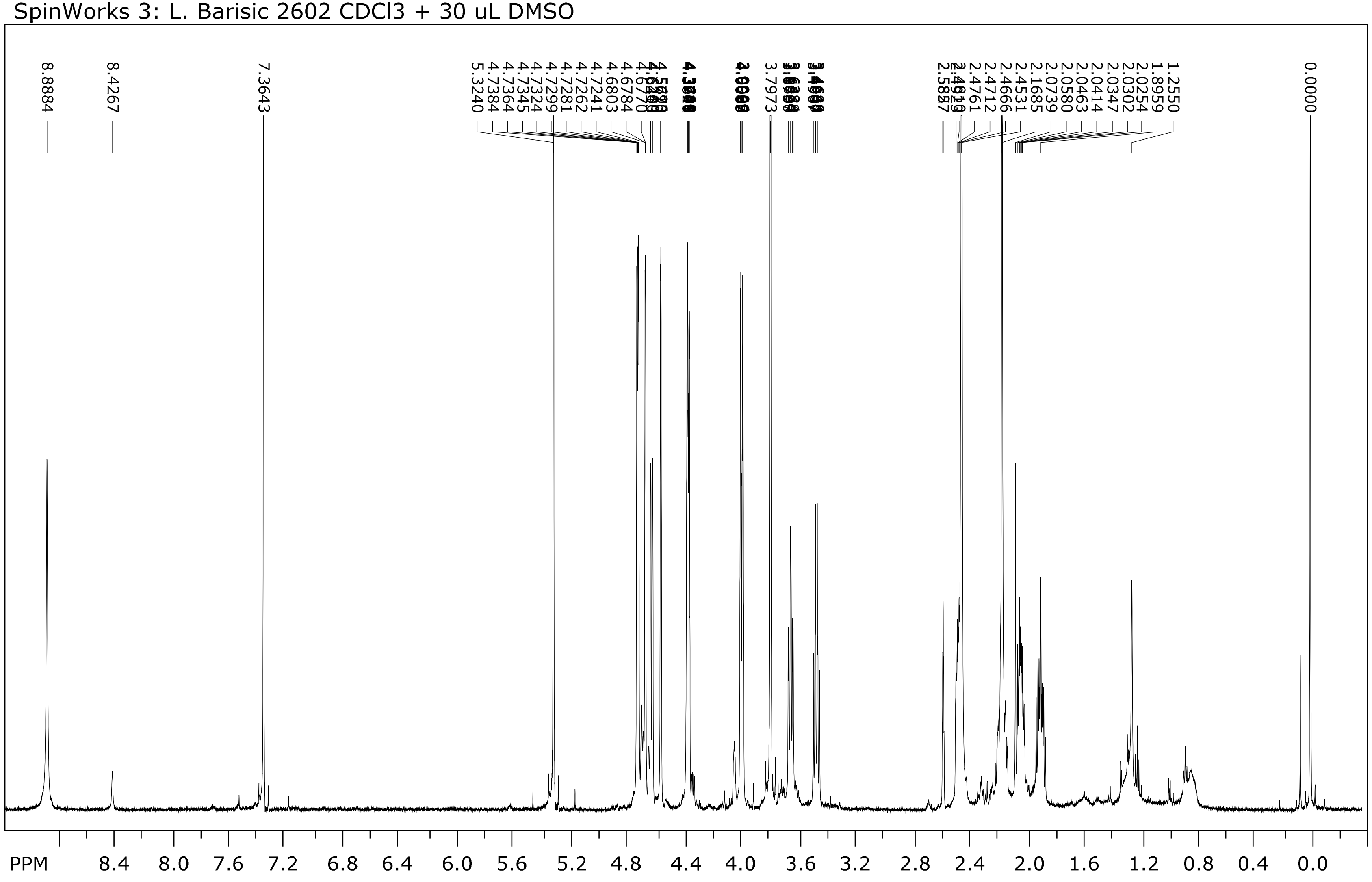
SpinWorks 3: L. Barisic 2602 CDCl3 + 30 uL DMSO
8.8884
8.4267
7.3643
5.3240
4.7384
4.7364
4.7345
4.7324
4.7299
4.7281
4.7262
4.7241
4.6803
4.6784
4.6770
3.7973
2.5857
2.4666
2.4531
2.1685
2.0739
2.0580
2.0463
2.0414
2.0347
2.0302
2.0254
1.8959
1.2550
0.0000
PPM
8.4
8.0
7.6
7.2
6.8
6.4
6.0
5.6
5.2
4.8
4.4
4.0
3.6
3.2
2.8
2.4
2.0
1.6
1.2
0.8
0.4
0.0

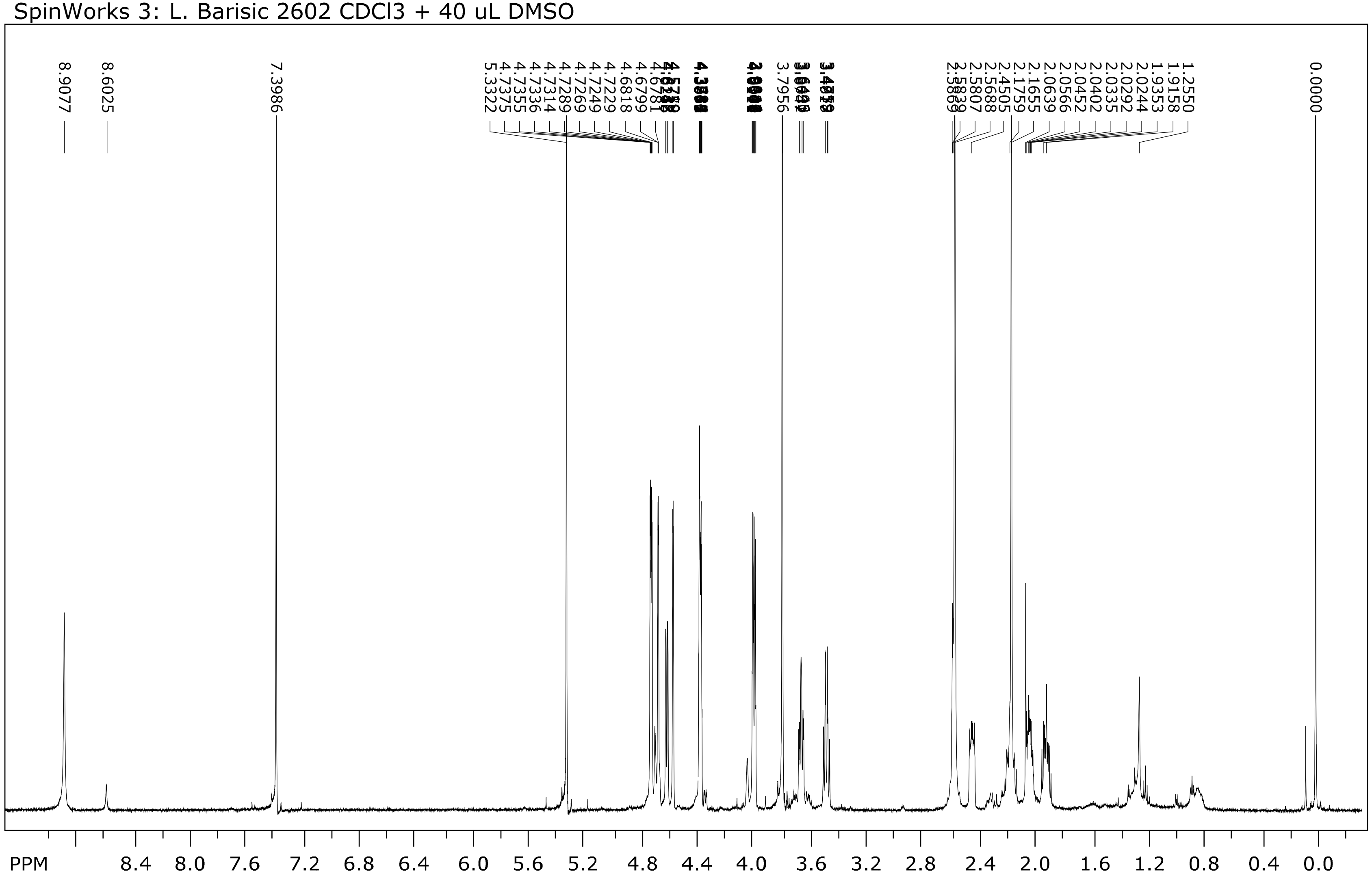
SpinWorks 3: L. Barisic 2602 CDCl3 + 40 uL DMSO
PPM

SpinWorks 3: L. Barisic 2602 CDCl3 + 50 uL DMSO

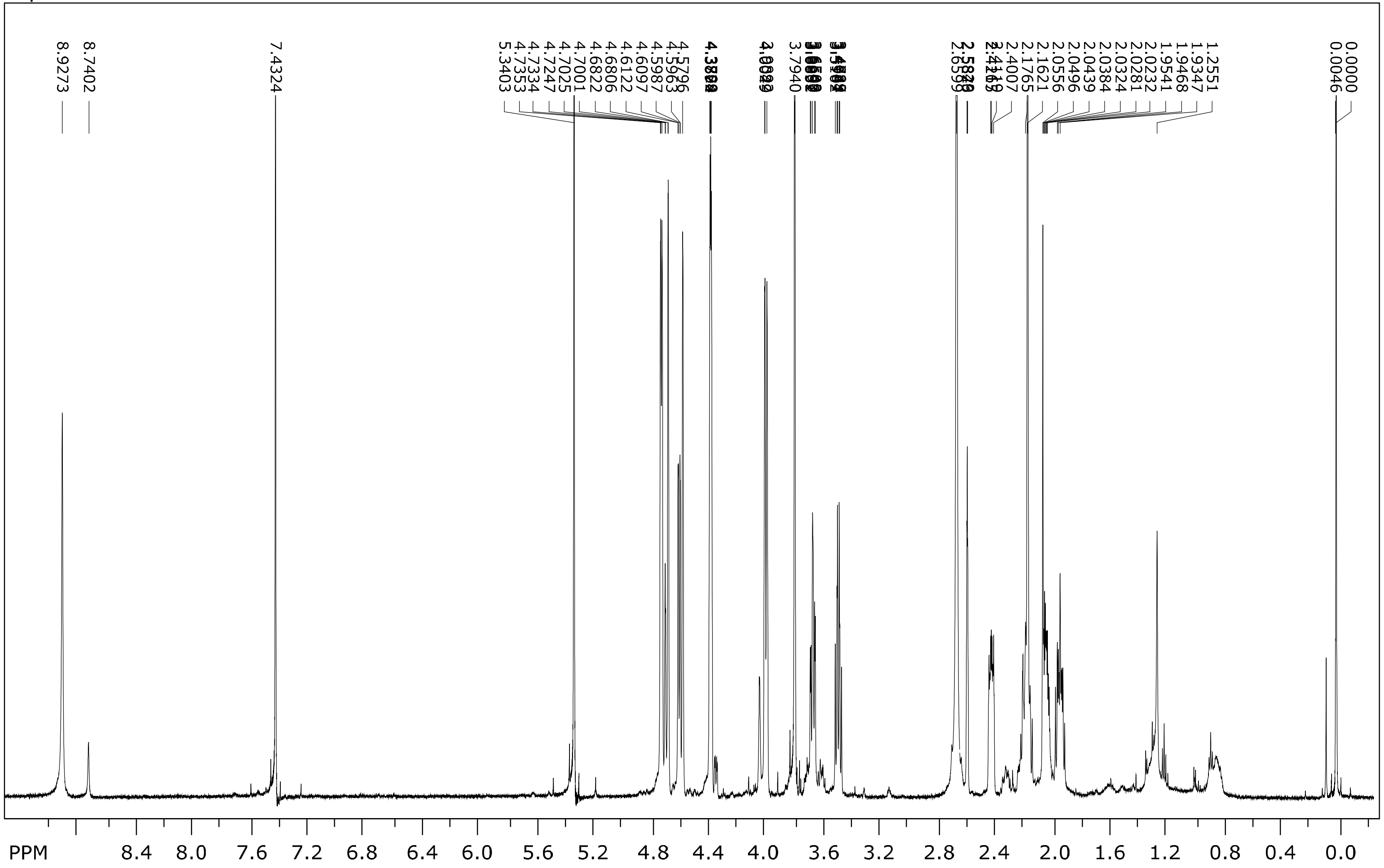

SpinWorks 3: L. Barisic 2602 CDCl3 + 70 uL DMSO

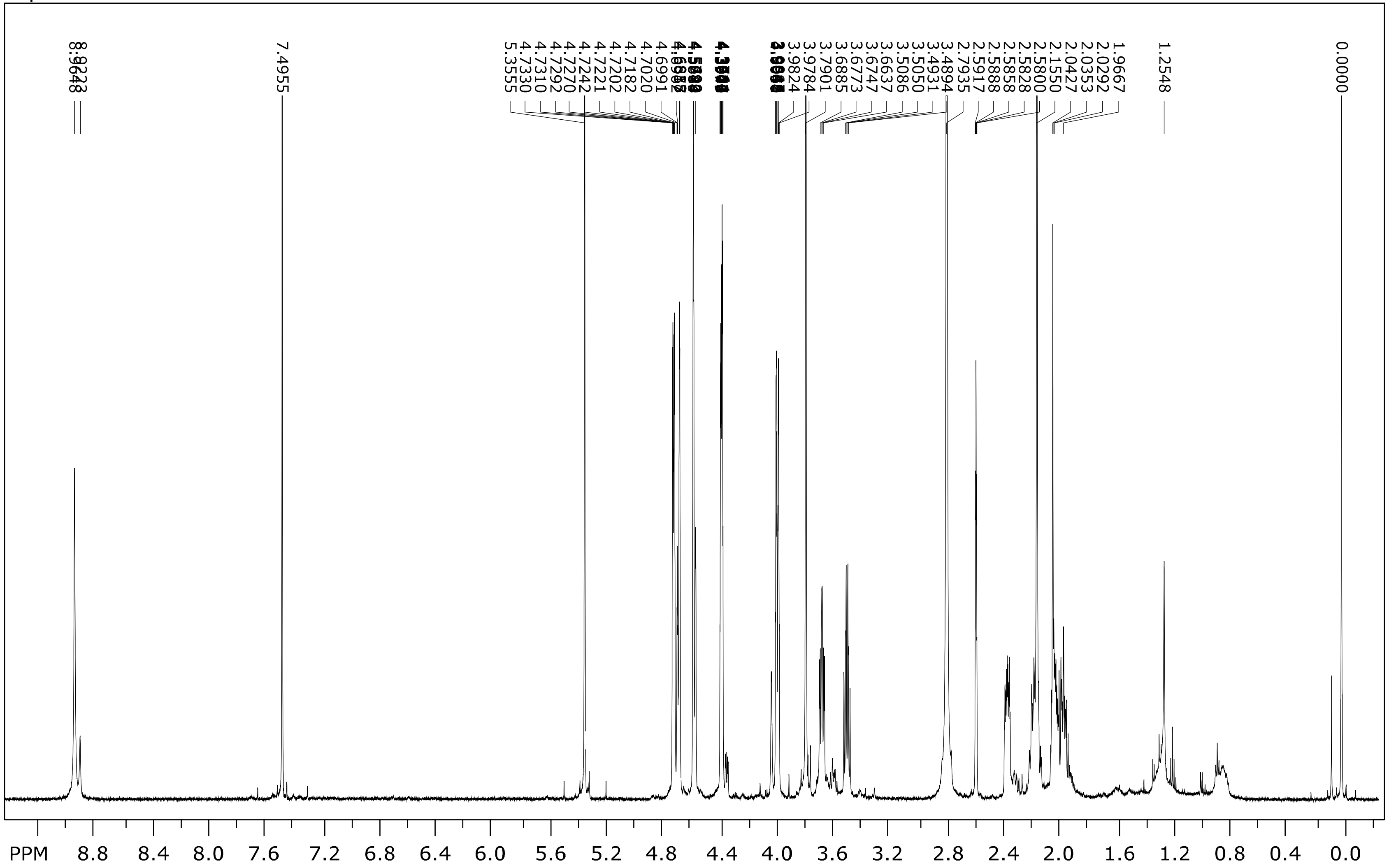

SpinWorks 3: L. Barisic 2602 CDCl3 + 90 uL DMSO

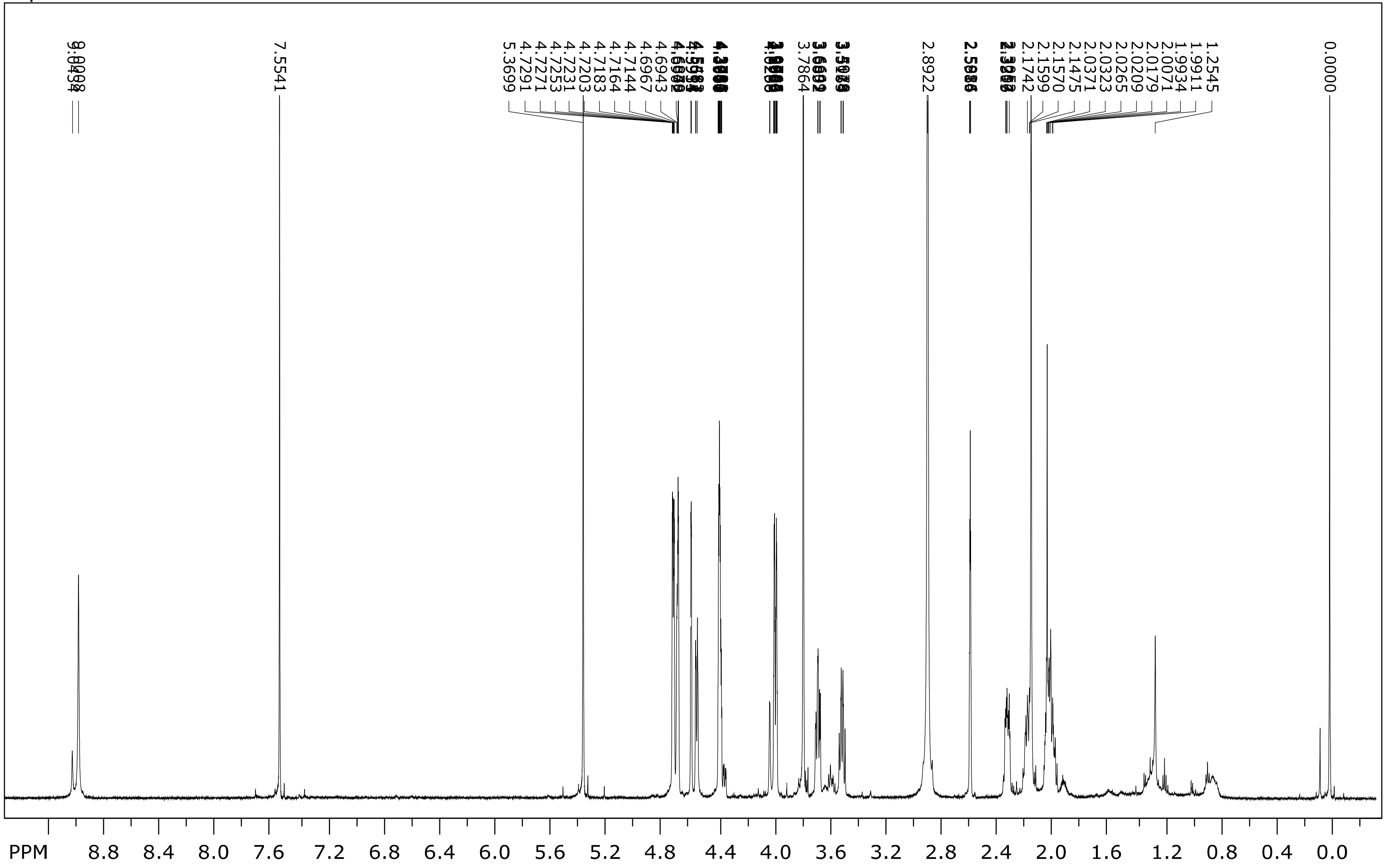

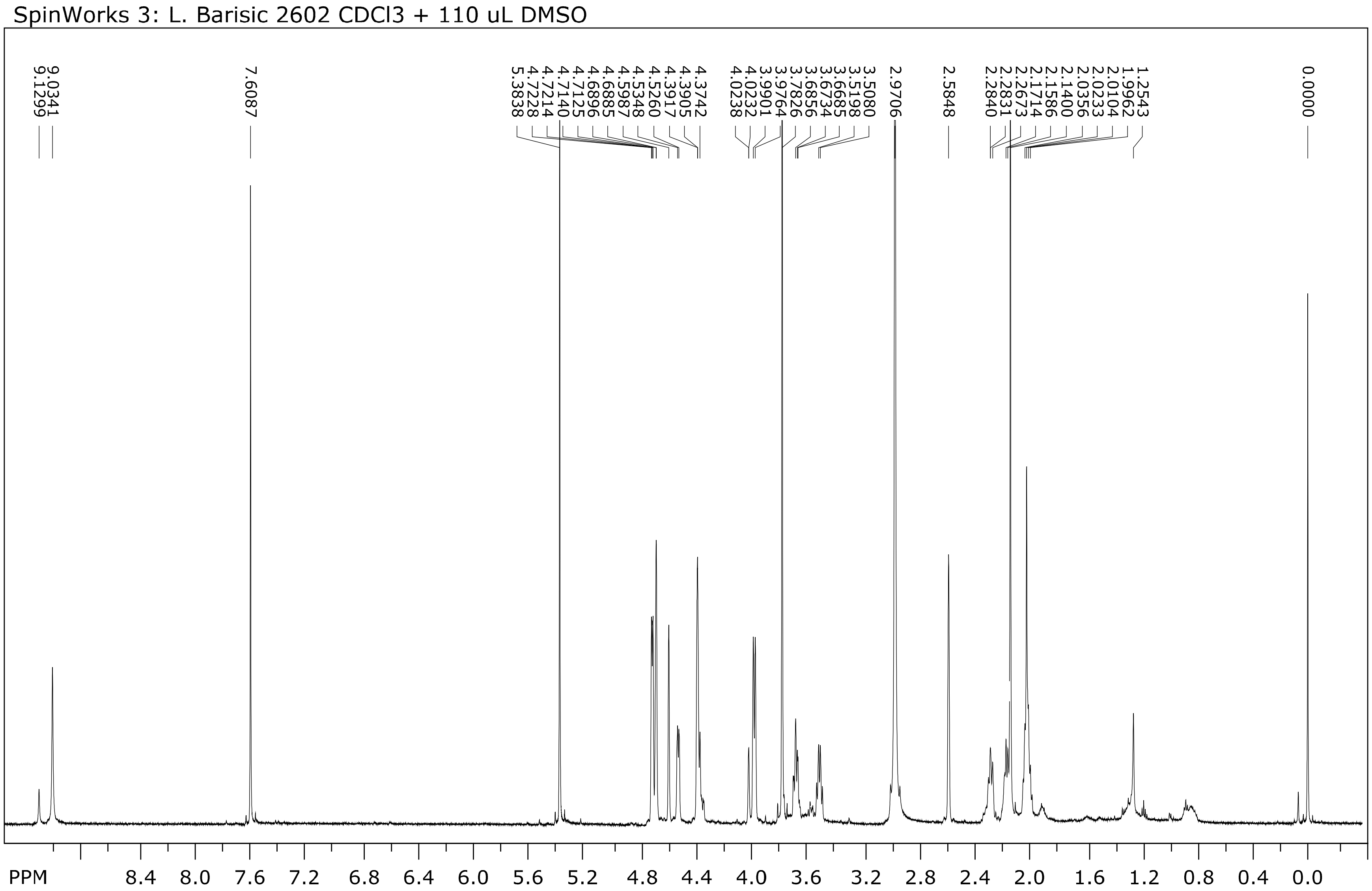
SpinWorks 3: L. Barisic 2602 CDCl3 + 110 uL DMSO
9.1299
9.0341
7.6087
5.3838
4.7228
4.7214
4.7140
4.7125
4.6896
4.6885
4.5987
4.5348
4.5260
4.3917
4.3905
4.3742
4.0238
4.0232
3.9901
3.9764
3.7826
3.6856
3.6734
3.6685
3.5198
3.5080
2.9706
2.5848
2.2840
2.2831
2.2673
2.1714
2.1586
2.1400
2.0356
2.0233
2.0104
1.9962
1.2543
0.0000
PPM
8.4
8.0
7.6
7.2
6.8
6.4
6.0
5.6
5.2
4.8
4.4
4.0
3.6
3.2
2.8
2.4
2.0
1.6
1.2
0.8
0.4
0.0

SpinWorks 3: L. Barisic 2602 CDCl3 + 130 uL DMSO

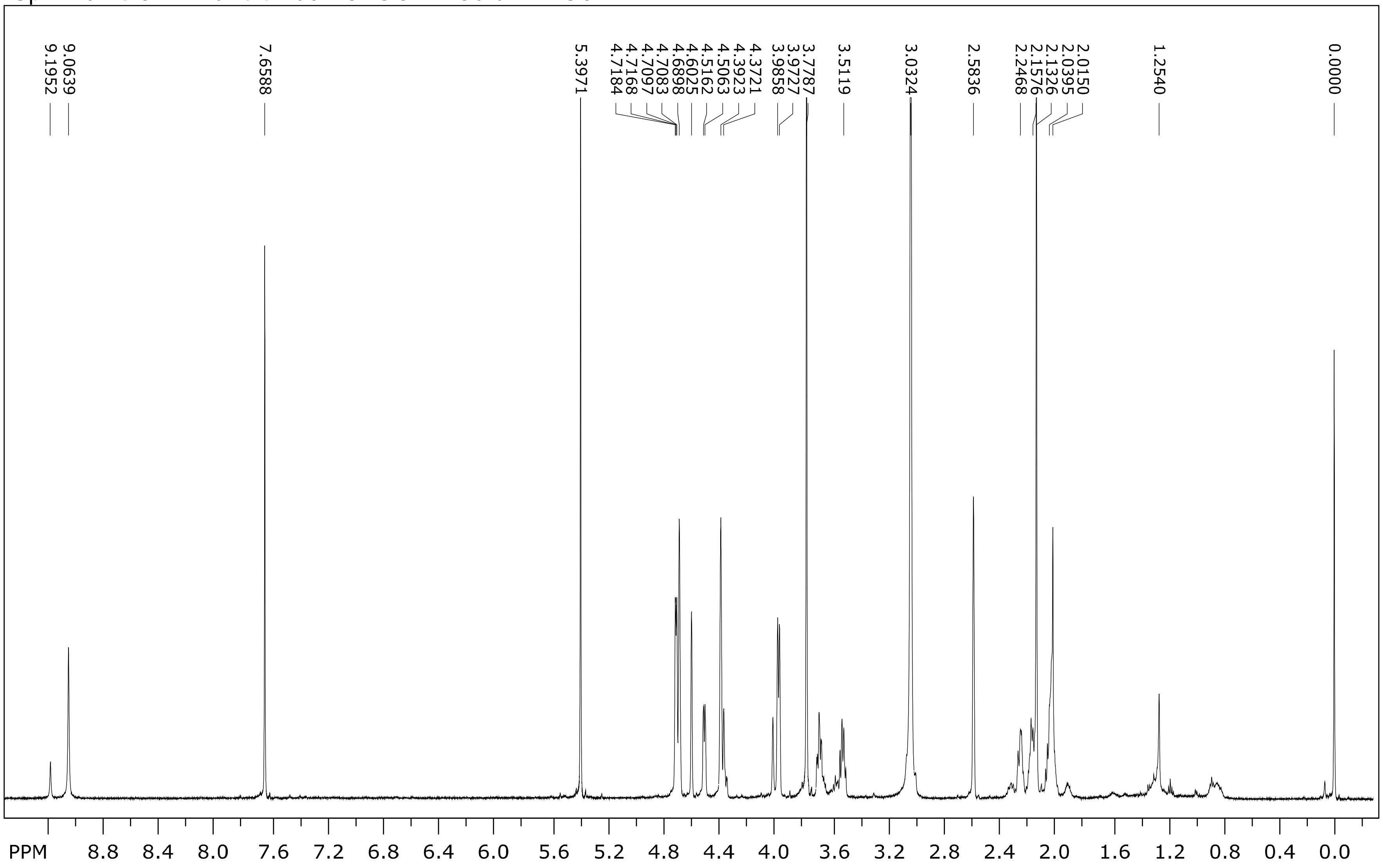

SpinWorks 3: L. Barisic 2602 CDCl3 + 180 uL DMSO

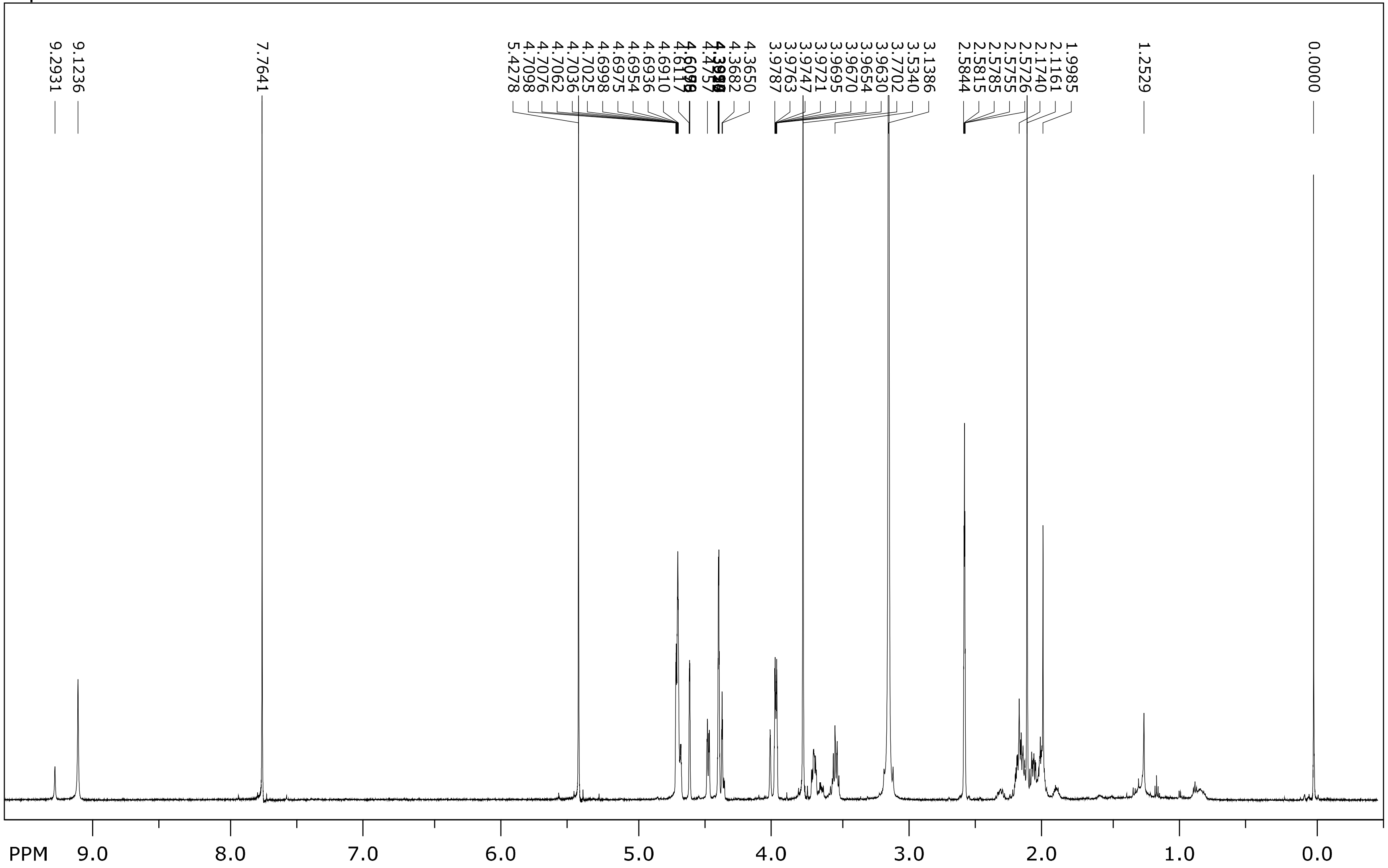

SpinWorks 3: L. Barisic 2602 CDCl3 + 280 uL DMSO

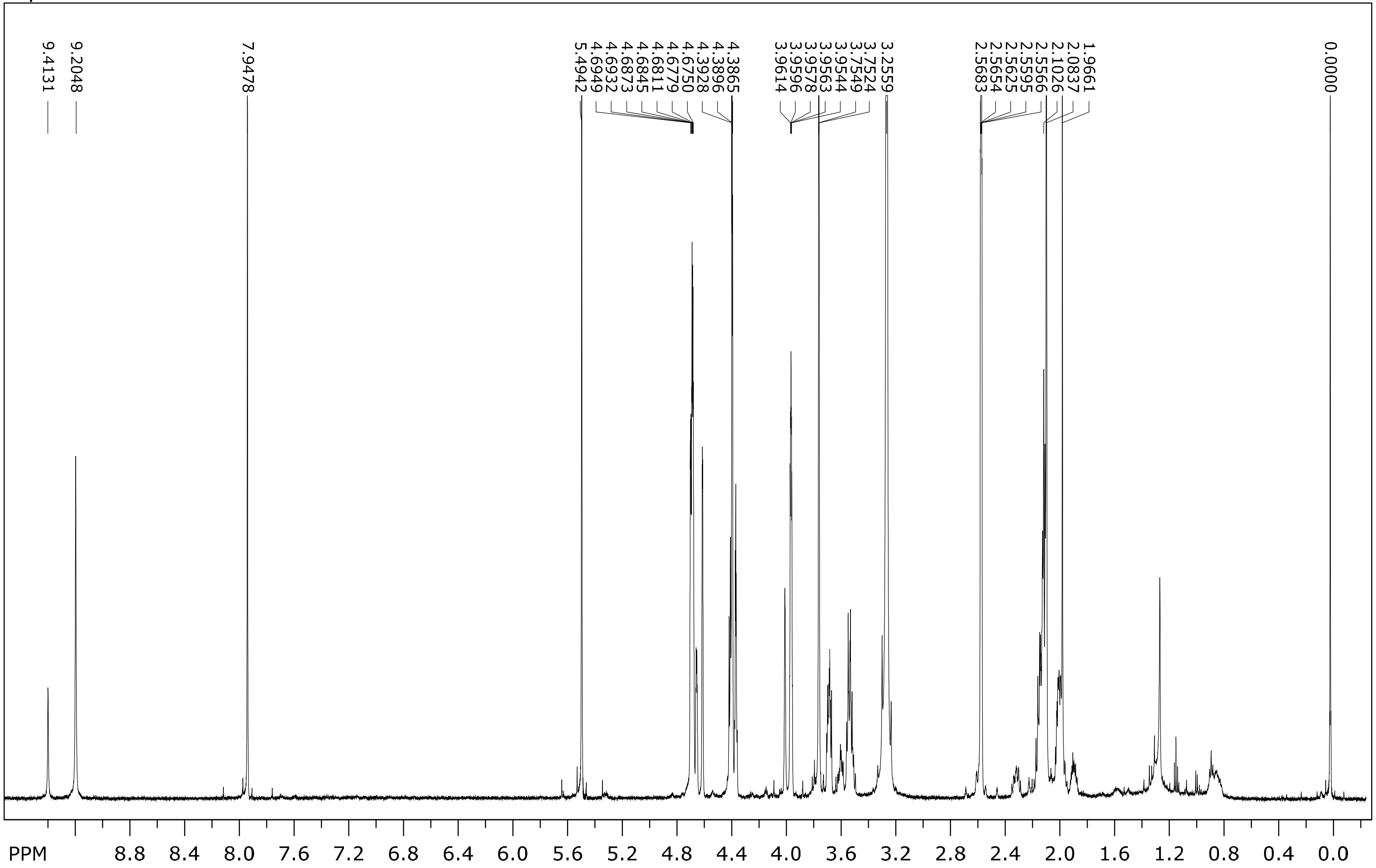

SpinWorks 3: L. Barisic 2602 CDCl3 + 430 uL DMSO

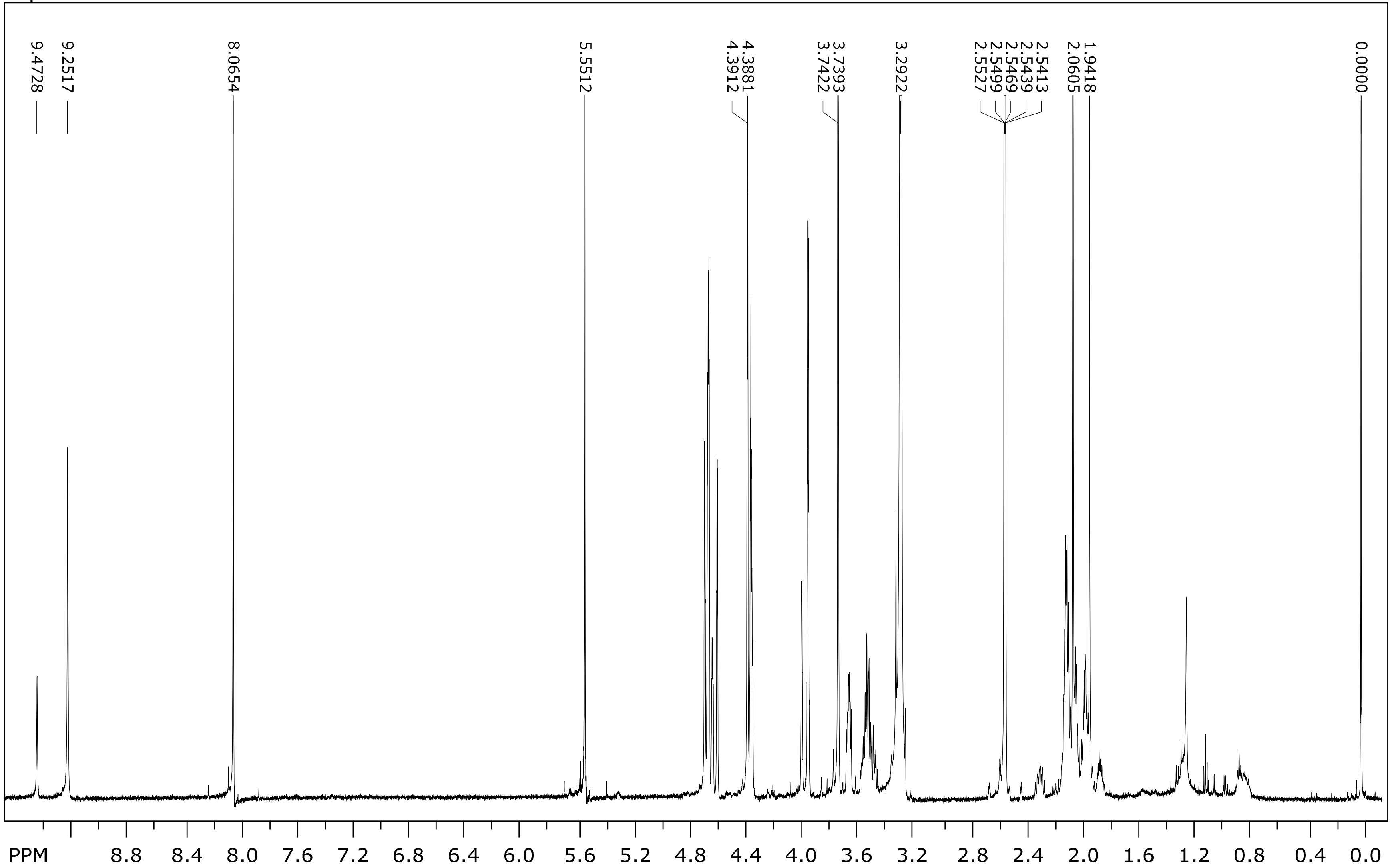

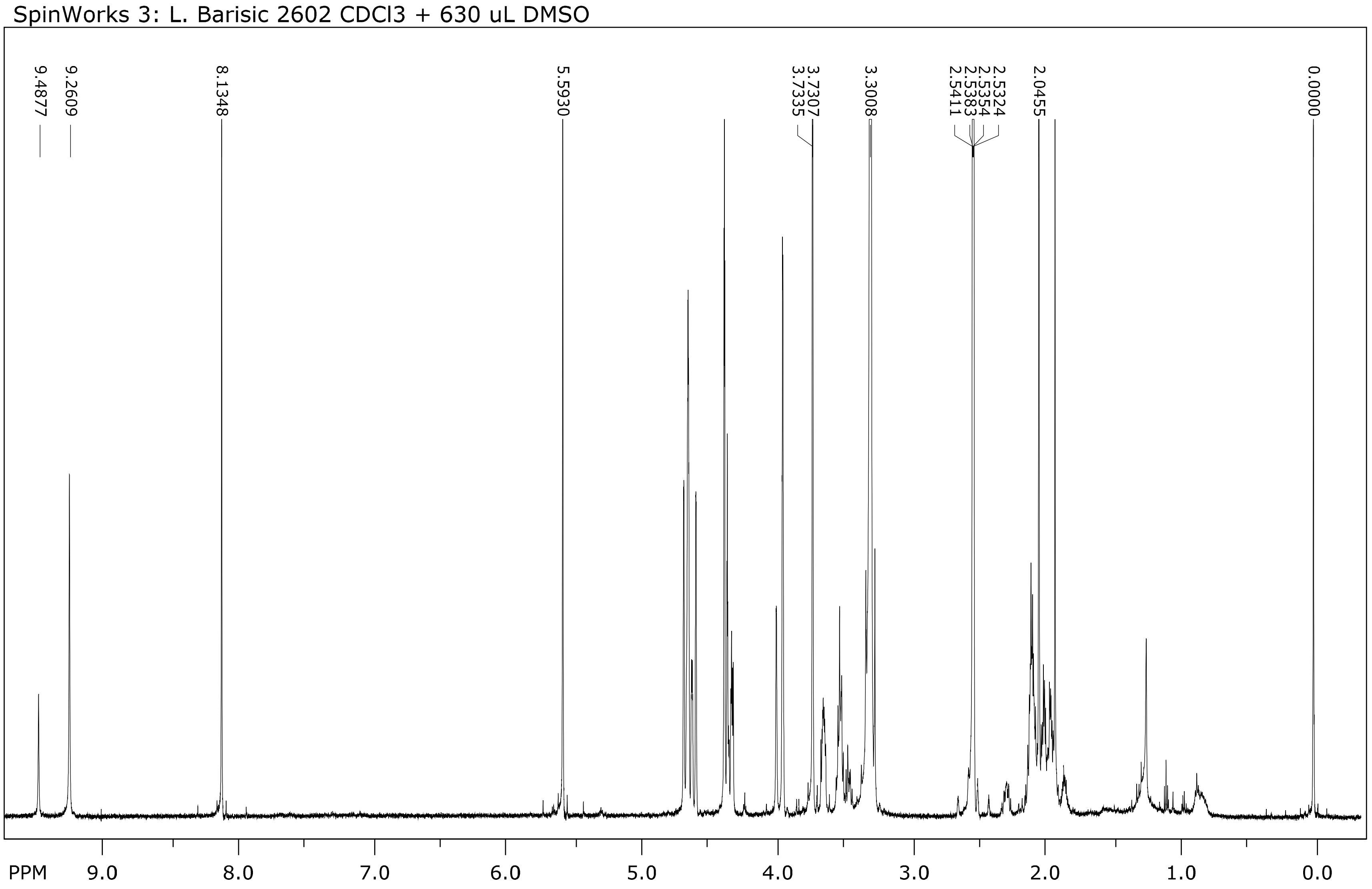
SpinWorks 3: L. Barisic 2602 CDCl3 + 630 uL DMSO
9.4877
9.2609
8.1348
5.5930
3.7335
3.7307
3.3008
2.5411
2.5383
2.5354
2.5324
2.0455
0.0000
PPM
9.0
8.0
7.0
6.0
5.0
4.0
3.0
2.0
1.0
0.0

Figure S20. Variable-temperature ^{1}H NMR spectra of **4**.

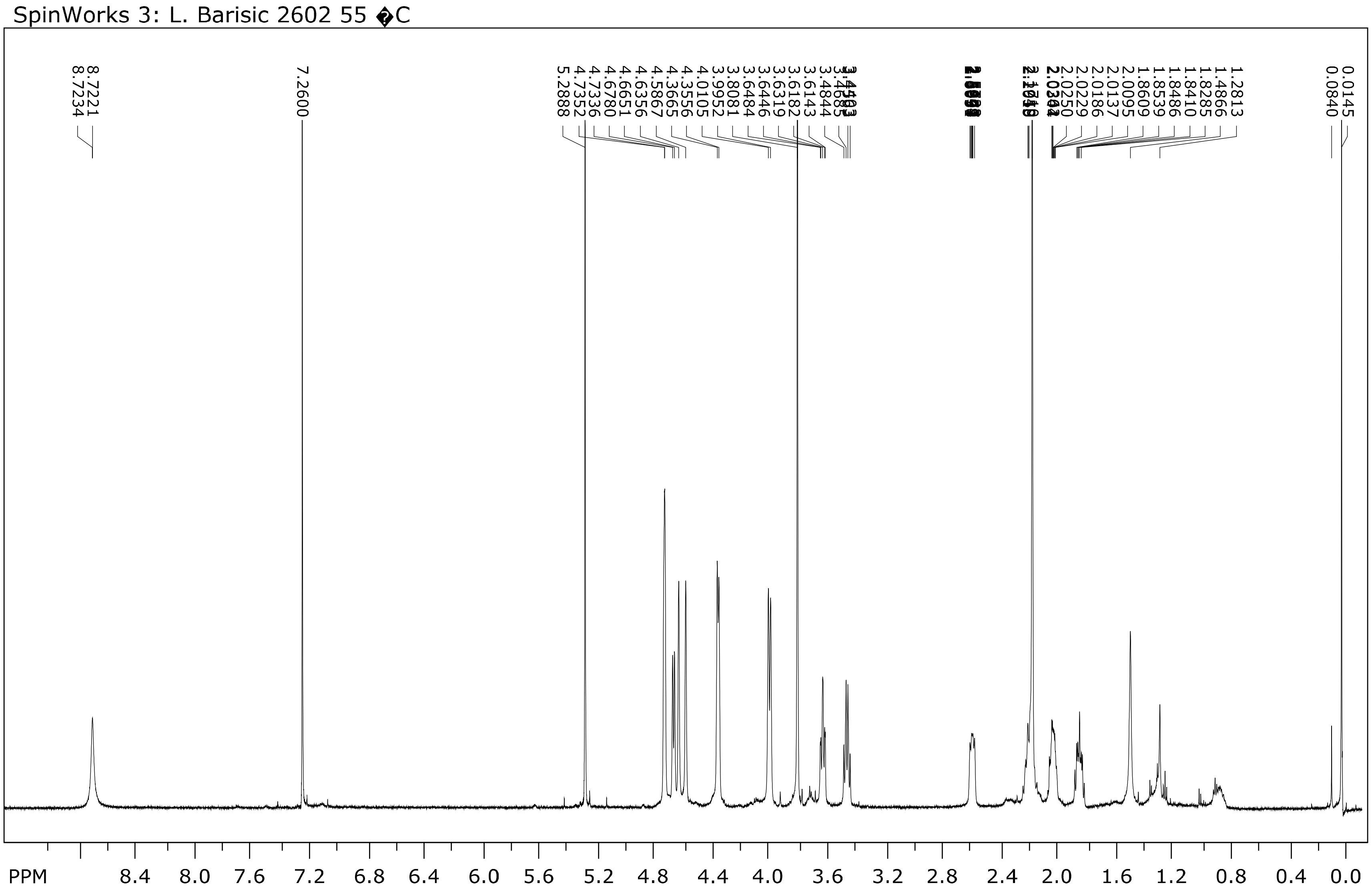

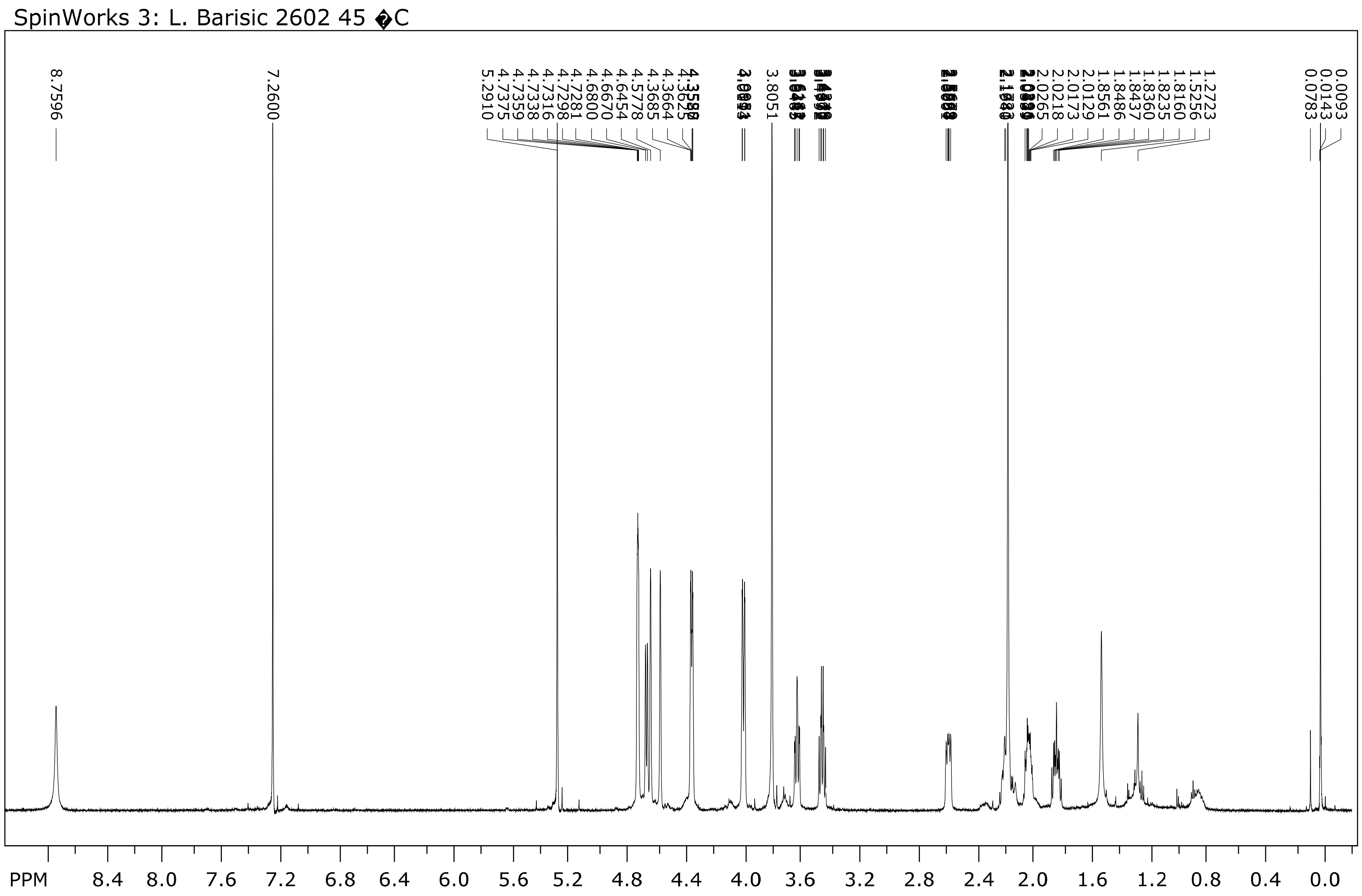
SpinWorks 3: L. Barisic 2602 45 �C
PPM
8.4 8.0 7.6 7.2 6.8 6.4 6.0 5.6 5.2 4.8 4.4 4.0 3.6 3.2 2.8 2.4 2.0 1.6 1.2 0.8 0.4 0.0

SpinWorks 3: L. Barisic 2602 35 �C

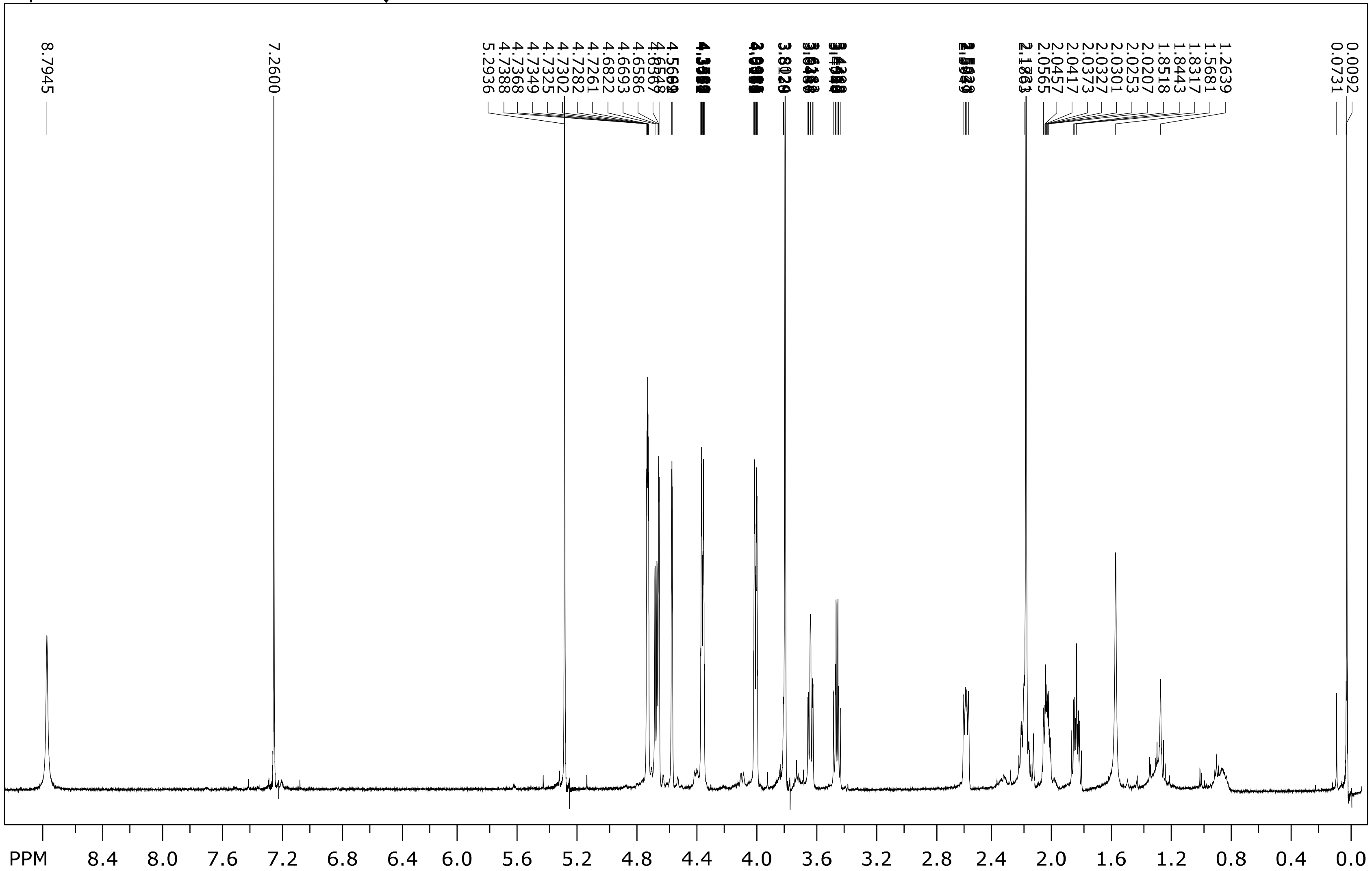

SpinWorks 3: L. Barisic 2602 25 �C

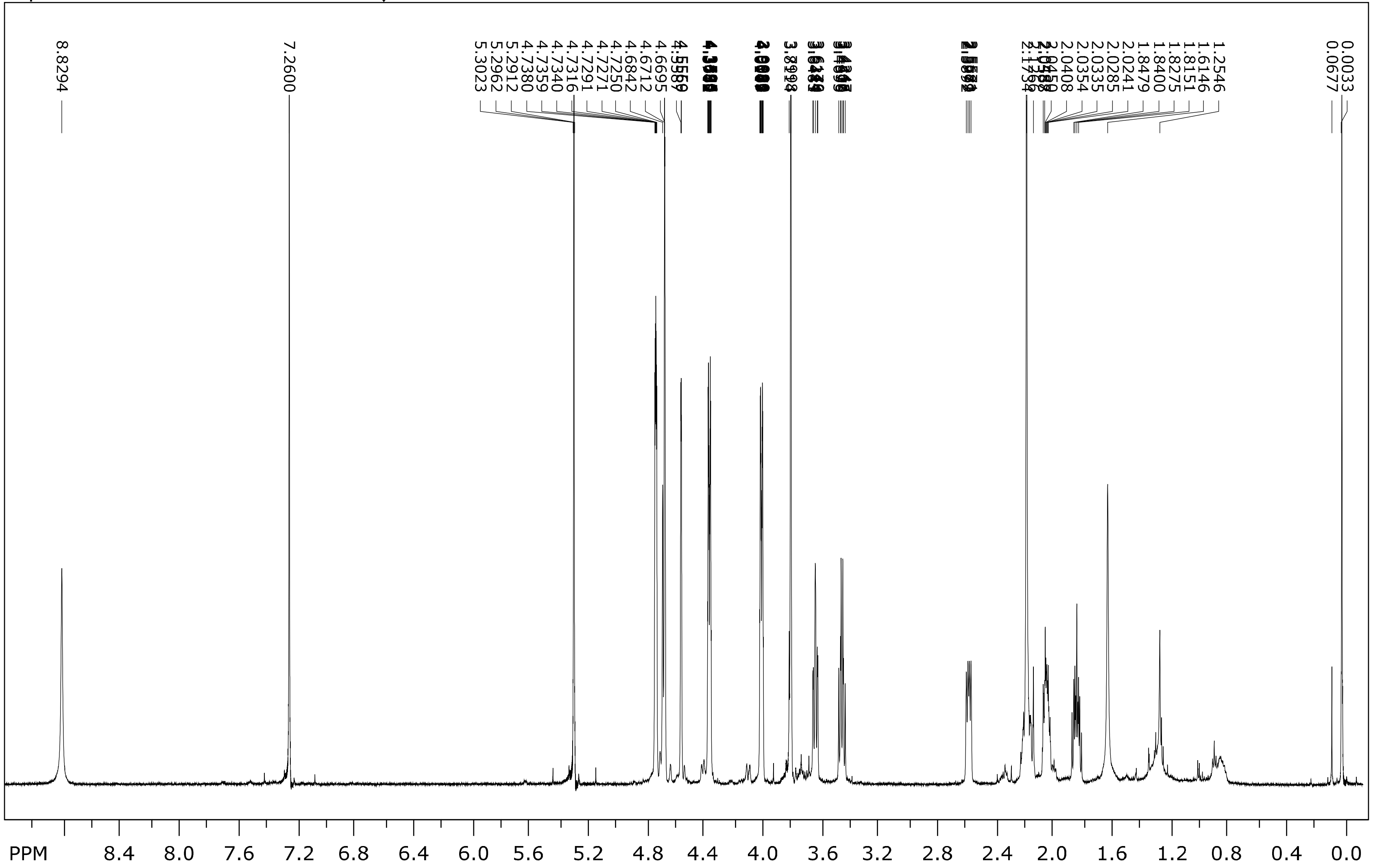

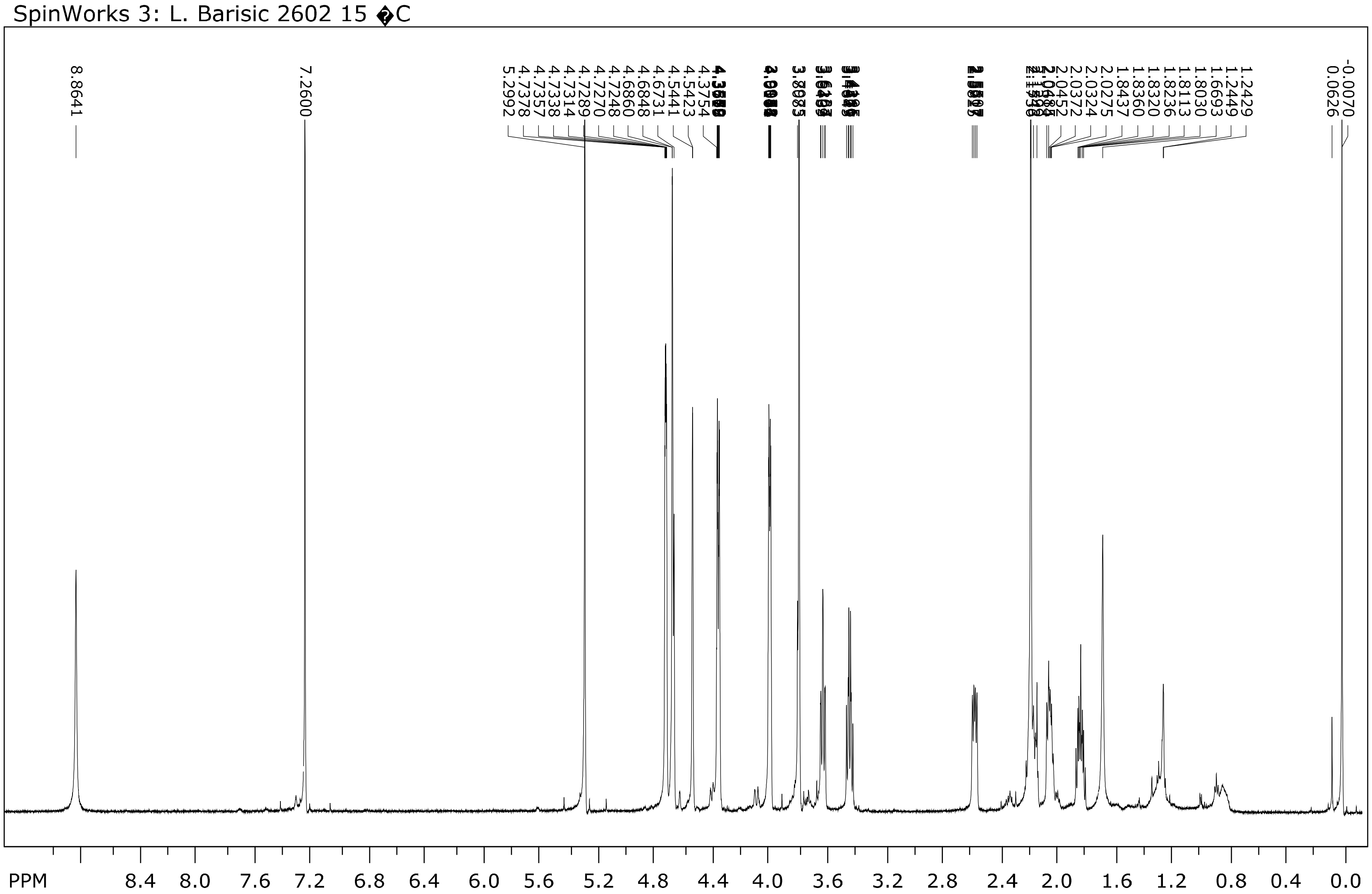
SpinWorks 3: L. Barisic 2602 15 �C
8.8641
7.2600
5.2992
4.7378
4.7357
4.7338
4.7314
4.7289
4.7270
4.7248
4.6860
4.6848
4.6731
4.5441
4.5423
4.3754
3.8085
2.0452
2.0372
2.0324
2.0275
1.8437
1.8360
1.8320
1.8236
1.8113
1.8030
1.6693
1.2449
1.2429
0.0626
-0.0070
PPM
8.4
8.0
7.6
7.2
6.8
6.4
6.0
5.6
5.2
4.8
4.4
4.0
3.6
3.2
2.8
2.4
2.0
1.6
1.2
0.8
0.4
0.0

SpinWorks 3: L. Barisic 2602 5 �C

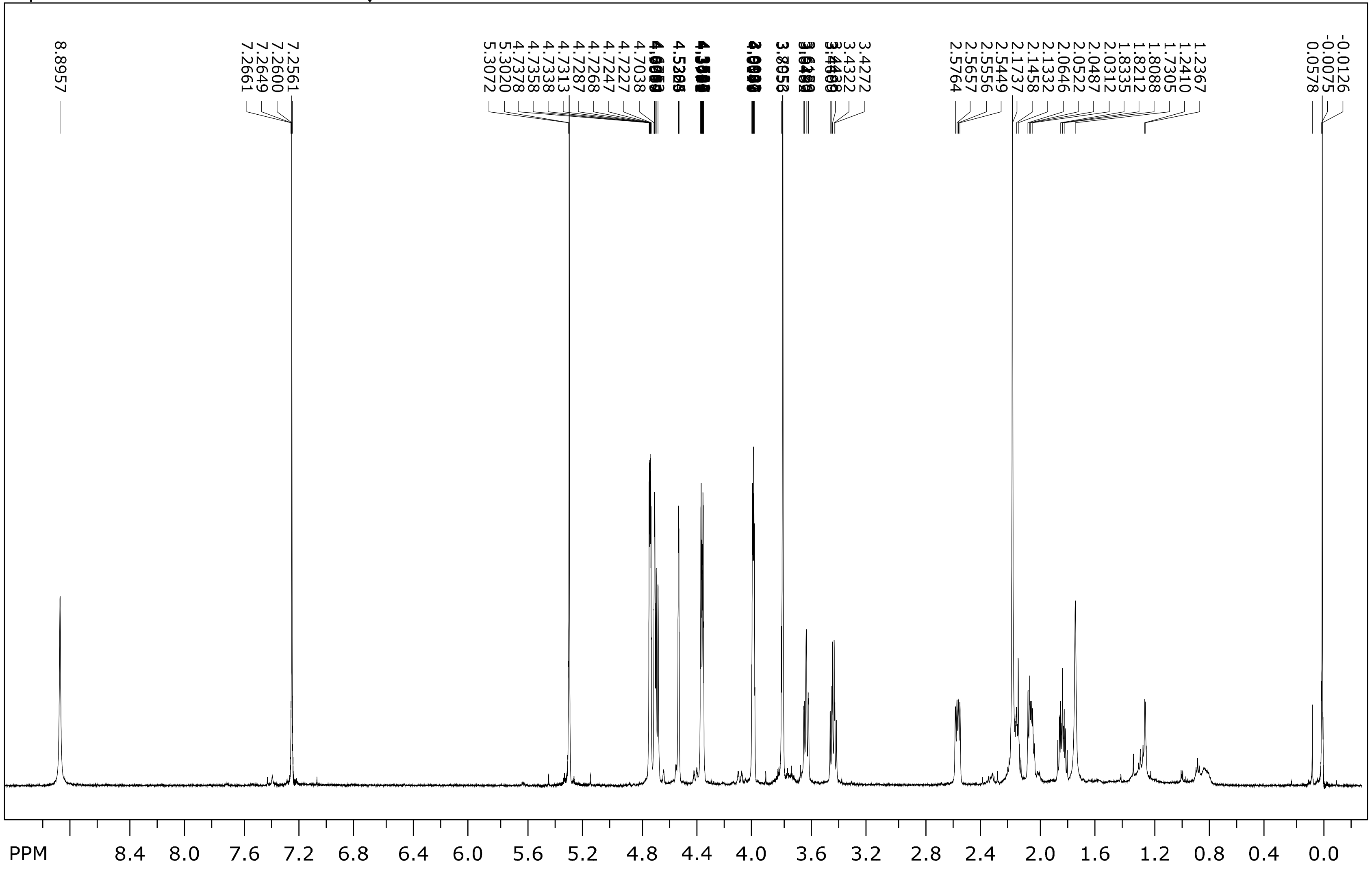

SpinWorks 3: L. Barisic 2602 -5 �C

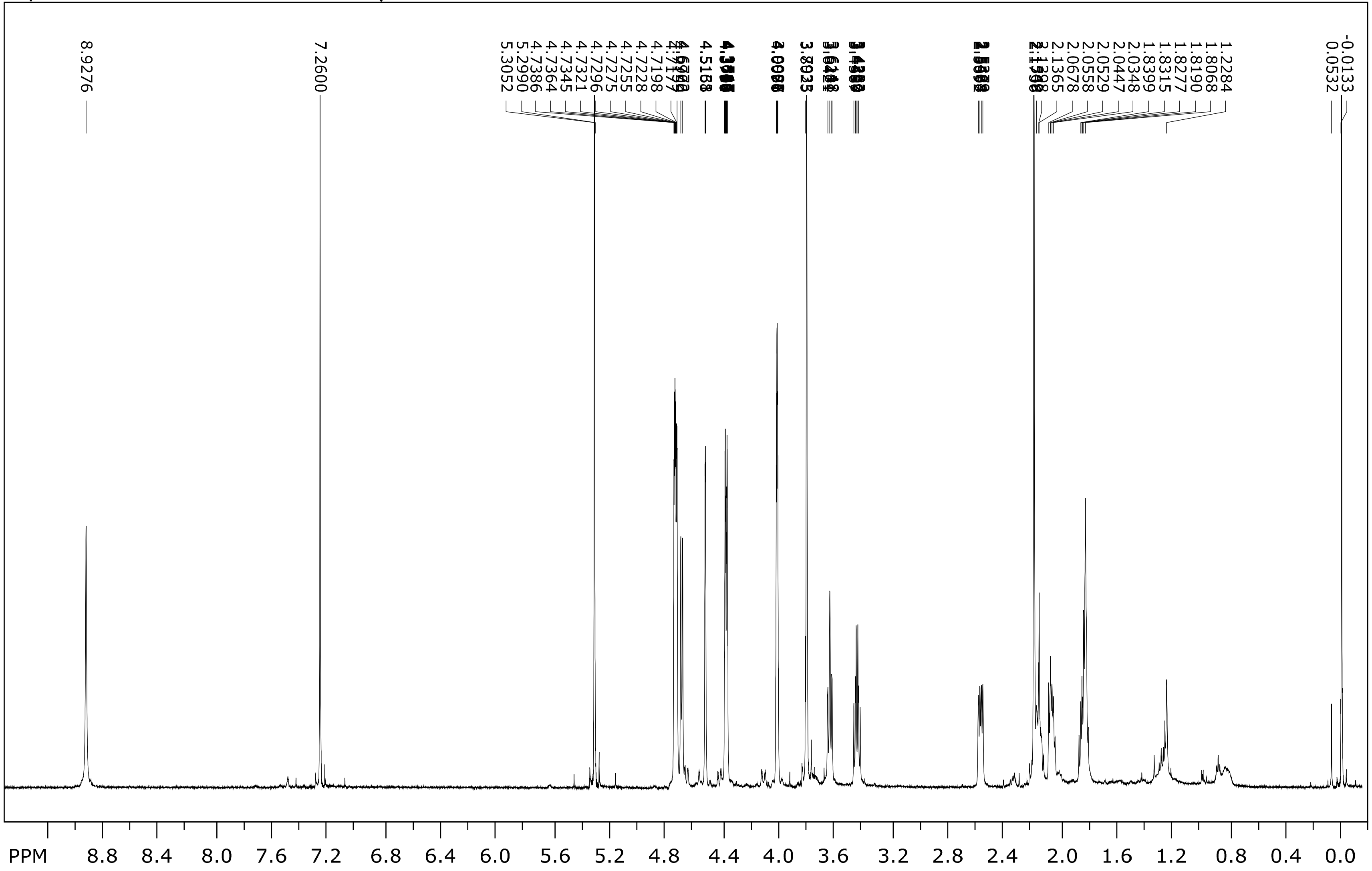

SpinWorks 3: L. Barisic 2602 -15 �C

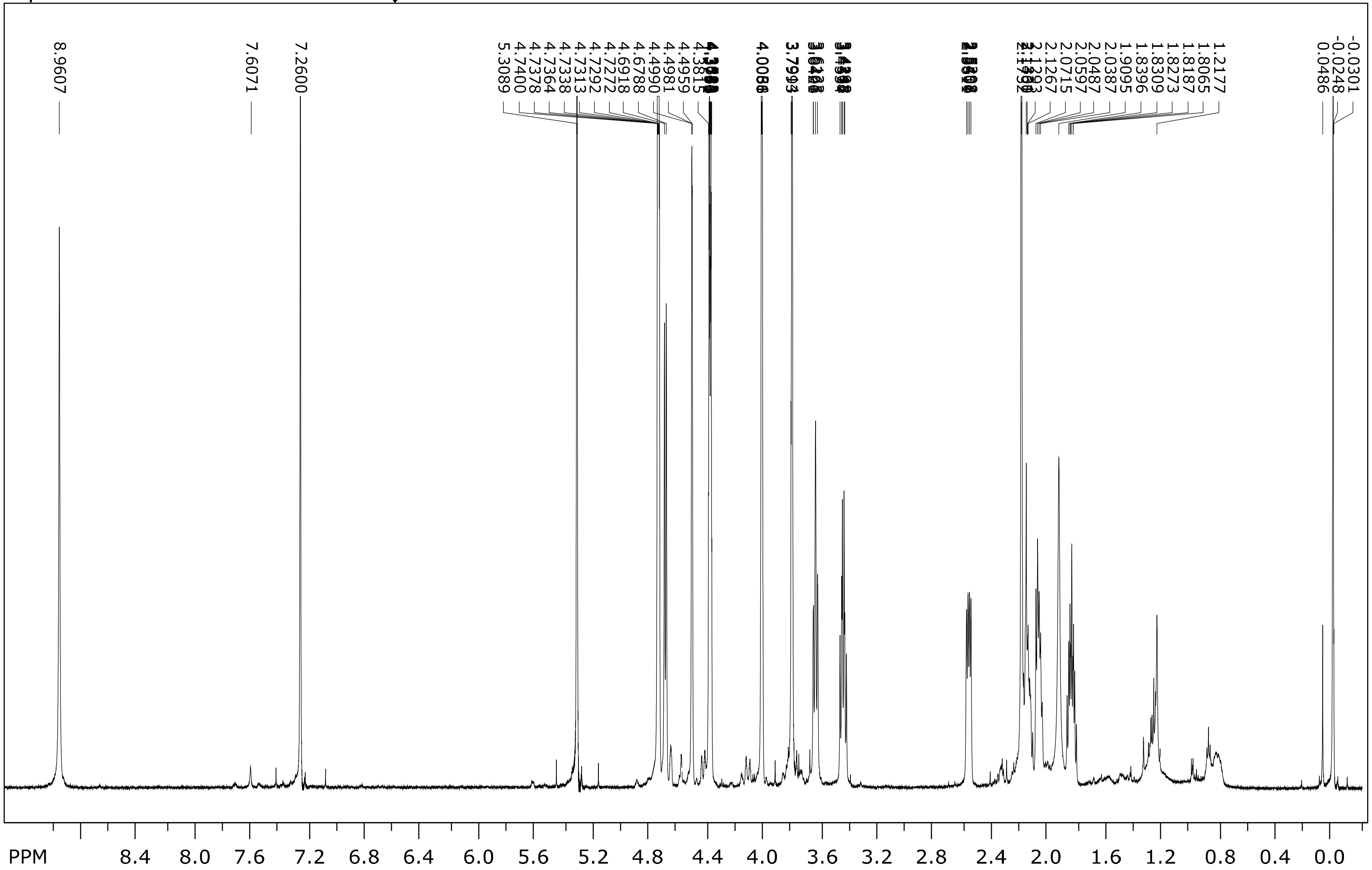

Figure S21. CheckCIF_PLATON report of compound **3**.

checkCIF/PLATON report

Structure factors have been supplied for datablock(s) I

THIS REPORT IS FOR GUIDANCE ONLY. IF USED AS PART OF A REVIEW PROCEDURE FOR PUBLICATION, IT SHOULD NOT REPLACE THE EXPERTISE OF AN EXPERIENCED CRYSTALLOGRAPHIC REFEREE.

No syntax errors found. CIF dictionary Interpreting this report

Datablock: I

```
Bond precision:   C-C = 0.0140 A          Wavelength=1.54179

Cell:             a=10.951(5)     b=8.571(5)          c=11.944(5)
                  alpha=90        beta=107.315(5)     gamma=90
Temperature:      293 K

                 Calculated                  Reported
Volume           1070.3(9)                   1070.3(9)
Space group      P 21                        P 1 21 1
Hall group       P 2yb                       ?
Moiety formula   C22 H28 Fe N2 O5            C22 H28 Fe1 N2 O5
Sum formula      C22 H28 Fe N2 O5            C22 H28 Fe N2 O5
Mr               456.31                      456.31
Dx,g cm-3        1.416                       1.416
Z                2                           2
Mu (mm-1)        5.954                       5.954
F000             480.0                       480.0
F000'            479.11
h,k,lmax         13,10,15                    13,10,14
Nref             4462[ 2385]                 3430
Tmin,Tmax        0.579,0.551                 0.251,1.000
Tmin'            0.525

Correction method= MULTI-SCAN

Data completeness= 1.44/0.77       Theta(max)= 75.860

R(reflections)= 0.1024( 3108)      wR2(reflections)= 0.2806( 3430)

S = 1.268                  Npar= Npar = 271
```

The following ALERTS were generated. Each ALERT has the format
test-name_ALERT_alert-type_alert-level.
Click on the hyperlinks for more details of the test.

Alert level C

```
ABSTY02_ALERT_1_C  An _exptl_absorpt_correction_type has been given without
            a literature citation. This should be contained in the
            _exptl_absorpt_process_details field.
            Absorption correction given as multi-scan
RFACG01_ALERT_3_C  The value of the R factor is > 0.10
            R factor given    0.102
RFACR01_ALERT_3_C  The value of the weighted R factor is > 0.25
            Weighted R factor given    0.281
PLAT084_ALERT_3_C High wR2 Value (i.e. > 0.25) ...................      0.28 Why ?
PLAT090_ALERT_3_C Poor Data / Parameter Ratio (Zmax > 18) ........      8.68 Note
PLAT125_ALERT_4_C No '_symmetry_space_group_name_Hall' Given .....    Please Do !
PLAT147_ALERT_1_C su on Symmetry Constrained Cell Angle(s) .......    Please Check
PLAT242_ALERT_2_C Low       Ueq as Compared to Neighbors for .....       C12 Check
PLAT341_ALERT_3_C Low Bond Precision on  C-C Bonds ...............    0.0140 Ang.
PLAT915_ALERT_3_C Low Friedel Pair Coverage ......................        52 %
```

Alert level G

```
PLAT005_ALERT_5_G No _iucr_refine_instructions_details  in the CIF    Please Do !
PLAT007_ALERT_5_G Number of Unrefined Donor-H Atoms ..............         1 Why ?
PLAT072_ALERT_2_G SHELXL First  Parameter in WGHT Unusually Large.      0.20 Why ?
PLAT093_ALERT_1_G No su's on H-positions, refinement reported as .     mixed
PLAT199_ALERT_1_G Reported _cell_measurement_temperature ..... (K)       293 Check
PLAT200_ALERT_1_G Reported   _diffrn_ambient_temperature ..... (K)       293 Check
PLAT791_ALERT_4_G The Model has Chirality at C7       .............        S Verify
PLAT912_ALERT_4_G Missing # of FCF Reflections Above STh/L=  0.600        31 Note
```

0 **ALERT level A** = Most likely a serious problem - resolve or explain
0 **ALERT level B** = A potentially serious problem, consider carefully
10 **ALERT level C** = Check. Ensure it is not caused by an omission or oversight
8 **ALERT level G** = General information/check it is not something unexpected

5 ALERT type 1 CIF construction/syntax error, inconsistent or missing data
2 ALERT type 2 Indicator that the structure model may be wrong or deficient
6 ALERT type 3 Indicator that the structure quality may be low
3 ALERT type 4 Improvement, methodology, query or suggestion
2 ALERT type 5 Informative message, check

Publication of your CIF

You should attempt to resolve as many as possible of the alerts in all categories. Often the minor alerts point to easily fixed oversights, errors and omissions in your CIF or refinement strategy, so attention to these fine details can be worthwhile. In order to resolve some of the more serious problems it may be necessary to carry out additional measurements or structure refinements. However, the nature of your study may justify the reported deviations from journal submission requirements and the more serious of these should be commented upon in the discussion or experimental section of a paper or in the "special_details" fields of the CIF. *checkCIF* was carefully designed to identify outliers and unusual parameters, but every test has its limitations and alerts that are not important in a particular case may appear. Conversely, the absence of alerts does not guarantee there are no aspects of the results needing attention. It is up to the individual to critically assess their own results and, if necessary, seek expert advice.

If you wish to submit your CIF for publication in Acta Crystallographica Section C or E, you should upload your CIF via the web. If your CIF is to form part of a submission to another IUCr journal, you will be asked, either during electronic submission or by the Co-editor handling your paper, to upload your CIF via our web site.

PLATON version of 05/02/2014; check.def file version of 05/02/2014

Datablock I - ellipsoid plot

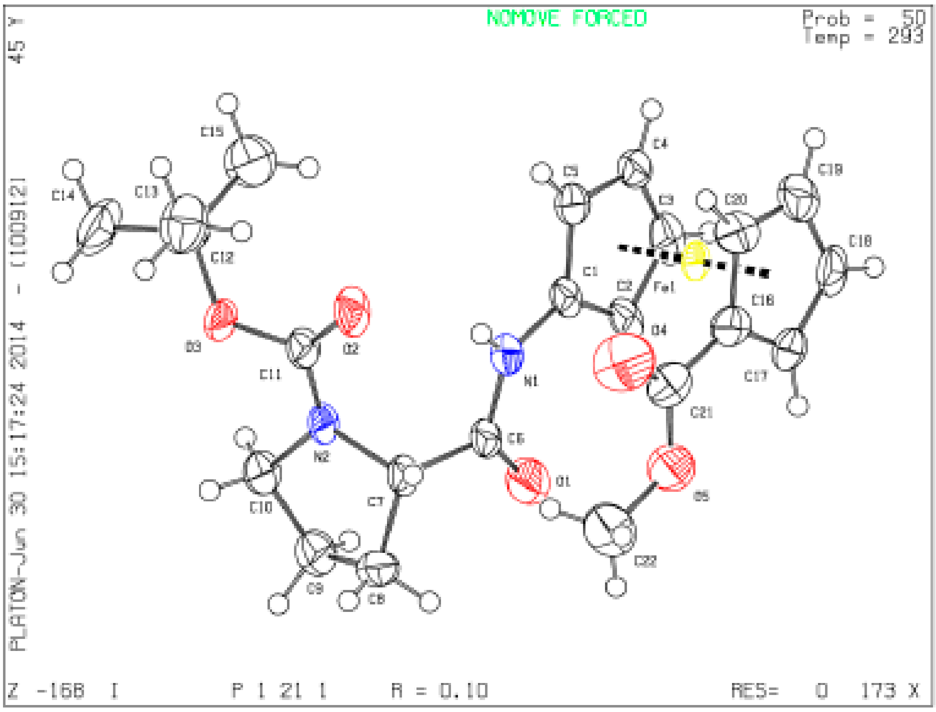

Supplement: Supplementary File 1 [file molecules-19-12852-s001.pdf]
